# Relação Causal entre Características das Células Sanguíneas e Doença Cardíaca Valvar: Um Estudo de Randomização Mendeliana com Duas Amostras

**DOI:** 10.36660/abc.20250063

**Published:** 2026-04-14

**Authors:** Xiaoli Wu, Yufeng Li, Jinyu Tian

**Affiliations:** 1 Department of Ultrasound Panzhihua Central Hospital Panzhihua China Department of Ultrasound - Panzhihua Central Hospital, Panzhihua – China

**Keywords:** Células Sanguíneas, Doenças das Valvas Cardíacas, Insuficiência da Valva Mitral, Análise da Randomização Mendeliana

## Abstract

**Fundamento:**

A patogênese da doença cardíaca valvar (DCV) permanece incerta, o que destaca a necessidade de mais pesquisas sobre os fatores de risco.

**Objetivos:**

Explorar as possíveis relações causais entre as características das células sanguíneas e quatro tipos de doenças valvares cardíacas: doença cardíaca reumática, doença da válvula mitral, doença da válvula aórtica e doença da válvula tricúspide.

**Métodos:**

Este estudo utilizou estatísticas resumidas de GWAS em larga escala e uma abordagem de randomização mendeliana de duas amostras, analisando treze características de células sanguíneas como exposições e quatro fenótipos de DCV como desfechos. O principal método de randomização mendeliana foi a variância inversa ponderada (IVW), complementada pelos métodos MR-Egger, mediana ponderada e moda ponderada, com extensas análises de sensibilidade para verificar a confiabilidade dos resultados, onde um valor de p < 0,05 foi considerado estatisticamente significativo.

**Resultados:**

Dentre as treze características das células sanguíneas examinadas, a contagem de neutrófilos apresentou associação causal positiva com doença da valva mitral (OR = 1,46, IC 95%: 1,16-1,85, p < 0,001) utilizando o método IVW. Esse achado foi corroborado pelo método MR-Egger (OR = 1,73, IC 95%: 1,09-2,75, p = 0,02), reforçando a evidência dessa relação causal. Para essa análise, 253 variantes genéticas robustas foram utilizadas como variáveis instrumentais. Adicionalmente, o volume corpuscular médio (VCM) apresentou associação causal negativa com doença da valva tricúspide (OR = 0,65, IC 95%: 0,44-0,96, p = 0,03) utilizando o método IVW, com base em 268 variáveis instrumentais. Outras características das células sanguíneas não apresentaram relações causais significativas com a DCV. Uma análise de sensibilidade abrangente corroborou a robustez dos nossos resultados.

**Conclusões:**

Os resultados deste estudo sugerem um possível envolvimento causal da contagem de neutrófilos e do VCM no desenvolvimento de doenças da válvula mitral e da válvula tricúspide, respectivamente.

## Graphical abstract

**Figura Central:**
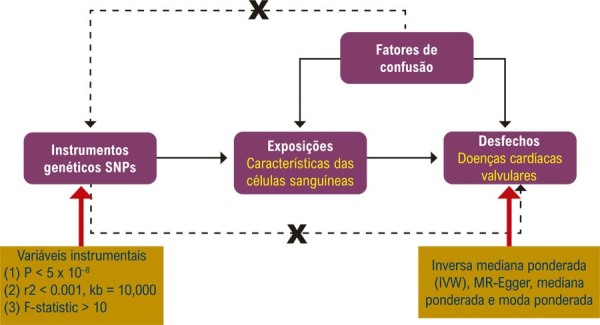
Relação Causal entre Características das Células Sanguíneas e Doença Cardíaca Valvar: Um Estudo de Randomização Mendeliana com Duas Amostras

Descrição do desenho do estudo nesta análise de RM.

## Introdução

A doença cardíaca valvar (DCV) é uma categoria de distúrbios cardíacos decorrentes do comprometimento da estrutura e função das válvulas cardíacas, compreendendo doença da válvula aórtica, doença da válvula mitral, doença da válvula tricúspide e doença cardíaca reumática.^1^ Assim como a doença arterial coronariana, as doenças valvares compartilham muitos fatores de risco e mecanismos patobiológicos relacionados à inflamação, disfunção endotelial e calcificação.^2^ Devido à crescente prevalência de outras doenças cardiovasculares e ao envelhecimento da população mundial, a valvopatia cardíaca se tornou uma causa importante de eventos cardiovasculares adversos, contribuindo para o ônus econômico global.^3^ Mesmo com os avanços no diagnóstico e tratamento da DCV, sua patogênese subjacente permanece não totalmente compreendida, o que ressalta a necessidade de pesquisas adicionais sobre os potenciais fatores de risco e mecanismos causais.

As células sanguíneas humanas (CSH) são vitais para o transporte de oxigênio, hemostasia e respostas imunes.^4^ As doenças cardiovasculares são influenciadas pela inflamação e pelo estresse oxidativo, que podem se refletir nas características das CSH.^5^ Estudos têm explorado a relação entre características das células sanguíneas e desfechos cardiovasculares.^6^ Níveis elevados de glóbulos brancos, particularmente neutrófilos, têm sido associados a um risco cardiovascular aumentado.^7^ Os neutrófilos contribuem para a aterosclerose e a desestabilização da placa.^8^ Os monócitos estão envolvidos na inflamação e remodelação vascular, afetando o risco de doenças cardiovasculares.^9^ Características dos glóbulos vermelhos (hemácias), como volume corpuscular médio (VCM), concentração de hemoglobina e amplitude de distribuição dos glóbulos vermelhos (RDW), têm sido investigadas em doenças cardiovasculares.^10^ Níveis baixos de VCM e hemoglobina podem aumentar o risco cardiovascular devido à redução do fornecimento de oxigênio.^11^ Níveis elevados de RDW têm sido associados a desfechos cardiovasculares adversos.^12^ Biomarcadores, incluindo hs-CRP, RDW e parâmetros eritrocitários, têm demonstrado potencial como preditores de complicações em pacientes com doenças valvares.^13^

Apesar da investigação das associações entre características das células sanguíneas e diversas doenças cardiovasculares em vários estudos observacionais, as relações causais específicas entre essas características e os subtipos de DCV permanecem pouco compreendidas. Uma abordagem analítica poderosa, a randomização mendeliana (RM), utiliza variantes genéticas como variáveis instrumentais (VIs) para avaliar possíveis associações causais entre exposições e desfechos.^14^ As análises de RM aproveitam a alocação aleatória de variantes genéticas durante a formação dos gametas para abordar questões de causalidade reversa e minimizar fatores de confusão, fornecendo evidências mais robustas de causalidade em comparação com estudos observacionais tradicionais.^15^ Utilizando um delineamento de RM com duas amostras, investigamos neste estudo as associações causais entre 13 características das células sanguíneas e 4 tipos principais de DCV.

## Métodos

### Desenho do estudo

Este estudo, um estudo de RM com duas amostras, se baseia em três pressupostos de VIs.^16^ ( Figura Central ). Primeiro, pelo menos uma das exposições está associada à variante genética. Segundo, nenhum dos fatores de confusão de cada associação exposição-desfecho está associado à variante. Terceiro, dadas as exposições e os fatores de confusão, a variante não está relacionada ao desfecho. O relato deste estudo seguiu rigorosamente as diretrizes do *Reporting of Observational Studies in Epidemiology using Mendelian Randomization* (STROBE-MR).^17 , 18^

### Fontes de dados

Do *Blood Cell Consortium* (BCX), obtivemos estatísticas resumidas para o instrumento de exposição, extraídas de um recente estudo de associação genômica ampla (GWAS) em larga escala sobre características de células sanguíneas, que envolveu 563.085 indivíduos de ascendência europeia.^19^ A partir deste GWAS, obtivemos variantes genéticas associadas aos níveis circulantes de leucócitos, linfócitos, monócitos, neutrófilos, eosinófilos, basófilos, plaquetas, contagem de eritrócitos, hematócrito, hemoglobina, VCM, hemoglobina corpuscular média e concentração de hemoglobina corpuscular média. A aplicação de GWA rápido ou GWA GLMM rápido a 3000 características em 456.422 indivíduos de ascendência europeia com genotipagem em array e 49.960 indivíduos com sequenciamento de exoma completo do UK Biobank foi realizada por Jiang et al.^20^ Disponíveis para download no portal de dados GWAS rápidos (https://yanglab.westlake.edu.cn/data/ukb_fastgwa/imp_binary/), os dados resumidos do GWAS associados à DCV abrangem doença reumática das válvulas cardíacas (275 casos e 456.073 controles), doença da válvula mitral (889 casos e 455.459 controles), doença da válvula aórtica (557 casos e 455.791 controles) e doença da válvula tricúspide (135 casos e 456.213 controles). Os dados sobre doenças são derivados do UK Biobank e definidos com base no PheCode. As informações detalhadas de todos os dados do GWAS podem ser encontradas na Tabela Suplementar 1 .

Como os dados foram obtidos de GWAS publicamente disponíveis ou aprovados eticamente e que estão em conformidade com a Declaração de Helsinque, nenhuma aprovação ética adicional foi necessária.

### Seleção de VIs

Aplicando um conjunto uniforme de critérios, nosso estudo selecionou meticulosamente SNPs como VIs. Abrangendo diversas fases cruciais, o paradigma de seleção foi implementado. Durante a fase de iniciação, as VIs potenciais foram identificadas como SNPs que apresentaram significância em todo o genoma (p < 5 × 10^−8^, R^2^ < 0,001, kb = 10.000).^21^ Em seguida, foi realizado um procedimento de agrupamento (R^2^ < 0,001, tamanho da janela = 10.000 kb) utilizando a função de agrupamento de dados, projetada para garantir que os SNPs selecionados não estivessem em desequilíbrio de ligação (DL) entre si.^22^ O valor da estatística F foi calculado para cada variável instrumental (VI) que apresentou correlação com a exposição. SNPs com valor F menor ou igual a 10 foram excluídos da análise para garantir que apenas VIs com associações fortes fossem selecionadas.^23^

Antes da análise de RM, realizamos um procedimento de harmonização para garantir que o efeito de cada SNP na exposição e no desfecho correspondesse ao mesmo alelo de efeito. Removemos SNPs palindrômicos com frequências alélicas intermediárias para evitar ambiguidade. As estimativas de efeito para todas as características das células sanguíneas foram escalonadas para representar um aumento por desvio padrão (DP), garantindo que as razões de chance para DCV sejam interpretadas de forma consistente em diferentes exposições.

### Análise estatística

Neste estudo, foram empregados múltiplos métodos complementares para explorar a relação causal entre as características das células sanguíneas e a doença valvar cardíaca, incluindo o método de variância inversa ponderada (IVW), a abordagem da mediana ponderada, a regressão MR-Egger e a moda ponderada, que foram utilizados como métodos de análise primária para estimativas causais. O método IVW, empregado como análise primária para estimativas causais, apresentou a maior precisão quando todas as VIs eram válidas.^24^ Quando pelo menos metade dos SNPs serviu como VIs eficazes, a abordagem da mediana ponderada estimou consistentemente o efeito causal.^23^ Utilizada para confirmar a presença de pleiotropia horizontal entre as variáveis independentes, a regressão MR-Egger fornece uma estimativa do efeito da pleiotropia horizontal por meio de seu intercepto.^25^ Como análises complementares, foi realizada a análise de moda ponderada.

Análises de sensibilidade adicionais foram conduzidas para avaliar a confiabilidade de nossas descobertas, nas quais o intercepto MR-Egger foi empregado para identificar pleiotropia horizontal direcional.^26^ Em seguida, o teste de soma residual de pleiotropia por RM e detecção de outliers (MR-PRESSO) foi utilizado para identificar a pleiotropia horizontal potencial e, posteriormente, corrigi-la por meio da eliminação de outliers.^27^ A avaliação da heterogeneidade entre os SNPs foi realizada utilizando o teste Q de Cochran.^28^ A investigação sobre se um único SNP influenciava a relação causal genética entre as exposições e os desfechos foi conduzida utilizando a análise leave-one-out.^29^ Utilizando o software R (versão 4.3.1), todas as análises estatísticas foram realizadas com os pacotes “TwoSampleMR” e “MR-PRESSO”. Um valor de p bicaudal < 0,05 foi considerado estatisticamente significativo para as estimativas causais primárias.

## Resultados

### Seleção de VIs

Como exposições, foram avaliadas 13 características de células sanguíneas, enquanto 4 fenótipos de DCV foram considerados como desfechos. Identificamos um conjunto de variantes genéticas robustas para servirem como VIs para cada par exposição-desfecho após a aplicação de critérios rigorosos de seleção de VIs (Tabela Suplementar 2). Todas as VIs têm um valor F superior a 10, indicando que eram suficientemente fortes para evitar viés de instrumento fraco. Todos os SNPs foram encontrados nos desfechos; nenhum SNP substituto foi utilizado. Detalhes sobre o número de SNPs são fornecidos na Tabela Suplementar 3.

### Análises de RM

**Figura 1:**
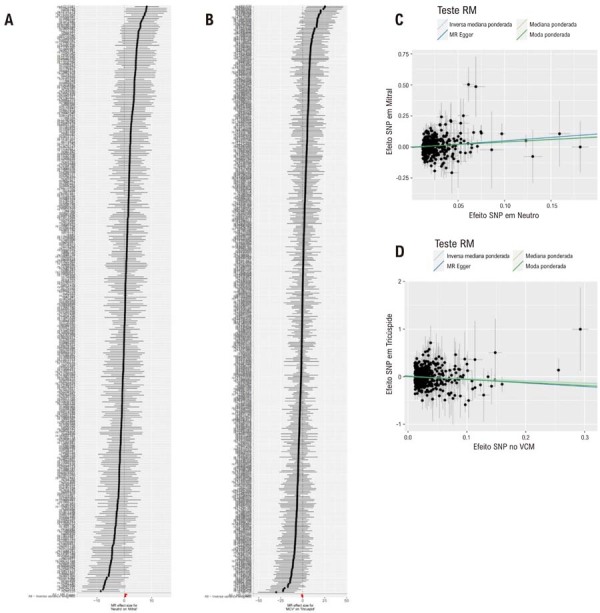
– Associações causais entre características das células sanguíneas e doença valvar cardíaca. Gráficos de floresta de A) contagem de neutrófilos na doença da válvula mitral; B) volume corpuscular médio na doença da válvula tricúspide. Gráficos de dispersão de C) contagem de neutrófilos na doença da válvula mitral; D) volume corpuscular médio na doença da válvula tricúspide.

A análise IVW primária revelou uma associação causal positiva geneticamente prevista entre uma contagem mais elevada de neut = ófilos e um risco aumentado de doença da válvula mitral ( Tabela 1 , Figura 1A ). Este achado foi consistente com os resultados do método MR-Egger, reforçando a evidência para esta relação causal ( Tabela 1 ).

**Tabela 1:** – Associação entre características das células sanguíneas e DCV

Não	Exposição	Desfecho	N.SNPs	Métodos	OR (IC 95%)	ou_lci95	ou_uci95	Valor de p
1	Baso	Mitral	152	MR. Egger	1,69	0,82	3,48	0,16
			152	Mediana ponderada	1,55	0,87	2,75	0,13
			152	IVW	1,27	0,89	1,81	0,19
			152	Moda ponderada	1,53	0,74	3.17	0,25
2	Eosino		383	MR. Egger	0,99	0,67	1,45	0,94
			383	Mediana ponderada	0,99	0,69	1,42	0,97
			383	IVW	0,97	0,79	1.19	0,77
			383	Moda ponderada	1.17	0,73	1,89	0,52
3	Linfático		398	MR.Egger	0,79	0,50	1,25	0,32
			398	Mediana ponderada	0,96	0,66	1,40	0,83
			398	IVW	1.01	0,81	1,25	0,92
			398	Moda ponderada	0,89	0,54	1,48	0,66
4	Mono		479	MR. Egger	1.06	0,80	1,40	0,69
			479	Mediana ponderada	0,84	0,61	1,15	0,27
			479	IVW	1,00	0,84	1.19	0,99
			479	Moda ponderada	1.02	0,74	1,41	0,91
5	Neutro		347	MR. Egger	1,73	1.09	2,75	0,02
			347	Mediana ponderada	1,49	0,94	2,34	0,09
			347	IVW	1,46	1.16	1,85	0,00
			347	Moda ponderada	1,50	0,90	2,48	0,12
6	Plaq		478	MR. Egger	0,91	0,67	1.21	0,51
			478	Mediana ponderada	0,94	0,70	1,27	0,71
			478	IVW	0,96	0,81	1.14	0,65
			478	Moda ponderada	0,96	0,70	1,32	0,82
7	Hemácias		410	MR. Egger	1.03	0,70	1,53	0,87
			410	Mediana ponderada	1.04	0,71	1,51	0,86
			410	IVW	1.09	0,89	1,34	0,41
			410	Moda ponderada	0,90	0,62	1,30	0,57
8	Leucócitos		387	MR. Egger	1,93	1.19	3.13	0,01
			387	Mediana ponderada	1.18	0,79	1,77	0,42
			387	IVW	1.21	0,96	1,53	0,11
			387	Moda ponderada	1,30	0,79	2.16	0,30
9	Ht		338	MR.Egger	0,79	0,49	1,28	0,34
			338	Mediana ponderada	0,77	0,52	1,15	0,20
			338	IVW	0,89	0,69	1,15	0,38
			338	Moda ponderada	0,69	0,46	1.04	0,07
10	Hb		362	MR.Egger	0,88	0,58	1,35	0,56
			362	Mediana ponderada	0,90	0,60	1,34	0,59
			362	IVW	0,92	0,73	1,15	0,46
			362	Moda ponderada	0,85	0,56	1,29	0,44
11	VCM		430	MR.Egger	0,82	0,64	1.05	0,11
			430	Mediana ponderada	0,97	0,76	1,25	0,83
			430	IVW	0,94	0,80	1.10	0,43
			430	Moda ponderada	1.01	0,78	1,30	0,96
12	MCH		400	MR.Egger	0,95	0,73	1,24	0,72
			400	Mediana ponderada	0,99	0,74	1,32	0,93
			400	IVW	0,93	0,79	1.09	0,38
			400	Moda ponderada	1.12	0,83	1,51	0,45
13	MCHC		187	MR.Egger	1.01	0,64	1,58	0,97
			187	Mediana ponderada	0,93	0,61	1,43	0,74
			187	IVW	0,87	0,66	1.14	0,30
			187	Moda ponderada	0,97	0,64	1,48	0,90
1	Baso	Tricúspide	152	MR.Egger	1.02	0,16	6,48	0,99
			152	Mediana ponderada	1.06	0,26	4,33	0,93
			152	IVW	0,76	0,30	1,88	0,55
			152	Moda ponderada	1,51	0,23	9,70	0,67
2	Eosino		383	MR.Egger	0,93	0,34	2,52	0,89
			383	Mediana ponderada	0,43	0,19	1,00	0,05
			383	IVW	0,78	0,46	1,30	0,34
			383	Moda ponderada	0,44	0,14	1,32	0,14
3	Linfático		398	MR.Egger	0,76	0,24	2,42	0,64
			398	Mediana ponderada	0,79	0,30	2.09	0,64
			398	IVW	1.09	0,63	1,90	0,75
			398	Moda ponderada	0,80	0,21	3.02	0,74
4	Mono		479	MR.Egger	1,38	0,68	2,81	0,38
			479	Mediana ponderada	1,33	0,61	2,92	0,48
			479	IVW	1,25	0,80	1,94	0,32
			479	Moda ponderada	1,36	0,64	2,89	0,43
5	Neutro		347	MR.Egger	0,85	0,27	2,70	0,79
			347	Mediana ponderada	0,60	0,20	1,78	0,36
			347	IVW	1.19	0,66	2.12	0,57
			347	Moda ponderada	0,79	0,22	2,77	0,71
6	Plaq		478	MR.Egger	0,67	0,32	1,40	0,29
			478	Mediana ponderada	1.09	0,54	2.20	0,80
			478	IVW	0,86	0,57	1.31	0,49
			478	Moda ponderada	1,24	0,51	2,98	0,63
7	Hemácias		410	MR.Egger	3,27	1.22	8,74	0,02
			410	Mediana ponderada	2,44	0,95	6.26	0,06
			410	IVW	1,45	0,87	2,43	0,16
			410	Moda ponderada	3.11	1.11	8,68	0,03
8	Leucócitos		387	MR.Egger	0,91	0,28	2,99	0,87
			387	Mediana ponderada	0,74	0,28	1,94	0,54
			387	IVW	1,29	0,73	2.29	0,38
			387	Moda ponderada	0,74	0,23	2,37	0,61
9	Ht		338	MR.Egger	1,24	0,39	3,95	0,72
			338	Mediana ponderada	1.21	0,42	3,52	0,72
			338	IVW	0,76	0,41	1,39	0,37
			338	Moda ponderada	1,58	0,45	5,52	0,47
10	Hb		362	MR.Egger	1,38	0,47	4.02	0,56
			362	Mediana ponderada	1.13	0,40	3.14	0,82
			362	IVW	0,76	0,42	1,36	0,36
			362	Moda ponderada	1.08	0,34	3,45	0,90
11	VCM		430	MR.Egger	0,48	0,26	0,90	0,02
			430	Mediana ponderada	0,61	0,31	1,20	0,16
			430	IVW	0,65	0,44	0,96	0,03
			430	Moda ponderada	0,54	0,27	1.08	0,08
12	MCH		400	MR.Egger	0,61	0,31	1.18	0,14
			400	Mediana ponderada	0,52	0,24	1.10	0,09
			400	IVW	0,75	0,50	1.12	0,16
			400	Moda ponderada	0,54	0,23	1,25	0,15
13	MCHC		187	MR.Egger	1,27	0,40	4.08	0,69
			187	Mediana ponderada	1,26	0,39	4.05	0,70
			187	IVW	1.17	0,58	2,35	0,67
			187	Moda ponderada	1,34	0,44	4.07	0,61

IVW: variância inversa ponderada; VCM: volume corpuscular médio; MCH: hemoglobina corpuscular média; MCHC: concentração de hemoglobina corpuscular média; Ht: hematócrito; Hb: hemoglobina; Plaq: plaquetas.

Além disso, nossa análise identificou uma relação causal negativa geneticamente prevista entre o VCM e o risco de doença da válvula tricúspide usando o método IVW ( Tabela 1 ). O gráfico de floresta ( Figura 1B ) representou visualmente as estimativas causais obtidas a partir de VIs individuais. Embora a mediana ponderada não tenha atingido significância estatística (OR = 0,61, IC 95%: 0,31-1,20, p = 0,16) ( Tabela 1 ), a estimativa de MR-Egger corroborou essa associação (OR = 0,48, IC 95%: 0,26-0,90, p = 0,02) (Tabela= 1). Não houve evidências robustas de associações causais para as demais características das células sanguíneas e a DCV ( Tabela 1 ). O gráfico de dispersão ( Figura 1C -D) visualizou os tamanhos de efeito estimados para os SNPs ligados às características das células sanguíneas que afetam a DCV.

### Análises de sensibilidade

Foram realizadas diversas análises de sensibilidade para avaliar a robustez de nossas descobertas e a validade das premissas da RM. Para as associações entre a contagem de neutrófilos e a doença da válvula mitral, e entre o VCM e a doença da válvula tricúspide, o teste Q de Cochran não indicou heterogeneidade significativa ( Tabela 2 ). O teste de intercepto MR-Egger não mostrou evidências de pleiotropia horizontal direcional, sugerindo que é improvável que nossos resultados sejam enviesados por efeitos pleiotrópicos ( Tabela 2 ). Além disso, não foram identificados valores discrepantes para as associações entre contagem de neutrófilos e doença da válvula mitral e entre VCM e doença da válvula tricúspide pelo teste global MR-PRESSO, exceto para o par contagem de neutrófilos e doença da válvula aórtica, onde um valor discrepante foi detectado (Tabela Suplementar 4). A remoção desse valor discrepante não afetou a consistência da estimativa causal (OR = 1,13, IC 95%: 0,83-1,52, p = 0,44) (Tabela Suplementar 4). Os gráficos de funil excluíram os vieses de publicação ( Figura Suplementar 1A-B ), enquanto as análises de exclusão de um por vez confirmaram que as estimativas causais não foram impulsionadas por nenhum SNP influente isolado, reforçando ainda mais a robustez de nossas descobertas ( Figura Suplementar 1C-D ).

**Tabela 2:** – Resultados do teste de heterogeneidade e do teste de pleiotropia das variáveis instrumentais

Exposição	Desfecho	Heterogeneidade		Pleiotropia	
		Estatísticas Q (IVW)	Valor de p	Intercepto MR-Egger	Valor de p
Baso	Reumático	150,13	0,50	0,028	0,101
Eosino		399,23	0,26	-0,003	0,810
Linfático		389,36	0,60	-0,004	0,679
Mono		514,38	0,12	0,006	0,447
Neutro		319,04	0,85	0,018	0,105
Plaq		480,83	0,44	0,001	0,930
Hemácias		440,09	0,14	-0,008	0,449
Leucócitos		440,60	0,03	-0,017	0,133
Ht		337,87	0,48	0,020	0,063
Hb		343,67	0,74	-0,001	0,937
VCM		425,15	0,54	0,007	0,387
MCH		446,84	0,05	0,020	0,033
MCHC		184,43	0,52	0,005	0,698
Baso	Mitral	143,74	0,65	-0,008	0,378
Eosino		331,83	0,97	-0,001	0,929
Linfático		373,00	0,80	0,007	0,234
Mono		481,11	0,45	-0,002	0,623
Neutro		367,19	0,21	-0,005	0,407
Plaq		507,78	0,16	0,002	0,623
Hemácias		429,80	0,23	0,002	0,750
Leucócitos		438,76	0,03	-0,014	0,033
Ht		385,16	0,04	0,004	0,567
Hb		370,77	0,35	0,001	0,838
VCM		449,36	0,24	0,007	0,166
MCH		407,96	0,37	-0,001	0,823
MCHC		165,32	0,86	-0,006	0,409
Baso	Aórtico	163,77	0,23	-0,006	0,619
Eosino		400,81	0,24	-0,004	0,547
Linfático		384,74	0,66	-0,004	0,603
Mono		498,01	0,25	-0,005	0,323
Neutro		393,65	0,04	0,000	0,954
Plaq		469,41	0,59	-0,009	0,134
Hemácias		448,82	0,08	-0,012	0,102
Leucócitos		425,29	0,08	0,000	0,982
Ht		324,57	0,68	-0,007	0,380
Hb		382,41	0,21	-0,009	0,214
VCM		441,29	0,33	-0,008	0,197
MCH		391,36	0,60	0,006	0,339
MCHC		165,56	0,86	0,009	0,319
Baso	Tricúspide	139,49	0,74	-0,009	0,721
Eosino		358,88	0,80	-0,006	0,672
Linfático		366,22	0,86	0,011	0,479
Mono		453,54	0,78	-0,004	0,725
Neutro		321,54	0,82	0,010	0,517
Plaq		452,79	0,78	0,010	0,418
Hemácias		389,81	0,74	-0,026	0,058
Leucócitos		398,53	0,32	0,010	0,507
Ht		331,10	0,58	-0,015	0,337
Hb		350,72	0,64	-0,018	0,194
VCM		416,95	0,65	0,014	0,221
MCH		395,75	0,54	0,010	0,438
MCHC		189,32	0,42	-0,003	0,859

IVW: variância inversa ponderada; VCM: volume corpuscular médio; MCH: hemoglobina corpuscular média; MCHC: concentração de hemoglobina corpuscular média; Ht: hematócrito; Hb: hemoglobina; Plaq: plaquetas.

## Discussão

Este estudo abrangente de RM com duas amostras investigou as potenciais relações causais entre 13 características das células sanguíneas e os 4 principais tipos de DCV: doença cardíaca reumática, doença da válvula mitral, doença da válvula aórtica e doença da válvula tricúspide. Nossos achados corroboram fortemente uma associação causal positiva entre a contagem de neutrófilos e o risco de doença da válvula mitral, bem como uma associação causal negativa entre o VCM e o risco de doença da válvula tricúspide. Nossos resultados, que demonstram consistência em múltiplos métodos de RM e robustez em análises de sensibilidade rigorosas, destacam a força e a confiabilidade dessas associações.

Nosso estudo revelou uma relação causal positiva entre a contagem de neutrófilos e o risco de doença da válvula mitral, consistente com o papel estabelecido da inflamação na patogênese da DCV.^30^ Os neutrófilos, como mediadores-chave da inflamação, provavelmente contribuem para o desenvolvimento de lesões valvares por meio de vários mecanismos, incluindo disfunção endotelial, estresse oxidativo e remodelamento da matriz extracelular.^31 , 32^ Esses resultados sugerem que a inflamação mediada por neutrófilos pode desempenhar um papel crucial no início e na progressão da doença da válvula mitral, destacando potenciais alvos terapêuticos nas vias inflamatórias impulsionadas por neutrófilos.

Curiosamente, não encontramos evidências de uma associação causal entre a contagem de neutrófilos e o risco de doença cardíaca reumática ou doença da valva aórtica. Esse efeito diferencial nos subtipos de DCV pode ser explicado por mecanismos patogênicos distintos. A doença cardíaca reumática é impulsionada principalmente por linfócitos e mecanismos mediados por anticorpos,^33^ enquanto a doença valvar aórtica calcificada está mais intimamente ligada à deposição de lipídios e às vias de sinalização osteogênicas.^34 , 35^

Outra descoberta intrigante é a associação causal negativa entre o VCM e o risco de doença da valva tricúspide. Um VCM baixo, indicativo de anemia microcítica, tem sido associado a diversas doenças cardiovasculares devido ao aumento do estresse oxidativo e à redução do transporte de oxigênio.^10 , 11^ Os mecanismos subjacentes a essa relação causal podem envolver disfunção endotelial, danos valvares e respostas imunes alteradas.^36 - 38^

Nosso estudo não encontrou evidências robustas de associações causais entre outras características das células sanguíneas e os subtipos de DCV. Isso sugere que os papéis causais das características das células sanguíneas na patogênese da DCV podem ser específicos para certos tipos celulares ou subtipos da doença. Alternativamente, outros fatores não mensurados podem desempenhar papéis mais dominantes nessas condições de DCV.

As potenciais implicações terapêuticas dessas descobertas merecem uma análise cuidadosa. Embora o direcionamento direto dos níveis de neutrófilos ou do VCM possa não ser viável na prática clínica, explorar abordagens para modular as vias subjacentes pode levar a novas estratégias terapêuticas para o tratamento da doença valvar cardíaca.

É crucial destacar as limitações do nosso estudo e a necessidade de interpretar os resultados com a devida cautela. Primeiro, como em todos os estudos de RM, nossos resultados podem ser influenciados por pleiotropia biológica não contabilizada, embora tenhamos nos esforçado para avaliar e mitigar rigorosamente essa possibilidade por meio de métodos complementares de RM e análises de sensibilidade. Segundo, uma limitação importante do nosso estudo é que as estatísticas resumidas do GWAS foram derivadas exclusivamente de indivíduos de ascendência europeia. Consequentemente, nossos resultados podem não ser generalizáveis para populações de outras ascendências, onde as arquiteturas genéticas e os fatores ambientais podem diferir. Pesquisas futuras em diversos grupos ancestrais são essenciais para validar essas relações causais globalmente. Terceiro, nossos resultados oferecem informações valiosas sobre os potenciais papéis causais da contagem de neutrófilos e do VCM na patogênese da DCV, mas as vias celulares e moleculares específicas envolvidas permanecem especulativas e requerem investigação adicional. Quarto, embora algumas características patológicas (como estenose e regurgitação) sejam de grande interesse e cruciais para a compreensão da DCV, não encontramos dados suficientes para esses subtipos nos recursos de GWAS disponíveis. Consequentemente, não conseguimos analisar esses importantes fenótipos patológicos, o que limita nossa capacidade de explorar plenamente suas associações causais. Pesquisas futuras devem se concentrar na coleta de dados de GWAS mais detalhados sobre os subtipos de DCV e no aprimoramento do registro detalhado das diferentes características patológicas. Em quinto lugar, existe um potencial de sobreposição de amostras entre os conjuntos de dados GWAS de exposição e desfecho, o que pode introduzir viés em análises de RM de duas amostras, particularmente quando as VIs são fracas. No entanto, as VIs selecionadas para nossas análises primárias apresentaram estatísticas F significativamente superiores a 10, qualificando-se, portanto, como instrumentos fortes. Consequentemente, a probabilidade de tal sobreposição influenciar substancialmente nossos resultados é mínima. Não obstante, futuras investigações utilizando subconjuntos de dados não sobrepostos são necessárias para mitigar ainda mais os potenciais vieses. Além disso, os fenótipos de DCV neste estudo foram definidos usando PheCodes, que podem incluir condições clinicamente heterogêneas. Por exemplo, a doença valvar mitral pode abranger tanto estenose quanto regurgitação, condições com patologias distintas. Devido à ausência de dados GWAS disponíveis publicamente para esses subtipos específicos, não foi possível realizar análises de sensibilidade em fenótipos mais granulares. Essa heterogeneidade fenotípica pode obscurecer ou diluir efeitos causais específicos de cada subtipo, destacando a necessidade de estudos futuros com definições de desfecho mais precisas. Apesar dessas limitações, nossas descobertas contribuem para o crescente conjunto de evidências que relacionam características das células sanguíneas à patogênese de doenças cardiovasculares. A identificação de possíveis papéis causais da contagem de neutrófilos e do VCM na doença da válvula mitral e na doença da válvula tricúspide, respectivamente, fornece novas perspectivas e incentiva uma investigação mais aprofundada dos mecanismos biológicos subjacentes.

## Conclusão

Este estudo abrangente de RM com duas amostras lança luz sobre as associações causais entre características específicas das células sanguíneas e certos subtipos de DCV. A relação causal positiva entre a contagem de neutrófilos e o risco de doença da valva mitral, bem como a associação causal negativa entre o VCM e o risco de doença da valva tricúspide, ressaltam a importância de considerar essas características das células sanguíneas como potenciais fatores de risco e alvos terapêuticos no contexto da DCV. Essas descobertas abrem caminho para futuras pesquisas com o objetivo de traduzir esses conhecimentos em estratégias aprimoradas de prevenção, detecção precoce e tratamento da DCV, além de inaugurar novas perspectivas para a exploração dos mecanismos biológicos subjacentes.

## *Material suplementar

Supplementary material Para Figura Suplementar, por favor, clique aqui. https://abccardiol.org/supplementary-material/2026/12303/2025-0063_supplementary_figure_01.pdf

Supplementary material Para Tabelas suplementares, por favor, clique aqui. https://abccardiol.org/supplementary-material/2026/12303/2025-0063_supplementary_tables_02.pdf

Supplementary material Para Tabela suplementar 3, por favor, clique aqui. https://abccardiol.org/supplementary-material/2026/12303/2025-0063_supplementary_Table_03.pdf
