# Supplementary material for: Relação Causal entre Características das Células Sanguíneas e Doença Cardíaca Valvar: Um Estudo de Randomização Mendeliana com Duas Amostras
Source: Arq Bras Cardiol. 2026 Apr 14;123(3):e20250063. [Article in Portuguese] doi: 10.36660/abc.20250063 (PMC13128221; doi:10.36660/abc.20250063)
Supplement: Supplementary material [file 0066-782x-abc-123-3-e20250063-suppl01.pdf]

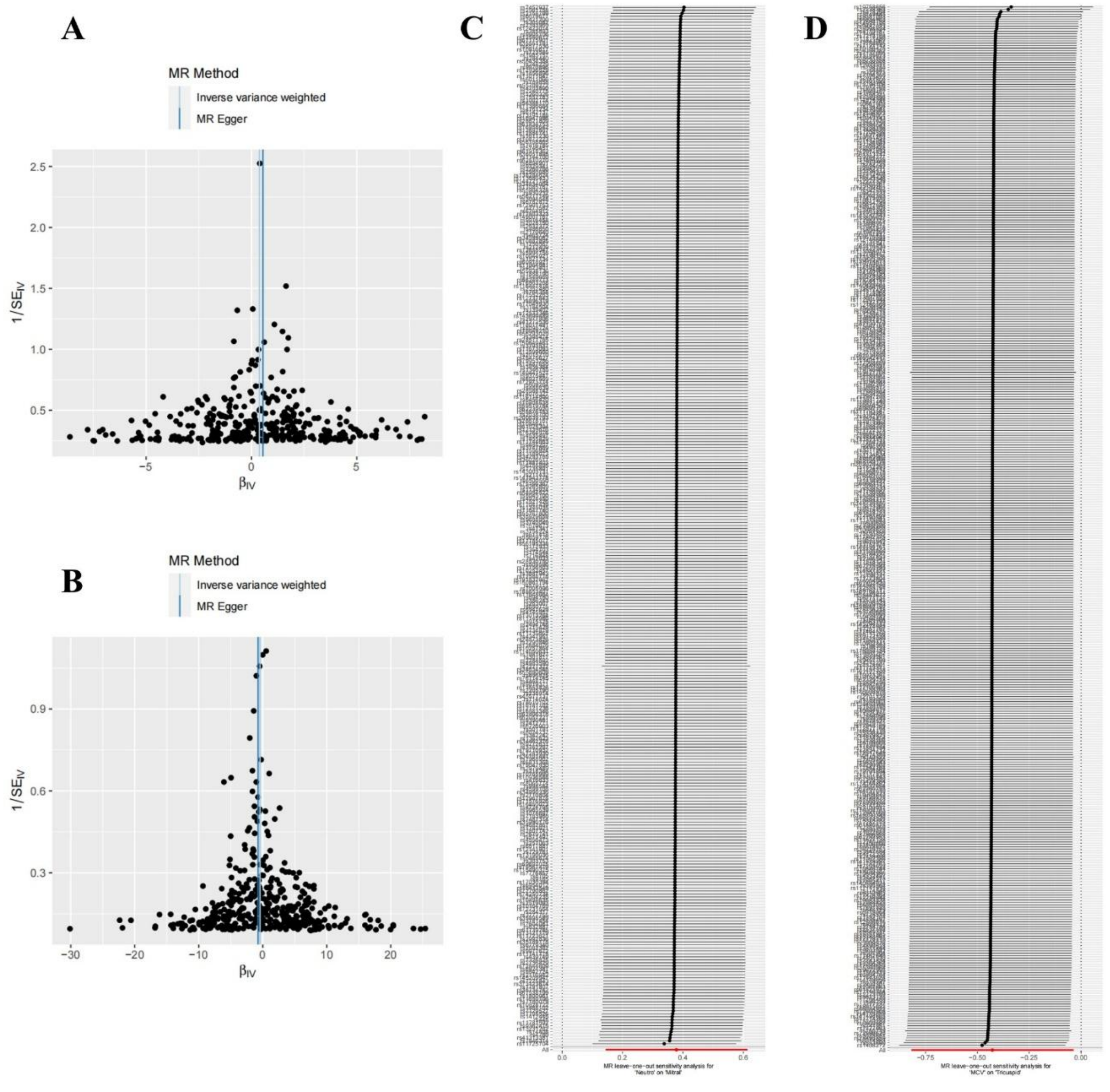

Supplementary Figure 1. Sensitivity analysis of blood cell traits on VHD. Funnel plots of A) neutrophil count on mitral valve disease; B) MCV on tricuspid valve disease. Leave-one-out plots of C) neutrophil count on mitral valve disease; D) MCV on tricuspid valve disease.
