# Supplementary material for: Relação Causal entre Características das Células Sanguíneas e Doença Cardíaca Valvar: Um Estudo de Randomização Mendeliana com Duas Amostras
Source: Arq Bras Cardiol. 2026 Apr 14;123(3):e20250063. [Article in Portuguese] doi: 10.36660/abc.20250063 (PMC13128221; doi:10.36660/abc.20250063)
Supplement: Supplementary material [file 0066-782x-abc-123-3-e20250063-suppl02.pdf]

Supplementary Table 1. Detailed information for the GWAS data.

**Character**	**Trait**	**GWAS ID**	**Publication**	**Consortium/Source**	**NSample sizes (cases/control)**	**N_SNPsAncestry**
Exposure	Basophil count（Baso）	GCST90002379			408,112	EuropeanNA
					408,112	NA
Exposure	Eosinophil counts（Eosino）	GCST90002381			408,112	NA
					408,112	NA
Exposure	Lymphocyte count（Lym）	GCST90002388			408,112	NA
			PMID: 32888494	the Blood Cell Consortium (BCX)	408,112	NA
Exposure	Monocyte count（Mono）	GCST90002393			408,112	NA
					408,112	NA
Exposure	Neutrophil count（Neutro）	GCST90002398			408,112	NA
					408,112	NA
Exposure	Platelet count（Plt）	GCST90002402			408,112	NA

Exposure	Red blood cell count (RBC)	GCST90002403	~~408,112~~	~~NA~~
Exposure	White blood cell count (WBC)	GCST90002407	~~408,112~~563,085	~~NA~~
Exposure	Hematocrit (Ht)	GCST90002383		~~NA~~
Exposure	Hemoglobin (Hb)	GCST90002384		~~NA~~
Exposure	Mean corpuscular volume (MCV)	GCST90002392		~~NA~~
Exposure	Mean corpuscular hemoglobin (MCH)	GCST90002390		~~NA~~
Exposure	Mean corpuscular	GCST90002391		

	hemoglobin concentration (MCHC)				
Outcome	Rheumatic disease of the heart valves	394_PheCode	PMID: 34737426		275/456,073
Outcome	Mitral valve disease	394.2_PheCode		the UK Biobank	889/455,459
Outcome	Aortic valve disease	394.3_PheCode			557/455,791
Outcome	Tricuspid valve disease	394.7_PheCode			135/456,213

Supplementary Table 2. The F value information of IVs in this study.

No	**Exposure**	N.SNPs	meanF	minF	maxF
1	Baso	152	89.26697	30.06259	611.7368
2	Eosino	383	121.5692	29.81901	2432.238
3	Lym	398	107.5933	29.78972	1922.207
4	Mono	479	174.8864	29.86109	5100.971
5	Neutro	347	100.517	29.83005	3129.775
6	Plt	478	148.1465	29.73643	3726.51
7	RBC	410	118.7615	29.96782	4251.907
8	WBC	387	105.2245	29.86061	2455.559
9	Ht	338	98.77513	29.87032	1911.299
10	Hb	362	101.3257	29.875	2246.582
11	MCV	430	197.8534	29.77239	4533.369
12	MCH	400	197.1222594	29.77638058	4528.146566
13	MCHC	187	129.9896	29.79632	2075.398

Supplementary Table 4. **MR-PRESSO test results.**

Exposure	Outcome	Raw				Outlier corrected				Global P
		OR (CI%)	lowCI	upCI	***P***	OR (CI%)	lowCI	upCI	***P***	
**Baso**	**Rheumatic**	1.08	0.57	2.05	0.81	NA	NA	NA	NA	0.34
**Eosino**		1.06	0.73	1.53	0.78	NA	NA	NA	NA	0.26
**Lym**		0.80	0.55	1.17	0.26	NA	NA	NA	NA	0.63
**Mono**		0.98	0.71	1.34	0.88	NA	NA	NA	NA	0.11
**Neutro**		1.21	0.83	1.78	0.33	NA	NA	NA	NA	0.88
**Plt**		0.94	0.70	1.27	0.70	NA	NA	NA	NA	0.43
**RBC**		0.96	0.66	1.38	0.81	NA	NA	NA	NA	0.18
**WBC**		1.00	0.67	1.50	0.98	NA	NA	NA	NA	0.05
**Ht**		0.87	0.57	1.32	0.50	NA	NA	NA	NA	0.53

**Hb**		0.84	0.57	1.25	0.39	NA	NA	NA	NA	0.70
**MCV**		0.90	0.69	1.18	0.46	NA	NA	NA	NA	0.57
**MCH**		0.98	0.73	1.32	0.91	NA	NA	NA	NA	0.06
**MCHC**		0.84	0.53	1.35	0.48	NA	NA	NA	NA	0.56
**Baso**	**Mitral**	1.21	0.86	1.69	0.27	NA	NA	NA	NA	0.72
**Eosino**		0.98	0.81	1.18	0.82	NA	NA	NA	NA	0.97
**Lym**		1.02	0.83	1.25	0.85	NA	NA	NA	NA	0.77
**Mono**		1.02	0.86	1.21	0.82	NA	NA	NA	NA	0.48
**Neutro**		1.42	1.13	1.78	0.00	NA	NA	NA	NA	0.29
**Plt**		0.95	0.80	1.12	0.52	NA	NA	NA	NA	0.13
**RBC**		1.09	0.89	1.34	0.42	NA	NA	NA	NA	0.16
**WBC**		1.20	0.96	1.50	0.12	NA	NA	NA	NA	0.05
**Ht**		0.90	0.70	1.15	0.39	NA	NA	NA	NA	0.04

**Hb**		0.92	0.73	1.16	0.48	NA	NA	NA	NA	0.40
**MCV**		0.94	0.81	1.10	0.46	NA	NA	NA	NA	0.26
**MCH**		0.94	0.80	1.10	0.44	NA	NA	NA	NA	0.33
**MCHC**		0.89	0.69	1.14	0.36	NA	NA	NA	NA	0.88
**Baso**	**Aortic**	1.06	0.67	1.67	0.82	NA	NA	NA	NA	0.17
**Eosino**		1.12	0.87	1.46	0.38	NA	NA	NA	NA	0.29
**Lym**		0.85	0.65	1.10	0.22	NA	NA	NA	NA	0.65
**Mono**		1.22	0.98	1.52	0.08	NA	NA	NA	NA	0.13
**Neutro**		1.19	0.88	1.61	0.26	1.13	0.83	1.52	0.44	0.02
**Plt**		0.99	0.80	1.21	0.90	NA	NA	NA	NA	0.52
**RBC**		0.87	0.67	1.13	0.29	NA	NA	NA	NA	0.10
**WBC**		1.20	0.90	1.59	0.21	NA	NA	NA	NA	0.09
**Ht**		0.93	0.69	1.24	0.60	NA	NA	NA	NA	0.68

**Hb**		0.91	0.68	1.23	0.55	NA	NA	NA	NA	0.15
**MCV**		0.97	0.80	1.18	0.76	NA	NA	NA	NA	0.29
**MCH**		1.00	0.82	1.22	0.99	NA	NA	NA	NA	0.58
**MCHC**		1.03	0.75	1.41	0.87	NA	NA	NA	NA	0.85
**Baso**	**Tricuspid**	0.81	0.34	1.90	0.62	NA	NA	NA	NA	0.77
**Eosino**		0.78	0.48	1.29	0.33	NA	NA	NA	NA	0.84
**Lym**		1.17	0.69	1.96	0.56	NA	NA	NA	NA	0.86
**Mono**		1.23	0.81	1.88	0.33	NA	NA	NA	NA	0.81
**Neutro**		1.24	0.72	2.16	0.44	NA	NA	NA	NA	0.85
**Plt**		0.84	0.56	1.26	0.40	NA	NA	NA	NA	0.74
**RBC**		1.42	0.86	2.33	0.17	NA	NA	NA	NA	0.76
**WBC**		1.33	0.76	2.32	0.32	NA	NA	NA	NA	0.35
**Ht**		0.77	0.43	1.40	0.39	NA	NA	NA	NA	0.61

**Hb**	0.79	0.45	1.39	0.41	NA	NA	NA	NA	0.60
**MCV**	0.66	0.45	0.97	0.04	NA	NA	NA	NA	0.56
**MCH**	0.76	0.51	1.14	0.19	NA	NA	NA	NA	0.55
**MCHC**	1.21	0.61	2.39	0.59	NA	NA	NA	NA	0.44
