# Supplementary material for: Relação Causal entre Características das Células Sanguíneas e Doença Cardíaca Valvar: Um Estudo de Randomização Mendeliana com Duas Amostras
Source: Arq Bras Cardiol. 2026 Apr 14;123(3):e20250063. [Article in Portuguese] doi: 10.36660/abc.20250063 (PMC13128221; doi:10.36660/abc.20250063)
Supplement: Supplementary material [file 0066-782x-abc-123-3-e20250063-suppl03.pdf]

exposure	outcome	id.exposure	id.outcome	samplesize	SNP	b	se	p
Baso	Mitral	ebi-a-GCST	BpI3Ho	424022	rs1002331	0,249054	0,181211	0,169321
Baso	Mitral	ebi-a-GCST	BpI3Ho	424022	rs1008656	0,229873	0,182795	0,208556
Baso	Mitral	ebi-a-GCST	BpI3Ho	424022	rs1021423	0,248203	0,181249	0,170874
Baso	Mitral	ebi-a-GCST	BpI3Ho	424022	rs1042916	0,252862	0,181086	0,162605
Baso	Mitral	ebi-a-GCST	BpI3Ho	424022	rs1043080	0,245058	0,181085	0,175968
Baso	Mitral	ebi-a-GCST	BpI3Ho	424022	rs1058900	0,2329	0,181252	0,198808
Baso	Mitral	ebi-a-GCST	BpI3Ho	424022	rs1077262	0,236463	0,181094	0,191639
Baso	Mitral	ebi-a-GCST	BpI3Ho	424022	rs1086893	0,18616	0,182105	0,306654
Baso	Mitral	ebi-a-GCST	BpI3Ho	424022	rs1089384	0,255721	0,181153	0,158059
Baso	Mitral	ebi-a-GCST	BpI3Ho	424022	rs1092707	0,207811	0,18486	0,260948
Baso	Mitral	ebi-a-GCST	BpI3Ho	424022	rs1095640	0,228183	0,181227	0,207995
Baso	Mitral	ebi-a-GCST	BpI3Ho	424022	rs1100575	0,25359	0,181491	0,162335
Baso	Mitral	ebi-a-GCST	BpI3Ho	424022	rs1106488	0,245868	0,181274	0,174994
Baso	Mitral	ebi-a-GCST	BpI3Ho	424022	rs1109778	0,241777	0,181179	0,182052
Baso	Mitral	ebi-a-GCST	BpI3Ho	424022	rs1109832	0,247819	0,181265	0,171574
Baso	Mitral	ebi-a-GCST	BpI3Ho	424022	rs1119595	0,237596	0,181113	0,189566
Baso	Mitral	ebi-a-GCST	BpI3Ho	424022	rs1125351	0,231474	0,183432	0,206983
Baso	Mitral	ebi-a-GCST	BpI3Ho	424022	rs1159154	0,253532	0,181601	0,162686
Baso	Mitral	ebi-a-GCST	BpI3Ho	424022	rs1170220	0,2346	0,181417	0,195959
Baso	Mitral	ebi-a-GCST	BpI3Ho	424022	rs1170538	0,204734	0,181757	0,259989
Baso	Mitral	ebi-a-GCST	BpI3Ho	424022	rs1177289	0,23488	0,182555	0,198225
Baso	Mitral	ebi-a-GCST	BpI3Ho	424022	rs1186222	0,255196	0,181812	0,16043
Baso	Mitral	ebi-a-GCST	BpI3Ho	424022	rs1193038	0,2318	0,181103	0,200568
Baso	Mitral	ebi-a-GCST	BpI3Ho	424022	rs1202636	0,240803	0,18109	0,183603
Baso	Mitral	ebi-a-GCST	BpI3Ho	424022	rs12075	0,240645	0,182109	0,186356
Baso	Mitral	ebi-a-GCST	BpI3Ho	424022	rs1212392	0,259125	0,182147	0,154848
Baso	Mitral	ebi-a-GCST	BpI3Ho	424022	rs1214361	0,229285	0,181158	0,205633
Baso	Mitral	ebi-a-GCST	BpI3Ho	424022	rs1227832	0,22865	0,18119	0,206972
Baso	Mitral	ebi-a-GCST	BpI3Ho	424022	rs1237651	0,240475	0,18115	0,184347
Baso	Mitral	ebi-a-GCST	BpI3Ho	424022	rs1245368	0,224168	0,181259	0,216189
Baso	Mitral	ebi-a-GCST	BpI3Ho	424022	rs1245941	0,251252	0,181197	0,165557
Baso	Mitral	ebi-a-GCST	BpI3Ho	424022	rs1247088	0,2472	0,181499	0,173202
Baso	Mitral	ebi-a-GCST	BpI3Ho	424022	rs1249769	0,2514	0,181184	0,165277
Baso	Mitral	ebi-a-GCST	BpI3Ho	424022	rs1253334	0,241943	0,181088	0,181534
Baso	Mitral	ebi-a-GCST	BpI3Ho	424022	rs1292735	0,239881	0,181125	0,18537
Baso	Mitral	ebi-a-GCST	BpI3Ho	424022	rs1294181	0,210565	0,183886	0,252174
Baso	Mitral	ebi-a-GCST	BpI3Ho	424022	rs1295927	0,212757	0,181826	0,241955
Baso	Mitral	ebi-a-GCST	BpI3Ho	424022	rs1306357	0,244676	0,181262	0,177065
Baso	Mitral	ebi-a-GCST	BpI3Ho	424022	rs1341976	0,22422	0,181238	0,216028
Baso	Mitral	ebi-a-GCST	BpI3Ho	424022	rs1343426	0,232412	0,181101	0,199379
Baso	Mitral	ebi-a-GCST	BpI3Ho	424022	rs1424951	0,236762	0,181137	0,191183
Baso	Mitral	ebi-a-GCST	BpI3Ho	424022	rs1427499	0,238986	0,181185	0,187164
Baso	Mitral	ebi-a-GCST	BpI3Ho	424022	rs1469706	0,243121	0,181177	0,179629
Baso	Mitral	ebi-a-GCST	BpI3Ho	424022	rs1490077	0,248752	0,181495	0,170508
Baso	Mitral	ebi-a-GCST	BpI3Ho	424022	rs1493912	0,265248	0,181233	0,143311
Baso	Mitral	ebi-a-GCST	BpI3Ho	424022	rs1504982	0,245436	0,181077	0,175282
Baso	Mitral	ebi-a-GCST	BpI3Ho	424022	rs1669340	0,215286	0,181322	0,235105
Baso	Mitral	ebi-a-GCST	BpI3Ho	424022	rs1692363	0,233123	0,181106	0,198019
Baso	Mitral	ebi-a-GCST	BpI3Ho	424022	rs1761333	0,254424	0,181154	0,160181

Baso	Mitral	ebi-a-GCST	BpI3Ho	424022	rs1775869!	0,230126	0,182522	0,207377
Baso	Mitral	ebi-a-GCST	BpI3Ho	424022	rs1847271	0,230657	0,181208	0,203057
Baso	Mitral	ebi-a-GCST	BpI3Ho	424022	rs1897191	0,22397	0,181312	0,216729
Baso	Mitral	ebi-a-GCST	BpI3Ho	424022	rs1973791	0,240351	0,181267	0,184857
Baso	Mitral	ebi-a-GCST	BpI3Ho	424022	rs2070596	0,249191	0,18121	0,169084
Baso	Mitral	ebi-a-GCST	BpI3Ho	424022	rs2073935	0,221785	0,181087	0,22067
Baso	Mitral	ebi-a-GCST	BpI3Ho	424022	rs2074585	0,244872	0,182623	0,179966
Baso	Mitral	ebi-a-GCST	BpI3Ho	424022	rs2118140	0,231796	0,181169	0,200738
Baso	Mitral	ebi-a-GCST	BpI3Ho	424022	rs2158799	0,249066	0,181196	0,169267
Baso	Mitral	ebi-a-GCST	BpI3Ho	424022	rs2239630	0,212829	0,181909	0,242011
Baso	Mitral	ebi-a-GCST	BpI3Ho	424022	rs2271352	0,267809	0,182778	0,142862
Baso	Mitral	ebi-a-GCST	BpI3Ho	424022	rs2276066	0,246191	0,181107	0,174031
Baso	Mitral	ebi-a-GCST	BpI3Ho	424022	rs2286599	0,246238	0,181131	0,174004
Baso	Mitral	ebi-a-GCST	BpI3Ho	424022	rs2289511	0,245327	0,181272	0,17594
Baso	Mitral	ebi-a-GCST	BpI3Ho	424022	rs2336068	0,236623	0,18112	0,191401
Baso	Mitral	ebi-a-GCST	BpI3Ho	424022	rs247830	0,228513	0,181144	0,207129
Baso	Mitral	ebi-a-GCST	BpI3Ho	424022	rs2524079	0,247838	0,182154	0,173642
Baso	Mitral	ebi-a-GCST	BpI3Ho	424022	rs2594836	0,266155	0,181179	0,141828
Baso	Mitral	ebi-a-GCST	BpI3Ho	424022	rs2738104	0,227771	0,181383	0,209208
Baso	Mitral	ebi-a-GCST	BpI3Ho	424022	rs2811708	0,246065	0,181229	0,174542
Baso	Mitral	ebi-a-GCST	BpI3Ho	424022	rs2836154	0,234728	0,181242	0,195283
Baso	Mitral	ebi-a-GCST	BpI3Ho	424022	rs2967595	0,220703	0,181506	0,224003
Baso	Mitral	ebi-a-GCST	BpI3Ho	424022	rs2998286	0,230398	0,181445	0,204156
Baso	Mitral	ebi-a-GCST	BpI3Ho	424022	rs310631	0,246587	0,181122	0,173375
Baso	Mitral	ebi-a-GCST	BpI3Ho	424022	rs3181077	0,247062	0,182914	0,176792
Baso	Mitral	ebi-a-GCST	BpI3Ho	424022	rs3184504	0,242384	0,181994	0,182919
Baso	Mitral	ebi-a-GCST	BpI3Ho	424022	rs3393198	0,226468	0,181066	0,211026
Baso	Mitral	ebi-a-GCST	BpI3Ho	424022	rs3415872	0,254878	0,182527	0,162598
Baso	Mitral	ebi-a-GCST	BpI3Ho	424022	rs34500	0,232546	0,181205	0,199374
Baso	Mitral	ebi-a-GCST	BpI3Ho	424022	rs3463865	0,249584	0,181111	0,16818
Baso	Mitral	ebi-a-GCST	BpI3Ho	424022	rs3498015	0,240871	0,181289	0,18396
Baso	Mitral	ebi-a-GCST	BpI3Ho	424022	rs377763	0,230475	0,181439	0,203992
Baso	Mitral	ebi-a-GCST	BpI3Ho	424022	rs3781452	0,224737	0,181446	0,215499
Baso	Mitral	ebi-a-GCST	BpI3Ho	424022	rs3806296	0,246137	0,181085	0,174073
Baso	Mitral	ebi-a-GCST	BpI3Ho	424022	rs3848148	0,232553	0,181219	0,199398
Baso	Mitral	ebi-a-GCST	BpI3Ho	424022	rs431329	0,24556	0,181243	0,17546
Baso	Mitral	ebi-a-GCST	BpI3Ho	424022	rs4324460	0,237976	0,181152	0,188954
Baso	Mitral	ebi-a-GCST	BpI3Ho	424022	rs445	0,199981	0,184441	0,278251
Baso	Mitral	ebi-a-GCST	BpI3Ho	424022	rs4475963	0,234912	0,181227	0,194896
Baso	Mitral	ebi-a-GCST	BpI3Ho	424022	rs4541868	0,233827	0,181218	0,196945
Baso	Mitral	ebi-a-GCST	BpI3Ho	424022	rs4557713	0,248733	0,181319	0,170126
Baso	Mitral	ebi-a-GCST	BpI3Ho	424022	rs4602187	0,244177	0,181137	0,177649
Baso	Mitral	ebi-a-GCST	BpI3Ho	424022	rs4753251	0,228186	0,181147	0,207788
Baso	Mitral	ebi-a-GCST	BpI3Ho	424022	rs4782302	0,238674	0,181304	0,188031
Baso	Mitral	ebi-a-GCST	BpI3Ho	424022	rs483640	0,232876	0,181086	0,198444
Baso	Mitral	ebi-a-GCST	BpI3Ho	424022	rs4851592	0,229862	0,181204	0,20461
Baso	Mitral	ebi-a-GCST	BpI3Ho	424022	rs4911102	0,235622	0,181134	0,193323
Baso	Mitral	ebi-a-GCST	BpI3Ho	424022	rs4931	0,243375	0,181467	0,17987
Baso	Mitral	ebi-a-GCST	BpI3Ho	424022	rs4941839	0,251807	0,181099	0,164395
Baso	Mitral	ebi-a-GCST	BpI3Ho	424022	rs4974107	0,241849	0,181105	0,181743

Baso	Mitral	ebi-a-GCST	BpI3Ho	424022	rs524137	0,253966	0,181089	0,160784
Baso	Mitral	ebi-a-GCST	BpI3Ho	424022	rs5591316	0,266752	0,18168	0,142036
Baso	Mitral	ebi-a-GCST	BpI3Ho	424022	rs5599090	0,246322	0,181144	0,17389
Baso	Mitral	ebi-a-GCST	BpI3Ho	424022	rs5601490	0,231777	0,181281	0,201055
Baso	Mitral	ebi-a-GCST	BpI3Ho	424022	rs561102	0,225601	0,181178	0,213063
Baso	Mitral	ebi-a-GCST	BpI3Ho	424022	rs5638817	0,240142	0,182108	0,187278
Baso	Mitral	ebi-a-GCST	BpI3Ho	424022	rs564449	0,242965	0,181093	0,179707
Baso	Mitral	ebi-a-GCST	BpI3Ho	424022	rs5745281	0,220278	0,181089	0,223829
Baso	Mitral	ebi-a-GCST	BpI3Ho	424022	rs5756822	0,239058	0,181133	0,186906
Baso	Mitral	ebi-a-GCST	BpI3Ho	424022	rs6045612	0,241853	0,181152	0,181849
Baso	Mitral	ebi-a-GCST	BpI3Ho	424022	rs6091176	0,247188	0,181113	0,172309
Baso	Mitral	ebi-a-GCST	BpI3Ho	424022	rs6141781	0,235781	0,181287	0,193398
Baso	Mitral	ebi-a-GCST	BpI3Ho	424022	rs6210547	0,252408	0,181124	0,163448
Baso	Mitral	ebi-a-GCST	BpI3Ho	424022	rs6239357	0,234536	0,181102	0,195303
Baso	Mitral	ebi-a-GCST	BpI3Ho	424022	rs6240820	0,2304	0,181078	0,203238
Baso	Mitral	ebi-a-GCST	BpI3Ho	424022	rs6717590	0,255561	0,181319	0,158701
Baso	Mitral	ebi-a-GCST	BpI3Ho	424022	rs6734238	0,236325	0,181076	0,191855
Baso	Mitral	ebi-a-GCST	BpI3Ho	424022	rs6766037	0,271582	0,182378	0,136458
Baso	Mitral	ebi-a-GCST	BpI3Ho	424022	rs6782812	0,242487	0,183229	0,185699
Baso	Mitral	ebi-a-GCST	BpI3Ho	424022	rs6927569	0,239722	0,181382	0,186287
Baso	Mitral	ebi-a-GCST	BpI3Ho	424022	rs695113	0,261674	0,181087	0,148453
Baso	Mitral	ebi-a-GCST	BpI3Ho	424022	rs7044519	0,246401	0,181087	0,173616
Baso	Mitral	ebi-a-GCST	BpI3Ho	424022	rs7166645	0,224189	0,18115	0,215869
Baso	Mitral	ebi-a-GCST	BpI3Ho	424022	rs7196129	0,245785	0,181314	0,175235
Baso	Mitral	ebi-a-GCST	BpI3Ho	424022	rs7250849	0,240554	0,181107	0,184098
Baso	Mitral	ebi-a-GCST	BpI3Ho	424022	rs7261724	0,234372	0,181085	0,195573
Baso	Mitral	ebi-a-GCST	BpI3Ho	424022	rs7272163	0,228319	0,18131	0,207931
Baso	Mitral	ebi-a-GCST	BpI3Ho	424022	rs7289523	0,228944	0,181243	0,206522
Baso	Mitral	ebi-a-GCST	BpI3Ho	424022	rs7304925	0,236657	0,181446	0,192137
Baso	Mitral	ebi-a-GCST	BpI3Ho	424022	rs7398760	0,24161	0,181293	0,182629
Baso	Mitral	ebi-a-GCST	BpI3Ho	424022	rs7418059	0,250414	0,181102	0,16675
Baso	Mitral	ebi-a-GCST	BpI3Ho	424022	rs742631	0,234609	0,181154	0,195292
Baso	Mitral	ebi-a-GCST	BpI3Ho	424022	rs7447289	0,214213	0,184476	0,245561
Baso	Mitral	ebi-a-GCST	BpI3Ho	424022	rs7453541	0,25568	0,181081	0,157961
Baso	Mitral	ebi-a-GCST	BpI3Ho	424022	rs7503461	0,227934	0,181127	0,208238
Baso	Mitral	ebi-a-GCST	BpI3Ho	424022	rs761702	0,244356	0,181339	0,177817
Baso	Mitral	ebi-a-GCST	BpI3Ho	424022	rs7642810	0,214581	0,181249	0,236453
Baso	Mitral	ebi-a-GCST	BpI3Ho	424022	rs7832357	0,249978	0,181097	0,167478
Baso	Mitral	ebi-a-GCST	BpI3Ho	424022	rs7874418	0,243886	0,183988	0,184988
Baso	Mitral	ebi-a-GCST	BpI3Ho	424022	rs7886295	0,244965	0,181168	0,176332
Baso	Mitral	ebi-a-GCST	BpI3Ho	424022	rs7914063	0,242367	0,181085	0,180763
Baso	Mitral	ebi-a-GCST	BpI3Ho	424022	rs8026614	0,247925	0,181123	0,171055
Baso	Mitral	ebi-a-GCST	BpI3Ho	424022	rs8113682	0,231072	0,181406	0,202741
Baso	Mitral	ebi-a-GCST	BpI3Ho	424022	rs8178414	0,248168	0,181396	0,171281
Baso	Mitral	ebi-a-GCST	BpI3Ho	424022	rs8179	0,235784	0,181895	0,194884
Baso	Mitral	ebi-a-GCST	BpI3Ho	424022	rs873636	0,24663	0,181519	0,17424
Baso	Mitral	ebi-a-GCST	BpI3Ho	424022	rs875740	0,237919	0,181303	0,189427
Baso	Mitral	ebi-a-GCST	BpI3Ho	424022	rs877116	0,224364	0,181254	0,215774
Baso	Mitral	ebi-a-GCST	BpI3Ho	424022	rs906149	0,24243	0,181148	0,1808
Baso	Mitral	ebi-a-GCST	BpI3Ho	424022	rs915125	0,233993	0,182112	0,198833

Baso	Mitral	ebi-a-GCST	BpI3Ho	424022	rs9376098	0,241254	0,181669	0,184182
Baso	Mitral	ebi-a-GCST	BpI3Ho	424022	rs9644063	0,259391	0,181439	0,152824
Baso	Mitral	ebi-a-GCST	BpI3Ho	424022	rs9819371	0,234285	0,181224	0,196083
Baso	Mitral	ebi-a-GCST	BpI3Ho	424022	All	0,238813	0,180884	0,186751
Eosino	Mitral	ebi-a-GCST	hDNVJo	451679	rs1004870	-0,03743	0,103366	0,717264
Eosino	Mitral	ebi-a-GCST	hDNVJo	451679	rs1006234	-0,0264	0,103581	0,798822
Eosino	Mitral	ebi-a-GCST	hDNVJo	451679	rs1006268	-0,02608	0,103309	0,80073
Eosino	Mitral	ebi-a-GCST	hDNVJo	451679	rs1013734	-0,02649	0,103244	0,797471
Eosino	Mitral	ebi-a-GCST	hDNVJo	451679	rs1016520	-0,03016	0,103346	0,770417
Eosino	Mitral	ebi-a-GCST	hDNVJo	451679	rs1016567	-0,02811	0,103242	0,785445
Eosino	Mitral	ebi-a-GCST	hDNVJo	451679	rs1017423	-0,028	0,103241	0,78623
Eosino	Mitral	ebi-a-GCST	hDNVJo	451679	rs1019571	-0,02865	0,103286	0,781451
Eosino	Mitral	ebi-a-GCST	hDNVJo	451679	rs1027589	-0,03484	0,10324	0,735742
Eosino	Mitral	ebi-a-GCST	hDNVJo	451679	rs1036332	-0,03301	0,103399	0,749537
Eosino	Mitral	ebi-a-GCST	hDNVJo	451679	rs1045502	-0,03214	0,103573	0,756311
Eosino	Mitral	ebi-a-GCST	hDNVJo	451679	rs1057258	-0,02936	0,103352	0,776359
Eosino	Mitral	ebi-a-GCST	hDNVJo	451679	rs1059091	-0,03158	0,103453	0,760151
Eosino	Mitral	ebi-a-GCST	hDNVJo	451679	rs1079466	-0,02511	0,103278	0,807923
Eosino	Mitral	ebi-a-GCST	hDNVJo	451679	rs1085927	-0,03297	0,10334	0,749715
Eosino	Mitral	ebi-a-GCST	hDNVJo	451679	rs1087655	-0,02502	0,103255	0,808571
Eosino	Mitral	ebi-a-GCST	hDNVJo	451679	rs1093033	-0,02974	0,103243	0,773301
Eosino	Mitral	ebi-a-GCST	hDNVJo	451679	rs1095671	-0,02203	0,103263	0,831076
Eosino	Mitral	ebi-a-GCST	hDNVJo	451679	rs1095797	-0,0266	0,103261	0,796683
Eosino	Mitral	ebi-a-GCST	hDNVJo	451679	rs1096267	-0,03033	0,103248	0,768966
Eosino	Mitral	ebi-a-GCST	hDNVJo	451679	rs1099853	-0,03234	0,103246	0,754075
Eosino	Mitral	ebi-a-GCST	hDNVJo	451679	rs1102464	-0,0229	0,103259	0,82448
Eosino	Mitral	ebi-a-GCST	hDNVJo	451679	rs1112642	-0,02865	0,103253	0,781414
Eosino	Mitral	ebi-a-GCST	hDNVJo	451679	rs1115482	-0,02978	0,103298	0,773086
Eosino	Mitral	ebi-a-GCST	hDNVJo	451679	rs1117061	-0,03285	0,103241	0,750378
Eosino	Mitral	ebi-a-GCST	hDNVJo	451679	rs1120468	-0,03672	0,103495	0,722748
Eosino	Mitral	ebi-a-GCST	hDNVJo	451679	rs1123681	-0,02724	0,103287	0,791995
Eosino	Mitral	ebi-a-GCST	hDNVJo	451679	rs1125351	-0,03326	0,103455	0,747831
Eosino	Mitral	ebi-a-GCST	hDNVJo	451679	rs1125550	-0,0246	0,103268	0,811691
Eosino	Mitral	ebi-a-GCST	hDNVJo	451679	rs1127338	-0,03291	0,103273	0,749975
Eosino	Mitral	ebi-a-GCST	hDNVJo	451679	rs1134736	-0,02882	0,103287	0,78021
Eosino	Mitral	ebi-a-GCST	hDNVJo	451679	rs1134966	-0,03107	0,103256	0,763453
Eosino	Mitral	ebi-a-GCST	hDNVJo	451679	rs1135423	-0,03028	0,103256	0,769305
Eosino	Mitral	ebi-a-GCST	hDNVJo	451679	rs1135777	-0,02658	0,103334	0,79704
Eosino	Mitral	ebi-a-GCST	hDNVJo	451679	rs1138358	-0,01738	0,103381	0,866497
Eosino	Mitral	ebi-a-GCST	hDNVJo	451679	rs1149456	-0,02842	0,103266	0,783155
Eosino	Mitral	ebi-a-GCST	hDNVJo	451679	rs1154787	-0,03459	0,103321	0,737767
Eosino	Mitral	ebi-a-GCST	hDNVJo	451679	rs1155554	-0,03286	0,103455	0,750773
Eosino	Mitral	ebi-a-GCST	hDNVJo	451679	rs1157028	-0,02582	0,10324	0,802519
Eosino	Mitral	ebi-a-GCST	hDNVJo	451679	rs1161853	-0,03374	0,103245	0,743792
Eosino	Mitral	ebi-a-GCST	hDNVJo	451679	rs1162856	-0,03697	0,10327	0,72034
Eosino	Mitral	ebi-a-GCST	hDNVJo	451679	rs1163788	-0,02884	0,103243	0,780019
Eosino	Mitral	ebi-a-GCST	hDNVJo	451679	rs1163825	-0,02935	0,103244	0,7762
Eosino	Mitral	ebi-a-GCST	hDNVJo	451679	rs1167802	-0,03078	0,103271	0,765699
Eosino	Mitral	ebi-a-GCST	hDNVJo	451679	rs1170144	-0,0285	0,103248	0,782554
Eosino	Mitral	ebi-a-GCST	hDNVJo	451679	rs1170439	-0,02663	0,103312	0,796603

Eosino	Mitral	ebi-a-GCST	hDNVJo	451679	rs1170685!	-0,03194	0,103298	0,757199
Eosino	Mitral	ebi-a-GCST	hDNVJo	451679	rs1177103:	-0,03512	0,103278	0,733844
Eosino	Mitral	ebi-a-GCST	hDNVJo	451679	rs117865З(	-0,03148	0,103308	0,760598
Eosino	Mitral	ebi-a-GCST	hDNVJo	451679	rs1179615:	-0,03326	0,103243	0,747339
Eosino	Mitral	ebi-a-GCST	hDNVJo	451679	rs1181010!	-0,02939	0,103254	0,775958
Eosino	Mitral	ebi-a-GCST	hDNVJo	451679	rs1181492	-0,02935	0,103234	0,776179
Eosino	Mitral	ebi-a-GCST	hDNVJo	451679	rs1182215	-0,02455	0,103242	0,812035
Eosino	Mitral	ebi-a-GCST	hDNVJo	451679	rs1193171:	-0,03633	0,10329	0,725065
Eosino	Mitral	ebi-a-GCST	hDNVJo	451679	rs1210003	-0,02855	0,103262	0,782191
Eosino	Mitral	ebi-a-GCST	hDNVJo	451679	rs1213774	-0,02881	0,103279	0,780289
Eosino	Mitral	ebi-a-GCST	hDNVJo	451679	rs1214263!	-0,0297	0,103248	0,773619
Eosino	Mitral	ebi-a-GCST	hDNVJo	451679	rs1215227(	-0,03051	0,103297	0,767731
Eosino	Mitral	ebi-a-GCST	hDNVJo	451679	rs1220810:	-0,03481	0,103418	0,736397
Eosino	Mitral	ebi-a-GCST	hDNVJo	451679	rs1231379(	-0,02832	0,103251	0,783891
Eosino	Mitral	ebi-a-GCST	hDNVJo	451679	rs1240893	-0,03091	0,103362	0,764929
Eosino	Mitral	ebi-a-GCST	hDNVJo	451679	rs1243269	-0,02992	0,103249	0,772014
Eosino	Mitral	ebi-a-GCST	hDNVJo	451679	rs1251518(	-0,00907	0,104498	0,930835
Eosino	Mitral	ebi-a-GCST	hDNVJo	451679	rs1253094(	-0,00197	0,103621	0,984842
Eosino	Mitral	ebi-a-GCST	hDNVJo	451679	rs1253761	-0,03167	0,103386	0,759324
Eosino	Mitral	ebi-a-GCST	hDNVJo	451679	rs1253898!	-0,03446	0,103267	0,738589
Eosino	Mitral	ebi-a-GCST	hDNVJo	451679	rs1257192	-0,03199	0,103238	0,756654
Eosino	Mitral	ebi-a-GCST	hDNVJo	451679	rs1258151:	-0,02781	0,10325	0,787664
Eosino	Mitral	ebi-a-GCST	hDNVJo	451679	rs1261928!	-0,01689	0,103985	0,870989
Eosino	Mitral	ebi-a-GCST	hDNVJo	451679	rs1262260(	-0,02978	0,103252	0,772994
Eosino	Mitral	ebi-a-GCST	hDNVJo	451679	rs1268164	-0,03145	0,103273	0,760755
Eosino	Mitral	ebi-a-GCST	hDNVJo	451679	rs1270584!	-0,02565	0,103313	0,803925
Eosino	Mitral	ebi-a-GCST	hDNVJo	451679	rs1271423	-0,02781	0,103244	0,787685
Eosino	Mitral	ebi-a-GCST	hDNVJo	451679	rs1282086:	-0,03009	0,103305	0,770825
Eosino	Mitral	ebi-a-GCST	hDNVJo	451679	rs1287565!	-0,03305	0,103258	0,748915
Eosino	Mitral	ebi-a-GCST	hDNVJo	451679	rs1293297(	-0,03521	0,103263	0,733107
Eosino	Mitral	ebi-a-GCST	hDNVJo	451679	rs1294106!	-0,02978	0,103248	0,772996
Eosino	Mitral	ebi-a-GCST	hDNVJo	451679	rs1295258:	-0,04019	0,103393	0,69747
Eosino	Mitral	ebi-a-GCST	hDNVJo	451679	rs1295370!	-0,03268	0,103276	0,75167
Eosino	Mitral	ebi-a-GCST	hDNVJo	451679	rs1296535	-0,03267	0,103259	0,751686
Eosino	Mitral	ebi-a-GCST	hDNVJo	451679	rs1296869	-0,03	0,103274	0,77147
Eosino	Mitral	ebi-a-GCST	hDNVJo	451679	rs1300474:	-0,02865	0,103241	0,78137
Eosino	Mitral	ebi-a-GCST	hDNVJo	451679	rs1302124	-0,03137	0,103245	0,76122
Eosino	Mitral	ebi-a-GCST	hDNVJo	451679	rs1306152:	-0,0283	0,103279	0,784061
Eosino	Mitral	ebi-a-GCST	hDNVJo	451679	rs1307368:	-0,02926	0,103263	0,776918
Eosino	Mitral	ebi-a-GCST	hDNVJo	451679	rs1307833(	-0,03022	0,103266	0,769828
Eosino	Mitral	ebi-a-GCST	hDNVJo	451679	rs1310568:	-0,02541	0,103245	0,805578
Eosino	Mitral	ebi-a-GCST	hDNVJo	451679	rs1312037:	-0,03069	0,103264	0,766284
Eosino	Mitral	ebi-a-GCST	hDNVJo	451679	rs1320779:	-0,02779	0,103248	0,787777
Eosino	Mitral	ebi-a-GCST	hDNVJo	451679	rs1322658:	-0,02202	0,103586	0,831662
Eosino	Mitral	ebi-a-GCST	hDNVJo	451679	rs1323650	-0,03114	0,103245	0,762953
Eosino	Mitral	ebi-a-GCST	hDNVJo	451679	rs1325593!	-0,02665	0,103246	0,796343
Eosino	Mitral	ebi-a-GCST	hDNVJo	451679	rs1331691	-0,0257	0,103333	0,803592
Eosino	Mitral	ebi-a-GCST	hDNVJo	451679	rs1333940	-0,02748	0,103244	0,790117
Eosino	Mitral	ebi-a-GCST	hDNVJo	451679	rs1334894	-0,03203	0,103299	0,756494
Eosino	Mitral	ebi-a-GCST	hDNVJo	451679	rs13511	-0,02757	0,10327	0,789511

Eosino	Mitral	ebi-a-GCST	hDNVJo	451679	rs1351442	-0,02951	0,10324	0,775004
Eosino	Mitral	ebi-a-GCST	hDNVJo	451679	rs1353286	-0,02633	0,103303	0,798801
Eosino	Mitral	ebi-a-GCST	hDNVJo	451679	rs1403602:	-0,02892	0,103238	0,77938
Eosino	Mitral	ebi-a-GCST	hDNVJo	451679	rs1404112	-0,03092	0,103249	0,764544
Eosino	Mitral	ebi-a-GCST	hDNVJo	451679	rs1427499	-0,03001	0,103275	0,771361
Eosino	Mitral	ebi-a-GCST	hDNVJo	451679	rs1444782	-0,03525	0,103599	0,733652
Eosino	Mitral	ebi-a-GCST	hDNVJo	451679	rs1458908	-0,02841	0,103241	0,783156
Eosino	Mitral	ebi-a-GCST	hDNVJo	451679	rs1491105	-0,03033	0,103261	0,768976
Eosino	Mitral	ebi-a-GCST	hDNVJo	451679	rs1506400	-0,03189	0,103348	0,757629
Eosino	Mitral	ebi-a-GCST	hDNVJo	451679	rs1516527	-0,03374	0,103254	0,743811
Eosino	Mitral	ebi-a-GCST	hDNVJo	451679	rs1519602	-0,03185	0,103252	0,757695
Eosino	Mitral	ebi-a-GCST	hDNVJo	451679	rs152197	-0,0423	0,103584	0,683015
Eosino	Mitral	ebi-a-GCST	hDNVJo	451679	rs1530161	-0,02826	0,103245	0,784296
Eosino	Mitral	ebi-a-GCST	hDNVJo	451679	rs159963	-0,02756	0,10329	0,789586
Eosino	Mitral	ebi-a-GCST	hDNVJo	451679	rs1611236	-0,02092	0,103573	0,839939
Eosino	Mitral	ebi-a-GCST	hDNVJo	451679	rs1684578	-0,02879	0,10327	0,780405
Eosino	Mitral	ebi-a-GCST	hDNVJo	451679	rs1689510	-0,03381	0,103366	0,743602
Eosino	Mitral	ebi-a-GCST	hDNVJo	451679	rs1690357	-0,03629	0,103262	0,725259
Eosino	Mitral	ebi-a-GCST	hDNVJo	451679	rs1691754	-0,01641	0,103665	0,874211
Eosino	Mitral	ebi-a-GCST	hDNVJo	451679	rs1694311	-0,03532	0,103251	0,732326
Eosino	Mitral	ebi-a-GCST	hDNVJo	451679	rs1695315	-0,02767	0,103492	0,789195
Eosino	Mitral	ebi-a-GCST	hDNVJo	451679	rs1700589	-0,02927	0,103653	0,777636
Eosino	Mitral	ebi-a-GCST	hDNVJo	451679	rs1712693	-0,02484	0,103255	0,809893
Eosino	Mitral	ebi-a-GCST	hDNVJo	451679	rs1717583	-0,0255	0,10339	0,805194
Eosino	Mitral	ebi-a-GCST	hDNVJo	451679	rs1724438	-0,03428	0,103381	0,740209
Eosino	Mitral	ebi-a-GCST	hDNVJo	451679	rs1729363	-0,03028	0,103365	0,769601
Eosino	Mitral	ebi-a-GCST	hDNVJo	451679	rs1737746	-0,03257	0,103255	0,752462
Eosino	Mitral	ebi-a-GCST	hDNVJo	451679	rs1745029	-0,03158	0,103277	0,759802
Eosino	Mitral	ebi-a-GCST	hDNVJo	451679	rs174548	-0,03051	0,103317	0,767745
Eosino	Mitral	ebi-a-GCST	hDNVJo	451679	rs1748247	-0,03165	0,103302	0,759329
Eosino	Mitral	ebi-a-GCST	hDNVJo	451679	rs175705	-0,03959	0,103504	0,702083
Eosino	Mitral	ebi-a-GCST	hDNVJo	451679	rs1768257	-0,03206	0,103258	0,756193
Eosino	Mitral	ebi-a-GCST	hDNVJo	451679	rs1771534	-0,02978	0,103238	0,773018
Eosino	Mitral	ebi-a-GCST	hDNVJo	451679	rs1775869	-0,03485	0,103611	0,736622
Eosino	Mitral	ebi-a-GCST	hDNVJo	451679	rs1776738	-0,0279	0,103242	0,786989
Eosino	Mitral	ebi-a-GCST	hDNVJo	451679	rs1784950	-0,02589	0,103246	0,801971
Eosino	Mitral	ebi-a-GCST	hDNVJo	451679	rs1788153	-0,03415	0,103259	0,740838
Eosino	Mitral	ebi-a-GCST	hDNVJo	451679	rs1800692	-0,03766	0,10329	0,715415
Eosino	Mitral	ebi-a-GCST	hDNVJo	451679	rs180506	-0,02931	0,103297	0,776574
Eosino	Mitral	ebi-a-GCST	hDNVJo	451679	rs1828803	-0,03018	0,103246	0,770079
Eosino	Mitral	ebi-a-GCST	hDNVJo	451679	rs1861160	-0,02946	0,103255	0,775418
Eosino	Mitral	ebi-a-GCST	hDNVJo	451679	rs1869843	-0,02621	0,103242	0,799561
Eosino	Mitral	ebi-a-GCST	hDNVJo	451679	rs1888909	0,008733	0,104979	0,933706
Eosino	Mitral	ebi-a-GCST	hDNVJo	451679	rs1915834	-0,02996	0,103253	0,771683
Eosino	Mitral	ebi-a-GCST	hDNVJo	451679	rs1916749	-0,02726	0,103313	0,791854
Eosino	Mitral	ebi-a-GCST	hDNVJo	451679	rs1969026	-0,03176	0,103236	0,758322
Eosino	Mitral	ebi-a-GCST	hDNVJo	451679	rs2004925	-0,03076	0,103264	0,765824
Eosino	Mitral	ebi-a-GCST	hDNVJo	451679	rs201798	-0,03017	0,103307	0,77023
Eosino	Mitral	ebi-a-GCST	hDNVJo	451679	rs2043293	-0,02853	0,103244	0,782327
Eosino	Mitral	ebi-a-GCST	hDNVJo	451679	rs2072130	-0,0184	0,103403	0,858745

Eosino	Mitral	ebi-a-GCST	hDNVJo	451679	rs2133480	-0,02943	0,103273	0,775668
Eosino	Mitral	ebi-a-GCST	hDNVJo	451679	rs214097	-0,03273	0,103242	0,751226
Eosino	Mitral	ebi-a-GCST	hDNVJo	451679	rs2161647	-0,02947	0,103264	0,775331
Eosino	Mitral	ebi-a-GCST	hDNVJo	451679	rs2228467	-0,02426	0,103425	0,814541
Eosino	Mitral	ebi-a-GCST	hDNVJo	451679	rs2239633	-0,03434	0,103504	0,740045
Eosino	Mitral	ebi-a-GCST	hDNVJo	451679	rs2242193	-0,02892	0,103247	0,779412
Eosino	Mitral	ebi-a-GCST	hDNVJo	451679	rs2242886	-0,0274	0,103873	0,791934
Eosino	Mitral	ebi-a-GCST	hDNVJo	451679	rs228951	-0,03021	0,103306	0,769987
Eosino	Mitral	ebi-a-GCST	hDNVJo	451679	rs2296618	-0,02645	0,103376	0,798042
Eosino	Mitral	ebi-a-GCST	hDNVJo	451679	rs2366640	-0,03049	0,103256	0,76774
Eosino	Mitral	ebi-a-GCST	hDNVJo	451679	rs2399441	-0,02853	0,103288	0,782415
Eosino	Mitral	ebi-a-GCST	hDNVJo	451679	rs2412771	-0,0318	0,103245	0,758068
Eosino	Mitral	ebi-a-GCST	hDNVJo	451679	rs2413631	-0,03237	0,103245	0,753856
Eosino	Mitral	ebi-a-GCST	hDNVJo	451679	rs2431097	-0,02631	0,103252	0,798902
Eosino	Mitral	ebi-a-GCST	hDNVJo	451679	rs244689	-0,02557	0,103255	0,804397
Eosino	Mitral	ebi-a-GCST	hDNVJo	451679	rs2502994	-0,02932	0,103309	0,776591
Eosino	Mitral	ebi-a-GCST	hDNVJo	451679	rs2544677	-0,02993	0,10324	0,771919
Eosino	Mitral	ebi-a-GCST	hDNVJo	451679	rs2568928	-0,02889	0,103251	0,779594
Eosino	Mitral	ebi-a-GCST	hDNVJo	451679	rs2579500	-0,04813	0,103776	0,642778
Eosino	Mitral	ebi-a-GCST	hDNVJo	451679	rs2646438	-0,03291	0,103308	0,750066
Eosino	Mitral	ebi-a-GCST	hDNVJo	451679	rs277591	-0,03354	0,103242	0,745282
Eosino	Mitral	ebi-a-GCST	hDNVJo	451679	rs2793925	-0,02753	0,103261	0,789763
Eosino	Mitral	ebi-a-GCST	hDNVJo	451679	rs2807742	-0,03489	0,103449	0,735921
Eosino	Mitral	ebi-a-GCST	hDNVJo	451679	rs2817399	-0,02999	0,103244	0,771487
Eosino	Mitral	ebi-a-GCST	hDNVJo	451679	rs2838331[illegible]	-0,0271	0,104137	0,794699
Eosino	Mitral	ebi-a-GCST	hDNVJo	451679	rs2840862[illegible]	-0,03358	0,103288	0,74508
Eosino	Mitral	ebi-a-GCST	hDNVJo	451679	rs2847266	-0,03316	0,103247	0,74805
Eosino	Mitral	ebi-a-GCST	hDNVJo	451679	rs2886634[illegible]	-0,03051	0,10327	0,767628
Eosino	Mitral	ebi-a-GCST	hDNVJo	451679	rs2943660	-0,0316	0,103241	0,759513
Eosino	Mitral	ebi-a-GCST	hDNVJo	451679	rs2992335	-0,02377	0,103487	0,818299
Eosino	Mitral	ebi-a-GCST	hDNVJo	451679	rs301161	-0,03139	0,103297	0,761228
Eosino	Mitral	ebi-a-GCST	hDNVJo	451679	rs3016175	-0,03292	0,103247	0,749815
Eosino	Mitral	ebi-a-GCST	hDNVJo	451679	rs3024971	-0,02049	0,103347	0,842849
Eosino	Mitral	ebi-a-GCST	hDNVJo	451679	rs3093023	-0,02943	0,103242	0,775563
Eosino	Mitral	ebi-a-GCST	hDNVJo	451679	rs3101486	-0,02797	0,103264	0,786463
Eosino	Mitral	ebi-a-GCST	hDNVJo	451679	rs310747	-0,03103	0,103403	0,764091
Eosino	Mitral	ebi-a-GCST	hDNVJo	451679	rs3110791	-0,03079	0,103256	0,765528
Eosino	Mitral	ebi-a-GCST	hDNVJo	451679	rs3132682	-0,03738	0,103927	0,719061
Eosino	Mitral	ebi-a-GCST	hDNVJo	451679	rs3184504	-0,03059	0,105983	0,772831
Eosino	Mitral	ebi-a-GCST	hDNVJo	451679	rs3208305	-0,02998	0,103239	0,771504
Eosino	Mitral	ebi-a-GCST	hDNVJo	451679	rs3398266[illegible]	-0,03147	0,103298	0,76065
Eosino	Mitral	ebi-a-GCST	hDNVJo	451679	rs3417306[illegible]	-0,02538	0,103344	0,806026
Eosino	Mitral	ebi-a-GCST	hDNVJo	451679	rs3421065[illegible]	-0,03475	0,103831	0,737869
Eosino	Mitral	ebi-a-GCST	hDNVJo	451679	rs3421286[illegible]	-0,03372	0,103286	0,744047
Eosino	Mitral	ebi-a-GCST	hDNVJo	451679	rs3429028[illegible]	-0,03918	0,103595	0,705248
Eosino	Mitral	ebi-a-GCST	hDNVJo	451679	rs3443969[illegible]	-0,02879	0,103257	0,780387
Eosino	Mitral	ebi-a-GCST	hDNVJo	451679	rs3444895[illegible]	-0,02934	0,103277	0,776336
Eosino	Mitral	ebi-a-GCST	hDNVJo	451679	rs3446695[illegible]	-0,03024	0,103244	0,769587
Eosino	Mitral	ebi-a-GCST	hDNVJo	451679	rs34495	-0,02884	0,103282	0,78009
Eosino	Mitral	ebi-a-GCST	hDNVJo	451679	rs3478050[illegible]	-0,03228	0,103416	0,75497

Eosino	Mitral	ebi-a-GCST	hDNVJo	451679	rs3504888	-0,02667	0,103334	0,796309
Eosino	Mitral	ebi-a-GCST	hDNVJo	451679	rs3524918	-0,03584	0,103338	0,728758
Eosino	Mitral	ebi-a-GCST	hDNVJo	451679	rs3608435	-0,02974	0,103366	0,773548
Eosino	Mitral	ebi-a-GCST	hDNVJo	451679	rs3746165	-0,0322	0,103252	0,755173
Eosino	Mitral	ebi-a-GCST	hDNVJo	451679	rs3759332	-0,02943	0,103242	0,775564
Eosino	Mitral	ebi-a-GCST	hDNVJo	451679	rs3768790	-0,03741	0,103381	0,717428
Eosino	Mitral	ebi-a-GCST	hDNVJo	451679	rs3786586	-0,03382	0,103331	0,743463
Eosino	Mitral	ebi-a-GCST	hDNVJo	451679	rs3790163	-0,03006	0,103245	0,770948
Eosino	Mitral	ebi-a-GCST	hDNVJo	451679	rs3824867	-0,02645	0,103271	0,797876
Eosino	Mitral	ebi-a-GCST	hDNVJo	451679	rs3848640	-0,02774	0,103254	0,788225
Eosino	Mitral	ebi-a-GCST	hDNVJo	451679	rs4072859	-0,01718	0,103362	0,868015
Eosino	Mitral	ebi-a-GCST	hDNVJo	451679	rs4074672	-0,03138	0,103258	0,761237
Eosino	Mitral	ebi-a-GCST	hDNVJo	451679	rs410867	-0,03922	0,103818	0,705599
Eosino	Mitral	ebi-a-GCST	hDNVJo	451679	rs4128000	-0,02271	0,103315	0,825994
Eosino	Mitral	ebi-a-GCST	hDNVJo	451679	rs412884	-0,03638	0,103911	0,726235
Eosino	Mitral	ebi-a-GCST	hDNVJo	451679	rs4129899	-0,02865	0,103252	0,781396
Eosino	Mitral	ebi-a-GCST	hDNVJo	451679	rs4131338	-0,04381	0,103276	0,671382
Eosino	Mitral	ebi-a-GCST	hDNVJo	451679	rs4236746	-0,03647	0,10328	0,724036
Eosino	Mitral	ebi-a-GCST	hDNVJo	451679	rs4243951	-0,03298	0,103246	0,749413
Eosino	Mitral	ebi-a-GCST	hDNVJo	451679	rs4272	-0,03152	0,103335	0,760332
Eosino	Mitral	ebi-a-GCST	hDNVJo	451679	rs4310436	-0,02626	0,103239	0,79924
Eosino	Mitral	ebi-a-GCST	hDNVJo	451679	rs4409785	-0,03007	0,103252	0,7709
Eosino	Mitral	ebi-a-GCST	hDNVJo	451679	rs445	-0,03912	0,103443	0,705305
Eosino	Mitral	ebi-a-GCST	hDNVJo	451679	rs4464488	-0,02552	0,103251	0,804809
Eosino	Mitral	ebi-a-GCST	hDNVJo	451679	rs4557713	-0,02643	0,103319	0,798093
Eosino	Mitral	ebi-a-GCST	hDNVJo	451679	rs4594881	-0,02919	0,103487	0,777916
Eosino	Mitral	ebi-a-GCST	hDNVJo	451679	rs4618204	-0,03716	0,103282	0,718967
Eosino	Mitral	ebi-a-GCST	hDNVJo	451679	rs4677079	-0,03529	0,103254	0,732513
Eosino	Mitral	ebi-a-GCST	hDNVJo	451679	rs4680250	-0,02819	0,103253	0,784812
Eosino	Mitral	ebi-a-GCST	hDNVJo	451679	rs4684037	-0,02674	0,103251	0,795653
Eosino	Mitral	ebi-a-GCST	hDNVJo	451679	rs4703589	-0,02722	0,103249	0,792047
Eosino	Mitral	ebi-a-GCST	hDNVJo	451679	rs4703730	-0,033	0,103277	0,749318
Eosino	Mitral	ebi-a-GCST	hDNVJo	451679	rs4721559	-0,02766	0,10326	0,788785
Eosino	Mitral	ebi-a-GCST	hDNVJo	451679	rs4739140	-0,0299	0,103263	0,772191
Eosino	Mitral	ebi-a-GCST	hDNVJo	451679	rs4778734	-0,02717	0,103481	0,792868
Eosino	Mitral	ebi-a-GCST	hDNVJo	451679	rs4785903	-0,02293	0,103412	0,824513
Eosino	Mitral	ebi-a-GCST	hDNVJo	451679	rs4789797	-0,02747	0,103238	0,790184
Eosino	Mitral	ebi-a-GCST	hDNVJo	451679	rs4841133	-0,02623	0,103239	0,799405
Eosino	Mitral	ebi-a-GCST	hDNVJo	451679	rs4848100	-0,03249	0,103355	0,753246
Eosino	Mitral	ebi-a-GCST	hDNVJo	451679	rs4870977	-0,02637	0,103247	0,798398
Eosino	Mitral	ebi-a-GCST	hDNVJo	451679	rs4871849	-0,03005	0,103423	0,771359
Eosino	Mitral	ebi-a-GCST	hDNVJo	451679	rs4916555	-0,03718	0,103284	0,718892
Eosino	Mitral	ebi-a-GCST	hDNVJo	451679	rs496475	-0,02595	0,103492	0,801981
Eosino	Mitral	ebi-a-GCST	hDNVJo	451679	rs4968392	-0,02893	0,103256	0,779376
Eosino	Mitral	ebi-a-GCST	hDNVJo	451679	rs5010868	-0,03014	0,103248	0,770322
Eosino	Mitral	ebi-a-GCST	hDNVJo	451679	rs5011652	-0,03249	0,103274	0,753074
Eosino	Mitral	ebi-a-GCST	hDNVJo	451679	rs5587974	-0,03006	0,103514	0,771496
Eosino	Mitral	ebi-a-GCST	hDNVJo	451679	rs5600370	-0,03382	0,103419	0,743652
Eosino	Mitral	ebi-a-GCST	hDNVJo	451679	rs5607227	-0,02798	0,103256	0,786412
Eosino	Mitral	ebi-a-GCST	hDNVJo	451679	rs561059	-0,02786	0,103239	0,787237

Eosino	Mitral	ebi-a-GCST	hDNVJo	451679	rs5617956[illegible]	-0,02936	0,103275	0,776213
Eosino	Mitral	ebi-a-GCST	hDNVJo	451679	rs5633046[illegible]	-0,0196	0,103602	0,849932
Eosino	Mitral	ebi-a-GCST	hDNVJo	451679	rs564443	-0,02515	0,10328	0,807645
Eosino	Mitral	ebi-a-GCST	hDNVJo	451679	rs5747308	-0,02732	0,103261	0,791375
Eosino	Mitral	ebi-a-GCST	hDNVJo	451679	rs5763347[illegible]	-0,02566	0,103275	0,803757
Eosino	Mitral	ebi-a-GCST	hDNVJo	451679	rs5783478[illegible]	-0,01186	0,103832	0,909045
Eosino	Mitral	ebi-a-GCST	hDNVJo	451679	rs5795975[illegible]	-0,03062	0,103248	0,766773
Eosino	Mitral	ebi-a-GCST	hDNVJo	451679	rs584001	-0,02926	0,103288	0,776924
Eosino	Mitral	ebi-a-GCST	hDNVJo	451679	rs5869386[illegible]	-0,0337	0,10324	0,744126
Eosino	Mitral	ebi-a-GCST	hDNVJo	451679	rs5874511[illegible]	-0,0286	0,103255	0,78176
Eosino	Mitral	ebi-a-GCST	hDNVJo	451679	rs5883393[illegible]	-0,0267	0,103259	0,795988
Eosino	Mitral	ebi-a-GCST	hDNVJo	451679	rs5889583[illegible]	-0,03412	0,103243	0,741027
Eosino	Mitral	ebi-a-GCST	hDNVJo	451679	rs5915095[illegible]	-0,02803	0,103294	0,786147
Eosino	Mitral	ebi-a-GCST	hDNVJo	451679	rs6017247	-0,03223	0,10332	0,755069
Eosino	Mitral	ebi-a-GCST	hDNVJo	451679	rs605093	-0,03368	0,103277	0,744339
Eosino	Mitral	ebi-a-GCST	hDNVJo	451679	rs6060000[illegible]	-0,03116	0,103354	0,76307
Eosino	Mitral	ebi-a-GCST	hDNVJo	451679	rs6119332[illegible]	-0,02899	0,103237	0,778837
Eosino	Mitral	ebi-a-GCST	hDNVJo	451679	rs6141755	-0,0313	0,103251	0,761773
Eosino	Mitral	ebi-a-GCST	hDNVJo	451679	rs6173111[illegible]	-0,0348	0,103387	0,73644
Eosino	Mitral	ebi-a-GCST	hDNVJo	451679	rs6196331[illegible]	-0,02865	0,103489	0,781873
Eosino	Mitral	ebi-a-GCST	hDNVJo	451679	rs6200617[illegible]	-0,03052	0,103263	0,767604
Eosino	Mitral	ebi-a-GCST	hDNVJo	451679	rs6208690[illegible]	-0,02899	0,103358	0,779088
Eosino	Mitral	ebi-a-GCST	hDNVJo	451679	rs6210548[illegible]	-0,02586	0,103255	0,80223
Eosino	Mitral	ebi-a-GCST	hDNVJo	451679	rs6211716[illegible]	-0,03233	0,103301	0,754332
Eosino	Mitral	ebi-a-GCST	hDNVJo	451679	rs6227094[illegible]	-0,03313	0,103256	0,748312
Eosino	Mitral	ebi-a-GCST	hDNVJo	451679	rs6232421[illegible]	-0,0237	0,103307	0,818551
Eosino	Mitral	ebi-a-GCST	hDNVJo	451679	rs6239578[illegible]	-0,03313	0,103277	0,748402
Eosino	Mitral	ebi-a-GCST	hDNVJo	451679	rs6240822[illegible]	-0,04358	0,103588	0,67399
Eosino	Mitral	ebi-a-GCST	hDNVJo	451679	rs6242076[illegible]	-0,02703	0,103246	0,793489
Eosino	Mitral	ebi-a-GCST	hDNVJo	451679	rs6247372[illegible]	-0,02796	0,103247	0,786506
Eosino	Mitral	ebi-a-GCST	hDNVJo	451679	rs6254153[illegible]	-0,02727	0,1034	0,79196
Eosino	Mitral	ebi-a-GCST	hDNVJo	451679	rs637064	-0,03013	0,103359	0,770649
Eosino	Mitral	ebi-a-GCST	hDNVJo	451679	rs6448432	-0,02746	0,103245	0,790234
Eosino	Mitral	ebi-a-GCST	hDNVJo	451679	rs6494871	-0,03123	0,103259	0,762279
Eosino	Mitral	ebi-a-GCST	hDNVJo	451679	rs6542368	-0,02915	0,10327	0,777725
Eosino	Mitral	ebi-a-GCST	hDNVJo	451679	rs6556313	-0,0313	0,103288	0,761828
Eosino	Mitral	ebi-a-GCST	hDNVJo	451679	rs6573020	-0,0231	0,10331	0,82304
Eosino	Mitral	ebi-a-GCST	hDNVJo	451679	rs6658698	-0,03027	0,103254	0,769416
Eosino	Mitral	ebi-a-GCST	hDNVJo	451679	rs6672038	-0,02592	0,103342	0,801987
Eosino	Mitral	ebi-a-GCST	hDNVJo	451679	rs6699190	-0,03095	0,103248	0,764375
Eosino	Mitral	ebi-a-GCST	hDNVJo	451679	rs6731125	-0,02703	0,103279	0,793544
Eosino	Mitral	ebi-a-GCST	hDNVJo	451679	rs6782812	-0,03238	0,104901	0,757572
Eosino	Mitral	ebi-a-GCST	hDNVJo	451679	rs6785619[illegible]	-0,0299	0,103324	0,772304
Eosino	Mitral	ebi-a-GCST	hDNVJo	451679	rs6836610	-0,03268	0,103282	0,751688
Eosino	Mitral	ebi-a-GCST	hDNVJo	451679	rs6869605	-0,03163	0,103268	0,759402
Eosino	Mitral	ebi-a-GCST	hDNVJo	451679	rs689274	-0,01915	0,103466	0,853195
Eosino	Mitral	ebi-a-GCST	hDNVJo	451679	rs6924387	-0,03612	0,103274	0,726556
Eosino	Mitral	ebi-a-GCST	hDNVJo	451679	rs6971710	-0,03121	0,103343	0,762643
Eosino	Mitral	ebi-a-GCST	hDNVJo	451679	rs6984825	-0,02814	0,103239	0,785173
Eosino	Mitral	ebi-a-GCST	hDNVJo	451679	rs6989099	-0,02946	0,103272	0,775411

Eosino	Mitral	ebi-a-GCST	hDNVJo	451679	rs7036656	-0,02631	0,103271	0,798891
Eosino	Mitral	ebi-a-GCST	hDNVJo	451679	rs7040707	-0,02545	0,103243	0,805275
Eosino	Mitral	ebi-a-GCST	hDNVJo	451679	rs7080536	-0,02749	0,103288	0,790149
Eosino	Mitral	ebi-a-GCST	hDNVJo	451679	rs7123726	-0,02811	0,103264	0,785451
Eosino	Mitral	ebi-a-GCST	hDNVJo	451679	rs715	-0,03148	0,103271	0,760468
Eosino	Mitral	ebi-a-GCST	hDNVJo	451679	rs7152277	-0,03025	0,103263	0,769597
Eosino	Mitral	ebi-a-GCST	hDNVJo	451679	rs7162818	-0,03018	0,10324	0,770026
Eosino	Mitral	ebi-a-GCST	hDNVJo	451679	rs7173571	-0,02779	0,10325	0,787783
Eosino	Mitral	ebi-a-GCST	hDNVJo	451679	rs7186106	-0,03629	0,103684	0,726304
Eosino	Mitral	ebi-a-GCST	hDNVJo	451679	rs7201518	-0,03301	0,103423	0,749621
Eosino	Mitral	ebi-a-GCST	hDNVJo	451679	rs7215391	-0,03573	0,103243	0,729319
Eosino	Mitral	ebi-a-GCST	hDNVJo	451679	rs7251811	-0,02859	0,103237	0,781796
Eosino	Mitral	ebi-a-GCST	hDNVJo	451679	rs7264802	-0,02989	0,103249	0,772173
Eosino	Mitral	ebi-a-GCST	hDNVJo	451679	rs7270419	-0,03279	0,103237	0,750772
Eosino	Mitral	ebi-a-GCST	hDNVJo	451679	rs7275529	-0,0273	0,103248	0,791464
Eosino	Mitral	ebi-a-GCST	hDNVJo	451679	rs7276663	-0,02987	0,103289	0,772453
Eosino	Mitral	ebi-a-GCST	hDNVJo	451679	rs7283475	-0,0289	0,103244	0,779519
Eosino	Mitral	ebi-a-GCST	hDNVJo	451679	rs7283656	-0,02972	0,103293	0,773536
Eosino	Mitral	ebi-a-GCST	hDNVJo	451679	rs7288670	-0,03642	0,103339	0,72449
Eosino	Mitral	ebi-a-GCST	hDNVJo	451679	rs7302975	-0,02622	0,103292	0,799598
Eosino	Mitral	ebi-a-GCST	hDNVJo	451679	rs7304923	-0,03047	0,103243	0,767929
Eosino	Mitral	ebi-a-GCST	hDNVJo	451679	rs7308654	-0,02678	0,103239	0,795296
Eosino	Mitral	ebi-a-GCST	hDNVJo	451679	rs7323120	-0,02756	0,103273	0,789593
Eosino	Mitral	ebi-a-GCST	hDNVJo	451679	rs7323288	-0,04622	0,103996	0,656754
Eosino	Mitral	ebi-a-GCST	hDNVJo	451679	rs7323820	-0,02884	0,103248	0,779988
Eosino	Mitral	ebi-a-GCST	hDNVJo	451679	rs7327960	-0,03027	0,103247	0,769348
Eosino	Mitral	ebi-a-GCST	hDNVJo	451679	rs7330209	-0,02982	0,103248	0,77269
Eosino	Mitral	ebi-a-GCST	hDNVJo	451679	rs7396371	-0,02439	0,103285	0,813292
Eosino	Mitral	ebi-a-GCST	hDNVJo	451679	rs741344	-0,03024	0,103239	0,769555
Eosino	Mitral	ebi-a-GCST	hDNVJo	451679	rs7423615	-0,02855	0,103287	0,782234
Eosino	Mitral	ebi-a-GCST	hDNVJo	451679	rs7443170	-0,03419	0,10337	0,740845
Eosino	Mitral	ebi-a-GCST	hDNVJo	451679	rs7448010	-0,02173	0,103487	0,833714
Eosino	Mitral	ebi-a-GCST	hDNVJo	451679	rs7461209	-0,0292	0,103411	0,777639
Eosino	Mitral	ebi-a-GCST	hDNVJo	451679	rs7498094	-0,02895	0,103245	0,779134
Eosino	Mitral	ebi-a-GCST	hDNVJo	451679	rs7569084	-0,03395	0,103289	0,74237
Eosino	Mitral	ebi-a-GCST	hDNVJo	451679	rs7611275	-0,03231	0,103268	0,754407
Eosino	Mitral	ebi-a-GCST	hDNVJo	451679	rs7646283	-0,02833	0,103354	0,78404
Eosino	Mitral	ebi-a-GCST	hDNVJo	451679	rs7647432	-0,02297	0,103272	0,823973
Eosino	Mitral	ebi-a-GCST	hDNVJo	451679	rs7649812	-0,03559	0,103258	0,730356
Eosino	Mitral	ebi-a-GCST	hDNVJo	451679	rs7664390	-0,02707	0,103296	0,793274
Eosino	Mitral	ebi-a-GCST	hDNVJo	451679	rs7683096	-0,0281	0,103256	0,785529
Eosino	Mitral	ebi-a-GCST	hDNVJo	451679	rs7687708	-0,02883	0,103241	0,780081
Eosino	Mitral	ebi-a-GCST	hDNVJo	451679	rs778756	-0,03018	0,103266	0,770082
Eosino	Mitral	ebi-a-GCST	hDNVJo	451679	rs7792989	-0,03342	0,103241	0,746194
Eosino	Mitral	ebi-a-GCST	hDNVJo	451679	rs7802366	-0,03082	0,103262	0,765322
Eosino	Mitral	ebi-a-GCST	hDNVJo	451679	rs7805796	-0,03141	0,103272	0,761003
Eosino	Mitral	ebi-a-GCST	hDNVJo	451679	rs7840212	-0,02428	0,10355	0,814644
Eosino	Mitral	ebi-a-GCST	hDNVJo	451679	rs7846314	-0,0186	0,103371	0,857166
Eosino	Mitral	ebi-a-GCST	hDNVJo	451679	rs7869187	-0,03283	0,103256	0,750502
Eosino	Mitral	ebi-a-GCST	hDNVJo	451679	rs7897422	-0,03745	0,103272	0,716907

Eosino	Mitral	ebi-a-GCST	hDNVJo	451679	rs7898307	-0,03239	0,103245	0,753732
Eosino	Mitral	ebi-a-GCST	hDNVJo	451679	rs7918084	-0,04064	0,103422	0,694342
Eosino	Mitral	ebi-a-GCST	hDNVJo	451679	rs792627	-0,0362	0,103241	0,725876
Eosino	Mitral	ebi-a-GCST	hDNVJo	451679	rs7936323	-0,0432	0,103617	0,676744
Eosino	Mitral	ebi-a-GCST	hDNVJo	451679	rs7951663	-0,03085	0,103268	0,765128
Eosino	Mitral	ebi-a-GCST	hDNVJo	451679	rs7971658	-0,02862	0,103271	0,781702
Eosino	Mitral	ebi-a-GCST	hDNVJo	451679	rs798565	-0,02519	0,103239	0,807202
Eosino	Mitral	ebi-a-GCST	hDNVJo	451679	rs7998939	-0,03075	0,103373	0,766112
Eosino	Mitral	ebi-a-GCST	hDNVJo	451679	rs8005161	-0,02886	0,10324	0,779803
Eosino	Mitral	ebi-a-GCST	hDNVJo	451679	rs8005417	-0,03656	0,103358	0,723564
Eosino	Mitral	ebi-a-GCST	hDNVJo	451679	rs8044920	-0,0321	0,103238	0,755829
Eosino	Mitral	ebi-a-GCST	hDNVJo	451679	rs8083368	-0,02749	0,103248	0,790018
Eosino	Mitral	ebi-a-GCST	hDNVJo	451679	rs875109	-0,02937	0,103242	0,776036
Eosino	Mitral	ebi-a-GCST	hDNVJo	451679	rs911603	-0,02286	0,103344	0,824929
Eosino	Mitral	ebi-a-GCST	hDNVJo	451679	rs912131	-0,02884	0,103358	0,780223
Eosino	Mitral	ebi-a-GCST	hDNVJo	451679	rs9266321	-0,03841	0,103545	0,710701
Eosino	Mitral	ebi-a-GCST	hDNVJo	451679	rs9389268	-0,03065	0,103611	0,767402
Eosino	Mitral	ebi-a-GCST	hDNVJo	451679	rs9395112	-0,02614	0,103256	0,800126
Eosino	Mitral	ebi-a-GCST	hDNVJo	451679	rs9410887	-0,0297	0,103294	0,773675
Eosino	Mitral	ebi-a-GCST	hDNVJo	451679	rs941616	-0,03016	0,103241	0,770171
Eosino	Mitral	ebi-a-GCST	hDNVJo	451679	rs9430574	-0,03016	0,103297	0,770304
Eosino	Mitral	ebi-a-GCST	hDNVJo	451679	rs9504361	-0,03609	0,103429	0,727113
Eosino	Mitral	ebi-a-GCST	hDNVJo	451679	rs9517778	-0,0277	0,103241	0,78845
Eosino	Mitral	ebi-a-GCST	hDNVJo	451679	rs9533117	-0,02887	0,10327	0,77984
Eosino	Mitral	ebi-a-GCST	hDNVJo	451679	rs954954	-0,03145	0,103315	0,76081
Eosino	Mitral	ebi-a-GCST	hDNVJo	451679	rs9557201	-0,02162	0,103275	0,834168
Eosino	Mitral	ebi-a-GCST	hDNVJo	451679	rs964184	-0,02523	0,103296	0,807062
Eosino	Mitral	ebi-a-GCST	hDNVJo	451679	rs9666598	-0,02005	0,103261	0,84603
Eosino	Mitral	ebi-a-GCST	hDNVJo	451679	rs973471	-0,03075	0,103239	0,765825
Eosino	Mitral	ebi-a-GCST	hDNVJo	451679	rs9815874	-0,02576	0,103373	0,803217
Eosino	Mitral	ebi-a-GCST	hDNVJo	451679	rs9818987	-0,0289	0,103244	0,779554
Eosino	Mitral	ebi-a-GCST	hDNVJo	451679	rs9829214	-0,03273	0,103264	0,751273
Eosino	Mitral	ebi-a-GCST	hDNVJo	451679	rs9868582	-0,03468	0,103344	0,737219
Eosino	Mitral	ebi-a-GCST	hDNVJo	451679	rs9924483	-0,01981	0,103317	0,847929
Eosino	Mitral	ebi-a-GCST	hDNVJo	451679	rs9934736	-0,02752	0,103247	0,789807
Eosino	Mitral	ebi-a-GCST	hDNVJo	451679	rs9955853	-0,02795	0,103243	0,786588
Eosino	Mitral	ebi-a-GCST	hDNVJo	451679	rs9979383	-0,03315	0,103411	0,748554
Eosino	Mitral	ebi-a-GCST	hDNVJo	451679	All	-0,02975	0,103205	0,773182
Lym	Mitral	ebi-a-GCST	GISIJ4	456154	rs1006380	0,021459	0,109938	0,845242
Lym	Mitral	ebi-a-GCST	GISIJ4	456154	rs1017341	0,005565	0,11016	0,959709
Lym	Mitral	ebi-a-GCST	GISIJ4	456154	rs1023752	0,008424	0,10993	0,938918
Lym	Mitral	ebi-a-GCST	GISIJ4	456154	rs1027589	0,004638	0,109876	0,966328
Lym	Mitral	ebi-a-GCST	GISIJ4	456154	rs1034564	0,008693	0,109951	0,936986
Lym	Mitral	ebi-a-GCST	GISIJ4	456154	rs1046690	0,026327	0,11049	0,811671
Lym	Mitral	ebi-a-GCST	GISIJ4	456154	rs1048005	0,014719	0,109901	0,89346
Lym	Mitral	ebi-a-GCST	GISIJ4	456154	rs1049478	0,004736	0,109996	0,96566
Lym	Mitral	ebi-a-GCST	GISIJ4	456154	rs1076226	0,014415	0,109947	0,895688
Lym	Mitral	ebi-a-GCST	GISIJ4	456154	rs1079417	0,001188	0,109968	0,99138
Lym	Mitral	ebi-a-GCST	GISIJ4	456154	rs1080563	0,008382	0,109875	0,939191
Lym	Mitral	ebi-a-GCST	GISIJ4	456154	rs1081509	0,011441	0,109883	0,917077

Lym	Mitral	ebi-a-GCST	GISIJ4	456154	rs1082363[illegible]	0,011063	0,109922	0,919833
Lym	Mitral	ebi-a-GCST	GISIJ4	456154	rs1082872[illegible]	0,0013	0,110128	0,990579
Lym	Mitral	ebi-a-GCST	GISIJ4	456154	rs1083173[illegible]	0,014717	0,109875	0,893447
Lym	Mitral	ebi-a-GCST	GISIJ4	456154	rs1083863[illegible]	0,011535	0,109901	0,916407
Lym	Mitral	ebi-a-GCST	GISIJ4	456154	rs1084470[illegible]	0,04368	0,111197	0,694454
Lym	Mitral	ebi-a-GCST	GISIJ4	456154	rs1087537[illegible]	0,012608	0,109966	0,90872
Lym	Mitral	ebi-a-GCST	GISIJ4	456154	rs1093710[illegible]	0,015315	0,109883	0,889153
Lym	Mitral	ebi-a-GCST	GISIJ4	456154	rs1095640[illegible]	0,004223	0,110002	0,969373
Lym	Mitral	ebi-a-GCST	GISIJ4	456154	rs1098591[illegible]	0,00835	0,109892	0,939434
Lym	Mitral	ebi-a-GCST	GISIJ4	456154	rs1108409[illegible]	0,008556	0,109911	0,937949
Lym	Mitral	ebi-a-GCST	GISIJ4	456154	rs1111890	0,00738	0,109975	0,946494
Lym	Mitral	ebi-a-GCST	GISIJ4	456154	rs1112929[illegible]	0,018442	0,109989	0,866845
Lym	Mitral	ebi-a-GCST	GISIJ4	456154	rs1115008[illegible]	0,0083	0,109891	0,939797
Lym	Mitral	ebi-a-GCST	GISIJ4	456154	rs1116824[illegible]	0,009989	0,109929	0,927601
Lym	Mitral	ebi-a-GCST	GISIJ4	456154	rs1122820[illegible]	0,009282	0,109983	0,932743
Lym	Mitral	ebi-a-GCST	GISIJ4	456154	rs1124771[illegible]	0,014728	0,109947	0,893438
Lym	Mitral	ebi-a-GCST	GISIJ4	456154	rs1127379[illegible]	0,008083	0,109884	0,94136
Lym	Mitral	ebi-a-GCST	GISIJ4	456154	rs1129926[illegible]	0,005447	0,109872	0,960459
Lym	Mitral	ebi-a-GCST	GISIJ4	456154	rs1133963[illegible]	0,012174	0,109872	0,911777
Lym	Mitral	ebi-a-GCST	GISIJ4	456154	rs1135423[illegible]	0,009138	0,110219	0,933926
Lym	Mitral	ebi-a-GCST	GISIJ4	456154	rs1136058[illegible]	0,013772	0,109877	0,900252
Lym	Mitral	ebi-a-GCST	GISIJ4	456154	rs1154190[illegible]	0,013163	0,109951	0,90471
Lym	Mitral	ebi-a-GCST	GISIJ4	456154	rs1156769[illegible]	0,028031	0,110537	0,799817
Lym	Mitral	ebi-a-GCST	GISIJ4	456154	rs1158422[illegible]	0,01084	0,109872	0,921406
Lym	Mitral	ebi-a-GCST	GISIJ4	456154	rs1159305[illegible]	0,005558	0,109886	0,959663
Lym	Mitral	ebi-a-GCST	GISIJ4	456154	rs1164322[illegible]	0,00726	0,109902	0,947328
Lym	Mitral	ebi-a-GCST	GISIJ4	456154	rs1165276[illegible]	0,012966	0,109898	0,90608
Lym	Mitral	ebi-a-GCST	GISIJ4	456154	rs1167629[illegible]	0,006026	0,109953	0,956292
Lym	Mitral	ebi-a-GCST	GISIJ4	456154	rs1168830[illegible]	0,012314	0,10988	0,91077
Lym	Mitral	ebi-a-GCST	GISIJ4	456154	rs1170638[illegible]	0,010273	0,109973	0,925572
Lym	Mitral	ebi-a-GCST	GISIJ4	456154	rs1171233[illegible]	0,00929	0,109891	0,932626
Lym	Mitral	ebi-a-GCST	GISIJ4	456154	rs1175341[illegible]	0,009579	0,109874	0,930525
Lym	Mitral	ebi-a-GCST	GISIJ4	456154	rs1175561[illegible]	0,001915	0,109959	0,986104
Lym	Mitral	ebi-a-GCST	GISIJ4	456154	rs1179341[illegible]	0,010165	0,109909	0,926315
Lym	Mitral	ebi-a-GCST	GISIJ4	456154	rs1184817[illegible]	0,01354	0,110055	0,902084
Lym	Mitral	ebi-a-GCST	GISIJ4	456154	rs1185592[illegible]	0,005328	0,109917	0,961336
Lym	Mitral	ebi-a-GCST	GISIJ4	456154	rs1188894[illegible]	0,009094	0,109886	0,934046
Lym	Mitral	ebi-a-GCST	GISIJ4	456154	rs1192657[illegible]	0,008801	0,109875	0,936155
Lym	Mitral	ebi-a-GCST	GISIJ4	456154	rs1202180[illegible]	0,013251	0,109915	0,904039
Lym	Mitral	ebi-a-GCST	GISIJ4	456154	rs1212826[illegible]	0,012293	0,109972	0,910992
Lym	Mitral	ebi-a-GCST	GISIJ4	456154	rs1220821[illegible]	0,004432	0,109906	0,967837
Lym	Mitral	ebi-a-GCST	GISIJ4	456154	rs1233667[illegible]	0,007557	0,109887	0,945172
Lym	Mitral	ebi-a-GCST	GISIJ4	456154	rs1248573[illegible]	0,013044	0,109913	0,905531
Lym	Mitral	ebi-a-GCST	GISIJ4	456154	rs1252669[illegible]	0,012381	0,110074	0,910441
Lym	Mitral	ebi-a-GCST	GISIJ4	456154	rs1254290[illegible]	0,007255	0,109877	0,947352
Lym	Mitral	ebi-a-GCST	GISIJ4	456154	rs1260326	0,013896	0,10999	0,899462
Lym	Mitral	ebi-a-GCST	GISIJ4	456154	rs1263692[illegible]	0,008854	0,109873	0,935773
Lym	Mitral	ebi-a-GCST	GISIJ4	456154	rs1264018[illegible]	0,016285	0,109876	0,882177
Lym	Mitral	ebi-a-GCST	GISIJ4	456154	rs1274541[illegible]	0,011462	0,109919	0,916951
Lym	Mitral	ebi-a-GCST	GISIJ4	456154	rs1279932[illegible]	0,012437	0,109877	0,909879

Lym	Mitral	ebi-a-GCST	GISIJ4	456154	rs1281682(	0,008578	0,109892	0,93778
Lym	Mitral	ebi-a-GCST	GISIJ4	456154	rs1288213(	0,009258	0,109911	0,93287
Lym	Mitral	ebi-a-GCST	GISIJ4	456154	rs1294142ﾅ	0,010711	0,109873	0,922343
Lym	Mitral	ebi-a-GCST	GISIJ4	456154	rs1295927	0,001862	0,109977	0,98649
Lym	Mitral	ebi-a-GCST	GISIJ4	456154	rs1297453	0,009902	0,110199	0,928405
Lym	Mitral	ebi-a-GCST	GISIJ4	456154	rs1299118	0,008765	0,109885	0,936421
Lym	Mitral	ebi-a-GCST	GISIJ4	456154	rs1301683	0,007817	0,109906	0,943298
Lym	Mitral	ebi-a-GCST	GISIJ4	456154	rs1301817	0,006885	0,109879	0,95004
Lym	Mitral	ebi-a-GCST	GISIJ4	456154	rs1301926(	0,008545	0,110072	0,938119
Lym	Mitral	ebi-a-GCST	GISIJ4	456154	rs1304549	0,016099	0,109916	0,883553
Lym	Mitral	ebi-a-GCST	GISIJ4	456154	rs1306357	0,016322	0,11066	0,88274
Lym	Mitral	ebi-a-GCST	GISIJ4	456154	rs1310598	0,012185	0,110418	0,912126
Lym	Mitral	ebi-a-GCST	GISIJ4	456154	rs1319247(	0,003426	0,109887	0,975131
Lym	Mitral	ebi-a-GCST	GISIJ4	456154	rs1386623	0,020515	0,111015	0,853387
Lym	Mitral	ebi-a-GCST	GISIJ4	456154	rs1399746	0,00488	0,109916	0,964585
Lym	Mitral	ebi-a-GCST	GISIJ4	456154	rs1407590	0,011889	0,109916	0,913868
Lym	Mitral	ebi-a-GCST	GISIJ4	456154	rs1423965	0,011581	0,109869	0,916056
Lym	Mitral	ebi-a-GCST	GISIJ4	456154	rs1432833	0,007037	0,109916	0,94895
Lym	Mitral	ebi-a-GCST	GISIJ4	456154	rs1438751	0,011608	0,109888	0,915873
Lym	Mitral	ebi-a-GCST	GISIJ4	456154	rs1441850	0,015081	0,110423	0,891369
Lym	Mitral	ebi-a-GCST	GISIJ4	456154	rs1456059	0,013278	0,109876	0,903816
Lym	Mitral	ebi-a-GCST	GISIJ4	456154	rs1486015	0,0145	0,109872	0,895006
Lym	Mitral	ebi-a-GCST	GISIJ4	456154	rs1491664	0,011261	0,109882	0,918373
Lym	Mitral	ebi-a-GCST	GISIJ4	456154	rs1494539	0,007036	0,109887	0,948945
Lym	Mitral	ebi-a-GCST	GISIJ4	456154	rs1506400	0,009092	0,109897	0,934068
Lym	Mitral	ebi-a-GCST	GISIJ4	456154	rs151912	0,016263	0,109881	0,882338
Lym	Mitral	ebi-a-GCST	GISIJ4	456154	rs1567124	0,005861	0,109889	0,957467
Lym	Mitral	ebi-a-GCST	GISIJ4	456154	rs1611236	0,021512	0,110335	0,845418
Lym	Mitral	ebi-a-GCST	GISIJ4	456154	rs1677490	0,014747	0,10988	0,893235
Lym	Mitral	ebi-a-GCST	GISIJ4	456154	rs168293	0,014252	0,109873	0,896794
Lym	Mitral	ebi-a-GCST	GISIJ4	456154	rs1687045	0,008945	0,109884	0,935119
Lym	Mitral	ebi-a-GCST	GISIJ4	456154	rs1697816	0,014334	0,109948	0,896271
Lym	Mitral	ebi-a-GCST	GISIJ4	456154	rs1700589	0,011287	0,110219	0,918436
Lym	Mitral	ebi-a-GCST	GISIJ4	456154	rs1704143	0,010261	0,109945	0,925639
Lym	Mitral	ebi-a-GCST	GISIJ4	456154	rs1705581	0,012236	0,110058	0,911472
Lym	Mitral	ebi-a-GCST	GISIJ4	456154	rs17119	0,010891	0,109891	0,92105
Lym	Mitral	ebi-a-GCST	GISIJ4	456154	rs1722904	0,009187	0,109901	0,933382
Lym	Mitral	ebi-a-GCST	GISIJ4	456154	rs1750769	0,007987	0,109891	0,942057
Lym	Mitral	ebi-a-GCST	GISIJ4	456154	rs175714	0,007988	0,11004	0,942129
Lym	Mitral	ebi-a-GCST	GISIJ4	456154	rs1770326	0,011832	0,109903	0,914265
Lym	Mitral	ebi-a-GCST	GISIJ4	456154	rs1771000	0,002061	0,109904	0,985037
Lym	Mitral	ebi-a-GCST	GISIJ4	456154	rs1780334	0,013688	0,109877	0,900858
Lym	Mitral	ebi-a-GCST	GISIJ4	456154	rs1783233	0,004014	0,109941	0,970879
Lym	Mitral	ebi-a-GCST	GISIJ4	456154	rs179383	0,009269	0,109894	0,932781
Lym	Mitral	ebi-a-GCST	GISIJ4	456154	rs1800057	0,010781	0,109882	0,92184
Lym	Mitral	ebi-a-GCST	GISIJ4	456154	rs1862191	0,008639	0,109875	0,937329
Lym	Mitral	ebi-a-GCST	GISIJ4	456154	rs1862409	0,009431	0,109919	0,931625
Lym	Mitral	ebi-a-GCST	GISIJ4	456154	rs1878036	0,017265	0,109891	0,875159
Lym	Mitral	ebi-a-GCST	GISIJ4	456154	rs1886268	0,007912	0,109891	0,942604
Lym	Mitral	ebi-a-GCST	GISIJ4	456154	rs1893592	0,004242	0,109897	0,96921

Lym	Mitral	ebi-a-GCST	GISIJ4	456154	rs1900942	0,012415	0,109886	0,910046
Lym	Mitral	ebi-a-GCST	GISIJ4	456154	rs1935007	0,01419	0,109899	0,897262
Lym	Mitral	ebi-a-GCST	GISIJ4	456154	rs1976055	0,01388	0,109878	0,899479
Lym	Mitral	ebi-a-GCST	GISIJ4	456154	rs1976451	0,008469	0,109872	0,938559
Lym	Mitral	ebi-a-GCST	GISIJ4	456154	rs1997577	0,013647	0,109881	0,901155
Lym	Mitral	ebi-a-GCST	GISIJ4	456154	rs202638	0,010725	0,109969	0,922311
Lym	Mitral	ebi-a-GCST	GISIJ4	456154	rs2041756	0,011174	0,109988	0,91908
Lym	Mitral	ebi-a-GCST	GISIJ4	456154	rs2057340	0,010919	0,109899	0,92086
Lym	Mitral	ebi-a-GCST	GISIJ4	456154	rs2060550	0,008693	0,109875	0,936942
Lym	Mitral	ebi-a-GCST	GISIJ4	456154	rs2065500	0,014601	0,110245	0,894634
Lym	Mitral	ebi-a-GCST	GISIJ4	456154	rs2070745	0,009737	0,109903	0,929403
Lym	Mitral	ebi-a-GCST	GISIJ4	456154	rs2075302	0,006664	0,109897	0,95165
Lym	Mitral	ebi-a-GCST	GISIJ4	456154	rs2089979	0,003675	0,11019	0,973392
Lym	Mitral	ebi-a-GCST	GISIJ4	456154	rs2091084	0,013766	0,10988	0,900304
Lym	Mitral	ebi-a-GCST	GISIJ4	456154	rs2155433	0,009217	0,109875	0,933146
Lym	Mitral	ebi-a-GCST	GISIJ4	456154	rs2157691	0,017959	0,110075	0,870398
Lym	Mitral	ebi-a-GCST	GISIJ4	456154	rs2235413	0,008092	0,109876	0,941293
Lym	Mitral	ebi-a-GCST	GISIJ4	456154	rs2237040	0,014124	0,109933	0,897773
Lym	Mitral	ebi-a-GCST	GISIJ4	456154	rs2249742	0,018061	0,111988	0,871876
Lym	Mitral	ebi-a-GCST	GISIJ4	456154	rs2286383	0,011404	0,109872	0,917331
Lym	Mitral	ebi-a-GCST	GISIJ4	456154	rs2291900	0,006422	0,109898	0,953404
Lym	Mitral	ebi-a-GCST	GISIJ4	456154	rs2294861	0,011111	0,10991	0,919474
Lym	Mitral	ebi-a-GCST	GISIJ4	456154	rs2297508	0,010096	0,109977	0,926853
Lym	Mitral	ebi-a-GCST	GISIJ4	456154	rs2298850	0,007432	0,109892	0,946083
Lym	Mitral	ebi-a-GCST	GISIJ4	456154	rs2313571	-0,00203	0,109926	0,985266
Lym	Mitral	ebi-a-GCST	GISIJ4	456154	rs2327531	0,007201	0,109891	0,947752
Lym	Mitral	ebi-a-GCST	GISIJ4	456154	rs2369391	0,016653	0,109924	0,879586
Lym	Mitral	ebi-a-GCST	GISIJ4	456154	rs2375636	0,01035	0,109926	0,924988
Lym	Mitral	ebi-a-GCST	GISIJ4	456154	rs2408014	0,010822	0,109893	0,921551
Lym	Mitral	ebi-a-GCST	GISIJ4	456154	rs2453582	0,007306	0,109925	0,947008
Lym	Mitral	ebi-a-GCST	GISIJ4	456154	rs247555	0,013108	0,109876	0,905039
Lym	Mitral	ebi-a-GCST	GISIJ4	456154	rs247826	0,006163	0,11011	0,955364
Lym	Mitral	ebi-a-GCST	GISIJ4	456154	rs251390	0,015981	0,109931	0,884413
Lym	Mitral	ebi-a-GCST	GISIJ4	456154	rs251398	0,012925	0,109921	0,906398
Lym	Mitral	ebi-a-GCST	GISIJ4	456154	rs2523625	0,018459	0,110717	0,867589
Lym	Mitral	ebi-a-GCST	GISIJ4	456154	rs2530482	0,007852	0,109885	0,943037
Lym	Mitral	ebi-a-GCST	GISIJ4	456154	rs2544920	0,010227	0,109888	0,925849
Lym	Mitral	ebi-a-GCST	GISIJ4	456154	rs2548499	0,013515	0,109908	0,902134
Lym	Mitral	ebi-a-GCST	GISIJ4	456154	rs259981	0,006271	0,110036	0,954555
Lym	Mitral	ebi-a-GCST	GISIJ4	456154	rs2635119	0,013054	0,109906	0,905455
Lym	Mitral	ebi-a-GCST	GISIJ4	456154	rs2647929	0,004971	0,109893	0,96392
Lym	Mitral	ebi-a-GCST	GISIJ4	456154	rs2665967	0,002517	0,109881	0,981723
Lym	Mitral	ebi-a-GCST	GISIJ4	456154	rs2710804	0,007798	0,109893	0,943432
Lym	Mitral	ebi-a-GCST	GISIJ4	456154	rs2727487	0,012327	0,109884	0,910681
Lym	Mitral	ebi-a-GCST	GISIJ4	456154	rs2755253	0,014151	0,110079	0,897713
Lym	Mitral	ebi-a-GCST	GISIJ4	456154	rs2791737	0,008029	0,109897	0,941759
Lym	Mitral	ebi-a-GCST	GISIJ4	456154	rs2800883	0,011636	0,109886	0,915669
Lym	Mitral	ebi-a-GCST	GISIJ4	456154	rs2807303	0,004399	0,109958	0,968085
Lym	Mitral	ebi-a-GCST	GISIJ4	456154	rs284324	0,006278	0,109874	0,954435
Lym	Mitral	ebi-a-GCST	GISIJ4	456154	rs2848573(	0,008874	0,109876	0,935628

Lym	Mitral	ebi-a-GCST	GISIJ4	456154	rs2853937:	0,000542	0,110245	0,996081
Lym	Mitral	ebi-a-GCST	GISIJ4	456154	rs2858874!	0,008784	0,109944	0,936323
Lym	Mitral	ebi-a-GCST	GISIJ4	456154	rs2920880	0,009707	0,109901	0,929619
Lym	Mitral	ebi-a-GCST	GISIJ4	456154	rs2954025	0,00754	0,109895	0,945303
Lym	Mitral	ebi-a-GCST	GISIJ4	456154	rs3014983	0,010321	0,109904	0,92518
Lym	Mitral	ebi-a-GCST	GISIJ4	456154	rs301817	0,008134	0,109937	0,941024
Lym	Mitral	ebi-a-GCST	GISIJ4	456154	rs309375	0,000411	0,10992	0,99702
Lym	Mitral	ebi-a-GCST	GISIJ4	456154	rs3184504	0,011791	0,112452	0,916493
Lym	Mitral	ebi-a-GCST	GISIJ4	456154	rs318493	0,008838	0,109872	0,93589
Lym	Mitral	ebi-a-GCST	GISIJ4	456154	rs332507	0,011492	0,109896	0,916712
Lym	Mitral	ebi-a-GCST	GISIJ4	456154	rs3402507:	0,010373	0,109952	0,924841
Lym	Mitral	ebi-a-GCST	GISIJ4	456154	rs3403081:	0,016124	0,109932	0,88339
Lym	Mitral	ebi-a-GCST	GISIJ4	456154	rs3406660{	0,004193	0,109957	0,969579
Lym	Mitral	ebi-a-GCST	GISIJ4	456154	rs3415993{	0,010906	0,110052	0,921062
Lym	Mitral	ebi-a-GCST	GISIJ4	456154	rs3423028:	0,005822	0,110042	0,957808
Lym	Mitral	ebi-a-GCST	GISIJ4	456154	rs3432394:	0,007203	0,109892	0,94774
Lym	Mitral	ebi-a-GCST	GISIJ4	456154	rs343829	0,013068	0,10989	0,905342
Lym	Mitral	ebi-a-GCST	GISIJ4	456154	rs3489156:	0,010067	0,10989	0,927008
Lym	Mitral	ebi-a-GCST	GISIJ4	456154	rs3501078(	0,009316	0,109891	0,93244
Lym	Mitral	ebi-a-GCST	GISIJ4	456154	rs3534575:	0,009316	0,109973	0,932491
Lym	Mitral	ebi-a-GCST	GISIJ4	456154	rs3559243:	0,00572	0,110124	0,958573
Lym	Mitral	ebi-a-GCST	GISIJ4	456154	rs3589614(	0,006196	0,10992	0,955046
Lym	Mitral	ebi-a-GCST	GISIJ4	456154	rs3599384(	0,01445	0,109931	0,89542
Lym	Mitral	ebi-a-GCST	GISIJ4	456154	rs360017	0,022423	0,10993	0,83837
Lym	Mitral	ebi-a-GCST	GISIJ4	456154	rs3731211	0,020217	0,110348	0,854632
Lym	Mitral	ebi-a-GCST	GISIJ4	456154	rs3735485	0,010474	0,110151	0,924248
Lym	Mitral	ebi-a-GCST	GISIJ4	456154	rs3761846	0,00996	0,109962	0,927829
Lym	Mitral	ebi-a-GCST	GISIJ4	456154	rs3769818	0,006633	0,110084	0,951951
Lym	Mitral	ebi-a-GCST	GISIJ4	456154	rs3812849	0,002937	0,110284	0,978754
Lym	Mitral	ebi-a-GCST	GISIJ4	456154	rs3815725	0,008167	0,109907	0,940768
Lym	Mitral	ebi-a-GCST	GISIJ4	456154	rs3824667	0,008419	0,109949	0,938962
Lym	Mitral	ebi-a-GCST	GISIJ4	456154	rs3828188	0,012049	0,109961	0,912747
Lym	Mitral	ebi-a-GCST	GISIJ4	456154	rs3851820	0,01174	0,109887	0,91492
Lym	Mitral	ebi-a-GCST	GISIJ4	456154	rs3861929	0,009356	0,109907	0,932164
Lym	Mitral	ebi-a-GCST	GISIJ4	456154	rs4068540	0,007076	0,109897	0,948662
Lym	Mitral	ebi-a-GCST	GISIJ4	456154	rs4126776!	0,01983	0,10989	0,856794
Lym	Mitral	ebi-a-GCST	GISIJ4	456154	rs4129534!	0,011625	0,109872	0,915737
Lym	Mitral	ebi-a-GCST	GISIJ4	456154	rs4130718:	0,01512	0,109885	0,890558
Lym	Mitral	ebi-a-GCST	GISIJ4	456154	rs4131727(	0,013931	0,10989	0,899121
Lym	Mitral	ebi-a-GCST	GISIJ4	456154	rs4149569	0,00227	0,109959	0,983531
Lym	Mitral	ebi-a-GCST	GISIJ4	456154	rs415297	0,009463	0,109902	0,931384
Lym	Mitral	ebi-a-GCST	GISIJ4	456154	rs419470	0,011137	0,109891	0,919277
Lym	Mitral	ebi-a-GCST	GISIJ4	456154	rs4261897	0,007843	0,109935	0,943124
Lym	Mitral	ebi-a-GCST	GISIJ4	456154	rs4288653	0,008156	0,109919	0,940853
Lym	Mitral	ebi-a-GCST	GISIJ4	456154	rs4407213	0,007041	0,109902	0,948917
Lym	Mitral	ebi-a-GCST	GISIJ4	456154	rs4411786	0,011778	0,110304	0,914968
Lym	Mitral	ebi-a-GCST	GISIJ4	456154	rs447361	0,010155	0,109932	0,926403
Lym	Mitral	ebi-a-GCST	GISIJ4	456154	rs4530278	0,007439	0,10993	0,946046
Lym	Mitral	ebi-a-GCST	GISIJ4	456154	rs4552893⁄	0,014816	0,109889	0,892747
Lym	Mitral	ebi-a-GCST	GISIJ4	456154	rs4553073!	0,011523	0,109907	0,916503

Lym	Mitral	ebi-a-GCST	GISIJ4	456154	rs4583569	0,016125	0,109967	0,883419
Lym	Mitral	ebi-a-GCST	GISIJ4	456154	rs4596195	0,004217	0,110115	0,969453
Lym	Mitral	ebi-a-GCST	GISIJ4	456154	rs4659611	0,00831	0,109902	0,939724
Lym	Mitral	ebi-a-GCST	GISIJ4	456154	rs4664108	0,013795	0,109876	0,900084
Lym	Mitral	ebi-a-GCST	GISIJ4	456154	rs4676482	0,010103	0,109878	0,926742
Lym	Mitral	ebi-a-GCST	GISIJ4	456154	rs4696314	0,007156	0,109903	0,948086
Lym	Mitral	ebi-a-GCST	GISIJ4	456154	rs4718976	0,009487	0,109894	0,931206
Lym	Mitral	ebi-a-GCST	GISIJ4	456154	rs4719922	0,009747	0,109948	0,929361
Lym	Mitral	ebi-a-GCST	GISIJ4	456154	rs4737010	0,010137	0,109992	0,926572
Lym	Mitral	ebi-a-GCST	GISIJ4	456154	rs4756265	0,015678	0,109941	0,886604
Lym	Mitral	ebi-a-GCST	GISIJ4	456154	rs4789299	0,01676	0,11	0,878903
Lym	Mitral	ebi-a-GCST	GISIJ4	456154	rs4794859	0,010963	0,109927	0,920556
Lym	Mitral	ebi-a-GCST	GISIJ4	456154	rs4795397	0,013726	0,110115	0,900801
Lym	Mitral	ebi-a-GCST	GISIJ4	456154	rs4805881	0,01764	0,10989	0,872466
Lym	Mitral	ebi-a-GCST	GISIJ4	456154	rs4823073	0,01644	0,110197	0,881409
Lym	Mitral	ebi-a-GCST	GISIJ4	456154	rs4833031	0,012514	0,109872	0,909319
Lym	Mitral	ebi-a-GCST	GISIJ4	456154	rs4852777	0,015619	0,10988	0,886962
Lym	Mitral	ebi-a-GCST	GISIJ4	456154	rs4890888	0,00903	0,109881	0,934507
Lym	Mitral	ebi-a-GCST	GISIJ4	456154	rs4907236	0,017099	0,109875	0,876329
Lym	Mitral	ebi-a-GCST	GISIJ4	456154	rs4913407	0,010033	0,109895	0,927256
Lym	Mitral	ebi-a-GCST	GISIJ4	456154	rs4940572	0,010417	0,109909	0,924489
Lym	Mitral	ebi-a-GCST	GISIJ4	456154	rs4948492	0,015726	0,109978	0,886296
Lym	Mitral	ebi-a-GCST	GISIJ4	456154	rs4970926	0,005985	0,110052	0,956628
Lym	Mitral	ebi-a-GCST	GISIJ4	456154	rs5011302	0,013089	0,109949	0,905236
Lym	Mitral	ebi-a-GCST	GISIJ4	456154	rs5498	-0,01681	0,110406	0,879004
Lym	Mitral	ebi-a-GCST	GISIJ4	456154	rs5570028	0,01194	0,109958	0,913529
Lym	Mitral	ebi-a-GCST	GISIJ4	456154	rs5570644	0,007003	0,11017	0,949313
Lym	Mitral	ebi-a-GCST	GISIJ4	456154	rs5572491	0,018559	0,109891	0,865883
Lym	Mitral	ebi-a-GCST	GISIJ4	456154	rs559771	0,011345	0,109875	0,917759
Lym	Mitral	ebi-a-GCST	GISIJ4	456154	rs5611684	0,011975	0,109944	0,91327
Lym	Mitral	ebi-a-GCST	GISIJ4	456154	rs5631062	0,004829	0,109872	0,964942
Lym	Mitral	ebi-a-GCST	GISIJ4	456154	rs5639499	0,008728	0,109952	0,936733
Lym	Mitral	ebi-a-GCST	GISIJ4	456154	rs5741699	0,0085	0,109894	0,93835
Lym	Mitral	ebi-a-GCST	GISIJ4	456154	rs574185	0,006141	0,109921	0,955448
Lym	Mitral	ebi-a-GCST	GISIJ4	456154	rs5762813	0,016061	0,10988	0,883789
Lym	Mitral	ebi-a-GCST	GISIJ4	456154	rs5810659	0,010744	0,109949	0,922153
Lym	Mitral	ebi-a-GCST	GISIJ4	456154	rs5843277	0,008694	0,110121	0,93707
Lym	Mitral	ebi-a-GCST	GISIJ4	456154	rs5883538	0,017051	0,10998	0,87679
Lym	Mitral	ebi-a-GCST	GISIJ4	456154	rs5910703	-0,00123	0,109965	0,991098
Lym	Mitral	ebi-a-GCST	GISIJ4	456154	rs6058094	0,009968	0,109888	0,92772
Lym	Mitral	ebi-a-GCST	GISIJ4	456154	rs6064762	0,013497	0,109885	0,902244
Lym	Mitral	ebi-a-GCST	GISIJ4	456154	rs6072080	0,02285	0,109949	0,835369
Lym	Mitral	ebi-a-GCST	GISIJ4	456154	rs6138719	0,009865	0,109976	0,928527
Lym	Mitral	ebi-a-GCST	GISIJ4	456154	rs6175092	0,016401	0,110184	0,881672
Lym	Mitral	ebi-a-GCST	GISIJ4	456154	rs6180229	0,014476	0,109904	0,895212
Lym	Mitral	ebi-a-GCST	GISIJ4	456154	rs6183966	0,009148	0,110056	0,933753
Lym	Mitral	ebi-a-GCST	GISIJ4	456154	rs6185068	0,006009	0,110072	0,956462
Lym	Mitral	ebi-a-GCST	GISIJ4	456154	rs6186376	0,010408	0,109896	0,92455
Lym	Mitral	ebi-a-GCST	GISIJ4	456154	rs6186714	0,01021	0,109903	0,925986
Lym	Mitral	ebi-a-GCST	GISIJ4	456154	rs6207363	0,006423	0,110012	0,953446

Lym	Mitral	ebi-a-GCST	GISIJ4	456154	rs6209199ε	0,00396	0,109985	0,97128
Lym	Mitral	ebi-a-GCST	GISIJ4	456154	rs6218025ℷ	-8,17E-05	0,110425	0,99941
Lym	Mitral	ebi-a-GCST	GISIJ4	456154	rs6233276ℷ	0,010223	0,110035	0,925978
Lym	Mitral	ebi-a-GCST	GISIJ4	456154	rs6248590!	0,014162	0,109927	0,897489
Lym	Mitral	ebi-a-GCST	GISIJ4	456154	rs6262181ℷ	0,011594	0,109902	0,915985
Lym	Mitral	ebi-a-GCST	GISIJ4	456154	rs631864	0,010952	0,109888	0,920609
Lym	Mitral	ebi-a-GCST	GISIJ4	456154	rs6474359	0,002811	0,109932	0,979602
Lym	Mitral	ebi-a-GCST	GISIJ4	456154	rs6475476	0,011198	0,109875	0,918824
Lym	Mitral	ebi-a-GCST	GISIJ4	456154	rs6488548	0,008913	0,109934	0,935381
Lym	Mitral	ebi-a-GCST	GISIJ4	456154	rs6488868	0,004924	0,109996	0,964295
Lym	Mitral	ebi-a-GCST	GISIJ4	456154	rs6502555	0,022495	0,110022	0,837996
Lym	Mitral	ebi-a-GCST	GISIJ4	456154	rs6531603	0,016375	0,109962	0,88162
Lym	Mitral	ebi-a-GCST	GISIJ4	456154	rs6538189	0,014824	0,109888	0,892688
Lym	Mitral	ebi-a-GCST	GISIJ4	456154	rs6545377	0,01035	0,109884	0,924959
Lym	Mitral	ebi-a-GCST	GISIJ4	456154	rs6557156	0,013436	0,109879	0,902679
Lym	Mitral	ebi-a-GCST	GISIJ4	456154	rs6586777	0,014946	0,109882	0,891806
Lym	Mitral	ebi-a-GCST	GISIJ4	456154	rs6679677	0,029719	0,110189	0,787383
Lym	Mitral	ebi-a-GCST	GISIJ4	456154	rs6696259	0,018128	0,110178	0,86931
Lym	Mitral	ebi-a-GCST	GISIJ4	456154	rs6700896	0,008243	0,109923	0,940225
Lym	Mitral	ebi-a-GCST	GISIJ4	456154	rs6721663	0,008735	0,109939	0,93667
Lym	Mitral	ebi-a-GCST	GISIJ4	456154	rs6727950ⁱ	0,007867	0,109874	0,942917
Lym	Mitral	ebi-a-GCST	GISIJ4	456154	rs6748379ℷ	0,008942	0,109891	0,935146
Lym	Mitral	ebi-a-GCST	GISIJ4	456154	rs6751671ℷ	0,008563	0,109907	0,937899
Lym	Mitral	ebi-a-GCST	GISIJ4	456154	rs6771541	0,013172	0,109892	0,904592
Lym	Mitral	ebi-a-GCST	GISIJ4	456154	rs6782672	-0,00098	0,109899	0,992859
Lym	Mitral	ebi-a-GCST	GISIJ4	456154	rs678393	0,006741	0,109892	0,951088
Lym	Mitral	ebi-a-GCST	GISIJ4	456154	rs6796	0,008632	0,110024	0,937468
Lym	Mitral	ebi-a-GCST	GISIJ4	456154	rs6859682	0,010734	0,10988	0,922178
Lym	Mitral	ebi-a-GCST	GISIJ4	456154	rs6882776	0,014494	0,109878	0,895056
Lym	Mitral	ebi-a-GCST	GISIJ4	456154	rs6917738	0,006418	0,110013	0,953476
Lym	Mitral	ebi-a-GCST	GISIJ4	456154	rs6926219	0,007616	0,110018	0,944813
Lym	Mitral	ebi-a-GCST	GISIJ4	456154	rs696	0,001154	0,110031	0,991632
Lym	Mitral	ebi-a-GCST	GISIJ4	456154	rs6982175	0,012156	0,109895	0,911922
Lym	Mitral	ebi-a-GCST	GISIJ4	456154	rs6995717	0,010658	0,109883	0,922733
Lym	Mitral	ebi-a-GCST	GISIJ4	456154	rs7006802	0,011404	0,109879	0,917342
Lym	Mitral	ebi-a-GCST	GISIJ4	456154	rs7007986	0,014779	0,10997	0,893094
Lym	Mitral	ebi-a-GCST	GISIJ4	456154	rs7018333	0,007117	0,109873	0,948354
Lym	Mitral	ebi-a-GCST	GISIJ4	456154	rs7033677	0,012955	0,109929	0,906188
Lym	Mitral	ebi-a-GCST	GISIJ4	456154	rs7071131	0,006568	0,109914	0,95235
Lym	Mitral	ebi-a-GCST	GISIJ4	456154	rs7123436	0,022662	0,110109	0,836934
Lym	Mitral	ebi-a-GCST	GISIJ4	456154	rs7127911	0,009999	0,109979	0,927559
Lym	Mitral	ebi-a-GCST	GISIJ4	456154	rs7134738	0,013101	0,109907	0,905115
Lym	Mitral	ebi-a-GCST	GISIJ4	456154	rs7142083(	0,015232	0,109942	0,889809
Lym	Mitral	ebi-a-GCST	GISIJ4	456154	rs715	0,012147	0,109889	0,91198
Lym	Mitral	ebi-a-GCST	GISIJ4	456154	rs7157267	0,008669	0,11002	0,937196
Lym	Mitral	ebi-a-GCST	GISIJ4	456154	rs7161799	0,01005	0,109947	0,92717
Lym	Mitral	ebi-a-GCST	GISIJ4	456154	rs7192652	0,006434	0,109977	0,953351
Lym	Mitral	ebi-a-GCST	GISIJ4	456154	rs721131	0,017933	0,109938	0,870428
Lym	Mitral	ebi-a-GCST	GISIJ4	456154	rs7214308	0,00963	0,109884	0,930163
Lym	Mitral	ebi-a-GCST	GISIJ4	456154	rs7249236	0,011298	0,109951	0,918159

Lym	Mitral	ebi-a-GCST	GISIJ4	456154	rs7278167[illegible]	0,009007	0,110343	0,93494
Lym	Mitral	ebi-a-GCST	GISIJ4	456154	rs7279086[illegible]	0,006053	0,109912	0,956082
Lym	Mitral	ebi-a-GCST	GISIJ4	456154	rs7292803[illegible]	0,002192	0,110053	0,984112
Lym	Mitral	ebi-a-GCST	GISIJ4	456154	rs7306550[illegible]	0,004933	0,109876	0,964188
Lym	Mitral	ebi-a-GCST	GISIJ4	456154	rs7310856[illegible]	0,012217	0,109921	0,911501
Lym	Mitral	ebi-a-GCST	GISIJ4	456154	rs7311032[illegible]	0,002866	0,109887	0,979196
Lym	Mitral	ebi-a-GCST	GISIJ4	456154	rs7311267[illegible]	0,011484	0,109895	0,916773
Lym	Mitral	ebi-a-GCST	GISIJ4	456154	rs7313523[illegible]	0,014171	0,109876	0,897382
Lym	Mitral	ebi-a-GCST	GISIJ4	456154	rs7314229[illegible]	0,008222	0,109886	0,940354
Lym	Mitral	ebi-a-GCST	GISIJ4	456154	rs7316552[illegible]	0,011874	0,109876	0,913946
Lym	Mitral	ebi-a-GCST	GISIJ4	456154	rs7316663[illegible]	0,010356	0,110163	0,925105
Lym	Mitral	ebi-a-GCST	GISIJ4	456154	rs7323927[illegible]	0,018473	0,110026	0,866662
Lym	Mitral	ebi-a-GCST	GISIJ4	456154	rs7349830[illegible]	0,010192	0,10991	0,926116
Lym	Mitral	ebi-a-GCST	GISIJ4	456154	rs7356449[illegible]	0,013538	0,109897	0,90196
Lym	Mitral	ebi-a-GCST	GISIJ4	456154	rs7396171[illegible]	0,009485	0,110178	0,931398
Lym	Mitral	ebi-a-GCST	GISIJ4	456154	rs7422770[illegible]	0,012951	0,109903	0,906195
Lym	Mitral	ebi-a-GCST	GISIJ4	456154	rs7437961	0,014807	0,10988	0,892808
Lym	Mitral	ebi-a-GCST	GISIJ4	456154	rs7440139[illegible]	0,007719	0,1099	0,944005
Lym	Mitral	ebi-a-GCST	GISIJ4	456154	rs7440881[illegible]	0,012763	0,109876	0,907526
Lym	Mitral	ebi-a-GCST	GISIJ4	456154	rs7467931[illegible]	0,012459	0,109891	0,909734
Lym	Mitral	ebi-a-GCST	GISIJ4	456154	rs748113	0,013687	0,110195	0,901149
Lym	Mitral	ebi-a-GCST	GISIJ4	456154	rs7501849[illegible]	0,005934	0,109879	0,956933
Lym	Mitral	ebi-a-GCST	GISIJ4	456154	rs7503337[illegible]	0,009679	0,110055	0,929922
Lym	Mitral	ebi-a-GCST	GISIJ4	456154	rs753612	0,003341	0,109889	0,975749
Lym	Mitral	ebi-a-GCST	GISIJ4	456154	rs7548606	0,002852	0,109885	0,979295
Lym	Mitral	ebi-a-GCST	GISIJ4	456154	rs7548699[illegible]	0,013863	0,109872	0,899596
Lym	Mitral	ebi-a-GCST	GISIJ4	456154	rs7565358[illegible]	0,013456	0,109892	0,902541
Lym	Mitral	ebi-a-GCST	GISIJ4	456154	rs7572278	0,000632	0,109934	0,995415
Lym	Mitral	ebi-a-GCST	GISIJ4	456154	rs7592506[illegible]	0,013439	0,110037	0,902791
Lym	Mitral	ebi-a-GCST	GISIJ4	456154	rs7625643	0,001799	0,110006	0,986954
Lym	Mitral	ebi-a-GCST	GISIJ4	456154	rs7637449	0,008259	0,109902	0,940098
Lym	Mitral	ebi-a-GCST	GISIJ4	456154	rs7642810[illegible]	0,003329	0,109882	0,975832
Lym	Mitral	ebi-a-GCST	GISIJ4	456154	rs765063	0,007588	0,110064	0,945035
Lym	Mitral	ebi-a-GCST	GISIJ4	456154	rs7665147	0,009519	0,109929	0,930993
Lym	Mitral	ebi-a-GCST	GISIJ4	456154	rs7672879	0,011241	0,109879	0,918513
Lym	Mitral	ebi-a-GCST	GISIJ4	456154	rs7675031[illegible]	0,013564	0,109892	0,901764
Lym	Mitral	ebi-a-GCST	GISIJ4	456154	rs7685722[illegible]	0,012038	0,109872	0,912758
Lym	Mitral	ebi-a-GCST	GISIJ4	456154	rs7710156[illegible]	0,003951	0,109881	0,971317
Lym	Mitral	ebi-a-GCST	GISIJ4	456154	rs7723211[illegible]	0,012592	0,109884	0,908767
Lym	Mitral	ebi-a-GCST	GISIJ4	456154	rs7723497[illegible]	0,008307	0,109913	0,939757
Lym	Mitral	ebi-a-GCST	GISIJ4	456154	rs7726538[illegible]	0,007323	0,109893	0,946873
Lym	Mitral	ebi-a-GCST	GISIJ4	456154	rs7755128[illegible]	0,009031	0,109913	0,934514
Lym	Mitral	ebi-a-GCST	GISIJ4	456154	rs7776054	0,010356	0,110195	0,925126
Lym	Mitral	ebi-a-GCST	GISIJ4	456154	rs7780328	0,014223	0,109952	0,897077
Lym	Mitral	ebi-a-GCST	GISIJ4	456154	rs7793008	0,01318	0,109927	0,904561
Lym	Mitral	ebi-a-GCST	GISIJ4	456154	rs7800072	0,014277	0,109874	0,896618
Lym	Mitral	ebi-a-GCST	GISIJ4	456154	rs780669	0,015953	0,109884	0,884571
Lym	Mitral	ebi-a-GCST	GISIJ4	456154	rs7827009[illegible]	0,013835	0,109895	0,899813
Lym	Mitral	ebi-a-GCST	GISIJ4	456154	rs7898307[illegible]	0,007484	0,109882	0,945699
Lym	Mitral	ebi-a-GCST	GISIJ4	456154	rs7920164	0,01237	0,109874	0,91036

Lym	Mitral	ebi-a-GCST	GISIJ4	456154	rs7945319	0,010496	0,109887	0,923902
Lym	Mitral	ebi-a-GCST	GISIJ4	456154	rs7980539	0,003541	0,109884	0,974291
Lym	Mitral	ebi-a-GCST	GISIJ4	456154	rs8052370	0,014505	0,109971	0,895065
Lym	Mitral	ebi-a-GCST	GISIJ4	456154	rs8060375	0,009974	0,109882	0,927675
Lym	Mitral	ebi-a-GCST	GISIJ4	456154	rs817329	0,006456	0,109876	0,953146
Lym	Mitral	ebi-a-GCST	GISIJ4	456154	rs8176526	0,006418	0,109943	0,953453
Lym	Mitral	ebi-a-GCST	GISIJ4	456154	rs845329	0,007779	0,109873	0,943556
Lym	Mitral	ebi-a-GCST	GISIJ4	456154	rs852432	0,008022	0,109884	0,941803
Lym	Mitral	ebi-a-GCST	GISIJ4	456154	rs872071	0,011621	0,110019	0,915877
Lym	Mitral	ebi-a-GCST	GISIJ4	456154	rs894885	0,005177	0,109908	0,962428
Lym	Mitral	ebi-a-GCST	GISIJ4	456154	rs909349	0,006972	0,110281	0,949589
Lym	Mitral	ebi-a-GCST	GISIJ4	456154	rs912416	0,007669	0,109877	0,944355
Lym	Mitral	ebi-a-GCST	GISIJ4	456154	rs9258357	0,011688	0,110029	0,915399
Lym	Mitral	ebi-a-GCST	GISIJ4	456154	rs9316484	0,026424	0,109987	0,810135
Lym	Mitral	ebi-a-GCST	GISIJ4	456154	rs9392840	0,01233	0,109974	0,91073
Lym	Mitral	ebi-a-GCST	GISIJ4	456154	rs9396582	0,01244	0,109902	0,909879
Lym	Mitral	ebi-a-GCST	GISIJ4	456154	rs9430574	0,010261	0,109877	0,925599
Lym	Mitral	ebi-a-GCST	GISIJ4	456154	rs9438860	0,011698	0,109907	0,91524
Lym	Mitral	ebi-a-GCST	GISIJ4	456154	rs949349	0,007805	0,109877	0,943368
Lym	Mitral	ebi-a-GCST	GISIJ4	456154	rs9513573	0,013691	0,109883	0,90084
Lym	Mitral	ebi-a-GCST	GISIJ4	456154	rs9532679	0,013917	0,10998	0,899305
Lym	Mitral	ebi-a-GCST	GISIJ4	456154	rs9533100	0,010641	0,109959	0,922905
Lym	Mitral	ebi-a-GCST	GISIJ4	456154	rs9549811	0,01212	0,109872	0,912161
Lym	Mitral	ebi-a-GCST	GISIJ4	456154	rs9568053	0,00641	0,109877	0,953482
Lym	Mitral	ebi-a-GCST	GISIJ4	456154	rs9590390	0,006003	0,110049	0,956501
Lym	Mitral	ebi-a-GCST	GISIJ4	456154	rs9592965	0,008758	0,109887	0,936479
Lym	Mitral	ebi-a-GCST	GISIJ4	456154	rs9607869	0,014528	0,109889	0,894819
Lym	Mitral	ebi-a-GCST	GISIJ4	456154	rs9651903	0,012231	0,109876	0,911368
Lym	Mitral	ebi-a-GCST	GISIJ4	456154	rs9716164	0,014717	0,109875	0,893449
Lym	Mitral	ebi-a-GCST	GISIJ4	456154	rs978917	0,011399	0,109915	0,9174
Lym	Mitral	ebi-a-GCST	GISIJ4	456154	rs9826367	0,009427	0,11019	0,931824
Lym	Mitral	ebi-a-GCST	GISIJ4	456154	rs9834250	0,010261	0,109934	0,925638
Lym	Mitral	ebi-a-GCST	GISIJ4	456154	rs9863821	0,015828	0,109965	0,885552
Lym	Mitral	ebi-a-GCST	GISIJ4	456154	rs9864087	0,012148	0,109888	0,911976
Lym	Mitral	ebi-a-GCST	GISIJ4	456154	rs9886191	0,013505	0,109888	0,902187
Lym	Mitral	ebi-a-GCST	GISIJ4	456154	rs9920	0,015295	0,109954	0,889368
Lym	Mitral	ebi-a-GCST	GISIJ4	456154	All	0,010512	0,109833	0,923755
Mono	Mitral	ebi-a-GCST	wRHUFK	456073	rs1002774	0,005072	0,088398	0,954243
Mono	Mitral	ebi-a-GCST	wRHUFK	456073	rs1009954	0,000302	0,088295	0,997272
Mono	Mitral	ebi-a-GCST	wRHUFK	456073	rs1014701	-0,00022	0,088258	0,99798
Mono	Mitral	ebi-a-GCST	wRHUFK	456073	rs1015760	0,004724	0,08815	0,957262
Mono	Mitral	ebi-a-GCST	wRHUFK	456073	rs1016680	0,003952	0,088313	0,964305
Mono	Mitral	ebi-a-GCST	wRHUFK	456073	rs1019780	-0,00454	0,088295	0,959019
Mono	Mitral	ebi-a-GCST	wRHUFK	456073	rs1031091	0,002724	0,088192	0,975363
Mono	Mitral	ebi-a-GCST	wRHUFK	456073	rs1040390	0,000756	0,088315	0,993167
Mono	Mitral	ebi-a-GCST	wRHUFK	456073	rs1040975	-0,00085	0,088181	0,992294
Mono	Mitral	ebi-a-GCST	wRHUFK	456073	rs1042555	0,002545	0,088242	0,976994
Mono	Mitral	ebi-a-GCST	wRHUFK	456073	rs1046015	0,002865	0,08826	0,974103
Mono	Mitral	ebi-a-GCST	wRHUFK	456073	rs1047805	-0,00158	0,088128	0,985703
Mono	Mitral	ebi-a-GCST	wRHUFK	456073	rs1052484	0,003245	0,088156	0,970638

Mono	Mitral	ebi-a-GCST	wRHUFK	456073	rs1074473:	-0,00333	0,087915	0,969782
Mono	Mitral	ebi-a-GCST	wRHUFK	456073	rs1075848:	0,002714	0,088251	0,97547
Mono	Mitral	ebi-a-GCST	wRHUFK	456073	rs1077716	-0,0002	0,088303	0,998155
Mono	Mitral	ebi-a-GCST	wRHUFK	456073	rs1078020!	0,016523	0,088205	0,851404
Mono	Mitral	ebi-a-GCST	wRHUFK	456073	rs1079682:	0,000513	0,088316	0,995367
Mono	Mitral	ebi-a-GCST	wRHUFK	456073	rs1080468:	-0,00086	0,088233	0,992232
Mono	Mitral	ebi-a-GCST	wRHUFK	456073	rs1081419:	0,002536	0,088253	0,977079
Mono	Mitral	ebi-a-GCST	wRHUFK	456073	rs1082872!	-0,00755	0,088467	0,931957
Mono	Mitral	ebi-a-GCST	wRHUFK	456073	rs1083150	-0,0009	0,088176	0,991817
Mono	Mitral	ebi-a-GCST	wRHUFK	456073	rs1084902(	0,005665	0,088134	0,948749
Mono	Mitral	ebi-a-GCST	wRHUFK	456073	rs1084944:	-0,00777	0,08838	0,929958
Mono	Mitral	ebi-a-GCST	wRHUFK	456073	rs1088539(	0,00152	0,0883	0,986266
Mono	Mitral	ebi-a-GCST	wRHUFK	456073	rs1089234:	-0,00022	0,088293	0,997984
Mono	Mitral	ebi-a-GCST	wRHUFK	456073	rs1093547:	0,002017	0,088362	0,981787
Mono	Mitral	ebi-a-GCST	wRHUFK	456073	rs1094831	0,010555	0,087959	0,904481
Mono	Mitral	ebi-a-GCST	wRHUFK	456073	rs1098079	0,012784	0,091037	0,888324
Mono	Mitral	ebi-a-GCST	wRHUFK	456073	rs1105784:	0,001381	0,088299	0,987525
Mono	Mitral	ebi-a-GCST	wRHUFK	456073	rs1108610:	-0,00135	0,088391	0,987827
Mono	Mitral	ebi-a-GCST	wRHUFK	456073	rs1109832	0,00235	0,088257	0,978757
Mono	Mitral	ebi-a-GCST	wRHUFK	456073	rs1110488:	-0,00045	0,088268	0,995911
Mono	Mitral	ebi-a-GCST	wRHUFK	456073	rs1113061:	-0,00252	0,087973	0,977187
Mono	Mitral	ebi-a-GCST	wRHUFK	456073	rs1114565:	-0,00114	0,088148	0,989652
Mono	Mitral	ebi-a-GCST	wRHUFK	456073	rs1115578	-0,00205	0,088109	0,981405
Mono	Mitral	ebi-a-GCST	wRHUFK	456073	rs1118915	-0,00198	0,088552	0,982128
Mono	Mitral	ebi-a-GCST	wRHUFK	456073	rs1119014:	0,000821	0,088544	0,9926
Mono	Mitral	ebi-a-GCST	wRHUFK	456073	rs1123568!	0,003662	0,088213	0,96689
Mono	Mitral	ebi-a-GCST	wRHUFK	456073	rs1124210!	0,001392	0,088349	0,987429
Mono	Mitral	ebi-a-GCST	wRHUFK	456073	rs1124606!	0,005916	0,088147	0,946488
Mono	Mitral	ebi-a-GCST	wRHUFK	456073	rs1124712:	0,001412	0,088288	0,987236
Mono	Mitral	ebi-a-GCST	wRHUFK	456073	rs1124790:	0,006528	0,088129	0,94095
Mono	Mitral	ebi-a-GCST	wRHUFK	456073	rs1125214:	-0,00034	0,088249	0,996913
Mono	Mitral	ebi-a-GCST	wRHUFK	456073	rs1125554:	-0,0002	0,088318	0,998222
Mono	Mitral	ebi-a-GCST	wRHUFK	456073	rs1126529	-0,00019	0,088252	0,998245
Mono	Mitral	ebi-a-GCST	wRHUFK	456073	rs1130152:	-0,00456	0,08793	0,958627
Mono	Mitral	ebi-a-GCST	wRHUFK	456073	rs1130630:	0,000503	0,088298	0,99545
Mono	Mitral	ebi-a-GCST	wRHUFK	456073	rs1131550:	0,005434	0,087916	0,950715
Mono	Mitral	ebi-a-GCST	wRHUFK	456073	rs1132326:	0,000542	0,088315	0,995104
Mono	Mitral	ebi-a-GCST	wRHUFK	456073	rs1132350	0,004287	0,088213	0,961241
Mono	Mitral	ebi-a-GCST	wRHUFK	456073	rs1132920	0,001409	0,088293	0,987269
Mono	Mitral	ebi-a-GCST	wRHUFK	456073	rs1136810!	-0,00316	0,087918	0,971316
Mono	Mitral	ebi-a-GCST	wRHUFK	456073	rs1146933	0,002991	0,088188	0,97294
Mono	Mitral	ebi-a-GCST	wRHUFK	456073	rs1150802(	-2,26E-06	0,08827	0,99998
Mono	Mitral	ebi-a-GCST	wRHUFK	456073	rs1152028:	0,003969	0,088132	0,964075
Mono	Mitral	ebi-a-GCST	wRHUFK	456073	rs1153400:	0,008195	0,088238	0,926007
Mono	Mitral	ebi-a-GCST	wRHUFK	456073	rs1155715	-0,00242	0,088254	0,978103
Mono	Mitral	ebi-a-GCST	wRHUFK	456073	rs1159038(	0,005215	0,088235	0,952873
Mono	Mitral	ebi-a-GCST	wRHUFK	456073	rs1160232:	-0,00478	0,088484	0,956914
Mono	Mitral	ebi-a-GCST	wRHUFK	456073	rs1163141!	0,002657	0,088222	0,975978
Mono	Mitral	ebi-a-GCST	wRHUFK	456073	rs1164066	0,003741	0,088239	0,966184
Mono	Mitral	ebi-a-GCST	wRHUFK	456073	rs1164412!	0,003648	0,088259	0,967027

Mono	Mitral	ebi-a-GCST	wRHUFK	456073	rs1166199	0,002288	0,088264	0,979318
Mono	Mitral	ebi-a-GCST	wRHUFK	456073	rs1171334	0,001056	0,08834	0,990464
Mono	Mitral	ebi-a-GCST	wRHUFK	456073	rs1172362	-0,00119	0,08818	0,9892
Mono	Mitral	ebi-a-GCST	wRHUFK	456073	rs1174789	-0,00121	0,08815	0,989057
Mono	Mitral	ebi-a-GCST	wRHUFK	456073	rs1176680	0,003866	0,08823	0,965048
Mono	Mitral	ebi-a-GCST	wRHUFK	456073	rs1179203	0,002035	0,08828	0,98161
Mono	Mitral	ebi-a-GCST	wRHUFK	456073	rs1180838	-0,00205	0,088252	0,98151
Mono	Mitral	ebi-a-GCST	wRHUFK	456073	rs1184435	0,001502	0,088282	0,986426
Mono	Mitral	ebi-a-GCST	wRHUFK	456073	rs1185439	0,004113	0,088522	0,962945
Mono	Mitral	ebi-a-GCST	wRHUFK	456073	rs1185760	-0,00054	0,088313	0,995139
Mono	Mitral	ebi-a-GCST	wRHUFK	456073	rs1196588	0,001701	0,088274	0,984628
Mono	Mitral	ebi-a-GCST	wRHUFK	456073	rs1205564	-0,00124	0,088223	0,988779
Mono	Mitral	ebi-a-GCST	wRHUFK	456073	rs1211049	0,001134	0,08829	0,989754
Mono	Mitral	ebi-a-GCST	wRHUFK	456073	rs1211844	0,003003	0,08828	0,972862
Mono	Mitral	ebi-a-GCST	wRHUFK	456073	rs1214411	0,000949	0,088294	0,991423
Mono	Mitral	ebi-a-GCST	wRHUFK	456073	rs1215128	-0,00659	0,088139	0,940355
Mono	Mitral	ebi-a-GCST	wRHUFK	456073	rs1219823	0,000706	0,088287	0,993623
Mono	Mitral	ebi-a-GCST	wRHUFK	456073	rs1220769	-0,00041	0,088232	0,996313
Mono	Mitral	ebi-a-GCST	wRHUFK	456073	rs1230957	6,03E-05	0,088289	0,999455
Mono	Mitral	ebi-a-GCST	wRHUFK	456073	rs1233267	-0,00025	0,088258	0,997767
Mono	Mitral	ebi-a-GCST	wRHUFK	456073	rs1237651	0,001567	0,088466	0,985869
Mono	Mitral	ebi-a-GCST	wRHUFK	456073	rs1242830	-0,00132	0,08816	0,988079
Mono	Mitral	ebi-a-GCST	wRHUFK	456073	rs1248046	-0,00595	0,088191	0,946196
Mono	Mitral	ebi-a-GCST	wRHUFK	456073	rs1248073	-0,00196	0,088604	0,982352
Mono	Mitral	ebi-a-GCST	wRHUFK	456073	rs1252249	7,83E-05	0,088264	0,999292
Mono	Mitral	ebi-a-GCST	wRHUFK	456073	rs1253007	-0,00062	0,088236	0,994357
Mono	Mitral	ebi-a-GCST	wRHUFK	456073	rs1254290	-0,00304	0,088257	0,972521
Mono	Mitral	ebi-a-GCST	wRHUFK	456073	rs1269473	-0,00181	0,088063	0,983632
Mono	Mitral	ebi-a-GCST	wRHUFK	456073	rs1274242	0,002755	0,08827	0,975103
Mono	Mitral	ebi-a-GCST	wRHUFK	456073	rs1274743	0,000147	0,088278	0,99867
Mono	Mitral	ebi-a-GCST	wRHUFK	456073	rs1275613	0,001467	0,088283	0,986743
Mono	Mitral	ebi-a-GCST	wRHUFK	456073	rs1283945	0,002865	0,088178	0,974078
Mono	Mitral	ebi-a-GCST	wRHUFK	456073	rs1292460	0,001058	0,088302	0,990441
Mono	Mitral	ebi-a-GCST	wRHUFK	456073	rs1293448	0,00062	0,088296	0,994399
Mono	Mitral	ebi-a-GCST	wRHUFK	456073	rs1294135	-0,00059	0,088286	0,994685
Mono	Mitral	ebi-a-GCST	wRHUFK	456073	rs1296049	0,003265	0,088111	0,970441
Mono	Mitral	ebi-a-GCST	wRHUFK	456073	rs1300273	0,001284	0,088292	0,988399
Mono	Mitral	ebi-a-GCST	wRHUFK	456073	rs1302741	0,000778	0,088292	0,992973
Mono	Mitral	ebi-a-GCST	wRHUFK	456073	rs1303249	-0,00035	0,088237	0,99682
Mono	Mitral	ebi-a-GCST	wRHUFK	456073	rs1303278	-0,00156	0,08826	0,98592
Mono	Mitral	ebi-a-GCST	wRHUFK	456073	rs1307903	0,002629	0,088246	0,976237
Mono	Mitral	ebi-a-GCST	wRHUFK	456073	rs1316728	-0,00218	0,088008	0,980195
Mono	Mitral	ebi-a-GCST	wRHUFK	456073	rs1326746	0,001833	0,088328	0,983446
Mono	Mitral	ebi-a-GCST	wRHUFK	456073	rs1327122	0,003756	0,088384	0,966107
Mono	Mitral	ebi-a-GCST	wRHUFK	456073	rs1335929	-0,00186	0,08821	0,983176
Mono	Mitral	ebi-a-GCST	wRHUFK	456073	rs1338517	-0,00336	0,088301	0,969672
Mono	Mitral	ebi-a-GCST	wRHUFK	456073	rs1367153	-0,00025	0,08824	0,997782
Mono	Mitral	ebi-a-GCST	wRHUFK	456073	rs1375493	0,011615	0,090797	0,898211
Mono	Mitral	ebi-a-GCST	wRHUFK	456073	rs1380281	-0,00814	0,088421	0,926644
Mono	Mitral	ebi-a-GCST	wRHUFK	456073	rs139271	0,001395	0,088306	0,987399

Mono	Mitral	ebi-a-GCST	wRHUFK	456073	rs1409052[illegible]	0,000291	0,088297	0,997367
Mono	Mitral	ebi-a-GCST	wRHUFK	456073	rs1425553	0,001283	0,088289	0,988408
Mono	Mitral	ebi-a-GCST	wRHUFK	456073	rs1452099[illegible]	-0,00443	0,087942	0,959841
Mono	Mitral	ebi-a-GCST	wRHUFK	456073	rs1454474[illegible]	-0,00014	0,088277	0,998768
Mono	Mitral	ebi-a-GCST	wRHUFK	456073	rs1457180[illegible]	-0,00017	0,08828	0,998477
Mono	Mitral	ebi-a-GCST	wRHUFK	456073	rs1457705	-0,00178	0,088042	0,983913
Mono	Mitral	ebi-a-GCST	wRHUFK	456073	rs1466812[illegible]	0,000452	0,088287	0,995917
Mono	Mitral	ebi-a-GCST	wRHUFK	456073	rs1468488	0,004394	0,088349	0,960338
Mono	Mitral	ebi-a-GCST	wRHUFK	456073	rs1490077[illegible]	-0,00352	0,088617	0,968279
Mono	Mitral	ebi-a-GCST	wRHUFK	456073	rs1491105[illegible]	0,000277	0,088382	0,997501
Mono	Mitral	ebi-a-GCST	wRHUFK	456073	rs1499029[illegible]	-0,00127	0,088134	0,988492
Mono	Mitral	ebi-a-GCST	wRHUFK	456073	rs1504496[illegible]	0,002452	0,088474	0,977886
Mono	Mitral	ebi-a-GCST	wRHUFK	456073	rs1504948[illegible]	-0,00087	0,088301	0,992109
Mono	Mitral	ebi-a-GCST	wRHUFK	456073	rs1506494[illegible]	0,009636	0,089602	0,914359
Mono	Mitral	ebi-a-GCST	wRHUFK	456073	rs1562309	0,002171	0,088287	0,980382
Mono	Mitral	ebi-a-GCST	wRHUFK	456073	rs1581296	-0,00029	0,08825	0,997372
Mono	Mitral	ebi-a-GCST	wRHUFK	456073	rs1587222	0,002872	0,088221	0,974028
Mono	Mitral	ebi-a-GCST	wRHUFK	456073	rs1611236	-0,00424	0,088322	0,961691
Mono	Mitral	ebi-a-GCST	wRHUFK	456073	rs1683113[illegible]	-0,00089	0,088224	0,991982
Mono	Mitral	ebi-a-GCST	wRHUFK	456073	rs1687481	-7,66E-05	0,088262	0,999307
Mono	Mitral	ebi-a-GCST	wRHUFK	456073	rs1693960[illegible]	0,003218	0,088266	0,970913
Mono	Mitral	ebi-a-GCST	wRHUFK	456073	rs1697817[illegible]	0,007316	0,088154	0,933858
Mono	Mitral	ebi-a-GCST	wRHUFK	456073	rs1700159	0,006397	0,087973	0,94203
Mono	Mitral	ebi-a-GCST	wRHUFK	456073	rs1700589[illegible]	0,001486	0,088509	0,986607
Mono	Mitral	ebi-a-GCST	wRHUFK	456073	rs1701172[illegible]	-0,00064	0,088303	0,994226
Mono	Mitral	ebi-a-GCST	wRHUFK	456073	rs1708624[illegible]	-0,00067	0,088375	0,993919
Mono	Mitral	ebi-a-GCST	wRHUFK	456073	rs1715653[illegible]	0,003271	0,088266	0,970441
Mono	Mitral	ebi-a-GCST	wRHUFK	456073	rs1719675[illegible]	0,010952	0,088643	0,901666
Mono	Mitral	ebi-a-GCST	wRHUFK	456073	rs1738788[illegible]	0,004452	0,088245	0,959763
Mono	Mitral	ebi-a-GCST	wRHUFK	456073	rs1749824	0,002813	0,088212	0,974565
Mono	Mitral	ebi-a-GCST	wRHUFK	456073	rs1765620[illegible]	-0,00201	0,088644	0,981868
Mono	Mitral	ebi-a-GCST	wRHUFK	456073	rs1770043[illegible]	0,000562	0,088297	0,994923
Mono	Mitral	ebi-a-GCST	wRHUFK	456073	rs1772143	-0,00011	0,088321	0,998994
Mono	Mitral	ebi-a-GCST	wRHUFK	456073	rs17731	0,000482	0,088298	0,995644
Mono	Mitral	ebi-a-GCST	wRHUFK	456073	rs1775869[illegible]	-0,00218	0,088452	0,980354
Mono	Mitral	ebi-a-GCST	wRHUFK	456073	rs1781799	0,003088	0,08816	0,972057
Mono	Mitral	ebi-a-GCST	wRHUFK	456073	rs178405	0,00345	0,088155	0,968785
Mono	Mitral	ebi-a-GCST	wRHUFK	456073	rs1786042[illegible]	0,001363	0,088292	0,987683
Mono	Mitral	ebi-a-GCST	wRHUFK	456073	rs1800973	0,003073	0,088722	0,972371
Mono	Mitral	ebi-a-GCST	wRHUFK	456073	rs1822534	-0,00158	0,088387	0,985777
Mono	Mitral	ebi-a-GCST	wRHUFK	456073	rs1823405[illegible]	0,00108	0,088293	0,990241
Mono	Mitral	ebi-a-GCST	wRHUFK	456073	rs1840582[illegible]	0,001555	0,08828	0,98595
Mono	Mitral	ebi-a-GCST	wRHUFK	456073	rs1858802[illegible]	-0,00051	0,088232	0,995397
Mono	Mitral	ebi-a-GCST	wRHUFK	456073	rs1863651	-0,00012	0,08829	0,998944
Mono	Mitral	ebi-a-GCST	wRHUFK	456073	rs1869365	0,003591	0,088246	0,967542
Mono	Mitral	ebi-a-GCST	wRHUFK	456073	rs1881754[illegible]	0,002885	0,088329	0,973948
Mono	Mitral	ebi-a-GCST	wRHUFK	456073	rs1883933[illegible]	-0,00275	0,08809	0,975096
Mono	Mitral	ebi-a-GCST	wRHUFK	456073	rs1885376	0,003204	0,088172	0,971008
Mono	Mitral	ebi-a-GCST	wRHUFK	456073	rs1885525	0,002473	0,088253	0,977648
Mono	Mitral	ebi-a-GCST	wRHUFK	456073	rs1892548	0,009551	0,088729	0,91428

Mono	Mitral	ebi-a-GCST	wRHUFK	456073	rs191139	-0,0012	0,08843	0,98913
Mono	Mitral	ebi-a-GCST	wRHUFK	456073	rs1933295	-0,00202	0,088257	0,981717
Mono	Mitral	ebi-a-GCST	wRHUFK	456073	rs1954735	0,000381	0,088304	0,996553
Mono	Mitral	ebi-a-GCST	wRHUFK	456073	rs1967309	-0,00121	0,088308	0,989062
Mono	Mitral	ebi-a-GCST	wRHUFK	456073	rs1970364	0,005157	0,088105	0,953323
Mono	Mitral	ebi-a-GCST	wRHUFK	456073	rs1973325	-0,00064	0,088341	0,99423
Mono	Mitral	ebi-a-GCST	wRHUFK	456073	rs1992057	0,000268	0,088282	0,997575
Mono	Mitral	ebi-a-GCST	wRHUFK	456073	rs1997577	0,004318	0,088257	0,960975
Mono	Mitral	ebi-a-GCST	wRHUFK	456073	rs2006234[illegible]	-0,00018	0,088289	0,998365
Mono	Mitral	ebi-a-GCST	wRHUFK	456073	rs2007483	-0,0016	0,08831	0,985546
Mono	Mitral	ebi-a-GCST	wRHUFK	456073	rs2018092	0,003302	0,088144	0,97012
Mono	Mitral	ebi-a-GCST	wRHUFK	456073	rs2038700	6,78E-05	0,088502	0,999388
Mono	Mitral	ebi-a-GCST	wRHUFK	456073	rs2084312	0,006219	0,088304	0,943852
Mono	Mitral	ebi-a-GCST	wRHUFK	456073	rs2163952	-0,01186	0,089789	0,894939
Mono	Mitral	ebi-a-GCST	wRHUFK	456073	rs2183246	-0,00026	0,088265	0,997627
Mono	Mitral	ebi-a-GCST	wRHUFK	456073	rs2184697	0,00192	0,088284	0,982645
Mono	Mitral	ebi-a-GCST	wRHUFK	456073	rs2213290	0,001707	0,088455	0,984605
Mono	Mitral	ebi-a-GCST	wRHUFK	456073	rs2228467	0,011431	0,089084	0,897902
Mono	Mitral	ebi-a-GCST	wRHUFK	456073	rs2239630	-0,01066	0,088422	0,904021
Mono	Mitral	ebi-a-GCST	wRHUFK	456073	rs224101	0,002983	0,088302	0,973052
Mono	Mitral	ebi-a-GCST	wRHUFK	456073	rs2269486	-0,00037	0,08828	0,99663
Mono	Mitral	ebi-a-GCST	wRHUFK	456073	rs2273215	0,000421	0,088319	0,996196
Mono	Mitral	ebi-a-GCST	wRHUFK	456073	rs2274664	-0,00107	0,088242	0,990361
Mono	Mitral	ebi-a-GCST	wRHUFK	456073	rs2280244	0,003005	0,088172	0,972812
Mono	Mitral	ebi-a-GCST	wRHUFK	456073	rs2300598	0,000725	0,088311	0,993453
Mono	Mitral	ebi-a-GCST	wRHUFK	456073	rs2302774	0,001395	0,088356	0,987407
Mono	Mitral	ebi-a-GCST	wRHUFK	456073	rs230493	0,001223	0,088315	0,98895
Mono	Mitral	ebi-a-GCST	wRHUFK	456073	rs231988	0,004112	0,088203	0,962813
Mono	Mitral	ebi-a-GCST	wRHUFK	456073	rs2338021	0,003377	0,088289	0,969485
Mono	Mitral	ebi-a-GCST	wRHUFK	456073	rs2343551	0,006223	0,087985	0,943615
Mono	Mitral	ebi-a-GCST	wRHUFK	456073	rs2371109	0,002467	0,088219	0,977686
Mono	Mitral	ebi-a-GCST	wRHUFK	456073	rs238136	0,00129	0,088297	0,988345
Mono	Mitral	ebi-a-GCST	wRHUFK	456073	rs2393573	0,001835	0,088281	0,98342
Mono	Mitral	ebi-a-GCST	wRHUFK	456073	rs2433279	-0,00061	0,088266	0,994445
Mono	Mitral	ebi-a-GCST	wRHUFK	456073	rs244760	0,00213	0,088265	0,980745
Mono	Mitral	ebi-a-GCST	wRHUFK	456073	rs2491104	0,002599	0,088231	0,976502
Mono	Mitral	ebi-a-GCST	wRHUFK	456073	rs2504209	-7,80E-05	0,088284	0,999295
Mono	Mitral	ebi-a-GCST	wRHUFK	456073	rs2523552	0,011767	0,088675	0,894434
Mono	Mitral	ebi-a-GCST	wRHUFK	456073	rs2535403	0,003251	0,088236	0,97061
Mono	Mitral	ebi-a-GCST	wRHUFK	456073	rs2548257	0,002341	0,088255	0,978839
Mono	Mitral	ebi-a-GCST	wRHUFK	456073	rs2646421	0,000645	0,088369	0,994173
Mono	Mitral	ebi-a-GCST	wRHUFK	456073	rs2651369	0,002731	0,08824	0,975306
Mono	Mitral	ebi-a-GCST	wRHUFK	456073	rs2651780	0,007928	0,087931	0,928162
Mono	Mitral	ebi-a-GCST	wRHUFK	456073	rs2665405	-0,00801	0,088304	0,927697
Mono	Mitral	ebi-a-GCST	wRHUFK	456073	rs2711981	-0,00072	0,088312	0,993526
Mono	Mitral	ebi-a-GCST	wRHUFK	456073	rs2729450	-0,00046	0,088408	0,995813
Mono	Mitral	ebi-a-GCST	wRHUFK	456073	rs2734442	-0,0034	0,088301	0,969316
Mono	Mitral	ebi-a-GCST	wRHUFK	456073	rs2784250	0,002262	0,088253	0,979549
Mono	Mitral	ebi-a-GCST	wRHUFK	456073	rs2817441	0,003806	0,088256	0,965604
Mono	Mitral	ebi-a-GCST	wRHUFK	456073	rs2836220	0,00121	0,08829	0,989066

Mono	Mitral	ebi-a-GCST	wRHUFK	456073	rs284324	-0,00376	0,088156	0,965935
Mono	Mitral	ebi-a-GCST	wRHUFK	456073	rs2846573	0,00194	0,08826	0,982462
Mono	Mitral	ebi-a-GCST	wRHUFK	456073	rs2849828:	0,003479	0,088257	0,968561
Mono	Mitral	ebi-a-GCST	wRHUFK	456073	rs2864569:	0,000381	0,088281	0,996556
Mono	Mitral	ebi-a-GCST	wRHUFK	456073	rs2872270!	-0,00171	0,08832	0,984529
Mono	Mitral	ebi-a-GCST	wRHUFK	456073	rs2872353(	0,000694	0,088287	0,993727
Mono	Mitral	ebi-a-GCST	wRHUFK	456073	rs2885735	0,002084	0,088253	0,981162
Mono	Mitral	ebi-a-GCST	wRHUFK	456073	rs2899496	-0,00024	0,088258	0,997857
Mono	Mitral	ebi-a-GCST	wRHUFK	456073	rs290243	0,005251	0,087917	0,952369
Mono	Mitral	ebi-a-GCST	wRHUFK	456073	rs2927	0,005099	0,088096	0,953848
Mono	Mitral	ebi-a-GCST	wRHUFK	456073	rs2950835	0,00027	0,088287	0,997556
Mono	Mitral	ebi-a-GCST	wRHUFK	456073	rs2957873	-0,00216	0,088189	0,980457
Mono	Mitral	ebi-a-GCST	wRHUFK	456073	rs2959356	0,00283	0,088185	0,974399
Mono	Mitral	ebi-a-GCST	wRHUFK	456073	rs30102	0,001033	0,088296	0,990664
Mono	Mitral	ebi-a-GCST	wRHUFK	456073	rs3012415	-0,00386	0,08797	0,964981
Mono	Mitral	ebi-a-GCST	wRHUFK	456073	rs3014807	0,003738	0,088217	0,966199
Mono	Mitral	ebi-a-GCST	wRHUFK	456073	rs3027012	0,002998	0,088313	0,97292
Mono	Mitral	ebi-a-GCST	wRHUFK	456073	rs3111414	0,000487	0,088299	0,995598
Mono	Mitral	ebi-a-GCST	wRHUFK	456073	rs31243	0,001718	0,08829	0,984473
Mono	Mitral	ebi-a-GCST	wRHUFK	456073	rs3177609	0,002549	0,088285	0,976964
Mono	Mitral	ebi-a-GCST	wRHUFK	456073	rs3184504	0,001241	0,088611	0,988824
Mono	Mitral	ebi-a-GCST	wRHUFK	456073	rs329125	0,001815	0,088287	0,983595
Mono	Mitral	ebi-a-GCST	wRHUFK	456073	rs333947	0,003629	0,088343	0,967235
Mono	Mitral	ebi-a-GCST	wRHUFK	456073	rs3397862:	0,003451	0,088238	0,968802
Mono	Mitral	ebi-a-GCST	wRHUFK	456073	rs3408629:	0,005487	0,087918	0,950236
Mono	Mitral	ebi-a-GCST	wRHUFK	456073	rs3423635(	-0,00178	0,088324	0,983946
Mono	Mitral	ebi-a-GCST	wRHUFK	456073	rs3441762!	0,003135	0,088163	0,971637
Mono	Mitral	ebi-a-GCST	wRHUFK	456073	rs3450510	-0,00575	0,088244	0,948011
Mono	Mitral	ebi-a-GCST	wRHUFK	456073	rs3460012(	0,003937	0,088237	0,964409
Mono	Mitral	ebi-a-GCST	wRHUFK	456073	rs3474688!	-6,70E-05	0,088291	0,999394
Mono	Mitral	ebi-a-GCST	wRHUFK	456073	rs3488132!	5,41E-05	0,088251	0,999511
Mono	Mitral	ebi-a-GCST	wRHUFK	456073	rs3489093(	0,012179	0,089121	0,8913
Mono	Mitral	ebi-a-GCST	wRHUFK	456073	rs3493132:	0,001256	0,088288	0,98865
Mono	Mitral	ebi-a-GCST	wRHUFK	456073	rs3494760!	-0,00138	0,088138	0,987544
Mono	Mitral	ebi-a-GCST	wRHUFK	456073	rs3506849:	0,001499	0,088289	0,986457
Mono	Mitral	ebi-a-GCST	wRHUFK	456073	rs3510872	0,000371	0,088278	0,996649
Mono	Mitral	ebi-a-GCST	wRHUFK	456073	rs3535129:	0,001978	0,088275	0,982124
Mono	Mitral	ebi-a-GCST	wRHUFK	456073	rs354037	0,000392	0,088283	0,996453
Mono	Mitral	ebi-a-GCST	wRHUFK	456073	rs3597982:	-0,00364	0,088224	0,96711
Mono	Mitral	ebi-a-GCST	wRHUFK	456073	rs360017	0,009456	0,087958	0,914384
Mono	Mitral	ebi-a-GCST	wRHUFK	456073	rs3731211	0,004725	0,088298	0,957328
Mono	Mitral	ebi-a-GCST	wRHUFK	456073	rs3740395(	-0,00114	0,088388	0,989701
Mono	Mitral	ebi-a-GCST	wRHUFK	456073	rs3760871	0,000927	0,088296	0,991624
Mono	Mitral	ebi-a-GCST	wRHUFK	456073	rs3761986	-0,00145	0,088139	0,98685
Mono	Mitral	ebi-a-GCST	wRHUFK	456073	rs3787513	0,000143	0,088281	0,998705
Mono	Mitral	ebi-a-GCST	wRHUFK	456073	rs3789062	-0,00052	0,088292	0,995313
Mono	Mitral	ebi-a-GCST	wRHUFK	456073	rs3795503	0,002072	0,088307	0,981281
Mono	Mitral	ebi-a-GCST	wRHUFK	456073	rs3807583	-0,00317	0,087913	0,971246
Mono	Mitral	ebi-a-GCST	wRHUFK	456073	rs3808609	-0,00214	0,088173	0,980605
Mono	Mitral	ebi-a-GCST	wRHUFK	456073	rs3821710	0,00539	0,088194	0,951266

Mono	Mitral	ebi-a-GCST	wRHUFK	456073	rs3856364	0,001488	0,088288	0,986554
Mono	Mitral	ebi-a-GCST	wRHUFK	456073	rs3859570	-0,0032	0,088294	0,971088
Mono	Mitral	ebi-a-GCST	wRHUFK	456073	rs3931	0,002973	0,088313	0,973149
Mono	Mitral	ebi-a-GCST	wRHUFK	456073	rs412209	-0,00029	0,088286	0,997363
Mono	Mitral	ebi-a-GCST	wRHUFK	456073	rs41268099	0,001646	0,08832	0,985131
Mono	Mitral	ebi-a-GCST	wRHUFK	456073	rs41313381	-0,00794	0,08793	0,928017
Mono	Mitral	ebi-a-GCST	wRHUFK	456073	rs413141	0,010563	0,088022	0,904485
Mono	Mitral	ebi-a-GCST	wRHUFK	456073	rs41409548	0,005254	0,088338	0,952569
Mono	Mitral	ebi-a-GCST	wRHUFK	456073	rs4142441	0,003141	0,08831	0,971624
Mono	Mitral	ebi-a-GCST	wRHUFK	456073	rs41430449	0,00048	0,088303	0,995665
Mono	Mitral	ebi-a-GCST	wRHUFK	456073	rs42034	0,000207	0,088272	0,99813
Mono	Mitral	ebi-a-GCST	wRHUFK	456073	rs4269828	0,001448	0,08829	0,986912
Mono	Mitral	ebi-a-GCST	wRHUFK	456073	rs4365101	-0,00274	0,088033	0,975201
Mono	Mitral	ebi-a-GCST	wRHUFK	456073	rs4385425	-0,00433	0,088724	0,961087
Mono	Mitral	ebi-a-GCST	wRHUFK	456073	rs4411554	0,005268	0,087915	0,952223
Mono	Mitral	ebi-a-GCST	wRHUFK	456073	rs4432538	-0,00622	0,088186	0,943794
Mono	Mitral	ebi-a-GCST	wRHUFK	456073	rs445	-0,01099	0,088582	0,901228
Mono	Mitral	ebi-a-GCST	wRHUFK	456073	rs445611	0,00321	0,08816	0,970956
Mono	Mitral	ebi-a-GCST	wRHUFK	456073	rs4462758	-0,0007	0,088383	0,99369
Mono	Mitral	ebi-a-GCST	wRHUFK	456073	rs45577137	0,003581	0,088307	0,967657
Mono	Mitral	ebi-a-GCST	wRHUFK	456073	rs4566648	0,002949	0,088339	0,973366
Mono	Mitral	ebi-a-GCST	wRHUFK	456073	rs4626924	0,006251	0,088024	0,943382
Mono	Mitral	ebi-a-GCST	wRHUFK	456073	rs4669869	0,002738	0,088309	0,975269
Mono	Mitral	ebi-a-GCST	wRHUFK	456073	rs4710973	0,00317	0,088188	0,971329
Mono	Mitral	ebi-a-GCST	wRHUFK	456073	rs4721699	0,001774	0,088274	0,983965
Mono	Mitral	ebi-a-GCST	wRHUFK	456073	rs4724578	-0,00403	0,087915	0,963393
Mono	Mitral	ebi-a-GCST	wRHUFK	456073	rs47341	0,000496	0,088412	0,995521
Mono	Mitral	ebi-a-GCST	wRHUFK	456073	rs4737009	0,001057	0,0883	0,990448
Mono	Mitral	ebi-a-GCST	wRHUFK	456073	rs475616	0,003439	0,088209	0,968901
Mono	Mitral	ebi-a-GCST	wRHUFK	456073	rs4763944	0,00091	0,08829	0,991776
Mono	Mitral	ebi-a-GCST	wRHUFK	456073	rs4810832	0,002753	0,088215	0,975106
Mono	Mitral	ebi-a-GCST	wRHUFK	456073	rs4813619	0,001942	0,088302	0,982455
Mono	Mitral	ebi-a-GCST	wRHUFK	456073	rs4865956	0,001522	0,088316	0,98625
Mono	Mitral	ebi-a-GCST	wRHUFK	456073	rs4871844	0,001143	0,088305	0,989672
Mono	Mitral	ebi-a-GCST	wRHUFK	456073	rs4886615	0,004163	0,088195	0,962354
Mono	Mitral	ebi-a-GCST	wRHUFK	456073	rs4903311	0,002516	0,088223	0,977247
Mono	Mitral	ebi-a-GCST	wRHUFK	456073	rs4905043	0,002387	0,088302	0,978437
Mono	Mitral	ebi-a-GCST	wRHUFK	456073	rs4907230	-0,00164	0,088192	0,985152
Mono	Mitral	ebi-a-GCST	wRHUFK	456073	rs4952782	-0,00084	0,08827	0,992403
Mono	Mitral	ebi-a-GCST	wRHUFK	456073	rs4970966	-0,00846	0,088734	0,924066
Mono	Mitral	ebi-a-GCST	wRHUFK	456073	rs4980325	-0,00067	0,088249	0,993906
Mono	Mitral	ebi-a-GCST	wRHUFK	456073	rs4983387	-0,00199	0,088066	0,981936
Mono	Mitral	ebi-a-GCST	wRHUFK	456073	rs4987353	-0,0032	0,088186	0,971066
Mono	Mitral	ebi-a-GCST	wRHUFK	456073	rs505922	0,01028	0,088345	0,907362
Mono	Mitral	ebi-a-GCST	wRHUFK	456073	rs55666429	-0,00251	0,088132	0,977321
Mono	Mitral	ebi-a-GCST	wRHUFK	456073	rs5568423(	0,0047	0,087914	0,957368
Mono	Mitral	ebi-a-GCST	wRHUFK	456073	rs5600779	0,002991	0,088431	0,973016
Mono	Mitral	ebi-a-GCST	wRHUFK	456073	rs5605842(	0,00251	0,088226	0,977307
Mono	Mitral	ebi-a-GCST	wRHUFK	456073	rs5634489	-0,00488	0,088488	0,956052
Mono	Mitral	ebi-a-GCST	wRHUFK	456073	rs5679560	8,80E-05	0,08827	0,999205

Mono	Mitral	ebi-a-GCST	wRHUFK	456073	rs5692590!	0,001849	0,088273	0,983285
Mono	Mitral	ebi-a-GCST	wRHUFK	456073	rs571497	-0,00133	0,088286	0,988013
Mono	Mitral	ebi-a-GCST	wRHUFK	456073	rs5722139	0,000579	0,088288	0,994769
Mono	Mitral	ebi-a-GCST	wRHUFK	456073	rs573790	0,002829	0,08826	0,97443
Mono	Mitral	ebi-a-GCST	wRHUFK	456073	rs5770156	-0,00149	0,088398	0,986584
Mono	Mitral	ebi-a-GCST	wRHUFK	456073	rs5881415	0,002853	0,08827	0,974216
Mono	Mitral	ebi-a-GCST	wRHUFK	456073	rs5929568	3,95E-05	0,088292	0,999643
Mono	Mitral	ebi-a-GCST	wRHUFK	456073	rs5981082	0,002818	0,088311	0,974547
Mono	Mitral	ebi-a-GCST	wRHUFK	456073	rs6044081	0,000225	0,088272	0,997967
Mono	Mitral	ebi-a-GCST	wRHUFK	456073	rs6055629	0,00215	0,088262	0,980569
Mono	Mitral	ebi-a-GCST	wRHUFK	456073	rs6069534	0,000755	0,088304	0,993178
Mono	Mitral	ebi-a-GCST	wRHUFK	456073	rs6069990	0,002078	0,08828	0,98122
Mono	Mitral	ebi-a-GCST	wRHUFK	456073	rs6116564	0,007567	0,087944	0,931437
Mono	Mitral	ebi-a-GCST	wRHUFK	456073	rs6148472	-0,00091	0,088197	0,991729
Mono	Mitral	ebi-a-GCST	wRHUFK	456073	rs6173951	0,00132	0,088293	0,988073
Mono	Mitral	ebi-a-GCST	wRHUFK	456073	rs6201133	7,03E-05	0,088269	0,999364
Mono	Mitral	ebi-a-GCST	wRHUFK	456073	rs6201815	0,00266	0,088228	0,975947
Mono	Mitral	ebi-a-GCST	wRHUFK	456073	rs6205480	0,001853	0,088316	0,983262
Mono	Mitral	ebi-a-GCST	wRHUFK	456073	rs6211386	0,001957	0,088285	0,982314
Mono	Mitral	ebi-a-GCST	wRHUFK	456073	rs6212661	0,001517	0,088305	0,986298
Mono	Mitral	ebi-a-GCST	wRHUFK	456073	rs6226197	-0,00032	0,088262	0,99715
Mono	Mitral	ebi-a-GCST	wRHUFK	456073	rs6232971	0,000112	0,088331	0,998992
Mono	Mitral	ebi-a-GCST	wRHUFK	456073	rs6245442	-0,00356	0,088087	0,967738
Mono	Mitral	ebi-a-GCST	wRHUFK	456073	rs6247067	-0,00082	0,088238	0,992596
Mono	Mitral	ebi-a-GCST	wRHUFK	456073	rs6250113	0,001755	0,088309	0,984144
Mono	Mitral	ebi-a-GCST	wRHUFK	456073	rs6250239	0,000572	0,088294	0,994827
Mono	Mitral	ebi-a-GCST	wRHUFK	456073	rs6251334	0,000654	0,088287	0,994089
Mono	Mitral	ebi-a-GCST	wRHUFK	456073	rs628615	0,002825	0,08819	0,974445
Mono	Mitral	ebi-a-GCST	wRHUFK	456073	rs628977	0,003892	0,0881	0,964759
Mono	Mitral	ebi-a-GCST	wRHUFK	456073	rs6429432	0,009182	0,08855	0,917414
Mono	Mitral	ebi-a-GCST	wRHUFK	456073	rs6434817	0,000537	0,088284	0,995145
Mono	Mitral	ebi-a-GCST	wRHUFK	456073	rs6436124	0,005403	0,087976	0,951029
Mono	Mitral	ebi-a-GCST	wRHUFK	456073	rs644492	0,000408	0,088307	0,996314
Mono	Mitral	ebi-a-GCST	wRHUFK	456073	rs6465661	-0,00237	0,088165	0,978584
Mono	Mitral	ebi-a-GCST	wRHUFK	456073	rs649729	0,001559	0,088325	0,98592
Mono	Mitral	ebi-a-GCST	wRHUFK	456073	rs6512627	0,008992	0,088326	0,918915
Mono	Mitral	ebi-a-GCST	wRHUFK	456073	rs6540234	-0,00188	0,088674	0,983099
Mono	Mitral	ebi-a-GCST	wRHUFK	456073	rs6579771	0,006469	0,08829	0,941596
Mono	Mitral	ebi-a-GCST	wRHUFK	456073	rs6591578	0,001746	0,088337	0,984231
Mono	Mitral	ebi-a-GCST	wRHUFK	456073	rs662333	0,0012	0,088292	0,989154
Mono	Mitral	ebi-a-GCST	wRHUFK	456073	rs6650817	0,001146	0,088292	0,989641
Mono	Mitral	ebi-a-GCST	wRHUFK	456073	rs6664626	0,002693	0,088229	0,975651
Mono	Mitral	ebi-a-GCST	wRHUFK	456073	rs6687430	-0,00061	0,088232	0,994503
Mono	Mitral	ebi-a-GCST	wRHUFK	456073	rs6696074	0,002425	0,088298	0,978088
Mono	Mitral	ebi-a-GCST	wRHUFK	456073	rs6722495	-0,00217	0,08825	0,980374
Mono	Mitral	ebi-a-GCST	wRHUFK	456073	rs6733162	0,002608	0,088221	0,976417
Mono	Mitral	ebi-a-GCST	wRHUFK	456073	rs6736362	-0,00455	0,088124	0,958845
Mono	Mitral	ebi-a-GCST	wRHUFK	456073	rs6772164	-0,00184	0,088062	0,983291
Mono	Mitral	ebi-a-GCST	wRHUFK	456073	rs6796	-0,00097	0,088517	0,991277
Mono	Mitral	ebi-a-GCST	wRHUFK	456073	rs6856799	0,000641	0,088286	0,99421

Mono	Mitral	ebi-a-GCST	wRHUFK	456073	rs6869021	0,002615	0,088273	0,976369
Mono	Mitral	ebi-a-GCST	wRHUFK	456073	rs6883116	0,00342	0,088253	0,969092
Mono	Mitral	ebi-a-GCST	wRHUFK	456073	rs695113	0,009147	0,087952	0,917167
Mono	Mitral	ebi-a-GCST	wRHUFK	456073	rs701905	-0,00766	0,08796	0,930572
Mono	Mitral	ebi-a-GCST	wRHUFK	456073	rs7020171	-9,13E-06	0,088269	0,999917
Mono	Mitral	ebi-a-GCST	wRHUFK	456073	rs7030655	-0,00222	0,088222	0,979922
Mono	Mitral	ebi-a-GCST	wRHUFK	456073	rs707794	0,004566	0,088363	0,958788
Mono	Mitral	ebi-a-GCST	wRHUFK	456073	rs7095778	0,003888	0,088078	0,964791
Mono	Mitral	ebi-a-GCST	wRHUFK	456073	rs7111769	0,001229	0,088304	0,988895
Mono	Mitral	ebi-a-GCST	wRHUFK	456073	rs7120300	0,005499	0,087997	0,95017
Mono	Mitral	ebi-a-GCST	wRHUFK	456073	rs7136811	-0,00095	0,088211	0,991392
Mono	Mitral	ebi-a-GCST	wRHUFK	456073	rs7148215(	0,003348	0,088154	0,969705
Mono	Mitral	ebi-a-GCST	wRHUFK	456073	rs7151854	-0,0046	0,087932	0,958241
Mono	Mitral	ebi-a-GCST	wRHUFK	456073	rs7180079	-0,01074	0,088185	0,903068
Mono	Mitral	ebi-a-GCST	wRHUFK	456073	rs7180804	0,024951	0,088594	0,778226
Mono	Mitral	ebi-a-GCST	wRHUFK	456073	rs7185007	0,000221	0,088294	0,998
Mono	Mitral	ebi-a-GCST	wRHUFK	456073	rs718515	0,003573	0,088274	0,967716
Mono	Mitral	ebi-a-GCST	wRHUFK	456073	rs7196129	0,002851	0,088344	0,974254
Mono	Mitral	ebi-a-GCST	wRHUFK	456073	rs723585	0,005249	0,088086	0,952486
Mono	Mitral	ebi-a-GCST	wRHUFK	456073	rs7249692	-0,00072	0,088275	0,993516
Mono	Mitral	ebi-a-GCST	wRHUFK	456073	rs7267375	0,000182	0,088286	0,998353
Mono	Mitral	ebi-a-GCST	wRHUFK	456073	rs7267557	0,002065	0,088292	0,981344
Mono	Mitral	ebi-a-GCST	wRHUFK	456073	rs7272020(	0,006617	0,08793	0,940009
Mono	Mitral	ebi-a-GCST	wRHUFK	456073	rs7274379(	-0,00081	0,088243	0,992695
Mono	Mitral	ebi-a-GCST	wRHUFK	456073	rs7275926	-0,00173	0,088185	0,984362
Mono	Mitral	ebi-a-GCST	wRHUFK	456073	rs7279086	-0,00226	0,088222	0,979591
Mono	Mitral	ebi-a-GCST	wRHUFK	456073	rs7281407(	-0,00122	0,088242	0,989007
Mono	Mitral	ebi-a-GCST	wRHUFK	456073	rs7283630	0,007035	0,088261	0,936467
Mono	Mitral	ebi-a-GCST	wRHUFK	456073	rs7298704(	0,002158	0,088298	0,980503
Mono	Mitral	ebi-a-GCST	wRHUFK	456073	rs7303651	-0,00306	0,08877	0,972545
Mono	Mitral	ebi-a-GCST	wRHUFK	456073	rs7305859	-0,00151	0,088397	0,986383
Mono	Mitral	ebi-a-GCST	wRHUFK	456073	rs7308348	0,002332	0,08825	0,978915
Mono	Mitral	ebi-a-GCST	wRHUFK	456073	rs7312002	0,000229	0,088274	0,997933
Mono	Mitral	ebi-a-GCST	wRHUFK	456073	rs7320196	0,001443	0,088307	0,98696
Mono	Mitral	ebi-a-GCST	wRHUFK	456073	rs7320305	0,002422	0,088261	0,978104
Mono	Mitral	ebi-a-GCST	wRHUFK	456073	rs7321747(	0,002117	0,088375	0,980893
Mono	Mitral	ebi-a-GCST	wRHUFK	456073	rs7346759	0,002252	0,088279	0,97965
Mono	Mitral	ebi-a-GCST	wRHUFK	456073	rs7378428	-0,00387	0,087914	0,964896
Mono	Mitral	ebi-a-GCST	wRHUFK	456073	rs7380916(	-0,0019	0,088136	0,982844
Mono	Mitral	ebi-a-GCST	wRHUFK	456073	rs739241	0,00119	0,08832	0,989249
Mono	Mitral	ebi-a-GCST	wRHUFK	456073	rs7423380	-0,00033	0,088277	0,996999
Mono	Mitral	ebi-a-GCST	wRHUFK	456073	rs7425956(	-0,00034	0,088297	0,996909
Mono	Mitral	ebi-a-GCST	wRHUFK	456073	rs745570	0,000626	0,088301	0,99434
Mono	Mitral	ebi-a-GCST	wRHUFK	456073	rs745822	-0,00096	0,088258	0,991312
Mono	Mitral	ebi-a-GCST	wRHUFK	456073	rs7473500	0,002597	0,088254	0,976522
Mono	Mitral	ebi-a-GCST	wRHUFK	456073	rs7493103(	0,00233	0,088253	0,978934
Mono	Mitral	ebi-a-GCST	wRHUFK	456073	rs7512283	0,001979	0,088282	0,982117
Mono	Mitral	ebi-a-GCST	wRHUFK	456073	rs7516138	0,001052	0,088346	0,990502
Mono	Mitral	ebi-a-GCST	wRHUFK	456073	rs7522307	-0,00123	0,088234	0,988887
Mono	Mitral	ebi-a-GCST	wRHUFK	456073	rs7524046	0,003563	0,088157	0,967764

Mono	Mitral	ebi-a-GCST wRHUFK	456073	rs7542018	-0,00093	0,088232	0,991574
Mono	Mitral	ebi-a-GCST wRHUFK	456073	rs754388	-0,00203	0,088301	0,981647
Mono	Mitral	ebi-a-GCST wRHUFK	456073	rs7547562	0,001464	0,088291	0,986768
Mono	Mitral	ebi-a-GCST wRHUFK	456073	rs7572278	-0,00494	0,087942	0,955186
Mono	Mitral	ebi-a-GCST wRHUFK	456073	rs7574456	0,006104	0,088393	0,944945
Mono	Mitral	ebi-a-GCST wRHUFK	456073	rs7591224	0,001952	0,088305	0,982362
Mono	Mitral	ebi-a-GCST wRHUFK	456073	rs7593080	0,00145	0,088284	0,986896
Mono	Mitral	ebi-a-GCST wRHUFK	456073	rs761841	2,63E-05	0,088273	0,999762
Mono	Mitral	ebi-a-GCST wRHUFK	456073	rs7620353	0,000991	0,088292	0,991045
Mono	Mitral	ebi-a-GCST wRHUFK	456073	rs7633965	0,00308	0,088205	0,972147
Mono	Mitral	ebi-a-GCST wRHUFK	456073	rs7642810	-0,03954	0,089754	0,659543
Mono	Mitral	ebi-a-GCST wRHUFK	456073	rs7669246	0,001965	0,088314	0,982247
Mono	Mitral	ebi-a-GCST wRHUFK	456073	rs7687559	-0,00023	0,08827	0,997895
Mono	Mitral	ebi-a-GCST wRHUFK	456073	rs7692994	-0,00024	0,088267	0,997791
Mono	Mitral	ebi-a-GCST wRHUFK	456073	rs7773374	0,003828	0,088039	0,965316
Mono	Mitral	ebi-a-GCST wRHUFK	456073	rs7776054	0,00086	0,088441	0,992241
Mono	Mitral	ebi-a-GCST wRHUFK	456073	rs7786376	0,002374	0,088238	0,978537
Mono	Mitral	ebi-a-GCST wRHUFK	456073	rs7787179	0,001879	0,088308	0,983023
Mono	Mitral	ebi-a-GCST wRHUFK	456073	rs7792525	-0,00054	0,08827	0,995159
Mono	Mitral	ebi-a-GCST wRHUFK	456073	rs7803075	0,001557	0,088291	0,985926
Mono	Mitral	ebi-a-GCST wRHUFK	456073	rs7805715	-0,0003	0,08828	0,997309
Mono	Mitral	ebi-a-GCST wRHUFK	456073	rs7824937	0,001274	0,088316	0,988491
Mono	Mitral	ebi-a-GCST wRHUFK	456073	rs7826487	-0,0007	0,088361	0,993656
Mono	Mitral	ebi-a-GCST wRHUFK	456073	rs7908618	-0,00111	0,088172	0,989994
Mono	Mitral	ebi-a-GCST wRHUFK	456073	rs7919533	0,000166	0,08833	0,998505
Mono	Mitral	ebi-a-GCST wRHUFK	456073	rs7970950	-0,0013	0,088153	0,988276
Mono	Mitral	ebi-a-GCST wRHUFK	456073	rs7975680	0,000669	0,088292	0,993956
Mono	Mitral	ebi-a-GCST wRHUFK	456073	rs7983334	0,004492	0,088022	0,959296
Mono	Mitral	ebi-a-GCST wRHUFK	456073	rs798563	0,005633	0,08802	0,948977
Mono	Mitral	ebi-a-GCST wRHUFK	456073	rs7989433	0,008972	0,088138	0,91892
Mono	Mitral	ebi-a-GCST wRHUFK	456073	rs8002732	0,002623	0,088213	0,976278
Mono	Mitral	ebi-a-GCST wRHUFK	456073	rs8016326	0,004862	0,088693	0,95628
Mono	Mitral	ebi-a-GCST wRHUFK	456073	rs8054439	0,002418	0,088278	0,978147
Mono	Mitral	ebi-a-GCST wRHUFK	456073	rs8077619	0,002173	0,08827	0,980356
Mono	Mitral	ebi-a-GCST wRHUFK	456073	rs8207	0,002822	0,088214	0,974478
Mono	Mitral	ebi-a-GCST wRHUFK	456073	rs835636	-0,00092	0,08822	0,991709
Mono	Mitral	ebi-a-GCST wRHUFK	456073	rs837225	0,004274	0,088151	0,961328
Mono	Mitral	ebi-a-GCST wRHUFK	456073	rs869785	-0,00215	0,088181	0,980537
Mono	Mitral	ebi-a-GCST wRHUFK	456073	rs871134	-0,00393	0,08843	0,964541
Mono	Mitral	ebi-a-GCST wRHUFK	456073	rs907612	-0,00329	0,088364	0,970308
Mono	Mitral	ebi-a-GCST wRHUFK	456073	rs915125	0,002877	0,0884	0,974039
Mono	Mitral	ebi-a-GCST wRHUFK	456073	rs919217	-0,00054	0,088212	0,995071
Mono	Mitral	ebi-a-GCST wRHUFK	456073	rs9261401	0,003659	0,088382	0,966979
Mono	Mitral	ebi-a-GCST wRHUFK	456073	rs9264277	0,003823	0,088582	0,965572
Mono	Mitral	ebi-a-GCST wRHUFK	456073	rs929446	0,003743	0,088238	0,966168
Mono	Mitral	ebi-a-GCST wRHUFK	456073	rs932905	0,002712	0,088299	0,975498
Mono	Mitral	ebi-a-GCST wRHUFK	456073	rs9375150	0,000218	0,088318	0,998028
Mono	Mitral	ebi-a-GCST wRHUFK	456073	rs9379077	-0,00288	0,088254	0,973997
Mono	Mitral	ebi-a-GCST wRHUFK	456073	rs9390460	-0,00064	0,088228	0,994216
Mono	Mitral	ebi-a-GCST wRHUFK	456073	rs9410425	-0,00031	0,088386	0,997218

Mono	Mitral	ebi-a-GCST	wRHUFK	456073	rs9480737	-0,00169	0,088275	0,984705
Mono	Mitral	ebi-a-GCST	wRHUFK	456073	rs9532580	-0,0009	0,088345	0,991862
Mono	Mitral	ebi-a-GCST	wRHUFK	456073	rs954954	7,99E-07	0,08831	0,999993
Mono	Mitral	ebi-a-GCST	wRHUFK	456073	rs9555596	-0,00454	0,088132	0,958901
Mono	Mitral	ebi-a-GCST	wRHUFK	456073	rs9583493	0,003684	0,088088	0,966642
Mono	Mitral	ebi-a-GCST	wRHUFK	456073	rs9625746	-0,00074	0,088316	0,993307
Mono	Mitral	ebi-a-GCST	wRHUFK	456073	rs9637714	-0,00111	0,088233	0,989985
Mono	Mitral	ebi-a-GCST	wRHUFK	456073	rs9734613	0,000276	0,088275	0,997506
Mono	Mitral	ebi-a-GCST	wRHUFK	456073	rs9787298	0,000917	0,0883	0,991712
Mono	Mitral	ebi-a-GCST	wRHUFK	456073	rs9809116	-0,00166	0,08823	0,984966
Mono	Mitral	ebi-a-GCST	wRHUFK	456073	rs9846508	0,001231	0,08829	0,988876
Mono	Mitral	ebi-a-GCST	wRHUFK	456073	rs9855969	-0,0025	0,08806	0,977313
Mono	Mitral	ebi-a-GCST	wRHUFK	456073	rs9898876	0,003009	0,088232	0,972791
Mono	Mitral	ebi-a-GCST	wRHUFK	456073	rs9915112	-0,00501	0,088068	0,954675
Mono	Mitral	ebi-a-GCST	wRHUFK	456073	rs9943753	-0,00203	0,088114	0,98159
Mono	Mitral	ebi-a-GCST	wRHUFK	456073	rs9963693	-0,00191	0,088077	0,982668
Mono	Mitral	ebi-a-GCST	wRHUFK	456073	All	0,000992	0,088181	0,99102
Neutro	Mitral	ebi-a-GCST	Pt5VAH	456073	rs1002774	0,386012	0,119778	0,00127
Neutro	Mitral	ebi-a-GCST	Pt5VAH	456073	rs1004492	0,381806	0,11957	0,001407
Neutro	Mitral	ebi-a-GCST	Pt5VAH	456073	rs1004921	0,380646	0,119661	0,001468
Neutro	Mitral	ebi-a-GCST	Pt5VAH	456073	rs1016476	0,373162	0,119754	0,001833
Neutro	Mitral	ebi-a-GCST	Pt5VAH	456073	rs1018110	0,376114	0,119667	0,001672
Neutro	Mitral	ebi-a-GCST	Pt5VAH	456073	rs1025245	0,378062	0,119704	0,001587
Neutro	Mitral	ebi-a-GCST	Pt5VAH	456073	rs1025786	0,376953	0,119668	0,001633
Neutro	Mitral	ebi-a-GCST	Pt5VAH	456073	rs1043354	0,373554	0,119545	0,001779
Neutro	Mitral	ebi-a-GCST	Pt5VAH	456073	rs1049863	0,373465	0,119729	0,001813
Neutro	Mitral	ebi-a-GCST	Pt5VAH	456073	rs1076599	0,375123	0,11957	0,001705
Neutro	Mitral	ebi-a-GCST	Pt5VAH	456073	rs1078697	0,374659	0,119566	0,001727
Neutro	Mitral	ebi-a-GCST	Pt5VAH	456073	rs1080853	0,379621	0,119686	0,001515
Neutro	Mitral	ebi-a-GCST	Pt5VAH	456073	rs1082872	0,368341	0,120053	0,002154
Neutro	Mitral	ebi-a-GCST	Pt5VAH	456073	rs1084641	0,379402	0,119725	0,00153
Neutro	Mitral	ebi-a-GCST	Pt5VAH	456073	rs1087222	0,384166	0,119303	0,001282
Neutro	Mitral	ebi-a-GCST	Pt5VAH	456073	rs1088289	0,382262	0,120302	0,001485
Neutro	Mitral	ebi-a-GCST	Pt5VAH	456073	rs1091661	0,389842	0,11922	0,001076
Neutro	Mitral	ebi-a-GCST	Pt5VAH	456073	rs1093658	0,375081	0,119575	0,001708
Neutro	Mitral	ebi-a-GCST	Pt5VAH	456073	rs1094799	0,384902	0,119077	0,001228
Neutro	Mitral	ebi-a-GCST	Pt5VAH	456073	rs1106488	0,381789	0,119699	0,001425
Neutro	Mitral	ebi-a-GCST	Pt5VAH	456073	rs1108576	0,383161	0,119429	0,001335
Neutro	Mitral	ebi-a-GCST	Pt5VAH	456073	rs1114598	0,377257	0,119974	0,001664
Neutro	Mitral	ebi-a-GCST	Pt5VAH	456073	rs1134924	0,378933	0,119747	0,001554
Neutro	Mitral	ebi-a-GCST	Pt5VAH	456073	rs1136864	0,383326	0,119421	0,001328
Neutro	Mitral	ebi-a-GCST	Pt5VAH	456073	rs1140506	0,376951	0,119938	0,001673
Neutro	Mitral	ebi-a-GCST	Pt5VAH	456073	rs1144700	0,383766	0,119779	0,001356
Neutro	Mitral	ebi-a-GCST	Pt5VAH	456073	rs1154903	0,373737	0,119459	0,001757
Neutro	Mitral	ebi-a-GCST	Pt5VAH	456073	rs1155692	0,379218	0,119729	0,001539
Neutro	Mitral	ebi-a-GCST	Pt5VAH	456073	rs1161148	0,37969	0,119672	0,00151
Neutro	Mitral	ebi-a-GCST	Pt5VAH	456073	rs1161164	0,38736	0,119353	0,001172
Neutro	Mitral	ebi-a-GCST	Pt5VAH	456073	rs1164474	0,381189	0,119607	0,001438
Neutro	Mitral	ebi-a-GCST	Pt5VAH	456073	rs1164774	0,378487	0,119753	0,001575
Neutro	Mitral	ebi-a-GCST	Pt5VAH	456073	rs1164866	0,377557	0,11971	0,001611

Neutro	Mitral	ebi-a-GCST	Pt5VAH	456073	rs1165010(	0,36879	0,119805	0,002082
Neutro	Mitral	ebi-a-GCST	Pt5VAH	456073	rs1165382(	0,376355	0,119691	0,001664
Neutro	Mitral	ebi-a-GCST	Pt5VAH	456073	rs1167309:	0,380314	0,120256	0,001564
Neutro	Mitral	ebi-a-GCST	Pt5VAH	456073	rs1172362:	0,371774	0,119739	0,001904
Neutro	Mitral	ebi-a-GCST	Pt5VAH	456073	rs1172570	0,337879	0,120916	0,005201
Neutro	Mitral	ebi-a-GCST	Pt5VAH	456073	rs1173566:	0,377062	0,11975	0,00164
Neutro	Mitral	ebi-a-GCST	Pt5VAH	456073	rs1174177!	0,371344	0,119735	0,001926
Neutro	Mitral	ebi-a-GCST	Pt5VAH	456073	rs1174466:	0,379265	0,119704	0,001533
Neutro	Mitral	ebi-a-GCST	Pt5VAH	456073	rs1178159:	0,363617	0,119099	0,002265
Neutro	Mitral	ebi-a-GCST	Pt5VAH	456073	rs1180174!	0,380725	0,119648	0,001462
Neutro	Mitral	ebi-a-GCST	Pt5VAH	456073	rs1188140:	0,379103	0,119689	0,001538
Neutro	Mitral	ebi-a-GCST	Pt5VAH	456073	rs1212123(	0,376077	0,119682	0,001676
Neutro	Mitral	ebi-a-GCST	Pt5VAH	456073	rs1213967(	0,379343	0,119724	0,001532
Neutro	Mitral	ebi-a-GCST	Pt5VAH	456073	rs1214190:	0,374157	0,119684	0,001771
Neutro	Mitral	ebi-a-GCST	Pt5VAH	456073	rs1242301	0,391108	0,118477	0,000963
Neutro	Mitral	ebi-a-GCST	Pt5VAH	456073	rs1245035	0,378522	0,11974	0,001571
Neutro	Mitral	ebi-a-GCST	Pt5VAH	456073	rs1248765	0,380113	0,119704	0,001496
Neutro	Mitral	ebi-a-GCST	Pt5VAH	456073	rs1250567	0,38217	0,11951	0,001385
Neutro	Mitral	ebi-a-GCST	Pt5VAH	456073	rs1253334(	0,380818	0,119743	0,001471
Neutro	Mitral	ebi-a-GCST	Pt5VAH	456073	rs1255061:	0,390379	0,119356	0,001073
Neutro	Mitral	ebi-a-GCST	Pt5VAH	456073	rs1258871	0,377807	0,119716	0,0016
Neutro	Mitral	ebi-a-GCST	Pt5VAH	456073	rs1259966(	0,380274	0,11965	0,001482
Neutro	Mitral	ebi-a-GCST	Pt5VAH	456073	rs1260326	0,386083	0,119916	0,001284
Neutro	Mitral	ebi-a-GCST	Pt5VAH	456073	rs1265894	0,381926	0,119531	0,001397
Neutro	Mitral	ebi-a-GCST	Pt5VAH	456073	rs1268407(	0,376966	0,119678	0,001634
Neutro	Mitral	ebi-a-GCST	Pt5VAH	456073	rs1269256	0,384648	0,119823	0,001327
Neutro	Mitral	ebi-a-GCST	Pt5VAH	456073	rs1273784:	0,381086	0,119616	0,001443
Neutro	Mitral	ebi-a-GCST	Pt5VAH	456073	rs1288474:	0,384411	0,119654	0,001315
Neutro	Mitral	ebi-a-GCST	Pt5VAH	456073	rs1293652!	0,387656	0,120002	0,001236
Neutro	Mitral	ebi-a-GCST	Pt5VAH	456073	rs1296787	0,374923	0,119552	0,001712
Neutro	Mitral	ebi-a-GCST	Pt5VAH	456073	rs1301326	0,377312	0,119702	0,001621
Neutro	Mitral	ebi-a-GCST	Pt5VAH	456073	rs1312117!	0,371822	0,119103	0,001797
Neutro	Mitral	ebi-a-GCST	Pt5VAH	456073	rs1318907:	0,379211	0,11969	0,001533
Neutro	Mitral	ebi-a-GCST	Pt5VAH	456073	rs1319003(	0,374013	0,119502	0,001749
Neutro	Mitral	ebi-a-GCST	Pt5VAH	456073	rs1323126:	0,362874	0,118451	0,002188
Neutro	Mitral	ebi-a-GCST	Pt5VAH	456073	rs1346755	0,379955	0,119659	0,001497
Neutro	Mitral	ebi-a-GCST	Pt5VAH	456073	rs1362623	0,375616	0,11966	0,001695
Neutro	Mitral	ebi-a-GCST	Pt5VAH	456073	rs1382846:	0,383339	0,119896	0,001387
Neutro	Mitral	ebi-a-GCST	Pt5VAH	456073	rs139402	0,380831	0,119646	0,001458
Neutro	Mitral	ebi-a-GCST	Pt5VAH	456073	rs1404415:	0,379914	0,119696	0,001504
Neutro	Mitral	ebi-a-GCST	Pt5VAH	456073	rs1412445	0,364489	0,118809	0,002156
Neutro	Mitral	ebi-a-GCST	Pt5VAH	456073	rs1415431	0,379058	0,119695	0,001541
Neutro	Mitral	ebi-a-GCST	Pt5VAH	456073	rs1430037:	0,379066	0,119706	0,001542
Neutro	Mitral	ebi-a-GCST	Pt5VAH	456073	rs1436994	0,380795	0,11979	0,001479
Neutro	Mitral	ebi-a-GCST	Pt5VAH	456073	rs14408	0,360473	0,120171	0,002703
Neutro	Mitral	ebi-a-GCST	Pt5VAH	456073	rs1447211!	0,383248	0,119399	0,001328
Neutro	Mitral	ebi-a-GCST	Pt5VAH	456073	rs1452099	0,371033	0,11912	0,001841
Neutro	Mitral	ebi-a-GCST	Pt5VAH	456073	rs1456896	0,387595	0,119502	0,001181
Neutro	Mitral	ebi-a-GCST	Pt5VAH	456073	rs1468102	0,368113	0,118932	0,001967
Neutro	Mitral	ebi-a-GCST	Pt5VAH	456073	rs1490077(	0,382768	0,119723	0,001388

Neutro	Mitral	ebi-a-GCST	Pt5VAH	456073	rs1506494[illegible]	0,380402	0,119626	0,001473
Neutro	Mitral	ebi-a-GCST	Pt5VAH	456073	rs1508617[illegible]	0,377658	0,119693	0,001604
Neutro	Mitral	ebi-a-GCST	Pt5VAH	456073	rs1521134	0,381804	0,119554	0,001405
Neutro	Mitral	ebi-a-GCST	Pt5VAH	456073	rs1549669	0,381537	0,119553	0,001416
Neutro	Mitral	ebi-a-GCST	Pt5VAH	456073	rs1611236	0,38427	0,119535	0,001306
Neutro	Mitral	ebi-a-GCST	Pt5VAH	456073	rs1684334[illegible]	0,375969	0,119629	0,001673
Neutro	Mitral	ebi-a-GCST	Pt5VAH	456073	rs1685007[illegible]	0,374844	0,120551	0,001875
Neutro	Mitral	ebi-a-GCST	Pt5VAH	456073	rs1695864[illegible]	0,384651	0,119285	0,001261
Neutro	Mitral	ebi-a-GCST	Pt5VAH	456073	rs1703039[illegible]	0,373626	0,1195	0,001768
Neutro	Mitral	ebi-a-GCST	Pt5VAH	456073	rs1704143[illegible]	0,378541	0,119704	0,001565
Neutro	Mitral	ebi-a-GCST	Pt5VAH	456073	rs1704993[illegible]	0,380842	0,119663	0,00146
Neutro	Mitral	ebi-a-GCST	Pt5VAH	456073	rs1715429	0,377212	0,119763	0,001635
Neutro	Mitral	ebi-a-GCST	Pt5VAH	456073	rs172933	0,378228	0,119698	0,001578
Neutro	Mitral	ebi-a-GCST	Pt5VAH	456073	rs1738668[illegible]	0,38584	0,119425	0,001234
Neutro	Mitral	ebi-a-GCST	Pt5VAH	456073	rs174544	0,378171	0,11986	0,001604
Neutro	Mitral	ebi-a-GCST	Pt5VAH	456073	rs1752142[illegible]	0,378712	0,119771	0,001567
Neutro	Mitral	ebi-a-GCST	Pt5VAH	456073	rs1778507[illegible]	0,378274	0,119699	0,001576
Neutro	Mitral	ebi-a-GCST	Pt5VAH	456073	rs1783921	0,374572	0,119576	0,001733
Neutro	Mitral	ebi-a-GCST	Pt5VAH	456073	rs1791807	0,370539	0,119343	0,001904
Neutro	Mitral	ebi-a-GCST	Pt5VAH	456073	rs1800961	0,369104	0,119316	0,001978
Neutro	Mitral	ebi-a-GCST	Pt5VAH	456073	rs1855829	0,379275	0,119735	0,001537
Neutro	Mitral	ebi-a-GCST	Pt5VAH	456073	rs1886534[illegible]	0,377575	0,119743	0,001615
Neutro	Mitral	ebi-a-GCST	Pt5VAH	456073	rs1888190	0,381598	0,119569	0,001416
Neutro	Mitral	ebi-a-GCST	Pt5VAH	456073	rs1918328[illegible]	0,377685	0,119765	0,001613
Neutro	Mitral	ebi-a-GCST	Pt5VAH	456073	rs1981627	0,376921	0,119693	0,001638
Neutro	Mitral	ebi-a-GCST	Pt5VAH	456073	rs1985157	0,389311	0,119525	0,001125
Neutro	Mitral	ebi-a-GCST	Pt5VAH	456073	rs2006397[illegible]	0,379474	0,119691	0,001522
Neutro	Mitral	ebi-a-GCST	Pt5VAH	456073	rs2015210	0,380251	0,119744	0,001496
Neutro	Mitral	ebi-a-GCST	Pt5VAH	456073	rs2028150	0,382581	0,119815	0,001408
Neutro	Mitral	ebi-a-GCST	Pt5VAH	456073	rs2038700	0,379494	0,12004	0,00157
Neutro	Mitral	ebi-a-GCST	Pt5VAH	456073	rs2082382	0,37165	0,119899	0,001937
Neutro	Mitral	ebi-a-GCST	Pt5VAH	456073	rs211432	0,379061	0,1197	0,001542
Neutro	Mitral	ebi-a-GCST	Pt5VAH	456073	rs2116942	0,371119	0,119555	0,001908
Neutro	Mitral	ebi-a-GCST	Pt5VAH	456073	rs212409	0,382056	0,11975	0,001421
Neutro	Mitral	ebi-a-GCST	Pt5VAH	456073	rs2158799	0,397139	0,120295	0,000962
Neutro	Mitral	ebi-a-GCST	Pt5VAH	456073	rs2170640	0,382474	0,119461	0,001366
Neutro	Mitral	ebi-a-GCST	Pt5VAH	456073	rs218264	0,375212	0,119927	0,001756
Neutro	Mitral	ebi-a-GCST	Pt5VAH	456073	rs2205190	0,376219	0,119693	0,001671
Neutro	Mitral	ebi-a-GCST	Pt5VAH	456073	rs2227322	0,376634	0,125135	0,002614
Neutro	Mitral	ebi-a-GCST	Pt5VAH	456073	rs2240775	0,386103	0,11919	0,001198
Neutro	Mitral	ebi-a-GCST	Pt5VAH	456073	rs2260766	0,383432	0,119708	0,00136
Neutro	Mitral	ebi-a-GCST	Pt5VAH	456073	rs2288592	0,3767	0,119661	0,001644
Neutro	Mitral	ebi-a-GCST	Pt5VAH	456073	rs2290846	0,377029	0,119734	0,001639
Neutro	Mitral	ebi-a-GCST	Pt5VAH	456073	rs2352480	0,381107	0,119591	0,001439
Neutro	Mitral	ebi-a-GCST	Pt5VAH	456073	rs238914	0,376201	0,119916	0,001706
Neutro	Mitral	ebi-a-GCST	Pt5VAH	456073	rs2393969	0,391232	0,11955	0,001066
Neutro	Mitral	ebi-a-GCST	Pt5VAH	456073	rs2412771	0,375898	0,119668	0,001683
Neutro	Mitral	ebi-a-GCST	Pt5VAH	456073	rs2421200	0,392572	0,118739	0,000946
Neutro	Mitral	ebi-a-GCST	Pt5VAH	456073	rs248229	0,388007	0,118658	0,001076
Neutro	Mitral	ebi-a-GCST	Pt5VAH	456073	rs2494748	0,37725	0,119692	0,001622

Neutro	Mitral	ebi-a-GCST	Pt5VAH	456073	rs2504235	0,373485	0,119785	0,001821
Neutro	Mitral	ebi-a-GCST	Pt5VAH	456073	rs2519093	0,373203	0,119959	0,001864
Neutro	Mitral	ebi-a-GCST	Pt5VAH	456073	rs2561758	0,401404	0,118666	0,000718
Neutro	Mitral	ebi-a-GCST	Pt5VAH	456073	rs257063	0,374441	0,119512	0,00173
Neutro	Mitral	ebi-a-GCST	Pt5VAH	456073	rs263067	0,377422	0,119727	0,00162
Neutro	Mitral	ebi-a-GCST	Pt5VAH	456073	rs2632372	0,382489	0,119728	0,0014
Neutro	Mitral	ebi-a-GCST	Pt5VAH	456073	rs2679741	0,374472	0,1197	0,001758
Neutro	Mitral	ebi-a-GCST	Pt5VAH	456073	rs2710804	0,374943	0,119746	0,001741
Neutro	Mitral	ebi-a-GCST	Pt5VAH	456073	rs273582	0,382869	0,119434	0,001347
Neutro	Mitral	ebi-a-GCST	Pt5VAH	456073	rs2736429	0,371168	0,119829	0,001952
Neutro	Mitral	ebi-a-GCST	Pt5VAH	456073	rs2807742	0,374515	0,119901	0,001787
Neutro	Mitral	ebi-a-GCST	Pt5VAH	456073	rs284317	0,372863	0,119398	0,001791
Neutro	Mitral	ebi-a-GCST	Pt5VAH	456073	rs284440	0,389203	0,118734	0,001046
Neutro	Mitral	ebi-a-GCST	Pt5VAH	456073	rs2853075(	0,378119	0,119847	0,001605
Neutro	Mitral	ebi-a-GCST	Pt5VAH	456073	rs2857176!	0,38048	0,119731	0,001484
Neutro	Mitral	ebi-a-GCST	Pt5VAH	456073	rs2857382(	0,377031	0,119704	0,001634
Neutro	Mitral	ebi-a-GCST	Pt5VAH	456073	rs2858814:	0,379758	0,119677	0,001508
Neutro	Mitral	ebi-a-GCST	Pt5VAH	456073	rs2862180!	0,3848	0,11961	0,001295
Neutro	Mitral	ebi-a-GCST	Pt5VAH	456073	rs2867816	0,379472	0,119777	0,001534
Neutro	Mitral	ebi-a-GCST	Pt5VAH	456073	rs2977806	0,380734	0,119647	0,001462
Neutro	Mitral	ebi-a-GCST	Pt5VAH	456073	rs2979489	0,375216	0,119708	0,001722
Neutro	Mitral	ebi-a-GCST	Pt5VAH	456073	rs2980888	0,383374	0,119702	0,001361
Neutro	Mitral	ebi-a-GCST	Pt5VAH	456073	rs2992836	0,371753	0,11959	0,00188
Neutro	Mitral	ebi-a-GCST	Pt5VAH	456073	rs301817	0,376882	0,119687	0,001639
Neutro	Mitral	ebi-a-GCST	Pt5VAH	456073	rs303753	0,373963	0,119725	0,001787
Neutro	Mitral	ebi-a-GCST	Pt5VAH	456073	rs305082	0,391697	0,119448	0,001041
Neutro	Mitral	ebi-a-GCST	Pt5VAH	456073	rs3094548	0,38297	0,119763	0,001385
Neutro	Mitral	ebi-a-GCST	Pt5VAH	456073	rs3184504	0,380838	0,11998	0,001503
Neutro	Mitral	ebi-a-GCST	Pt5VAH	456073	rs342242	0,375701	0,119687	0,001695
Neutro	Mitral	ebi-a-GCST	Pt5VAH	456073	rs3429378!	0,379203	0,120326	0,001624
Neutro	Mitral	ebi-a-GCST	Pt5VAH	456073	rs3446237(	0,375577	0,119595	0,001687
Neutro	Mitral	ebi-a-GCST	Pt5VAH	456073	rs3459908:	0,382393	0,120259	0,001474
Neutro	Mitral	ebi-a-GCST	Pt5VAH	456073	rs3463454!	0,376587	0,119894	0,001684
Neutro	Mitral	ebi-a-GCST	Pt5VAH	456073	rs3483400	0,383177	0,119376	0,001328
Neutro	Mitral	ebi-a-GCST	Pt5VAH	456073	rs3499033(	0,374956	0,119517	0,001705
Neutro	Mitral	ebi-a-GCST	Pt5VAH	456073	rs354703	0,378191	0,119721	0,001583
Neutro	Mitral	ebi-a-GCST	Pt5VAH	456073	rs3555119:	0,390035	0,118686	0,001015
Neutro	Mitral	ebi-a-GCST	Pt5VAH	456073	rs3573424:	0,370443	0,119628	0,001957
Neutro	Mitral	ebi-a-GCST	Pt5VAH	456073	rs3578917!	0,371692	0,119271	0,001831
Neutro	Mitral	ebi-a-GCST	Pt5VAH	456073	rs3589858!	0,372718	0,11933	0,001788
Neutro	Mitral	ebi-a-GCST	Pt5VAH	456073	rs3592965!	0,378434	0,11972	0,001572
Neutro	Mitral	ebi-a-GCST	Pt5VAH	456073	rs3599017(	0,374613	0,119526	0,001723
Neutro	Mitral	ebi-a-GCST	Pt5VAH	456073	rs3602651	0,379182	0,119761	0,001545
Neutro	Mitral	ebi-a-GCST	Pt5VAH	456073	rs3605189!	0,383967	0,119308	0,00129
Neutro	Mitral	ebi-a-GCST	Pt5VAH	456073	rs3610149:	0,375378	0,119741	0,001719
Neutro	Mitral	ebi-a-GCST	Pt5VAH	456073	rs3731236:	0,370574	0,119035	0,001851
Neutro	Mitral	ebi-a-GCST	Pt5VAH	456073	rs3740049	0,37841	0,119726	0,001574
Neutro	Mitral	ebi-a-GCST	Pt5VAH	456073	rs3747869	0,379244	0,119778	0,001544
Neutro	Mitral	ebi-a-GCST	Pt5VAH	456073	rs3754224	0,37832	0,119728	0,001579
Neutro	Mitral	ebi-a-GCST	Pt5VAH	456073	rs3762297	0,37555	0,119752	0,001712

Neutro	Mitral	ebi-a-GCST	Pt5VAH	456073	rs3777755	0,372812	0,119375	0,00179
Neutro	Mitral	ebi-a-GCST	Pt5VAH	456073	rs3781454	0,372448	0,119683	0,001858
Neutro	Mitral	ebi-a-GCST	Pt5VAH	456073	rs3793537	0,38038	0,119642	0,001476
Neutro	Mitral	ebi-a-GCST	Pt5VAH	456073	rs3856364	0,379982	0,119704	0,001502
Neutro	Mitral	ebi-a-GCST	Pt5VAH	456073	rs385893	0,390834	0,119469	0,00107
Neutro	Mitral	ebi-a-GCST	Pt5VAH	456073	rs386243	0,377497	0,119826	0,001631
Neutro	Mitral	ebi-a-GCST	Pt5VAH	456073	rs3887943	0,378034	0,119713	0,001589
Neutro	Mitral	ebi-a-GCST	Pt5VAH	456073	rs398474	0,380544	0,119638	0,001469
Neutro	Mitral	ebi-a-GCST	Pt5VAH	456073	rs4127253(	0,380728	0,119655	0,001463
Neutro	Mitral	ebi-a-GCST	Pt5VAH	456073	rs4131338:	0,357217	0,117777	0,002422
Neutro	Mitral	ebi-a-GCST	Pt5VAH	456073	rs4145952	0,377402	0,119706	0,001617
Neutro	Mitral	ebi-a-GCST	Pt5VAH	456073	rs416633	0,375055	0,119561	0,001707
Neutro	Mitral	ebi-a-GCST	Pt5VAH	456073	rs42033	0,37812	0,120044	0,001634
Neutro	Mitral	ebi-a-GCST	Pt5VAH	456073	rs4347951	0,377058	0,11968	0,00163
Neutro	Mitral	ebi-a-GCST	Pt5VAH	456073	rs439749	0,371265	0,119047	0,001817
Neutro	Mitral	ebi-a-GCST	Pt5VAH	456073	rs445	0,36419	0,120708	0,002552
Neutro	Mitral	ebi-a-GCST	Pt5VAH	456073	rs4468717	0,37646	0,119695	0,00166
Neutro	Mitral	ebi-a-GCST	Pt5VAH	456073	rs4535497	0,379302	0,119717	0,001533
Neutro	Mitral	ebi-a-GCST	Pt5VAH	456073	rs4556473	0,374451	0,119525	0,001731
Neutro	Mitral	ebi-a-GCST	Pt5VAH	456073	rs4557713	0,376163	0,119653	0,001668
Neutro	Mitral	ebi-a-GCST	Pt5VAH	456073	rs4599108	0,375001	0,119725	0,001735
Neutro	Mitral	ebi-a-GCST	Pt5VAH	456073	rs4623401	0,381766	0,11967	0,001422
Neutro	Mitral	ebi-a-GCST	Pt5VAH	456073	rs465724	0,382432	0,119678	0,001396
Neutro	Mitral	ebi-a-GCST	Pt5VAH	456073	rs4682867	0,374576	0,119748	0,00176
Neutro	Mitral	ebi-a-GCST	Pt5VAH	456073	rs4703890	0,386534	0,119774	0,00125
Neutro	Mitral	ebi-a-GCST	Pt5VAH	456073	rs4724795	0,374751	0,11955	0,00172
Neutro	Mitral	ebi-a-GCST	Pt5VAH	456073	rs47341	0,378364	0,1197	0,001573
Neutro	Mitral	ebi-a-GCST	Pt5VAH	456073	rs4734879	0,377102	0,11976	0,001639
Neutro	Mitral	ebi-a-GCST	Pt5VAH	456073	rs4760	0,358596	0,120454	0,00291
Neutro	Mitral	ebi-a-GCST	Pt5VAH	456073	rs4761234	0,385514	0,119626	0,00127
Neutro	Mitral	ebi-a-GCST	Pt5VAH	456073	rs486650	0,382485	0,119521	0,001374
Neutro	Mitral	ebi-a-GCST	Pt5VAH	456073	rs488639	0,379787	0,119673	0,001506
Neutro	Mitral	ebi-a-GCST	Pt5VAH	456073	rs4924450	0,378829	0,119708	0,001553
Neutro	Mitral	ebi-a-GCST	Pt5VAH	456073	rs4983590	0,376664	0,119719	0,001654
Neutro	Mitral	ebi-a-GCST	Pt5VAH	456073	rs501791	0,375834	0,119666	0,001686
Neutro	Mitral	ebi-a-GCST	Pt5VAH	456073	rs509596	0,3781	0,119697	0,001584
Neutro	Mitral	ebi-a-GCST	Pt5VAH	456073	rs5565917(	0,378283	0,11976	0,001585
Neutro	Mitral	ebi-a-GCST	Pt5VAH	456073	rs5569060!	0,376555	0,119674	0,001652
Neutro	Mitral	ebi-a-GCST	Pt5VAH	456073	rs5570927:	0,386548	0,120095	0,001288
Neutro	Mitral	ebi-a-GCST	Pt5VAH	456073	rs5576780(	0,378448	0,119703	0,001569
Neutro	Mitral	ebi-a-GCST	Pt5VAH	456073	rs5587615:	0,3795	0,119766	0,001531
Neutro	Mitral	ebi-a-GCST	Pt5VAH	456073	rs5590432ι	0,383131	0,119422	0,001336
Neutro	Mitral	ebi-a-GCST	Pt5VAH	456073	rs5593813(	0,381713	0,119704	0,001429
Neutro	Mitral	ebi-a-GCST	Pt5VAH	456073	rs5618886!	0,384055	0,119792	0,001346
Neutro	Mitral	ebi-a-GCST	Pt5VAH	456073	rs5619533ι	0,378247	0,119765	0,001587
Neutro	Mitral	ebi-a-GCST	Pt5VAH	456073	rs5621714!	0,38306	0,119436	0,00134
Neutro	Mitral	ebi-a-GCST	Pt5VAH	456073	rs5624971:	0,381459	0,119654	0,001433
Neutro	Mitral	ebi-a-GCST	Pt5VAH	456073	rs5629302!	0,380562	0,119969	0,001513
Neutro	Mitral	ebi-a-GCST	Pt5VAH	456073	rs5638817(	0,38596	0,12107	0,001433
Neutro	Mitral	ebi-a-GCST	Pt5VAH	456073	rs571497	0,374457	0,11988	0,001787

Neutro	Mitral	ebi-a-GCST	Pt5VAH	456073	rs5746451	0,384042	0,119695	0,001334
Neutro	Mitral	ebi-a-GCST	Pt5VAH	456073	rs579111	0,377634	0,119697	0,001605
Neutro	Mitral	ebi-a-GCST	Pt5VAH	456073	rs5843438	0,388443	0,119123	0,001111
Neutro	Mitral	ebi-a-GCST	Pt5VAH	456073	rs5898452	0,378862	0,119698	0,00155
Neutro	Mitral	ebi-a-GCST	Pt5VAH	456073	rs5951978	0,379576	0,119687	0,001517
Neutro	Mitral	ebi-a-GCST	Pt5VAH	456073	rs5969707	0,373884	0,120096	0,001851
Neutro	Mitral	ebi-a-GCST	Pt5VAH	456073	rs5994158	0,38183	0,11957	0,001406
Neutro	Mitral	ebi-a-GCST	Pt5VAH	456073	rs6011066	0,387117	0,118983	0,00114
Neutro	Mitral	ebi-a-GCST	Pt5VAH	456073	rs6091892	0,381792	0,119648	0,001418
Neutro	Mitral	ebi-a-GCST	Pt5VAH	456073	rs609264	0,379791	0,119682	0,001507
Neutro	Mitral	ebi-a-GCST	Pt5VAH	456073	rs6102539	0,379352	0,119755	0,001536
Neutro	Mitral	ebi-a-GCST	Pt5VAH	456073	rs611418	0,378288	0,119727	0,00158
Neutro	Mitral	ebi-a-GCST	Pt5VAH	456073	rs6142137	0,385067	0,119058	0,001219
Neutro	Mitral	ebi-a-GCST	Pt5VAH	456073	rs6173928	0,371893	0,119361	0,001835
Neutro	Mitral	ebi-a-GCST	Pt5VAH	456073	rs6183875	0,384682	0,119501	0,001286
Neutro	Mitral	ebi-a-GCST	Pt5VAH	456073	rs6191836	0,383977	0,119239	0,001281
Neutro	Mitral	ebi-a-GCST	Pt5VAH	456073	rs6205722	0,375963	0,119602	0,00167
Neutro	Mitral	ebi-a-GCST	Pt5VAH	456073	rs6223992	0,379538	0,119697	0,00152
Neutro	Mitral	ebi-a-GCST	Pt5VAH	456073	rs6246631	0,375964	0,119744	0,001691
Neutro	Mitral	ebi-a-GCST	Pt5VAH	456073	rs6251027	0,383664	0,119648	0,001343
Neutro	Mitral	ebi-a-GCST	Pt5VAH	456073	rs632887	0,371988	0,119357	0,001829
Neutro	Mitral	ebi-a-GCST	Pt5VAH	456073	rs6456420	0,379639	0,119714	0,001518
Neutro	Mitral	ebi-a-GCST	Pt5VAH	456073	rs6465674	0,373977	0,119551	0,001759
Neutro	Mitral	ebi-a-GCST	Pt5VAH	456073	rs6500550	0,379051	0,119811	0,001558
Neutro	Mitral	ebi-a-GCST	Pt5VAH	456073	rs6577536	0,389908	0,119412	0,001094
Neutro	Mitral	ebi-a-GCST	Pt5VAH	456073	rs6679677	0,393214	0,118934	0,000946
Neutro	Mitral	ebi-a-GCST	Pt5VAH	456073	rs6717590	0,390035	0,119577	0,001107
Neutro	Mitral	ebi-a-GCST	Pt5VAH	456073	rs6726903	0,375838	0,11961	0,001677
Neutro	Mitral	ebi-a-GCST	Pt5VAH	456073	rs6753819	0,370021	0,119174	0,001904
Neutro	Mitral	ebi-a-GCST	Pt5VAH	456073	rs6755895	0,379102	0,119726	0,001543
Neutro	Mitral	ebi-a-GCST	Pt5VAH	456073	rs6764912	0,382868	0,119638	0,001373
Neutro	Mitral	ebi-a-GCST	Pt5VAH	456073	rs6779340	0,371665	0,11935	0,001845
Neutro	Mitral	ebi-a-GCST	Pt5VAH	456073	rs6782812	0,382915	0,120183	0,001442
Neutro	Mitral	ebi-a-GCST	Pt5VAH	456073	rs680775	0,377418	0,119745	0,001622
Neutro	Mitral	ebi-a-GCST	Pt5VAH	456073	rs6817881	0,374338	0,119506	0,001734
Neutro	Mitral	ebi-a-GCST	Pt5VAH	456073	rs6831368	0,375325	0,119775	0,001727
Neutro	Mitral	ebi-a-GCST	Pt5VAH	456073	rs6894901	0,383616	0,119316	0,001304
Neutro	Mitral	ebi-a-GCST	Pt5VAH	456073	rs6924387	0,371141	0,119351	0,001873
Neutro	Mitral	ebi-a-GCST	Pt5VAH	456073	rs6927569	0,379838	0,11991	0,001536
Neutro	Mitral	ebi-a-GCST	Pt5VAH	456073	rs694180	0,377542	0,119747	0,001617
Neutro	Mitral	ebi-a-GCST	Pt5VAH	456073	rs6947629	0,377404	0,119687	0,001615
Neutro	Mitral	ebi-a-GCST	Pt5VAH	456073	rs6954012	0,373591	0,119562	0,00178
Neutro	Mitral	ebi-a-GCST	Pt5VAH	456073	rs696825	0,376537	0,11976	0,001666
Neutro	Mitral	ebi-a-GCST	Pt5VAH	456073	rs7005996	0,377605	0,119711	0,001609
Neutro	Mitral	ebi-a-GCST	Pt5VAH	456073	rs7129527	0,368041	0,118984	0,00198
Neutro	Mitral	ebi-a-GCST	Pt5VAH	456073	rs714624	0,37615	0,119667	0,001671
Neutro	Mitral	ebi-a-GCST	Pt5VAH	456073	rs7157692	0,374911	0,119539	0,001711
Neutro	Mitral	ebi-a-GCST	Pt5VAH	456073	rs716848	0,37813	0,119721	0,001586
Neutro	Mitral	ebi-a-GCST	Pt5VAH	456073	rs7180079	0,368363	0,11934	0,002024
Neutro	Mitral	ebi-a-GCST	Pt5VAH	456073	rs7183988	0,374643	0,119658	0,001742

Neutro	Mitral	ebi-a-GCST	Pt5VAH	456073	rs7225843	0,37078	0,119414	0,001903
Neutro	Mitral	ebi-a-GCST	Pt5VAH	456073	rs723585	0,389531	0,11932	0,001096
Neutro	Mitral	ebi-a-GCST	Pt5VAH	456073	rs7273156	0,372911	0,119466	0,001799
Neutro	Mitral	ebi-a-GCST	Pt5VAH	456073	rs7275650	0,378068	0,119704	0,001587
Neutro	Mitral	ebi-a-GCST	Pt5VAH	456073	rs7279086	0,373546	0,119631	0,001793
Neutro	Mitral	ebi-a-GCST	Pt5VAH	456073	rs7280332	0,382839	0,119489	0,001355
Neutro	Mitral	ebi-a-GCST	Pt5VAH	456073	rs7283930	0,375524	0,119588	0,001689
Neutro	Mitral	ebi-a-GCST	Pt5VAH	456073	rs7284460	0,371167	0,11934	0,00187
Neutro	Mitral	ebi-a-GCST	Pt5VAH	456073	rs7290175	0,382878	0,119495	0,001355
Neutro	Mitral	ebi-a-GCST	Pt5VAH	456073	rs7297371	0,379814	0,11977	0,001518
Neutro	Mitral	ebi-a-GCST	Pt5VAH	456073	rs729761	0,374088	0,119556	0,001754
Neutro	Mitral	ebi-a-GCST	Pt5VAH	456073	rs7319118	0,38499	0,119341	0,001256
Neutro	Mitral	ebi-a-GCST	Pt5VAH	456073	rs7326825	0,371182	0,119499	0,001895
Neutro	Mitral	ebi-a-GCST	Pt5VAH	456073	rs7407632	0,37972	0,119711	0,001514
Neutro	Mitral	ebi-a-GCST	Pt5VAH	456073	rs7425073	0,373494	0,119479	0,001772
Neutro	Mitral	ebi-a-GCST	Pt5VAH	456073	rs7452937	0,403535	0,120752	0,000832
Neutro	Mitral	ebi-a-GCST	Pt5VAH	456073	rs7487314	0,374622	0,119784	0,001763
Neutro	Mitral	ebi-a-GCST	Pt5VAH	456073	rs7516636	0,37902	0,119715	0,001545
Neutro	Mitral	ebi-a-GCST	Pt5VAH	456073	rs7547562	0,380215	0,119732	0,001496
Neutro	Mitral	ebi-a-GCST	Pt5VAH	456073	rs7552783	0,386019	0,119658	0,001255
Neutro	Mitral	ebi-a-GCST	Pt5VAH	456073	rs7604081	0,372218	0,119317	0,001811
Neutro	Mitral	ebi-a-GCST	Pt5VAH	456073	rs764358	0,381093	0,119647	0,001447
Neutro	Mitral	ebi-a-GCST	Pt5VAH	456073	rs7671414	0,376517	0,119665	0,001653
Neutro	Mitral	ebi-a-GCST	Pt5VAH	456073	rs7676497	0,379309	0,119685	0,001528
Neutro	Mitral	ebi-a-GCST	Pt5VAH	456073	rs7679673	0,378384	0,11985	0,001593
Neutro	Mitral	ebi-a-GCST	Pt5VAH	456073	rs7686326	0,381276	0,119648	0,001439
Neutro	Mitral	ebi-a-GCST	Pt5VAH	456073	rs7705526	0,366265	0,119704	0,002215
Neutro	Mitral	ebi-a-GCST	Pt5VAH	456073	rs7735891	0,379073	0,11982	0,001558
Neutro	Mitral	ebi-a-GCST	Pt5VAH	456073	rs7755226	0,38109	0,119851	0,001474
Neutro	Mitral	ebi-a-GCST	Pt5VAH	456073	rs7776857	0,373668	0,119504	0,001767
Neutro	Mitral	ebi-a-GCST	Pt5VAH	456073	rs7816785	0,384053	0,119624	0,001325
Neutro	Mitral	ebi-a-GCST	Pt5VAH	456073	rs7846314	0,355869	0,120245	0,003081
Neutro	Mitral	ebi-a-GCST	Pt5VAH	456073	rs7861010	0,376117	0,119713	0,001679
Neutro	Mitral	ebi-a-GCST	Pt5VAH	456073	rs789858	0,387103	0,118945	0,001136
Neutro	Mitral	ebi-a-GCST	Pt5VAH	456073	rs7904793	0,375251	0,119583	0,001701
Neutro	Mitral	ebi-a-GCST	Pt5VAH	456073	rs7956747	0,373813	0,11965	0,001783
Neutro	Mitral	ebi-a-GCST	Pt5VAH	456073	rs7968902	0,375354	0,119596	0,001698
Neutro	Mitral	ebi-a-GCST	Pt5VAH	456073	rs7971093	0,375536	0,119697	0,001705
Neutro	Mitral	ebi-a-GCST	Pt5VAH	456073	rs7995328	0,380202	0,11966	0,001486
Neutro	Mitral	ebi-a-GCST	Pt5VAH	456073	rs8024737	0,375815	0,119636	0,001682
Neutro	Mitral	ebi-a-GCST	Pt5VAH	456073	rs8045100	0,378852	0,119791	0,001564
Neutro	Mitral	ebi-a-GCST	Pt5VAH	456073	rs8065774	0,375051	0,119551	0,001706
Neutro	Mitral	ebi-a-GCST	Pt5VAH	456073	rs8098724	0,380698	0,119671	0,001467
Neutro	Mitral	ebi-a-GCST	Pt5VAH	456073	rs8705	0,37364	0,119629	0,001788
Neutro	Mitral	ebi-a-GCST	Pt5VAH	456073	rs896319	0,381036	0,119667	0,001452
Neutro	Mitral	ebi-a-GCST	Pt5VAH	456073	rs915125	0,377254	0,119722	0,001627
Neutro	Mitral	ebi-a-GCST	Pt5VAH	456073	rs9165	0,373628	0,119541	0,001775
Neutro	Mitral	ebi-a-GCST	Pt5VAH	456073	rs9265809	0,378956	0,120458	0,001655
Neutro	Mitral	ebi-a-GCST	Pt5VAH	456073	rs930232	0,383066	0,119715	0,001375
Neutro	Mitral	ebi-a-GCST	Pt5VAH	456073	rs9329341	0,383459	0,119615	0,001347

Neutro	Mitral	ebi-a-GCST	Pt5VAH	456073	rs9362415	0,363557	0,119296	0,002307
Neutro	Mitral	ebi-a-GCST	Pt5VAH	456073	rs9375447	0,379864	0,11971	0,001508
Neutro	Mitral	ebi-a-GCST	Pt5VAH	456073	rs9390461	0,375937	0,119715	0,001688
Neutro	Mitral	ebi-a-GCST	Pt5VAH	456073	rs9688952	0,37842	0,119697	0,00157
Neutro	Mitral	ebi-a-GCST	Pt5VAH	456073	rs9772631	0,375555	0,11963	0,001693
Neutro	Mitral	ebi-a-GCST	Pt5VAH	456073	rs9819371	0,376389	0,119819	0,001682
Neutro	Mitral	ebi-a-GCST	Pt5VAH	456073	rs9842724	0,37114	0,119026	0,00182
Neutro	Mitral	ebi-a-GCST	Pt5VAH	456073	rs9885207	0,379248	0,119752	0,00154
Neutro	Mitral	ebi-a-GCST	Pt5VAH	456073	rs9905106	0,390596	0,11898	0,001028
Neutro	Mitral	ebi-a-GCST	Pt5VAH	456073	rs9916458	0,382611	0,119563	0,001374
Neutro	Mitral	ebi-a-GCST	Pt5VAH	456073	rs9965539	0,374672	0,119767	0,001758
Neutro	Mitral	ebi-a-GCST	Pt5VAH	456073	rs9970896	0,388001	0,119978	0,001221
Neutro	Mitral	ebi-a-GCST	Pt5VAH	456073	rs9977672	0,371353	0,119563	0,001897
Neutro	Mitral	ebi-a-GCST	Pt5VAH	456073	rs998584	0,375027	0,119597	0,001714
Neutro	Mitral	ebi-a-GCST	Pt5VAH	456073	All	0,378378	0,119475	0,00154
Plt	Mitral	ebi-a-GCST	oOqrag	440846	rs1004874	-0,03575	0,086065	0,677867
Plt	Mitral	ebi-a-GCST	oOqrag	440846	rs1011697	-0,04381	0,085893	0,610016
Plt	Mitral	ebi-a-GCST	oOqrag	440846	rs1012777	-0,04408	0,086094	0,608691
Plt	Mitral	ebi-a-GCST	oOqrag	440846	rs1013541	-0,0381	0,086225	0,658578
Plt	Mitral	ebi-a-GCST	oOqrag	440846	rs1013919	-0,03958	0,086148	0,645922
Plt	Mitral	ebi-a-GCST	oOqrag	440846	rs1014066	-0,03802	0,086161	0,659045
Plt	Mitral	ebi-a-GCST	oOqrag	440846	rs1014427	-0,03712	0,086333	0,667236
Plt	Mitral	ebi-a-GCST	oOqrag	440846	rs10174	-0,04071	0,086135	0,636491
Plt	Mitral	ebi-a-GCST	oOqrag	440846	rs1035317	-0,04041	0,086139	0,638955
Plt	Mitral	ebi-a-GCST	oOqrag	440846	rs1044722	-0,04104	0,086088	0,633525
Plt	Mitral	ebi-a-GCST	oOqrag	440846	rs1045872	-0,03837	0,086177	0,656154
Plt	Mitral	ebi-a-GCST	oOqrag	440846	rs1047891	-0,0374	0,086223	0,664477
Plt	Mitral	ebi-a-GCST	oOqrag	440846	rs1051041	-0,03722	0,086121	0,665626
Plt	Mitral	ebi-a-GCST	oOqrag	440846	rs1053651	-0,03677	0,086028	0,669095
Plt	Mitral	ebi-a-GCST	oOqrag	440846	rs1075848	-0,03605	0,086194	0,675783
Plt	Mitral	ebi-a-GCST	oOqrag	440846	rs1076994	-0,03372	0,085667	0,693898
Plt	Mitral	ebi-a-GCST	oOqrag	440846	rs1077440	-0,02355	0,086022	0,784273
Plt	Mitral	ebi-a-GCST	oOqrag	440846	rs1078338	-0,03905	0,086209	0,650589
Plt	Mitral	ebi-a-GCST	oOqrag	440846	rs1081700	-0,0379	0,086151	0,659998
Plt	Mitral	ebi-a-GCST	oOqrag	440846	rs1082060	-0,04619	0,086239	0,592253
Plt	Mitral	ebi-a-GCST	oOqrag	440846	rs1082112	-0,0401	0,086137	0,641557
Plt	Mitral	ebi-a-GCST	oOqrag	440846	rs1086436	-0,03344	0,086033	0,697495
Plt	Mitral	ebi-a-GCST	oOqrag	440846	rs1087655	-0,02778	0,086334	0,747583
Plt	Mitral	ebi-a-GCST	oOqrag	440846	rs1087698	-0,04047	0,086108	0,638391
Plt	Mitral	ebi-a-GCST	oOqrag	440846	rs1087817	-0,04003	0,08615	0,64214
Plt	Mitral	ebi-a-GCST	oOqrag	440846	rs1088643	-0,04052	0,086152	0,638125
Plt	Mitral	ebi-a-GCST	oOqrag	440846	rs1091169	-0,03786	0,086139	0,660293
Plt	Mitral	ebi-a-GCST	oOqrag	440846	rs1094047	-0,03787	0,086136	0,66015
Plt	Mitral	ebi-a-GCST	oOqrag	440846	rs1094141	-0,03869	0,086158	0,653428
Plt	Mitral	ebi-a-GCST	oOqrag	440846	rs1097515	-0,04141	0,086029	0,63026
Plt	Mitral	ebi-a-GCST	oOqrag	440846	rs1107172	-0,03298	0,086235	0,702129
Plt	Mitral	ebi-a-GCST	oOqrag	440846	rs1108230	-0,03437	0,086458	0,690963
Plt	Mitral	ebi-a-GCST	oOqrag	440846	rs1108830	-0,04221	0,086132	0,62413
Plt	Mitral	ebi-a-GCST	oOqrag	440846	rs1112659	-0,03671	0,086083	0,669761
Plt	Mitral	ebi-a-GCST	oOqrag	440846	rs1112929	-0,03584	0,086024	0,676924

Plt	Mitral	ebi-a-GCST	oOqrag	440846	rs1113101	-0,04071	0,086099	0,636363
Plt	Mitral	ebi-a-GCST	oOqrag	440846	rs1113447	-0,03208	0,085673	0,70809
Plt	Mitral	ebi-a-GCST	oOqrag	440846	rs1116413	-0,03754	0,086219	0,663307
Plt	Mitral	ebi-a-GCST	oOqrag	440846	rs1116824	-0,03856	0,08627	0,654911
Plt	Mitral	ebi-a-GCST	oOqrag	440846	rs1120964	-0,0388	0,086161	0,652517
Plt	Mitral	ebi-a-GCST	oOqrag	440846	rs1121832	-0,04257	0,085933	0,620352
Plt	Mitral	ebi-a-GCST	oOqrag	440846	rs1122502	-0,03883	0,086159	0,652188
Plt	Mitral	ebi-a-GCST	oOqrag	440846	rs1122724	-0,03901	0,086223	0,650993
Plt	Mitral	ebi-a-GCST	oOqrag	440846	rs1125918	-0,03854	0,086169	0,654709
Plt	Mitral	ebi-a-GCST	oOqrag	440846	rs1127338	-0,04107	0,086116	0,633414
Plt	Mitral	ebi-a-GCST	oOqrag	440846	rs1127707	-0,04106	0,086157	0,633676
Plt	Mitral	ebi-a-GCST	oOqrag	440846	rs1130630	-0,03965	0,086185	0,645494
Plt	Mitral	ebi-a-GCST	oOqrag	440846	rs1131468	-0,04383	0,085735	0,609191
Plt	Mitral	ebi-a-GCST	oOqrag	440846	rs1135423	-0,03999	0,086317	0,643167
Plt	Mitral	ebi-a-GCST	oOqrag	440846	rs1136934	-0,03801	0,086206	0,659229
Plt	Mitral	ebi-a-GCST	oOqrag	440846	rs1137214	-0,03475	0,086128	0,686566
Plt	Mitral	ebi-a-GCST	oOqrag	440846	rs1138251	-0,04075	0,086111	0,636045
Plt	Mitral	ebi-a-GCST	oOqrag	440846	rs1146941	-0,04917	0,086642	0,570405
Plt	Mitral	ebi-a-GCST	oOqrag	440846	rs1147004	-0,04283	0,085923	0,618125
Plt	Mitral	ebi-a-GCST	oOqrag	440846	rs1149680	-0,04324	0,085983	0,615028
Plt	Mitral	ebi-a-GCST	oOqrag	440846	rs1152435	-0,03658	0,086235	0,671449
Plt	Mitral	ebi-a-GCST	oOqrag	440846	rs1152948	-0,0438	0,085457	0,608302
Plt	Mitral	ebi-a-GCST	oOqrag	440846	rs1154674	-0,03795	0,086147	0,659524
Plt	Mitral	ebi-a-GCST	oOqrag	440846	rs1155369	-0,03591	0,086489	0,677963
Plt	Mitral	ebi-a-GCST	oOqrag	440846	rs1155663	-0,04094	0,086061	0,634312
Plt	Mitral	ebi-a-GCST	oOqrag	440846	rs1158221	-0,03653	0,085976	0,670924
Plt	Mitral	ebi-a-GCST	oOqrag	440846	rs1160412	-0,04951	0,086874	0,568709
Plt	Mitral	ebi-a-GCST	oOqrag	440846	rs1160528	-0,04013	0,086173	0,641429
Plt	Mitral	ebi-a-GCST	oOqrag	440846	rs1161185	-0,03992	0,086196	0,643297
Plt	Mitral	ebi-a-GCST	oOqrag	440846	rs1162748	-0,04071	0,086198	0,636733
Plt	Mitral	ebi-a-GCST	oOqrag	440846	rs1167629	-0,042	0,086143	0,625853
Plt	Mitral	ebi-a-GCST	oOqrag	440846	rs1167932	-0,03877	0,086159	0,652752
Plt	Mitral	ebi-a-GCST	oOqrag	440846	rs1171233	-0,0396	0,086148	0,645783
Plt	Mitral	ebi-a-GCST	oOqrag	440846	rs1173409	-0,02697	0,085928	0,753651
Plt	Mitral	ebi-a-GCST	oOqrag	440846	rs1176439	-0,04391	0,086031	0,609801
Plt	Mitral	ebi-a-GCST	oOqrag	440846	rs1179352	-0,03814	0,086151	0,657938
Plt	Mitral	ebi-a-GCST	oOqrag	440846	rs1180838	-0,04067	0,086083	0,636621
Plt	Mitral	ebi-a-GCST	oOqrag	440846	rs1181846	-0,04307	0,086072	0,616765
Plt	Mitral	ebi-a-GCST	oOqrag	440846	rs1182689	-0,0385	0,086152	0,654999
Plt	Mitral	ebi-a-GCST	oOqrag	440846	rs1184131	-0,03218	0,086217	0,708993
Plt	Mitral	ebi-a-GCST	oOqrag	440846	rs1187909	-0,03603	0,08595	0,67511
Plt	Mitral	ebi-a-GCST	oOqrag	440846	rs1189155	-0,04133	0,086038	0,630989
Plt	Mitral	ebi-a-GCST	oOqrag	440846	rs1191713	-0,03837	0,086185	0,656178
Plt	Mitral	ebi-a-GCST	oOqrag	440846	rs1195056	-0,03821	0,086207	0,657613
Plt	Mitral	ebi-a-GCST	oOqrag	440846	rs1200025	-0,04221	0,086084	0,623891
Plt	Mitral	ebi-a-GCST	oOqrag	440846	rs1203713	-0,0449	0,086433	0,603459
Plt	Mitral	ebi-a-GCST	oOqrag	440846	rs1204133	-0,04309	0,086004	0,616351
Plt	Mitral	ebi-a-GCST	oOqrag	440846	rs1205271	-0,03751	0,086279	0,663708
Plt	Mitral	ebi-a-GCST	oOqrag	440846	rs1211806	-0,04498	0,086064	0,601235
Plt	Mitral	ebi-a-GCST	oOqrag	440846	rs1211989	-0,03226	0,085708	0,706649

Plt	Mitral	ebi-a-GCST	oOqrag	440846	rs1212129	-0,03816	0,086138	0,657765
Plt	Mitral	ebi-a-GCST	oOqrag	440846	rs1212520	-0,03684	0,086224	0,669158
Plt	Mitral	ebi-a-GCST	oOqrag	440846	rs1212619	-0,04048	0,086156	0,638471
Plt	Mitral	ebi-a-GCST	oOqrag	440846	rs1213909	-0,04094	0,086107	0,634461
Plt	Mitral	ebi-a-GCST	oOqrag	440846	rs1214916	-0,04005	0,086169	0,642107
Plt	Mitral	ebi-a-GCST	oOqrag	440846	rs1215503	-0,03698	0,086279	0,668206
Plt	Mitral	ebi-a-GCST	oOqrag	440846	rs1223973	-0,03624	0,085961	0,673333
Plt	Mitral	ebi-a-GCST	oOqrag	440846	rs1224382	-0,03853	0,086181	0,65482
Plt	Mitral	ebi-a-GCST	oOqrag	440846	rs1237651	-0,03863	0,086302	0,654465
Plt	Mitral	ebi-a-GCST	oOqrag	440846	rs1238684	-0,03724	0,086102	0,665394
Plt	Mitral	ebi-a-GCST	oOqrag	440846	rs1242583	-0,03342	0,085896	0,697214
Plt	Mitral	ebi-a-GCST	oOqrag	440846	rs1243914	-0,03481	0,085907	0,685362
Plt	Mitral	ebi-a-GCST	oOqrag	440846	rs1244145	-0,03881	0,08616	0,652366
Plt	Mitral	ebi-a-GCST	oOqrag	440846	rs1245147	-0,03729	0,086114	0,665026
Plt	Mitral	ebi-a-GCST	oOqrag	440846	rs1248129	-0,04025	0,08612	0,640255
Plt	Mitral	ebi-a-GCST	oOqrag	440846	rs1249178	-0,04095	0,086179	0,634655
Plt	Mitral	ebi-a-GCST	oOqrag	440846	rs1249269	-0,04064	0,086077	0,636798
Plt	Mitral	ebi-a-GCST	oOqrag	440846	rs1253340	-0,04161	0,086069	0,628743
Plt	Mitral	ebi-a-GCST	oOqrag	440846	rs1256480	-0,04105	0,086271	0,634202
Plt	Mitral	ebi-a-GCST	oOqrag	440846	rs1258012	-0,03748	0,086143	0,663455
Plt	Mitral	ebi-a-GCST	oOqrag	440846	rs1260326	-0,03579	0,086333	0,67846
Plt	Mitral	ebi-a-GCST	oOqrag	440846	rs1260405	-0,04017	0,086174	0,641089
Plt	Mitral	ebi-a-GCST	oOqrag	440846	rs1267610	-0,04576	0,085636	0,593072
Plt	Mitral	ebi-a-GCST	oOqrag	440846	rs1270851	-0,04614	0,085597	0,589875
Plt	Mitral	ebi-a-GCST	oOqrag	440846	rs1275446	-0,04015	0,086181	0,641333
Plt	Mitral	ebi-a-GCST	oOqrag	440846	rs1276293	-0,03825	0,086175	0,65712
Plt	Mitral	ebi-a-GCST	oOqrag	440846	rs1282945	-0,03636	0,086162	0,673014
Plt	Mitral	ebi-a-GCST	oOqrag	440846	rs1290913	-0,03936	0,086164	0,647779
Plt	Mitral	ebi-a-GCST	oOqrag	440846	rs1292331	-0,0421	0,085957	0,624273
Plt	Mitral	ebi-a-GCST	oOqrag	440846	rs1297557	-0,03792	0,086158	0,65984
Plt	Mitral	ebi-a-GCST	oOqrag	440846	rs1297989	-0,03681	0,086092	0,668937
Plt	Mitral	ebi-a-GCST	oOqrag	440846	rs1298301	-0,03151	0,085841	0,713541
Plt	Mitral	ebi-a-GCST	oOqrag	440846	rs1298407	-0,03852	0,086152	0,654827
Plt	Mitral	ebi-a-GCST	oOqrag	440846	rs1300064	-0,03858	0,086154	0,65433
Plt	Mitral	ebi-a-GCST	oOqrag	440846	rs1303249	-0,04029	0,086106	0,639856
Plt	Mitral	ebi-a-GCST	oOqrag	440846	rs1308431	-0,03649	0,086077	0,671656
Plt	Mitral	ebi-a-GCST	oOqrag	440846	rs1308435	-0,03735	0,086206	0,664849
Plt	Mitral	ebi-a-GCST	oOqrag	440846	rs1313872	-0,03825	0,086163	0,657116
Plt	Mitral	ebi-a-GCST	oOqrag	440846	rs1315083	-0,04095	0,086168	0,634608
Plt	Mitral	ebi-a-GCST	oOqrag	440846	rs1315490	-0,03606	0,086021	0,675038
Plt	Mitral	ebi-a-GCST	oOqrag	440846	rs1318548	-0,03843	0,086175	0,655632
Plt	Mitral	ebi-a-GCST	oOqrag	440846	rs1319194	-0,03742	0,086202	0,664225
Plt	Mitral	ebi-a-GCST	oOqrag	440846	rs1320804	-0,03858	0,086183	0,654385
Plt	Mitral	ebi-a-GCST	oOqrag	440846	rs1322408	-0,03755	0,086243	0,663253
Plt	Mitral	ebi-a-GCST	oOqrag	440846	rs1324195	-0,03571	0,086105	0,678369
Plt	Mitral	ebi-a-GCST	oOqrag	440846	rs1326644	-0,04225	0,086115	0,623711
Plt	Mitral	ebi-a-GCST	oOqrag	440846	rs1332569	-0,03011	0,085914	0,726001
Plt	Mitral	ebi-a-GCST	oOqrag	440846	rs1339481	-0,0386	0,086169	0,654186
Plt	Mitral	ebi-a-GCST	oOqrag	440846	rs1354034	-0,02837	0,088389	0,748254
Plt	Mitral	ebi-a-GCST	oOqrag	440846	rs1399746	-0,04634	0,086169	0,59073

Plt	Mitral	ebi-a-GCST	oOqrag	440846	rs1404361!	-0,03929	0,086196	0,648514
Plt	Mitral	ebi-a-GCST	oOqrag	440846	rs1407590	-0,03838	0,086156	0,655963
Plt	Mitral	ebi-a-GCST	oOqrag	440846	rs1443170	-0,03995	0,086155	0,642894
Plt	Mitral	ebi-a-GCST	oOqrag	440846	rs1456638	-0,03856	0,086155	0,654456
Plt	Mitral	ebi-a-GCST	oOqrag	440846	rs1472265	-0,03889	0,086167	0,651743
Plt	Mitral	ebi-a-GCST	oOqrag	440846	rs1476096	-0,04038	0,086166	0,639297
Plt	Mitral	ebi-a-GCST	oOqrag	440846	rs151234	-0,04219	0,086377	0,625272
Plt	Mitral	ebi-a-GCST	oOqrag	440846	rs1569419	-0,03783	0,086172	0,66069
Plt	Mitral	ebi-a-GCST	oOqrag	440846	rs1602456	-0,03507	0,086063	0,683674
Plt	Mitral	ebi-a-GCST	oOqrag	440846	rs160697	-0,0383	0,086148	0,656603
Plt	Mitral	ebi-a-GCST	oOqrag	440846	rs1641861	-0,03892	0,086167	0,651516
Plt	Mitral	ebi-a-GCST	oOqrag	440846	rs165177	-0,03481	0,085837	0,685048
Plt	Mitral	ebi-a-GCST	oOqrag	440846	rs1660155	-0,03786	0,086127	0,660201
Plt	Mitral	ebi-a-GCST	oOqrag	440846	rs167454	-0,04037	0,086121	0,639276
Plt	Mitral	ebi-a-GCST	oOqrag	440846	rs167924	-0,0433	0,086013	0,614658
Plt	Mitral	ebi-a-GCST	oOqrag	440846	rs1686629	-0,03841	0,086147	0,65573
Plt	Mitral	ebi-a-GCST	oOqrag	440846	rs1688959	-0,04054	0,086113	0,637775
Plt	Mitral	ebi-a-GCST	oOqrag	440846	rs1694359!	-0,04362	0,085778	0,611046
Plt	Mitral	ebi-a-GCST	oOqrag	440846	rs1711645	-0,03885	0,086171	0,652095
Plt	Mitral	ebi-a-GCST	oOqrag	440846	rs1713872	-0,03853	0,086222	0,654936
Plt	Mitral	ebi-a-GCST	oOqrag	440846	rs1716505	-0,04887	0,086067	0,570146
Plt	Mitral	ebi-a-GCST	oOqrag	440846	rs1717583	-0,03599	0,08622	0,67639
Plt	Mitral	ebi-a-GCST	oOqrag	440846	rs1719271	-0,04556	0,086174	0,596984
Plt	Mitral	ebi-a-GCST	oOqrag	440846	rs1730200!	-0,04014	0,086136	0,641199
Plt	Mitral	ebi-a-GCST	oOqrag	440846	rs1757210!	-0,03665	0,086251	0,670854
Plt	Mitral	ebi-a-GCST	oOqrag	440846	rs1768584	-0,04133	0,08642	0,632472
Plt	Mitral	ebi-a-GCST	oOqrag	440846	rs1770746	-0,03685	0,086077	0,668593
Plt	Mitral	ebi-a-GCST	oOqrag	440846	rs1775869!	-0,04079	0,086182	0,636017
Plt	Mitral	ebi-a-GCST	oOqrag	440846	rs178010	-0,03819	0,086148	0,657562
Plt	Mitral	ebi-a-GCST	oOqrag	440846	rs178761	-0,03873	0,086163	0,653079
Plt	Mitral	ebi-a-GCST	oOqrag	440846	rs1835831	-0,03308	0,08582	0,699919
Plt	Mitral	ebi-a-GCST	oOqrag	440846	rs1865761	-0,03969	0,086155	0,645001
Plt	Mitral	ebi-a-GCST	oOqrag	440846	rs1882076	-0,03712	0,086096	0,666359
Plt	Mitral	ebi-a-GCST	oOqrag	440846	rs1895490	-0,03281	0,085709	0,701875
Plt	Mitral	ebi-a-GCST	oOqrag	440846	rs1924930	-0,03773	0,086117	0,661301
Plt	Mitral	ebi-a-GCST	oOqrag	440846	rs1982101	-0,04159	0,086109	0,629079
Plt	Mitral	ebi-a-GCST	oOqrag	440846	rs198217	-0,0395	0,086149	0,646565
Plt	Mitral	ebi-a-GCST	oOqrag	440846	rs1997719	-0,04078	0,08617	0,635995
Plt	Mitral	ebi-a-GCST	oOqrag	440846	rs2015210	-0,03832	0,086203	0,656618
Plt	Mitral	ebi-a-GCST	oOqrag	440846	rs2015599	-0,03649	0,086394	0,672761
Plt	Mitral	ebi-a-GCST	oOqrag	440846	rs2017372	-0,04073	0,086093	0,636147
Plt	Mitral	ebi-a-GCST	oOqrag	440846	rs2038700	-0,03934	0,08617	0,647967
Plt	Mitral	ebi-a-GCST	oOqrag	440846	rs2046823	-0,03664	0,086202	0,670762
Plt	Mitral	ebi-a-GCST	oOqrag	440846	rs2068888	-0,03945	0,086204	0,647186
Plt	Mitral	ebi-a-GCST	oOqrag	440846	rs2070738	-0,04106	0,086067	0,63334
Plt	Mitral	ebi-a-GCST	oOqrag	440846	rs210798	-0,04054	0,086158	0,637985
Plt	Mitral	ebi-a-GCST	oOqrag	440846	rs211510	-0,03934	0,086162	0,647959
Plt	Mitral	ebi-a-GCST	oOqrag	440846	rs2122403	-0,03763	0,086113	0,662139
Plt	Mitral	ebi-a-GCST	oOqrag	440846	rs218265	-0,04118	0,08607	0,632363
Plt	Mitral	ebi-a-GCST	oOqrag	440846	rs2187416	-0,03479	0,085742	0,684902

Plt	Mitral	ebi-a-GCST	oOqrag	440846	rs2243103	-0,03476	0,086291	0,687096
Plt	Mitral	ebi-a-GCST	oOqrag	440846	rs2255531	-0,03662	0,086169	0,670879
Plt	Mitral	ebi-a-GCST	oOqrag	440846	rs2274319	-0,03736	0,086214	0,664742
Plt	Mitral	ebi-a-GCST	oOqrag	440846	rs2281232	-0,03831	0,086146	0,656541
Plt	Mitral	ebi-a-GCST	oOqrag	440846	rs2297066	-0,04081	0,086394	0,636676
Plt	Mitral	ebi-a-GCST	oOqrag	440846	rs2305769	-0,03917	0,08616	0,649359
Plt	Mitral	ebi-a-GCST	oOqrag	440846	rs2319627	-0,03701	0,086044	0,667131
Plt	Mitral	ebi-a-GCST	oOqrag	440846	rs2389874	-0,03976	0,086182	0,644551
Plt	Mitral	ebi-a-GCST	oOqrag	440846	rs2417965	-0,03485	0,085831	0,684738
Plt	Mitral	ebi-a-GCST	oOqrag	440846	rs2455425	-0,04	0,086127	0,642369
Plt	Mitral	ebi-a-GCST	oOqrag	440846	rs2475218	-0,03877	0,086169	0,652725
Plt	Mitral	ebi-a-GCST	oOqrag	440846	rs2494663	-0,03912	0,086199	0,649948
Plt	Mitral	ebi-a-GCST	oOqrag	440846	rs2520096	-0,03596	0,086216	0,676573
Plt	Mitral	ebi-a-GCST	oOqrag	440846	rs2641443	-0,05098	0,085425	0,550672
Plt	Mitral	ebi-a-GCST	oOqrag	440846	rs2689216	-0,03589	0,086056	0,676614
Plt	Mitral	ebi-a-GCST	oOqrag	440846	rs2700936	-0,03782	0,086158	0,660691
Plt	Mitral	ebi-a-GCST	oOqrag	440846	rs2722159	-0,03969	0,086153	0,645027
Plt	Mitral	ebi-a-GCST	oOqrag	440846	rs2724564	-0,03994	0,086217	0,643174
Plt	Mitral	ebi-a-GCST	oOqrag	440846	rs2755214	-0,03887	0,086203	0,652022
Plt	Mitral	ebi-a-GCST	oOqrag	440846	rs2758994	-0,03822	0,086168	0,657385
Plt	Mitral	ebi-a-GCST	oOqrag	440846	rs2786802	-0,04046	0,086116	0,638492
Plt	Mitral	ebi-a-GCST	oOqrag	440846	rs2788500	-0,03715	0,086155	0,666318
Plt	Mitral	ebi-a-GCST	oOqrag	440846	rs2797688	-0,03905	0,086193	0,650552
Plt	Mitral	ebi-a-GCST	oOqrag	440846	rs2823002	-0,03713	0,086077	0,666178
Plt	Mitral	ebi-a-GCST	oOqrag	440846	rs2836425	-0,03851	0,086226	0,655137
Plt	Mitral	ebi-a-GCST	oOqrag	440846	rs28456	-0,03829	0,086318	0,657356
Plt	Mitral	ebi-a-GCST	oOqrag	440846	rs2850567	-0,04127	0,086231	0,632235
Plt	Mitral	ebi-a-GCST	oOqrag	440846	rs2887399	-0,03669	0,086031	0,669793
Plt	Mitral	ebi-a-GCST	oOqrag	440846	rs2900177	-0,03536	0,085968	0,680803
Plt	Mitral	ebi-a-GCST	oOqrag	440846	rs296852	-0,04541	0,086262	0,598584
Plt	Mitral	ebi-a-GCST	oOqrag	440846	rs2999157	-0,03815	0,086258	0,658323
Plt	Mitral	ebi-a-GCST	oOqrag	440846	rs301371	-0,03487	0,086278	0,686058
Plt	Mitral	ebi-a-GCST	oOqrag	440846	rs303968	-0,04134	0,086122	0,631248
Plt	Mitral	ebi-a-GCST	oOqrag	440846	rs3122414	-0,04172	0,086036	0,627726
Plt	Mitral	ebi-a-GCST	oOqrag	440846	rs3184504	-0,03984	0,087618	0,64935
Plt	Mitral	ebi-a-GCST	oOqrag	440846	rs3414360	-0,03605	0,08603	0,675182
Plt	Mitral	ebi-a-GCST	oOqrag	440846	rs3415216	-0,03904	0,086164	0,650525
Plt	Mitral	ebi-a-GCST	oOqrag	440846	rs3416410	-0,03855	0,087694	0,660212
Plt	Mitral	ebi-a-GCST	oOqrag	440846	rs342299	-0,04565	0,08676	0,598806
Plt	Mitral	ebi-a-GCST	oOqrag	440846	rs34329	-0,0366	0,086076	0,670668
Plt	Mitral	ebi-a-GCST	oOqrag	440846	rs3434192	-0,03668	0,086167	0,67031
Plt	Mitral	ebi-a-GCST	oOqrag	440846	rs3434655	-0,03698	0,08611	0,667555
Plt	Mitral	ebi-a-GCST	oOqrag	440846	rs3452489	-0,03849	0,086336	0,655696
Plt	Mitral	ebi-a-GCST	oOqrag	440846	rs3459282	-0,03861	0,086586	0,655671
Plt	Mitral	ebi-a-GCST	oOqrag	440846	rs34651	-0,04084	0,086189	0,635603
Plt	Mitral	ebi-a-GCST	oOqrag	440846	rs3496891	-0,03795	0,086158	0,659612
Plt	Mitral	ebi-a-GCST	oOqrag	440846	rs3506996	-0,03771	0,086145	0,661584
Plt	Mitral	ebi-a-GCST	oOqrag	440846	rs3526705	-0,03566	0,08599	0,678399
Plt	Mitral	ebi-a-GCST	oOqrag	440846	rs3529256	-0,04072	0,086092	0,636213
Plt	Mitral	ebi-a-GCST	oOqrag	440846	rs3533052	-0,0366	0,086128	0,67088

Plt	Mitral	ebi-a-GCST	oOqrag	440846	rs3535514(	-0,04241	0,085905	0,621526
Plt	Mitral	ebi-a-GCST	oOqrag	440846	rs3558407!	-0,03686	0,086151	0,668742
Plt	Mitral	ebi-a-GCST	oOqrag	440846	rs3577352:	-0,0411	0,086063	0,632943
Plt	Mitral	ebi-a-GCST	oOqrag	440846	rs3610990:	-0,03508	0,086536	0,685177
Plt	Mitral	ebi-a-GCST	oOqrag	440846	rs3620539	-0,0366	0,086096	0,670716
Plt	Mitral	ebi-a-GCST	oOqrag	440846	rs3731211	-0,03398	0,086281	0,693678
Plt	Mitral	ebi-a-GCST	oOqrag	440846	rs3736313	-0,03468	0,085753	0,6859
Plt	Mitral	ebi-a-GCST	oOqrag	440846	rs3740395(	-0,03983	0,086144	0,643789
Plt	Mitral	ebi-a-GCST	oOqrag	440846	rs3742028	-0,04142	0,086386	0,631575
Plt	Mitral	ebi-a-GCST	oOqrag	440846	rs3742271	-0,03978	0,086166	0,64436
Plt	Mitral	ebi-a-GCST	oOqrag	440846	rs3742321	-0,03998	0,08614	0,642566
Plt	Mitral	ebi-a-GCST	oOqrag	440846	rs3760868	-0,03725	0,086124	0,665338
Plt	Mitral	ebi-a-GCST	oOqrag	440846	rs3764535	-0,04059	0,086168	0,637611
Plt	Mitral	ebi-a-GCST	oOqrag	440846	rs3767812	-0,04326	0,086187	0,615703
Plt	Mitral	ebi-a-GCST	oOqrag	440846	rs3771529	-0,03695	0,086196	0,668178
Plt	Mitral	ebi-a-GCST	oOqrag	440846	rs3800458	-0,03369	0,086022	0,695318
Plt	Mitral	ebi-a-GCST	oOqrag	440846	rs3804749	-0,04151	0,086298	0,630524
Plt	Mitral	ebi-a-GCST	oOqrag	440846	rs3806233	-0,04049	0,086113	0,63825
Plt	Mitral	ebi-a-GCST	oOqrag	440846	rs381500	-0,03343	0,086134	0,697944
Plt	Mitral	ebi-a-GCST	oOqrag	440846	rs385893	-0,01747	0,087531	0,841766
Plt	Mitral	ebi-a-GCST	oOqrag	440846	rs3865444	-0,0356	0,086119	0,679348
Plt	Mitral	ebi-a-GCST	oOqrag	440846	rs3883952	-0,03844	0,086183	0,655543
Plt	Mitral	ebi-a-GCST	oOqrag	440846	rs3912392	-0,04206	0,085999	0,624819
Plt	Mitral	ebi-a-GCST	oOqrag	440846	rs3943516	-0,04088	0,086096	0,634911
Plt	Mitral	ebi-a-GCST	oOqrag	440846	rs3956381	-0,04077	0,086111	0,635869
Plt	Mitral	ebi-a-GCST	oOqrag	440846	rs4061659	-0,04191	0,086237	0,626994
Plt	Mitral	ebi-a-GCST	oOqrag	440846	rs4072669	-0,04012	0,086174	0,641506
Plt	Mitral	ebi-a-GCST	oOqrag	440846	rs4076566	-0,03821	0,086168	0,657451
Plt	Mitral	ebi-a-GCST	oOqrag	440846	rs4137514!	-0,03619	0,086052	0,674087
Plt	Mitral	ebi-a-GCST	oOqrag	440846	rs4148445	-0,04121	0,086353	0,633218
Plt	Mitral	ebi-a-GCST	oOqrag	440846	rs4286082	-0,03987	0,086154	0,643508
Plt	Mitral	ebi-a-GCST	oOqrag	440846	rs4357501	-0,03782	0,086122	0,660522
Plt	Mitral	ebi-a-GCST	oOqrag	440846	rs4377525	-0,03674	0,086183	0,669888
Plt	Mitral	ebi-a-GCST	oOqrag	440846	rs4442541	-0,04049	0,08617	0,63842
Plt	Mitral	ebi-a-GCST	oOqrag	440846	rs4477285	-0,04095	0,086129	0,634425
Plt	Mitral	ebi-a-GCST	oOqrag	440846	rs4538653	-0,03911	0,086353	0,650607
Plt	Mitral	ebi-a-GCST	oOqrag	440846	rs4588	-0,04107	0,08605	0,633197
Plt	Mitral	ebi-a-GCST	oOqrag	440846	rs458857	-0,04021	0,086142	0,640676
Plt	Mitral	ebi-a-GCST	oOqrag	440846	rs4670779	-0,03818	0,086172	0,657709
Plt	Mitral	ebi-a-GCST	oOqrag	440846	rs4686534	-0,04101	0,08607	0,633739
Plt	Mitral	ebi-a-GCST	oOqrag	440846	rs4711890	-0,0404	0,086215	0,639358
Plt	Mitral	ebi-a-GCST	oOqrag	440846	rs4733495	-0,04043	0,08616	0,638907
Plt	Mitral	ebi-a-GCST	oOqrag	440846	rs4754299	-0,03895	0,086197	0,651369
Plt	Mitral	ebi-a-GCST	oOqrag	440846	rs4771666	-0,03537	0,085808	0,680194
Plt	Mitral	ebi-a-GCST	oOqrag	440846	rs4780355	-0,03672	0,086133	0,669913
Plt	Mitral	ebi-a-GCST	oOqrag	440846	rs4783189	-0,04175	0,08621	0,628147
Plt	Mitral	ebi-a-GCST	oOqrag	440846	rs4793987	-0,04044	0,086108	0,638628
Plt	Mitral	ebi-a-GCST	oOqrag	440846	rs4815915	-0,04015	0,086159	0,641215
Plt	Mitral	ebi-a-GCST	oOqrag	440846	rs4818033	-0,03848	0,08618	0,655225
Plt	Mitral	ebi-a-GCST	oOqrag	440846	rs4846082	-0,03011	0,086137	0,726648

Plt	Mitral	ebi-a-GCST	oOqrag	440846	rs4849845	-0,02535	0,085568	0,766998
Plt	Mitral	ebi-a-GCST	oOqrag	440846	rs4883519	-0,04032	0,086112	0,639631
Plt	Mitral	ebi-a-GCST	oOqrag	440846	rs4902244	-0,04086	0,086066	0,634951
Plt	Mitral	ebi-a-GCST	oOqrag	440846	rs4913405	-0,03864	0,086169	0,653826
Plt	Mitral	ebi-a-GCST	oOqrag	440846	rs4936066	-0,0395	0,086161	0,646602
Plt	Mitral	ebi-a-GCST	oOqrag	440846	rs4946366	-0,03881	0,086159	0,652368
Plt	Mitral	ebi-a-GCST	oOqrag	440846	rs4947490	-0,03644	0,086066	0,672047
Plt	Mitral	ebi-a-GCST	oOqrag	440846	rs4965426	-0,03351	0,086107	0,697144
Plt	Mitral	ebi-a-GCST	oOqrag	440846	rs4972760	-0,04121	0,086042	0,631971
Plt	Mitral	ebi-a-GCST	oOqrag	440846	rs4979859	-0,03955	0,086173	0,646287
Plt	Mitral	ebi-a-GCST	oOqrag	440846	rs511515	-0,06105	0,087285	0,484249
Plt	Mitral	ebi-a-GCST	oOqrag	440846	rs5566415	-0,04146	0,086134	0,630268
Plt	Mitral	ebi-a-GCST	oOqrag	440846	rs5570881	-0,03707	0,086154	0,667015
Plt	Mitral	ebi-a-GCST	oOqrag	440846	rs5577354	-0,04394	0,085814	0,608612
Plt	Mitral	ebi-a-GCST	oOqrag	440846	rs5579472	-0,04168	0,086285	0,629082
Plt	Mitral	ebi-a-GCST	oOqrag	440846	rs5586839	-0,04342	0,085771	0,612668
Plt	Mitral	ebi-a-GCST	oOqrag	440846	rs5587978	-0,03923	0,086203	0,649024
Plt	Mitral	ebi-a-GCST	oOqrag	440846	rs5604307	-0,03361	0,086795	0,698623
Plt	Mitral	ebi-a-GCST	oOqrag	440846	rs5608875	-0,03572	0,086	0,677925
Plt	Mitral	ebi-a-GCST	oOqrag	440846	rs5633697	-0,03532	0,085884	0,680848
Plt	Mitral	ebi-a-GCST	oOqrag	440846	rs5727348	-0,04015	0,086135	0,641141
Plt	Mitral	ebi-a-GCST	oOqrag	440846	rs5748672	-0,03493	0,086141	0,68513
Plt	Mitral	ebi-a-GCST	oOqrag	440846	rs5753146	-0,03787	0,086131	0,660166
Plt	Mitral	ebi-a-GCST	oOqrag	440846	rs5755699	-0,03604	0,086026	0,675239
Plt	Mitral	ebi-a-GCST	oOqrag	440846	rs5779596	-0,03931	0,086156	0,648185
Plt	Mitral	ebi-a-GCST	oOqrag	440846	rs5784363	-0,03229	0,086578	0,709185
Plt	Mitral	ebi-a-GCST	oOqrag	440846	rs5824161	-0,04188	0,086034	0,626444
Plt	Mitral	ebi-a-GCST	oOqrag	440846	rs583306	-0,03847	0,086186	0,655367
Plt	Mitral	ebi-a-GCST	oOqrag	440846	rs5872218	-0,04149	0,086145	0,630042
Plt	Mitral	ebi-a-GCST	oOqrag	440846	rs594942	-0,0389	0,08618	0,651693
Plt	Mitral	ebi-a-GCST	oOqrag	440846	rs5960233	-0,042	0,086115	0,625726
Plt	Mitral	ebi-a-GCST	oOqrag	440846	rs5978104	-0,03908	0,086162	0,650177
Plt	Mitral	ebi-a-GCST	oOqrag	440846	rs6001292	-0,04434	0,085663	0,604742
Plt	Mitral	ebi-a-GCST	oOqrag	440846	rs6002915	-0,03757	0,086124	0,662672
Plt	Mitral	ebi-a-GCST	oOqrag	440846	rs6017460	-0,0366	0,086032	0,670535
Plt	Mitral	ebi-a-GCST	oOqrag	440846	rs6018861	-0,03931	0,086155	0,648221
Plt	Mitral	ebi-a-GCST	oOqrag	440846	rs6035274	-0,0383	0,086157	0,65669
Plt	Mitral	ebi-a-GCST	oOqrag	440846	rs6038456	-0,03971	0,08619	0,645028
Plt	Mitral	ebi-a-GCST	oOqrag	440846	rs6045612	-0,03784	0,086325	0,661121
Plt	Mitral	ebi-a-GCST	oOqrag	440846	rs6060978	-0,0369	0,086186	0,668545
Plt	Mitral	ebi-a-GCST	oOqrag	440846	rs6066390	-0,03715	0,086088	0,666061
Plt	Mitral	ebi-a-GCST	oOqrag	440846	rs6074203	-0,03747	0,08609	0,66337
Plt	Mitral	ebi-a-GCST	oOqrag	440846	rs6075741	-0,03989	0,086318	0,643966
Plt	Mitral	ebi-a-GCST	oOqrag	440846	rs6102539	-0,0389	0,086165	0,651654
Plt	Mitral	ebi-a-GCST	oOqrag	440846	rs6103669	-0,03662	0,086086	0,670545
Plt	Mitral	ebi-a-GCST	oOqrag	440846	rs6126926	-0,03762	0,086108	0,662223
Plt	Mitral	ebi-a-GCST	oOqrag	440846	rs6140670	-0,04169	0,086018	0,627877
Plt	Mitral	ebi-a-GCST	oOqrag	440846	rs6141	-0,04752	0,086549	0,582964
Plt	Mitral	ebi-a-GCST	oOqrag	440846	rs6160828	-0,04285	0,085847	0,617658
Plt	Mitral	ebi-a-GCST	oOqrag	440846	rs6178129	-0,04265	0,086121	0,620456

Plt	Mitral	ebi-a-GCST	oOqrag	440846	rs6198659	-0,03846	0,086182	0,65542
Plt	Mitral	ebi-a-GCST	oOqrag	440846	rs6200231	-0,04479	0,085484	0,600339
Plt	Mitral	ebi-a-GCST	oOqrag	440846	rs6202286	-0,03962	0,08616	0,645668
Plt	Mitral	ebi-a-GCST	oOqrag	440846	rs6211396	-0,04066	0,086091	0,636689
Plt	Mitral	ebi-a-GCST	oOqrag	440846	rs6218391	-0,0401	0,086155	0,641615
Plt	Mitral	ebi-a-GCST	oOqrag	440846	rs6221779	-0,03355	0,085775	0,69567
Plt	Mitral	ebi-a-GCST	oOqrag	440846	rs6249138	-0,0369	0,086113	0,668285
Plt	Mitral	ebi-a-GCST	oOqrag	440846	rs6252377	-0,03926	0,08619	0,648749
Plt	Mitral	ebi-a-GCST	oOqrag	440846	rs630505	-0,03656	0,086069	0,671019
Plt	Mitral	ebi-a-GCST	oOqrag	440846	rs6420998	-0,04144	0,086154	0,630505
Plt	Mitral	ebi-a-GCST	oOqrag	440846	rs6435080	-0,0445	0,085851	0,604242
Plt	Mitral	ebi-a-GCST	oOqrag	440846	rs6439675	-0,03914	0,086181	0,649728
Plt	Mitral	ebi-a-GCST	oOqrag	440846	rs6444571	-0,03966	0,086183	0,645374
Plt	Mitral	ebi-a-GCST	oOqrag	440846	rs646809	-0,03915	0,086168	0,649563
Plt	Mitral	ebi-a-GCST	oOqrag	440846	rs648103	-0,03898	0,086174	0,650995
Plt	Mitral	ebi-a-GCST	oOqrag	440846	rs6537255	-0,03767	0,086158	0,661982
Plt	Mitral	ebi-a-GCST	oOqrag	440846	rs6545465	-0,04303	0,085823	0,616072
Plt	Mitral	ebi-a-GCST	oOqrag	440846	rs6546909	-0,04615	0,08549	0,589352
Plt	Mitral	ebi-a-GCST	oOqrag	440846	rs655029	-0,03256	0,0866	0,706949
Plt	Mitral	ebi-a-GCST	oOqrag	440846	rs6554595	-0,04239	0,085933	0,62183
Plt	Mitral	ebi-a-GCST	oOqrag	440846	rs6556405	-0,0369	0,086182	0,668508
Plt	Mitral	ebi-a-GCST	oOqrag	440846	rs656163	-0,03973	0,086139	0,644599
Plt	Mitral	ebi-a-GCST	oOqrag	440846	rs6572992	-0,03755	0,086119	0,662812
Plt	Mitral	ebi-a-GCST	oOqrag	440846	rs6588634	-0,03639	0,08603	0,672281
Plt	Mitral	ebi-a-GCST	oOqrag	440846	rs658974	-0,04171	0,086058	0,627903
Plt	Mitral	ebi-a-GCST	oOqrag	440846	rs6592965	-0,04302	0,085926	0,616595
Plt	Mitral	ebi-a-GCST	oOqrag	440846	rs6652794	-0,04064	0,086083	0,636875
Plt	Mitral	ebi-a-GCST	oOqrag	440846	rs6664992	-0,04182	0,085918	0,626462
Plt	Mitral	ebi-a-GCST	oOqrag	440846	rs669556	-0,03832	0,086172	0,656544
Plt	Mitral	ebi-a-GCST	oOqrag	440846	rs6696074	-0,03797	0,086147	0,65941
Plt	Mitral	ebi-a-GCST	oOqrag	440846	rs6706095	-0,04089	0,086153	0,635057
Plt	Mitral	ebi-a-GCST	oOqrag	440846	rs6714956	-0,03443	0,085931	0,688643
Plt	Mitral	ebi-a-GCST	oOqrag	440846	rs6815294	-0,04143	0,086147	0,630558
Plt	Mitral	ebi-a-GCST	oOqrag	440846	rs6863275	-0,03725	0,086096	0,665299
Plt	Mitral	ebi-a-GCST	oOqrag	440846	rs6880286	-0,03692	0,08614	0,668219
Plt	Mitral	ebi-a-GCST	oOqrag	440846	rs6883116	-0,0375	0,086099	0,663157
Plt	Mitral	ebi-a-GCST	oOqrag	440846	rs6883827	-0,03771	0,086233	0,661873
Plt	Mitral	ebi-a-GCST	oOqrag	440846	rs6985149	-0,03813	0,086171	0,658155
Plt	Mitral	ebi-a-GCST	oOqrag	440846	rs6993770	-0,04458	0,086643	0,606909
Plt	Mitral	ebi-a-GCST	oOqrag	440846	rs7010394	-0,04214	0,086058	0,624379
Plt	Mitral	ebi-a-GCST	oOqrag	440846	rs706025	-0,03826	0,086146	0,656937
Plt	Mitral	ebi-a-GCST	oOqrag	440846	rs7088799	-0,05324	0,086754	0,539407
Plt	Mitral	ebi-a-GCST	oOqrag	440846	rs7103603	-0,04262	0,086179	0,620888
Plt	Mitral	ebi-a-GCST	oOqrag	440846	rs7117878	-0,04448	0,085639	0,603466
Plt	Mitral	ebi-a-GCST	oOqrag	440846	rs7124681	-0,04047	0,086205	0,63876
Plt	Mitral	ebi-a-GCST	oOqrag	440846	rs7126519	-0,03878	0,086164	0,652652
Plt	Mitral	ebi-a-GCST	oOqrag	440846	rs7233932	-0,03639	0,08602	0,672257
Plt	Mitral	ebi-a-GCST	oOqrag	440846	rs7249692	-0,04171	0,086203	0,62845
Plt	Mitral	ebi-a-GCST	oOqrag	440846	rs7252328	-0,03853	0,086177	0,654776
Plt	Mitral	ebi-a-GCST	oOqrag	440846	rs7253820	-0,03948	0,0862	0,646963

Plt	Mitral	ebi-a-GCST	oOqrag	440846	rs7254827	-0,04805	0,085571	0,574421
Plt	Mitral	ebi-a-GCST	oOqrag	440846	rs7264883(	-0,03625	0,086042	0,673559
Plt	Mitral	ebi-a-GCST	oOqrag	440846	rs7268700(	-0,03876	0,086167	0,652856
Plt	Mitral	ebi-a-GCST	oOqrag	440846	rs7272515{	-0,03664	0,086288	0,671151
Plt	Mitral	ebi-a-GCST	oOqrag	440846	rs7276094(	-0,04065	0,086101	0,636825
Plt	Mitral	ebi-a-GCST	oOqrag	440846	rs7278164	-0,03885	0,086163	0,65211
Plt	Mitral	ebi-a-GCST	oOqrag	440846	rs7296982(	-0,03882	0,086185	0,652399
Plt	Mitral	ebi-a-GCST	oOqrag	440846	rs7303652(	-0,0416	0,086359	0,630007
Plt	Mitral	ebi-a-GCST	oOqrag	440846	rs7303718!	-0,04042	0,086106	0,638779
Plt	Mitral	ebi-a-GCST	oOqrag	440846	rs7314012!	-0,03668	0,085996	0,669693
Plt	Mitral	ebi-a-GCST	oOqrag	440846	rs7323160!	-0,04145	0,086019	0,629918
Plt	Mitral	ebi-a-GCST	oOqrag	440846	rs7332115	-0,03165	0,085874	0,712468
Plt	Mitral	ebi-a-GCST	oOqrag	440846	rs7385804	-0,04243	0,086157	0,622399
Plt	Mitral	ebi-a-GCST	oOqrag	440846	rs7388485(	-0,04076	0,086066	0,635783
Plt	Mitral	ebi-a-GCST	oOqrag	440846	rs7452899:	-0,04323	0,085662	0,613793
Plt	Mitral	ebi-a-GCST	oOqrag	440846	rs7456180(	-0,03611	0,086045	0,674693
Plt	Mitral	ebi-a-GCST	oOqrag	440846	rs7506560	-0,03791	0,086124	0,659846
Plt	Mitral	ebi-a-GCST	oOqrag	440846	rs7510779:	-0,04472	0,086551	0,605377
Plt	Mitral	ebi-a-GCST	oOqrag	440846	rs7516301	-0,03671	0,086103	0,66986
Plt	Mitral	ebi-a-GCST	oOqrag	440846	rs7565998	-0,03808	0,086146	0,658493
Plt	Mitral	ebi-a-GCST	oOqrag	440846	rs7585866	-0,04216	0,086053	0,624168
Plt	Mitral	ebi-a-GCST	oOqrag	440846	rs7606494	-0,04296	0,086013	0,617449
Plt	Mitral	ebi-a-GCST	oOqrag	440846	rs761841	-0,03997	0,086142	0,642632
Plt	Mitral	ebi-a-GCST	oOqrag	440846	rs7630419	-0,03793	0,086151	0,659703
Plt	Mitral	ebi-a-GCST	oOqrag	440846	rs7641325	-0,04084	0,086299	0,636056
Plt	Mitral	ebi-a-GCST	oOqrag	440846	rs7644537{	-0,04197	0,086165	0,626157
Plt	Mitral	ebi-a-GCST	oOqrag	440846	rs7649610!	-0,0417	0,086063	0,628048
Plt	Mitral	ebi-a-GCST	oOqrag	440846	rs7660913	-0,04082	0,086086	0,635356
Plt	Mitral	ebi-a-GCST	oOqrag	440846	rs7671752	-0,04044	0,086132	0,638744
Plt	Mitral	ebi-a-GCST	oOqrag	440846	rs7685966!	-0,03722	0,086086	0,665451
Plt	Mitral	ebi-a-GCST	oOqrag	440846	rs7705526	-0,04855	0,086123	0,572929
Plt	Mitral	ebi-a-GCST	oOqrag	440846	rs7733816(	-0,03937	0,086156	0,647676
Plt	Mitral	ebi-a-GCST	oOqrag	440846	rs7749401	-0,03764	0,08611	0,662042
Plt	Mitral	ebi-a-GCST	oOqrag	440846	rs7760090	-0,03631	0,086095	0,67324
Plt	Mitral	ebi-a-GCST	oOqrag	440846	rs777490	-0,03818	0,086148	0,657645
Plt	Mitral	ebi-a-GCST	oOqrag	440846	rs7780828:	-0,03651	0,086113	0,671585
Plt	Mitral	ebi-a-GCST	oOqrag	440846	rs7789916	-0,03738	0,086157	0,66442
Plt	Mitral	ebi-a-GCST	oOqrag	440846	rs7815724{	-0,03706	0,086092	0,666861
Plt	Mitral	ebi-a-GCST	oOqrag	440846	rs7826103	-0,04141	0,086043	0,630301
Plt	Mitral	ebi-a-GCST	oOqrag	440846	rs7830416	-0,03649	0,086026	0,671445
Plt	Mitral	ebi-a-GCST	oOqrag	440846	rs7833924	-0,0506	0,086054	0,556512
Plt	Mitral	ebi-a-GCST	oOqrag	440846	rs7837624:	-0,04218	0,085966	0,6237
Plt	Mitral	ebi-a-GCST	oOqrag	440846	rs7843024	-0,04356	0,085831	0,611824
Plt	Mitral	ebi-a-GCST	oOqrag	440846	rs7851531	-0,0397	0,086141	0,644885
Plt	Mitral	ebi-a-GCST	oOqrag	440846	rs7890903:	-0,02935	0,086177	0,733427
Plt	Mitral	ebi-a-GCST	oOqrag	440846	rs7900750	-0,04122	0,086288	0,632892
Plt	Mitral	ebi-a-GCST	oOqrag	440846	rs7912685	-0,03524	0,085913	0,681643
Plt	Mitral	ebi-a-GCST	oOqrag	440846	rs7920539	-0,03973	0,086148	0,644635
Plt	Mitral	ebi-a-GCST	oOqrag	440846	rs7937770	-0,03831	0,086155	0,656523
Plt	Mitral	ebi-a-GCST	oOqrag	440846	rs7954157{	-0,04207	0,086038	0,624886

Plt	Mitral	ebi-a-GCST	oOqrag	440846	rs796004	-0,03889	0,086162	0,65169
Plt	Mitral	ebi-a-GCST	oOqrag	440846	rs7971658	-0,03833	0,08616	0,656376
Plt	Mitral	ebi-a-GCST	oOqrag	440846	rs8001273	-0,03245	0,085834	0,705376
Plt	Mitral	ebi-a-GCST	oOqrag	440846	rs8005417	-0,03243	0,08622	0,706814
Plt	Mitral	ebi-a-GCST	oOqrag	440846	rs8014303	-0,04108	0,086353	0,634297
Plt	Mitral	ebi-a-GCST	oOqrag	440846	rs8049394	-0,03729	0,086159	0,665167
Plt	Mitral	ebi-a-GCST	oOqrag	440846	rs8050500	-0,03982	0,086172	0,64402
Plt	Mitral	ebi-a-GCST	oOqrag	440846	rs8056774	-0,03836	0,086151	0,656126
Plt	Mitral	ebi-a-GCST	oOqrag	440846	rs8071834	-0,04005	0,086132	0,641956
Plt	Mitral	ebi-a-GCST	oOqrag	440846	rs8072157	-0,03472	0,085902	0,686113
Plt	Mitral	ebi-a-GCST	oOqrag	440846	rs8098454	-0,04394	0,08569	0,608104
Plt	Mitral	ebi-a-GCST	oOqrag	440846	rs8106212	-0,0389	0,086191	0,651716
Plt	Mitral	ebi-a-GCST	oOqrag	440846	rs8129054	-0,04101	0,086124	0,63396
Plt	Mitral	ebi-a-GCST	oOqrag	440846	rs8178824	-0,03548	0,086254	0,680807
Plt	Mitral	ebi-a-GCST	oOqrag	440846	rs850736	-0,03219	0,086218	0,708851
Plt	Mitral	ebi-a-GCST	oOqrag	440846	rs855791	-0,04232	0,086177	0,623402
Plt	Mitral	ebi-a-GCST	oOqrag	440846	rs858516	-0,03736	0,086135	0,664453
Plt	Mitral	ebi-a-GCST	oOqrag	440846	rs882405	-0,03617	0,086176	0,674708
Plt	Mitral	ebi-a-GCST	oOqrag	440846	rs896156	-0,03731	0,086163	0,665033
Plt	Mitral	ebi-a-GCST	oOqrag	440846	rs9264781	-0,03846	0,086151	0,655272
Plt	Mitral	ebi-a-GCST	oOqrag	440846	rs9266658	-0,02694	0,086093	0,754334
Plt	Mitral	ebi-a-GCST	oOqrag	440846	rs9294458	-0,04085	0,086057	0,635004
Plt	Mitral	ebi-a-GCST	oOqrag	440846	rs9299006	-0,04208	0,086061	0,624908
Plt	Mitral	ebi-a-GCST	oOqrag	440846	rs9371672	-0,03959	0,08615	0,645874
Plt	Mitral	ebi-a-GCST	oOqrag	440846	rs9376060	-0,04154	0,086202	0,629916
Plt	Mitral	ebi-a-GCST	oOqrag	440846	rs9382146	-0,03933	0,086222	0,648294
Plt	Mitral	ebi-a-GCST	oOqrag	440846	rs9400379	-0,03733	0,086156	0,664781
Plt	Mitral	ebi-a-GCST	oOqrag	440846	rs9410195	-0,03986	0,086264	0,644047
Plt	Mitral	ebi-a-GCST	oOqrag	440846	rs9429767	-0,03619	0,086039	0,674047
Plt	Mitral	ebi-a-GCST	oOqrag	440846	rs949787	-0,03552	0,085931	0,679365
Plt	Mitral	ebi-a-GCST	oOqrag	440846	rs950965	-0,03938	0,086203	0,647783
Plt	Mitral	ebi-a-GCST	oOqrag	440846	rs9535495	-0,03903	0,086164	0,650544
Plt	Mitral	ebi-a-GCST	oOqrag	440846	rs963997	-0,03984	0,086142	0,643719
Plt	Mitral	ebi-a-GCST	oOqrag	440846	rs964184	-0,03521	0,086131	0,682705
Plt	Mitral	ebi-a-GCST	oOqrag	440846	rs9704476	-0,04081	0,086109	0,635577
Plt	Mitral	ebi-a-GCST	oOqrag	440846	rs9809116	-0,04176	0,086107	0,627656
Plt	Mitral	ebi-a-GCST	oOqrag	440846	rs9829114	-0,04098	0,086172	0,63439
Plt	Mitral	ebi-a-GCST	oOqrag	440846	rs9843122	-0,03939	0,086155	0,647565
Plt	Mitral	ebi-a-GCST	oOqrag	440846	rs9879207	-0,03884	0,086161	0,652185
Plt	Mitral	ebi-a-GCST	oOqrag	440846	rs9916458	-0,03655	0,086124	0,671283
Plt	Mitral	ebi-a-GCST	oOqrag	440846	rs9919955	-0,04054	0,086182	0,638092
Plt	Mitral	ebi-a-GCST	oOqrag	440846	rs9947269	-0,04254	0,086083	0,621199
Plt	Mitral	ebi-a-GCST	oOqrag	440846	rs9950562	-0,03798	0,08615	0,65928
Plt	Mitral	ebi-a-GCST	oOqrag	440846	All	-0,03898	0,08605	0,650548
RBC	Mitral	ebi-a-GCST	SQMtTf	453596	rs1016273	0,089376	0,105155	0,395357
RBC	Mitral	ebi-a-GCST	SQMtTf	453596	rs1016834	0,108239	0,105931	0,306878
RBC	Mitral	ebi-a-GCST	SQMtTf	453596	rs1021370	0,084138	0,105184	0,423758
RBC	Mitral	ebi-a-GCST	SQMtTf	453596	rs1026522	0,094623	0,106013	0,372089
RBC	Mitral	ebi-a-GCST	SQMtTf	453596	rs1037117	0,089506	0,105164	0,394706
RBC	Mitral	ebi-a-GCST	SQMtTf	453596	rs1043356	0,083374	0,105243	0,428241

RBC	Mitral	ebi-a-GCST	SQMtTf	453596	rs1047891	0,088133	0,105287	0,402551
RBC	Mitral	ebi-a-GCST	SQMtTf	453596	rs1048675	0,089911	0,105165	0,392577
RBC	Mitral	ebi-a-GCST	SQMtTf	453596	rs1049496	0,085045	0,105334	0,419446
RBC	Mitral	ebi-a-GCST	SQMtTf	453596	rs1075818	0,084156	0,105176	0,423625
RBC	Mitral	ebi-a-GCST	SQMtTf	453596	rs1076266	0,087136	0,105292	0,407915
RBC	Mitral	ebi-a-GCST	SQMtTf	453596	rs1076467	0,086776	0,105294	0,409862
RBC	Mitral	ebi-a-GCST	SQMtTf	453596	rs1078655	0,088954	0,105302	0,39825
RBC	Mitral	ebi-a-GCST	SQMtTf	453596	rs1079782	0,087772	0,105283	0,404463
RBC	Mitral	ebi-a-GCST	SQMtTf	453596	rs1088783	0,087085	0,10529	0,408185
RBC	Mitral	ebi-a-GCST	SQMtTf	453596	rs1089027	0,091426	0,105122	0,384457
RBC	Mitral	ebi-a-GCST	SQMtTf	453596	rs1091047	0,086823	0,105337	0,409805
RBC	Mitral	ebi-a-GCST	SQMtTf	453596	rs1095693	0,085016	0,105303	0,419469
RBC	Mitral	ebi-a-GCST	SQMtTf	453596	rs1105637	0,087009	0,105301	0,408638
RBC	Mitral	ebi-a-GCST	SQMtTf	453596	rs1106836	0,086937	0,105297	0,409008
RBC	Mitral	ebi-a-GCST	SQMtTf	453596	rs1107480	0,084656	0,105309	0,421468
RBC	Mitral	ebi-a-GCST	SQMtTf	453596	rs1109909	0,08087	0,105242	0,442238
RBC	Mitral	ebi-a-GCST	SQMtTf	453596	rs1110078	0,092384	0,104743	0,377776
RBC	Mitral	ebi-a-GCST	SQMtTf	453596	rs1118798	0,089606	0,105192	0,39431
RBC	Mitral	ebi-a-GCST	SQMtTf	453596	rs1119151	0,083734	0,105264	0,426345
RBC	Mitral	ebi-a-GCST	SQMtTf	453596	rs1122929	0,082802	0,10517	0,431097
RBC	Mitral	ebi-a-GCST	SQMtTf	453596	rs1124140	0,084673	0,10529	0,42129
RBC	Mitral	ebi-a-GCST	SQMtTf	453596	rs1124545	0,0911	0,105025	0,385713
RBC	Mitral	ebi-a-GCST	SQMtTf	453596	rs1125139	0,081364	0,105006	0,438425
RBC	Mitral	ebi-a-GCST	SQMtTf	453596	rs1125542	0,084645	0,10522	0,421131
RBC	Mitral	ebi-a-GCST	SQMtTf	453596	rs1126531	0,093051	0,104611	0,373734
RBC	Mitral	ebi-a-GCST	SQMtTf	453596	rs1135263	0,090034	0,105092	0,3916
RBC	Mitral	ebi-a-GCST	SQMtTf	453596	rs1153584	0,086363	0,105336	0,412287
RBC	Mitral	ebi-a-GCST	SQMtTf	453596	rs1154515	0,089258	0,105245	0,396381
RBC	Mitral	ebi-a-GCST	SQMtTf	453596	rs1159862	0,091526	0,105328	0,384868
RBC	Mitral	ebi-a-GCST	SQMtTf	453596	rs1160198	0,081575	0,105137	0,437814
RBC	Mitral	ebi-a-GCST	SQMtTf	453596	rs1162575	0,083234	0,105086	0,428327
RBC	Mitral	ebi-a-GCST	SQMtTf	453596	rs1162827	0,088994	0,10541	0,39852
RBC	Mitral	ebi-a-GCST	SQMtTf	453596	rs1167749	0,08444	0,105257	0,422421
RBC	Mitral	ebi-a-GCST	SQMtTf	453596	rs1169718	0,088233	0,105341	0,402256
RBC	Mitral	ebi-a-GCST	SQMtTf	453596	rs1170631	0,086945	0,105299	0,408979
RBC	Mitral	ebi-a-GCST	SQMtTf	453596	rs1172001	0,090119	0,105103	0,391207
RBC	Mitral	ebi-a-GCST	SQMtTf	453596	rs1172337	0,085597	0,105302	0,41629
RBC	Mitral	ebi-a-GCST	SQMtTf	453596	rs1175550	0,092813	0,1052	0,37764
RBC	Mitral	ebi-a-GCST	SQMtTf	453596	rs1175834	0,086088	0,105284	0,413547
RBC	Mitral	ebi-a-GCST	SQMtTf	453596	rs1176520	0,093504	0,104969	0,373049
RBC	Mitral	ebi-a-GCST	SQMtTf	453596	rs1190139	0,084641	0,105247	0,421274
RBC	Mitral	ebi-a-GCST	SQMtTf	453596	rs1205088	0,084377	0,105308	0,422991
RBC	Mitral	ebi-a-GCST	SQMtTf	453596	rs1212819	0,080678	0,104697	0,440956
RBC	Mitral	ebi-a-GCST	SQMtTf	453596	rs1213357	0,081624	0,104963	0,436778
RBC	Mitral	ebi-a-GCST	SQMtTf	453596	rs1215161	0,092189	0,105305	0,381333
RBC	Mitral	ebi-a-GCST	SQMtTf	453596	rs1215503	0,089717	0,10543	0,394789
RBC	Mitral	ebi-a-GCST	SQMtTf	453596	rs1220935	0,091385	0,105117	0,384648
RBC	Mitral	ebi-a-GCST	SQMtTf	453596	rs1233878	0,08016	0,104911	0,444821
RBC	Mitral	ebi-a-GCST	SQMtTf	453596	rs123698	0,090024	0,1053	0,392587
RBC	Mitral	ebi-a-GCST	SQMtTf	453596	rs1247383	0,089925	0,105218	0,39274

RBC	Mitral	ebi-a-GCST	SQMtTf	453596	rs1249993ℓ	0,0796	0,104932	0,448099
RBC	Mitral	ebi-a-GCST	SQMtTf	453596	rs1250561(	0,090507	0,105459	0,390773
RBC	Mitral	ebi-a-GCST	SQMtTf	453596	rs1251288:	0,090388	0,105272	0,390553
RBC	Mitral	ebi-a-GCST	SQMtTf	453596	rs1256061	0,076891	0,104816	0,463208
RBC	Mitral	ebi-a-GCST	SQMtTf	453596	rs1257415	0,087889	0,10531	0,403956
RBC	Mitral	ebi-a-GCST	SQMtTf	453596	rs1260201(	0,087813	0,105368	0,404623
RBC	Mitral	ebi-a-GCST	SQMtTf	453596	rs1260789ℓ	0,079852	0,105369	0,448551
RBC	Mitral	ebi-a-GCST	SQMtTf	453596	rs1270612:	0,08436	0,105211	0,422661
RBC	Mitral	ebi-a-GCST	SQMtTf	453596	rs1273065:	0,08927	0,105178	0,39602
RBC	Mitral	ebi-a-GCST	SQMtTf	453596	rs1277725ℓ	0,086373	0,1057	0,413844
RBC	Mitral	ebi-a-GCST	SQMtTf	453596	rs1278339(	0,093744	0,104939	0,371683
RBC	Mitral	ebi-a-GCST	SQMtTf	453596	rs1281151:	0,086535	0,105366	0,411485
RBC	Mitral	ebi-a-GCST	SQMtTf	453596	rs128494	0,086705	0,105413	0,410781
RBC	Mitral	ebi-a-GCST	SQMtTf	453596	rs1288186!	0,089728	0,105254	0,393944
RBC	Mitral	ebi-a-GCST	SQMtTf	453596	rs1288587ℓ	0,096192	0,10458	0,357682
RBC	Mitral	ebi-a-GCST	SQMtTf	453596	rs1288926:	0,083782	0,10523	0,425927
RBC	Mitral	ebi-a-GCST	SQMtTf	453596	rs1289741⁄	0,094947	0,104574	0,36391
RBC	Mitral	ebi-a-GCST	SQMtTf	453596	rs1294098:	0,079353	0,104988	0,449752
RBC	Mitral	ebi-a-GCST	SQMtTf	453596	rs1296871!	0,085841	0,105299	0,414949
RBC	Mitral	ebi-a-GCST	SQMtTf	453596	rs1298796:	0,085371	0,105272	0,41739
RBC	Mitral	ebi-a-GCST	SQMtTf	453596	rs1302896ℓ	0,090939	0,105088	0,386843
RBC	Mitral	ebi-a-GCST	SQMtTf	453596	rs1310732!	0,081333	0,105027	0,438694
RBC	Mitral	ebi-a-GCST	SQMtTf	453596	rs1314635!	0,085894	0,105437	0,415273
RBC	Mitral	ebi-a-GCST	SQMtTf	453596	rs1316542⁄	0,092063	0,105194	0,381479
RBC	Mitral	ebi-a-GCST	SQMtTf	453596	rs1332243!	0,088227	0,105265	0,401952
RBC	Mitral	ebi-a-GCST	SQMtTf	453596	rs1338921!	0,088468	0,105299	0,400821
RBC	Mitral	ebi-a-GCST	SQMtTf	453596	rs1394090!	0,085024	0,105357	0,419662
RBC	Mitral	ebi-a-GCST	SQMtTf	453596	rs1394224!	0,083609	0,105278	0,427093
RBC	Mitral	ebi-a-GCST	SQMtTf	453596	rs1404256(	0,092673	0,105071	0,377777
RBC	Mitral	ebi-a-GCST	SQMtTf	453596	rs1405077!	0,083539	0,105241	0,427318
RBC	Mitral	ebi-a-GCST	SQMtTf	453596	rs140522	0,084851	0,105935	0,423148
RBC	Mitral	ebi-a-GCST	SQMtTf	453596	rs1413611	0,088727	0,105266	0,399292
RBC	Mitral	ebi-a-GCST	SQMtTf	453596	rs1426374	0,091353	0,105093	0,384707
RBC	Mitral	ebi-a-GCST	SQMtTf	453596	rs1427445	0,093049	0,105334	0,377039
RBC	Mitral	ebi-a-GCST	SQMtTf	453596	rs1434282	0,082817	0,105647	0,433096
RBC	Mitral	ebi-a-GCST	SQMtTf	453596	rs1469710	0,087674	0,105283	0,404992
RBC	Mitral	ebi-a-GCST	SQMtTf	453596	rs1479559	0,083473	0,10522	0,427594
RBC	Mitral	ebi-a-GCST	SQMtTf	453596	rs1480839⁄	0,09096	0,105205	0,387257
RBC	Mitral	ebi-a-GCST	SQMtTf	453596	rs1481028(	0,091081	0,105077	0,386051
RBC	Mitral	ebi-a-GCST	SQMtTf	453596	rs1482941	0,094146	0,104697	0,368532
RBC	Mitral	ebi-a-GCST	SQMtTf	453596	rs1486140ℓ	0,082854	0,105319	0,431459
RBC	Mitral	ebi-a-GCST	SQMtTf	453596	rs1496354	0,086429	0,105323	0,411867
RBC	Mitral	ebi-a-GCST	SQMtTf	453596	rs1546723	0,082938	0,106119	0,434475
RBC	Mitral	ebi-a-GCST	SQMtTf	453596	rs1555405	0,090641	0,105063	0,388288
RBC	Mitral	ebi-a-GCST	SQMtTf	453596	rs1569419	0,088934	0,105345	0,398548
RBC	Mitral	ebi-a-GCST	SQMtTf	453596	rs159058	0,087386	0,105352	0,40684
RBC	Mitral	ebi-a-GCST	SQMtTf	453596	rs1629862	0,092465	0,104999	0,378519
RBC	Mitral	ebi-a-GCST	SQMtTf	453596	rs163790	0,085619	0,105277	0,41606
RBC	Mitral	ebi-a-GCST	SQMtTf	453596	rs1691664:	0,087996	0,105301	0,403343
RBC	Mitral	ebi-a-GCST	SQMtTf	453596	rs1694275:	0,093522	0,104386	0,370293

RBC	Mitral	ebi-a-GCST	SQMtTf	453596	rs1696807	0,091639	0,105056	0,383052
RBC	Mitral	ebi-a-GCST	SQMtTf	453596	rs1703045	0,084913	0,105384	0,420387
RBC	Mitral	ebi-a-GCST	SQMtTf	453596	rs1704577	0,086609	0,105351	0,411019
RBC	Mitral	ebi-a-GCST	SQMtTf	453596	rs1711638	0,083476	0,105259	0,427744
RBC	Mitral	ebi-a-GCST	SQMtTf	453596	rs1715441	0,084546	0,10525	0,421807
RBC	Mitral	ebi-a-GCST	SQMtTf	453596	rs174574	0,087322	0,105528	0,40797
RBC	Mitral	ebi-a-GCST	SQMtTf	453596	rs1747636	0,102311	0,105634	0,332774
RBC	Mitral	ebi-a-GCST	SQMtTf	453596	rs1748148	0,090122	0,105251	0,391852
RBC	Mitral	ebi-a-GCST	SQMtTf	453596	rs1771647	0,087218	0,105362	0,407787
RBC	Mitral	ebi-a-GCST	SQMtTf	453596	rs1775869	0,0837	0,105424	0,427234
RBC	Mitral	ebi-a-GCST	SQMtTf	453596	rs1775935	0,089856	0,105177	0,392922
RBC	Mitral	ebi-a-GCST	SQMtTf	453596	rs1781669	0,084542	0,10528	0,421962
RBC	Mitral	ebi-a-GCST	SQMtTf	453596	rs1799899	0,081881	0,104972	0,435375
RBC	Mitral	ebi-a-GCST	SQMtTf	453596	rs180223	0,091443	0,104917	0,38344
RBC	Mitral	ebi-a-GCST	SQMtTf	453596	rs1826847	0,082016	0,105068	0,435036
RBC	Mitral	ebi-a-GCST	SQMtTf	453596	rs1863127	0,089222	0,105299	0,396816
RBC	Mitral	ebi-a-GCST	SQMtTf	453596	rs1865637	0,084315	0,105212	0,422912
RBC	Mitral	ebi-a-GCST	SQMtTf	453596	rs1868274	0,078904	0,105134	0,452949
RBC	Mitral	ebi-a-GCST	SQMtTf	453596	rs1874228	0,082328	0,105366	0,434597
RBC	Mitral	ebi-a-GCST	SQMtTf	453596	rs1891120	0,086274	0,105294	0,412581
RBC	Mitral	ebi-a-GCST	SQMtTf	453596	rs1903879	0,085246	0,105237	0,417919
RBC	Mitral	ebi-a-GCST	SQMtTf	453596	rs1940751	0,087242	0,105373	0,407707
RBC	Mitral	ebi-a-GCST	SQMtTf	453596	rs1966228	0,081268	0,105083	0,439304
RBC	Mitral	ebi-a-GCST	SQMtTf	453596	rs1978154	0,083491	0,105099	0,426962
RBC	Mitral	ebi-a-GCST	SQMtTf	453596	rs1987070	0,085876	0,105318	0,414846
RBC	Mitral	ebi-a-GCST	SQMtTf	453596	rs198833	0,087045	0,10535	0,408668
RBC	Mitral	ebi-a-GCST	SQMtTf	453596	rs2001735	0,086607	0,105295	0,410781
RBC	Mitral	ebi-a-GCST	SQMtTf	453596	rs2007471	0,074952	0,104549	0,473428
RBC	Mitral	ebi-a-GCST	SQMtTf	453596	rs2008883	0,08143	0,105559	0,440456
RBC	Mitral	ebi-a-GCST	SQMtTf	453596	rs2015803	0,090282	0,105055	0,390131
RBC	Mitral	ebi-a-GCST	SQMtTf	453596	rs2020204	0,088485	0,105259	0,400549
RBC	Mitral	ebi-a-GCST	SQMtTf	453596	rs2033878	0,088808	0,105217	0,398644
RBC	Mitral	ebi-a-GCST	SQMtTf	453596	rs2067663	0,085897	0,105409	0,415134
RBC	Mitral	ebi-a-GCST	SQMtTf	453596	rs2071243	0,082279	0,105174	0,434027
RBC	Mitral	ebi-a-GCST	SQMtTf	453596	rs2075672	0,108523	0,106573	0,308537
RBC	Mitral	ebi-a-GCST	SQMtTf	453596	rs2085600	0,079192	0,104734	0,449575
RBC	Mitral	ebi-a-GCST	SQMtTf	453596	rs2089111	0,093355	0,104753	0,372825
RBC	Mitral	ebi-a-GCST	SQMtTf	453596	rs2111048	0,0845	0,105263	0,422116
RBC	Mitral	ebi-a-GCST	SQMtTf	453596	rs217184	0,092805	0,105041	0,376958
RBC	Mitral	ebi-a-GCST	SQMtTf	453596	rs218264	0,102719	0,107211	0,33801
RBC	Mitral	ebi-a-GCST	SQMtTf	453596	rs2186037	0,088016	0,105389	0,403634
RBC	Mitral	ebi-a-GCST	SQMtTf	453596	rs2209098	0,084247	0,105306	0,423702
RBC	Mitral	ebi-a-GCST	SQMtTf	453596	rs2230278	0,08616	0,105296	0,413204
RBC	Mitral	ebi-a-GCST	SQMtTf	453596	rs2255767	0,092347	0,105098	0,379577
RBC	Mitral	ebi-a-GCST	SQMtTf	453596	rs2273799	0,081772	0,105337	0,437578
RBC	Mitral	ebi-a-GCST	SQMtTf	453596	rs2309753	0,087967	0,105327	0,40362
RBC	Mitral	ebi-a-GCST	SQMtTf	453596	rs2343596	0,084547	0,105223	0,421685
RBC	Mitral	ebi-a-GCST	SQMtTf	453596	rs2394186	0,089538	0,105224	0,394808
RBC	Mitral	ebi-a-GCST	SQMtTf	453596	rs240051	0,083394	0,105167	0,427796
RBC	Mitral	ebi-a-GCST	SQMtTf	453596	rs2415121	0,081273	0,105134	0,439498

RBC	Mitral	ebi-a-GCST	SQMtTf	453596	rs2465405	0,083492	0,105156	0,427205
RBC	Mitral	ebi-a-GCST	SQMtTf	453596	rs2541639	0,084117	0,1054	0,424828
RBC	Mitral	ebi-a-GCST	SQMtTf	453596	rs2568844	0,090346	0,105108	0,390036
RBC	Mitral	ebi-a-GCST	SQMtTf	453596	rs2584641	0,086654	0,105311	0,410596
RBC	Mitral	ebi-a-GCST	SQMtTf	453596	rs261290	0,084571	0,105263	0,421732
RBC	Mitral	ebi-a-GCST	SQMtTf	453596	rs2629452	0,083814	0,105147	0,425386
RBC	Mitral	ebi-a-GCST	SQMtTf	453596	rs2661794	0,087554	0,105313	0,405761
RBC	Mitral	ebi-a-GCST	SQMtTf	453596	rs270981	0,084261	0,105182	0,423078
RBC	Mitral	ebi-a-GCST	SQMtTf	453596	rs2749790	0,086998	0,105315	0,408761
RBC	Mitral	ebi-a-GCST	SQMtTf	453596	rs2792561	0,089602	0,105149	0,394137
RBC	Mitral	ebi-a-GCST	SQMtTf	453596	rs279613	0,087925	0,105267	0,403577
RBC	Mitral	ebi-a-GCST	SQMtTf	453596	rs2808607	0,087927	0,105386	0,404094
RBC	Mitral	ebi-a-GCST	SQMtTf	453596	rs2823139	0,082433	0,105286	0,43366
RBC	Mitral	ebi-a-GCST	SQMtTf	453596	rs2823270	0,087568	0,105362	0,405909
RBC	Mitral	ebi-a-GCST	SQMtTf	453596	rs2834253	0,082077	0,105186	0,435211
RBC	Mitral	ebi-a-GCST	SQMtTf	453596	rs2835435	0,089554	0,105354	0,395307
RBC	Mitral	ebi-a-GCST	SQMtTf	453596	rs2841892	0,083477	0,105201	0,427484
RBC	Mitral	ebi-a-GCST	SQMtTf	453596	rs2843436	0,08511	0,105418	0,419461
RBC	Mitral	ebi-a-GCST	SQMtTf	453596	rs2849687	0,096312	0,104865	0,358389
RBC	Mitral	ebi-a-GCST	SQMtTf	453596	rs2854159	0,085475	0,105349	0,417168
RBC	Mitral	ebi-a-GCST	SQMtTf	453596	rs2854913	0,083995	0,105164	0,424461
RBC	Mitral	ebi-a-GCST	SQMtTf	453596	rs2860300	0,088282	0,105261	0,40164
RBC	Mitral	ebi-a-GCST	SQMtTf	453596	rs2862189	0,087885	0,105276	0,403827
RBC	Mitral	ebi-a-GCST	SQMtTf	453596	rs2865376	0,088608	0,105337	0,400246
RBC	Mitral	ebi-a-GCST	SQMtTf	453596	rs2876262	0,089633	0,105361	0,394924
RBC	Mitral	ebi-a-GCST	SQMtTf	453596	rs2888630	0,084584	0,105298	0,42181
RBC	Mitral	ebi-a-GCST	SQMtTf	453596	rs2923105	0,084573	0,105209	0,421482
RBC	Mitral	ebi-a-GCST	SQMtTf	453596	rs2941465	0,083857	0,105183	0,425305
RBC	Mitral	ebi-a-GCST	SQMtTf	453596	rs2943636	0,085943	0,105317	0,414475
RBC	Mitral	ebi-a-GCST	SQMtTf	453596	rs2970876	0,090268	0,105054	0,390197
RBC	Mitral	ebi-a-GCST	SQMtTf	453596	rs3025009	0,082898	0,105368	0,43143
RBC	Mitral	ebi-a-GCST	SQMtTf	453596	rs3127239	0,090048	0,105097	0,391548
RBC	Mitral	ebi-a-GCST	SQMtTf	453596	rs3184504	0,08794	0,105821	0,405957
RBC	Mitral	ebi-a-GCST	SQMtTf	453596	rs3458748	0,087092	0,105307	0,408218
RBC	Mitral	ebi-a-GCST	SQMtTf	453596	rs3493611	0,086825	0,105294	0,409603
RBC	Mitral	ebi-a-GCST	SQMtTf	453596	rs3500360	0,072415	0,104628	0,488861
RBC	Mitral	ebi-a-GCST	SQMtTf	453596	rs3504203	0,086133	0,105344	0,413565
RBC	Mitral	ebi-a-GCST	SQMtTf	453596	rs3512440	0,087825	0,105453	0,404939
RBC	Mitral	ebi-a-GCST	SQMtTf	453596	rs3524099	0,081816	0,105272	0,43705
RBC	Mitral	ebi-a-GCST	SQMtTf	453596	rs354547	0,09158	0,105154	0,383803
RBC	Mitral	ebi-a-GCST	SQMtTf	453596	rs357282	0,083169	0,105209	0,429227
RBC	Mitral	ebi-a-GCST	SQMtTf	453596	rs3576341	0,091317	0,105079	0,384829
RBC	Mitral	ebi-a-GCST	SQMtTf	453596	rs3593621	0,082777	0,105187	0,431307
RBC	Mitral	ebi-a-GCST	SQMtTf	453596	rs360017	0,093902	0,104474	0,368754
RBC	Mitral	ebi-a-GCST	SQMtTf	453596	rs3702477	0,087161	0,105275	0,407708
RBC	Mitral	ebi-a-GCST	SQMtTf	453596	rs3751305	0,086081	0,105295	0,413628
RBC	Mitral	ebi-a-GCST	SQMtTf	453596	rs3767844	0,08115	0,105442	0,441527
RBC	Mitral	ebi-a-GCST	SQMtTf	453596	rs3780474	0,08454	0,105324	0,422167
RBC	Mitral	ebi-a-GCST	SQMtTf	453596	rs3794809	0,086001	0,105285	0,41402
RBC	Mitral	ebi-a-GCST	SQMtTf	453596	rs3809627	0,082412	0,105727	0,435701

RBC	Mitral	ebi-a-GCST	SQMtTf	453596	rs3811444	0,087371	0,105675	0,408356
RBC	Mitral	ebi-a-GCST	SQMtTf	453596	rs381500	0,09608	0,105297	0,361527
RBC	Mitral	ebi-a-GCST	SQMtTf	453596	rs3824430	0,084292	0,105764	0,42546
RBC	Mitral	ebi-a-GCST	SQMtTf	453596	rs3879293	0,080275	0,104921	0,444214
RBC	Mitral	ebi-a-GCST	SQMtTf	453596	rs3943540	0,081036	0,105185	0,441053
RBC	Mitral	ebi-a-GCST	SQMtTf	453596	rs4008353	0,086036	0,105339	0,414067
RBC	Mitral	ebi-a-GCST	SQMtTf	453596	rs4075958	0,086577	0,105402	0,411415
RBC	Mitral	ebi-a-GCST	SQMtTf	453596	rs4138134	0,084796	0,105291	0,420618
RBC	Mitral	ebi-a-GCST	SQMtTf	453596	rs4144866	0,086102	0,10529	0,413496
RBC	Mitral	ebi-a-GCST	SQMtTf	453596	rs4240624	0,091015	0,105081	0,386416
RBC	Mitral	ebi-a-GCST	SQMtTf	453596	rs4242906	0,082288	0,105266	0,434379
RBC	Mitral	ebi-a-GCST	SQMtTf	453596	rs4255510	0,074244	0,104694	0,478232
RBC	Mitral	ebi-a-GCST	SQMtTf	453596	rs4305701	0,086585	0,105292	0,410888
RBC	Mitral	ebi-a-GCST	SQMtTf	453596	rs4368548	0,084035	0,105181	0,424319
RBC	Mitral	ebi-a-GCST	SQMtTf	453596	rs4447863	0,086594	0,105293	0,410845
RBC	Mitral	ebi-a-GCST	SQMtTf	453596	rs4479574	0,088217	0,105264	0,402002
RBC	Mitral	ebi-a-GCST	SQMtTf	453596	rs451157	0,086698	0,10531	0,410357
RBC	Mitral	ebi-a-GCST	SQMtTf	453596	rs4535497	0,08743	0,10539	0,406772
RBC	Mitral	ebi-a-GCST	SQMtTf	453596	rs464063	0,085893	0,105296	0,414654
RBC	Mitral	ebi-a-GCST	SQMtTf	453596	rs4655580	0,086702	0,105295	0,410267
RBC	Mitral	ebi-a-GCST	SQMtTf	453596	rs4663199	0,086264	0,105303	0,41267
RBC	Mitral	ebi-a-GCST	SQMtTf	453596	rs4669306	0,083065	0,105253	0,429997
RBC	Mitral	ebi-a-GCST	SQMtTf	453596	rs4729523	0,087656	0,105275	0,405049
RBC	Mitral	ebi-a-GCST	SQMtTf	453596	rs4743150	0,083528	0,105358	0,427898
RBC	Mitral	ebi-a-GCST	SQMtTf	453596	rs4760682	0,097798	0,104975	0,351526
RBC	Mitral	ebi-a-GCST	SQMtTf	453596	rs477992	0,084263	0,105336	0,423739
RBC	Mitral	ebi-a-GCST	SQMtTf	453596	rs4792687	0,085625	0,105348	0,416343
RBC	Mitral	ebi-a-GCST	SQMtTf	453596	rs4810479	0,086164	0,105311	0,413249
RBC	Mitral	ebi-a-GCST	SQMtTf	453596	rs4820405	0,085455	0,105311	0,417107
RBC	Mitral	ebi-a-GCST	SQMtTf	453596	rs4886755	0,08206	0,105435	0,436388
RBC	Mitral	ebi-a-GCST	SQMtTf	453596	rs4930077	0,090967	0,105106	0,386778
RBC	Mitral	ebi-a-GCST	SQMtTf	453596	rs495261	0,084424	0,105206	0,422285
RBC	Mitral	ebi-a-GCST	SQMtTf	453596	rs4985152	0,088934	0,105198	0,397893
RBC	Mitral	ebi-a-GCST	SQMtTf	453596	rs5004355	0,083845	0,105217	0,425525
RBC	Mitral	ebi-a-GCST	SQMtTf	453596	rs520000	0,086051	0,105284	0,413745
RBC	Mitral	ebi-a-GCST	SQMtTf	453596	rs541619	0,08682	0,105403	0,410111
RBC	Mitral	ebi-a-GCST	SQMtTf	453596	rs550057	0,104388	0,106207	0,325669
RBC	Mitral	ebi-a-GCST	SQMtTf	453596	rs5573572	0,087321	0,105296	0,406942
RBC	Mitral	ebi-a-GCST	SQMtTf	453596	rs5575079	0,079769	0,10457	0,445568
RBC	Mitral	ebi-a-GCST	SQMtTf	453596	rs5583473	0,089053	0,105225	0,397383
RBC	Mitral	ebi-a-GCST	SQMtTf	453596	rs5583585	0,087666	0,105291	0,405066
RBC	Mitral	ebi-a-GCST	SQMtTf	453596	rs5608492	0,091528	0,105073	0,383704
RBC	Mitral	ebi-a-GCST	SQMtTf	453596	rs5624181	0,084836	0,10525	0,420215
RBC	Mitral	ebi-a-GCST	SQMtTf	453596	rs5635638	0,087048	0,105585	0,409694
RBC	Mitral	ebi-a-GCST	SQMtTf	453596	rs5636474	0,089713	0,105211	0,393828
RBC	Mitral	ebi-a-GCST	SQMtTf	453596	rs5751348	0,088053	0,105527	0,404045
RBC	Mitral	ebi-a-GCST	SQMtTf	453596	rs5782885	0,083998	0,10531	0,425086
RBC	Mitral	ebi-a-GCST	SQMtTf	453596	rs5790821	0,079777	0,105309	0,448722
RBC	Mitral	ebi-a-GCST	SQMtTf	453596	rs5814140	0,071657	0,10459	0,493268
RBC	Mitral	ebi-a-GCST	SQMtTf	453596	rs581694	0,088791	0,105468	0,399859

RBC	Mitral	ebi-a-GCST	SQMtTf	453596	rs5897695(	0,084791	0,105237	0,420409
RBC	Mitral	ebi-a-GCST	SQMtTf	453596	rs590856	0,083795	0,105705	0,427943
RBC	Mitral	ebi-a-GCST	SQMtTf	453596	rs5977440!	0,087902	0,105334	0,403992
RBC	Mitral	ebi-a-GCST	SQMtTf	453596	rs5994579	0,080938	0,105345	0,4423
RBC	Mitral	ebi-a-GCST	SQMtTf	453596	rs6060912	0,089375	0,105313	0,396073
RBC	Mitral	ebi-a-GCST	SQMtTf	453596	rs6073289	0,086469	0,105337	0,411716
RBC	Mitral	ebi-a-GCST	SQMtTf	453596	rs6089381	0,086991	0,105322	0,408829
RBC	Mitral	ebi-a-GCST	SQMtTf	453596	rs6126019	0,096645	0,105304	0,358741
RBC	Mitral	ebi-a-GCST	SQMtTf	453596	rs618249	0,084377	0,105197	0,422502
RBC	Mitral	ebi-a-GCST	SQMtTf	453596	rs6182872:	0,086202	0,105315	0,413066
RBC	Mitral	ebi-a-GCST	SQMtTf	453596	rs6186369:	0,083665	0,105178	0,426347
RBC	Mitral	ebi-a-GCST	SQMtTf	453596	rs6191439:	0,083315	0,105077	0,427839
RBC	Mitral	ebi-a-GCST	SQMtTf	453596	rs6206227:	0,090531	0,105756	0,391978
RBC	Mitral	ebi-a-GCST	SQMtTf	453596	rs6225809-	0,082598	0,105254	0,432604
RBC	Mitral	ebi-a-GCST	SQMtTf	453596	rs6237157:	0,085833	0,1053	0,415002
RBC	Mitral	ebi-a-GCST	SQMtTf	453596	rs6241053!	0,089636	0,105394	0,395052
RBC	Mitral	ebi-a-GCST	SQMtTf	453596	rs6460528	0,081485	0,104912	0,437338
RBC	Mitral	ebi-a-GCST	SQMtTf	453596	rs646179	0,090151	0,105185	0,391409
RBC	Mitral	ebi-a-GCST	SQMtTf	453596	rs6486109	0,089262	0,105186	0,396098
RBC	Mitral	ebi-a-GCST	SQMtTf	453596	rs6494533	0,088618	0,105246	0,399785
RBC	Mitral	ebi-a-GCST	SQMtTf	453596	rs6538148	0,083024	0,105384	0,430802
RBC	Mitral	ebi-a-GCST	SQMtTf	453596	rs6544640	0,081839	0,104893	0,435268
RBC	Mitral	ebi-a-GCST	SQMtTf	453596	rs6592965	0,104884	0,105382	0,319601
RBC	Mitral	ebi-a-GCST	SQMtTf	453596	rs6602909	0,079679	0,105133	0,448519
RBC	Mitral	ebi-a-GCST	SQMtTf	453596	rs6673347	0,090448	0,105157	0,389719
RBC	Mitral	ebi-a-GCST	SQMtTf	453596	rs6697193	0,080753	0,104723	0,440644
RBC	Mitral	ebi-a-GCST	SQMtTf	453596	rs6702992	0,081721	0,104965	0,436244
RBC	Mitral	ebi-a-GCST	SQMtTf	453596	rs6706720	0,084254	0,105208	0,423231
RBC	Mitral	ebi-a-GCST	SQMtTf	453596	rs673583	0,08894	0,105198	0,397856
RBC	Mitral	ebi-a-GCST	SQMtTf	453596	rs6740980:	0,086739	0,105292	0,410056
RBC	Mitral	ebi-a-GCST	SQMtTf	453596	rs6756054(	0,079041	0,105516	0,453803
RBC	Mitral	ebi-a-GCST	SQMtTf	453596	rs6777965	0,083982	0,105406	0,425597
RBC	Mitral	ebi-a-GCST	SQMtTf	453596	rs6805945	0,083149	0,105392	0,430142
RBC	Mitral	ebi-a-GCST	SQMtTf	453596	rs6834187	0,089514	0,105173	0,394705
RBC	Mitral	ebi-a-GCST	SQMtTf	453596	rs685724	0,084502	0,105263	0,422109
RBC	Mitral	ebi-a-GCST	SQMtTf	453596	rs6864691	0,085317	0,105312	0,417862
RBC	Mitral	ebi-a-GCST	SQMtTf	453596	rs6899555	0,085018	0,105256	0,419248
RBC	Mitral	ebi-a-GCST	SQMtTf	453596	rs6954581	0,088896	0,105202	0,398109
RBC	Mitral	ebi-a-GCST	SQMtTf	453596	rs6994725	0,087953	0,105285	0,403503
RBC	Mitral	ebi-a-GCST	SQMtTf	453596	rs702634	0,08929	0,105197	0,395995
RBC	Mitral	ebi-a-GCST	SQMtTf	453596	rs7040572	0,095856	0,10441	0,358581
RBC	Mitral	ebi-a-GCST	SQMtTf	453596	rs7077322	0,085865	0,105286	0,414764
RBC	Mitral	ebi-a-GCST	SQMtTf	453596	rs7088969	0,0857	0,105298	0,415712
RBC	Mitral	ebi-a-GCST	SQMtTf	453596	rs7090091	0,083578	0,105102	0,426489
RBC	Mitral	ebi-a-GCST	SQMtTf	453596	rs7130503	0,087329	0,105293	0,406884
RBC	Mitral	ebi-a-GCST	SQMtTf	453596	rs7143938	0,089087	0,105284	0,397465
RBC	Mitral	ebi-a-GCST	SQMtTf	453596	rs7149013	0,087606	0,105316	0,4055
RBC	Mitral	ebi-a-GCST	SQMtTf	453596	rs7187289	0,090787	0,105337	0,388756
RBC	Mitral	ebi-a-GCST	SQMtTf	453596	rs7189510	0,085158	0,105267	0,418533
RBC	Mitral	ebi-a-GCST	SQMtTf	453596	rs7210099	0,086621	0,105302	0,410736

RBC	Mitral	ebi-a-GCST	SQMtTf	453596	rs7265464	0,096915	0,104383	0,35317
RBC	Mitral	ebi-a-GCST	SQMtTf	453596	rs7271979	0,091737	0,105063	0,382573
RBC	Mitral	ebi-a-GCST	SQMtTf	453596	rs7273157	0,080712	0,104783	0,441136
RBC	Mitral	ebi-a-GCST	SQMtTf	453596	rs7278167	0,086049	0,105464	0,414553
RBC	Mitral	ebi-a-GCST	SQMtTf	453596	rs7279961	0,088414	0,105238	0,400836
RBC	Mitral	ebi-a-GCST	SQMtTf	453596	rs7281323	0,084393	0,105214	0,422491
RBC	Mitral	ebi-a-GCST	SQMtTf	453596	rs7285446	0,089435	0,105291	0,395656
RBC	Mitral	ebi-a-GCST	SQMtTf	453596	rs7299903	0,09165	0,105133	0,383345
RBC	Mitral	ebi-a-GCST	SQMtTf	453596	rs7303652	0,088078	0,105294	0,402878
RBC	Mitral	ebi-a-GCST	SQMtTf	453596	rs7307932	0,083557	0,105181	0,426959
RBC	Mitral	ebi-a-GCST	SQMtTf	453596	rs7311930	0,091287	0,104888	0,384122
RBC	Mitral	ebi-a-GCST	SQMtTf	453596	rs7313642	0,091375	0,105269	0,385389
RBC	Mitral	ebi-a-GCST	SQMtTf	453596	rs7316967	0,07935	0,104728	0,448644
RBC	Mitral	ebi-a-GCST	SQMtTf	453596	rs7324360	0,088981	0,105305	0,39812
RBC	Mitral	ebi-a-GCST	SQMtTf	453596	rs744103	0,075492	0,105253	0,473223
RBC	Mitral	ebi-a-GCST	SQMtTf	453596	rs7496099	0,085142	0,10527	0,418634
RBC	Mitral	ebi-a-GCST	SQMtTf	453596	rs7507721	0,086943	0,105294	0,408966
RBC	Mitral	ebi-a-GCST	SQMtTf	453596	rs7525164	0,081478	0,105235	0,438783
RBC	Mitral	ebi-a-GCST	SQMtTf	453596	rs752590	0,081155	0,105336	0,441037
RBC	Mitral	ebi-a-GCST	SQMtTf	453596	rs7530647	0,087585	0,105278	0,405442
RBC	Mitral	ebi-a-GCST	SQMtTf	453596	rs7532110	0,083725	0,105264	0,426392
RBC	Mitral	ebi-a-GCST	SQMtTf	453596	rs7542570	0,092083	0,104842	0,379781
RBC	Mitral	ebi-a-GCST	SQMtTf	453596	rs7575789	0,086853	0,105393	0,409889
RBC	Mitral	ebi-a-GCST	SQMtTf	453596	rs7579941	0,089862	0,105164	0,392834
RBC	Mitral	ebi-a-GCST	SQMtTf	453596	rs7583880	0,083596	0,105222	0,426922
RBC	Mitral	ebi-a-GCST	SQMtTf	453596	rs7587489	0,086931	0,105366	0,409351
RBC	Mitral	ebi-a-GCST	SQMtTf	453596	rs7587636	0,089873	0,10519	0,392892
RBC	Mitral	ebi-a-GCST	SQMtTf	453596	rs760077	0,084877	0,105457	0,420904
RBC	Mitral	ebi-a-GCST	SQMtTf	453596	rs7606538	0,085489	0,105292	0,416835
RBC	Mitral	ebi-a-GCST	SQMtTf	453596	rs7618703	0,08498	0,105302	0,419663
RBC	Mitral	ebi-a-GCST	SQMtTf	453596	rs7650602	0,076507	0,105257	0,467314
RBC	Mitral	ebi-a-GCST	SQMtTf	453596	rs7651288	0,08527	0,10533	0,418199
RBC	Mitral	ebi-a-GCST	SQMtTf	453596	rs7676376	0,085514	0,105269	0,416598
RBC	Mitral	ebi-a-GCST	SQMtTf	453596	rs7705509	0,080308	0,105266	0,445521
RBC	Mitral	ebi-a-GCST	SQMtTf	453596	rs7705526	0,073992	0,105309	0,48229
RBC	Mitral	ebi-a-GCST	SQMtTf	453596	rs7705980	0,086786	0,105313	0,409898
RBC	Mitral	ebi-a-GCST	SQMtTf	453596	rs7708799	0,086251	0,105328	0,412853
RBC	Mitral	ebi-a-GCST	SQMtTf	453596	rs7714751	0,082977	0,105051	0,429598
RBC	Mitral	ebi-a-GCST	SQMtTf	453596	rs7751930	0,091264	0,104954	0,384543
RBC	Mitral	ebi-a-GCST	SQMtTf	453596	rs7777566	0,088753	0,105475	0,40009
RBC	Mitral	ebi-a-GCST	SQMtTf	453596	rs7785356	0,088864	0,105252	0,398499
RBC	Mitral	ebi-a-GCST	SQMtTf	453596	rs7785763	0,088188	0,105255	0,402117
RBC	Mitral	ebi-a-GCST	SQMtTf	453596	rs7803036	0,086813	0,105326	0,409808
RBC	Mitral	ebi-a-GCST	SQMtTf	453596	rs7810260	0,081686	0,104952	0,436382
RBC	Mitral	ebi-a-GCST	SQMtTf	453596	rs7817028	0,087189	0,105339	0,407841
RBC	Mitral	ebi-a-GCST	SQMtTf	453596	rs7819099	0,092831	0,104846	0,37594
RBC	Mitral	ebi-a-GCST	SQMtTf	453596	rs7819224	0,085469	0,105356	0,417231
RBC	Mitral	ebi-a-GCST	SQMtTf	453596	rs7828	0,090515	0,105095	0,389086
RBC	Mitral	ebi-a-GCST	SQMtTf	453596	rs786913	0,085477	0,105328	0,41706
RBC	Mitral	ebi-a-GCST	SQMtTf	453596	rs7874418	0,089051	0,105998	0,400839

RBC	Mitral	ebi-a-GCST	SQMtTf	453596	rs7876925(	0,084188	0,105186	0,423495
RBC	Mitral	ebi-a-GCST	SQMtTf	453596	rs7890903:	0,080666	0,105042	0,442523
RBC	Mitral	ebi-a-GCST	SQMtTf	453596	rs791270	0,086899	0,105308	0,409264
RBC	Mitral	ebi-a-GCST	SQMtTf	453596	rs7918400	0,086372	0,105305	0,412101
RBC	Mitral	ebi-a-GCST	SQMtTf	453596	rs7946785	0,086387	0,105301	0,412
RBC	Mitral	ebi-a-GCST	SQMtTf	453596	rs7952436	0,088003	0,105361	0,403574
RBC	Mitral	ebi-a-GCST	SQMtTf	453596	rs796040	0,088463	0,10525	0,400626
RBC	Mitral	ebi-a-GCST	SQMtTf	453596	rs7960765	0,091075	0,10503	0,385867
RBC	Mitral	ebi-a-GCST	SQMtTf	453596	rs7991576	0,091017	0,105251	0,387171
RBC	Mitral	ebi-a-GCST	SQMtTf	453596	rs800882	0,084255	0,105338	0,423796
RBC	Mitral	ebi-a-GCST	SQMtTf	453596	rs8015423	0,085401	0,105265	0,417195
RBC	Mitral	ebi-a-GCST	SQMtTf	453596	rs8019529	0,083233	0,10516	0,428655
RBC	Mitral	ebi-a-GCST	SQMtTf	453596	rs8020977	0,088769	0,105223	0,398877
RBC	Mitral	ebi-a-GCST	SQMtTf	453596	rs8030759:	0,086929	0,105297	0,409055
RBC	Mitral	ebi-a-GCST	SQMtTf	453596	rs8060857	0,088889	0,105443	0,399227
RBC	Mitral	ebi-a-GCST	SQMtTf	453596	rs8062405	0,089008	0,105367	0,398252
RBC	Mitral	ebi-a-GCST	SQMtTf	453596	rs8110787	0,090984	0,105836	0,389968
RBC	Mitral	ebi-a-GCST	SQMtTf	453596	rs8176635	0,083094	0,105409	0,430522
RBC	Mitral	ebi-a-GCST	SQMtTf	453596	rs8181322	0,082747	0,105009	0,430699
RBC	Mitral	ebi-a-GCST	SQMtTf	453596	rs8193003	0,085686	0,105302	0,415804
RBC	Mitral	ebi-a-GCST	SQMtTf	453596	rs837763	0,093076	0,105516	0,377721
RBC	Mitral	ebi-a-GCST	SQMtTf	453596	rs847821	0,089004	0,10523	0,39766
RBC	Mitral	ebi-a-GCST	SQMtTf	453596	rs853199	0,089029	0,105237	0,39756
RBC	Mitral	ebi-a-GCST	SQMtTf	453596	rs863678	0,087269	0,105391	0,407645
RBC	Mitral	ebi-a-GCST	SQMtTf	453596	rs865923	0,08842	0,105282	0,400998
RBC	Mitral	ebi-a-GCST	SQMtTf	453596	rs869785	0,077483	0,105429	0,462381
RBC	Mitral	ebi-a-GCST	SQMtTf	453596	rs8887	0,082974	0,105314	0,430774
RBC	Mitral	ebi-a-GCST	SQMtTf	453596	rs901683	0,081583	0,105446	0,439113
RBC	Mitral	ebi-a-GCST	SQMtTf	453596	rs904819	0,080682	0,10475	0,441162
RBC	Mitral	ebi-a-GCST	SQMtTf	453596	rs905670	0,080757	0,105065	0,442107
RBC	Mitral	ebi-a-GCST	SQMtTf	453596	rs918227	0,079205	0,104932	0,450358
RBC	Mitral	ebi-a-GCST	SQMtTf	453596	rs9349205	0,101643	0,106578	0,340238
RBC	Mitral	ebi-a-GCST	SQMtTf	453596	rs9366428	0,086996	0,105327	0,408826
RBC	Mitral	ebi-a-GCST	SQMtTf	453596	rs939317	0,088661	0,105243	0,399544
RBC	Mitral	ebi-a-GCST	SQMtTf	453596	rs9394605	0,083045	0,105055	0,429244
RBC	Mitral	ebi-a-GCST	SQMtTf	453596	rs9402540	0,090375	0,105126	0,389964
RBC	Mitral	ebi-a-GCST	SQMtTf	453596	rs9402702	0,087507	0,105372	0,406279
RBC	Mitral	ebi-a-GCST	SQMtTf	453596	rs9483788	0,090696	0,110097	0,410063
RBC	Mitral	ebi-a-GCST	SQMtTf	453596	rs950784	0,082333	0,104983	0,432897
RBC	Mitral	ebi-a-GCST	SQMtTf	453596	rs9508759	0,088217	0,105248	0,401929
RBC	Mitral	ebi-a-GCST	SQMtTf	453596	rs9521025	0,088827	0,1053	0,398916
RBC	Mitral	ebi-a-GCST	SQMtTf	453596	rs9549260	0,084811	0,105381	0,42093
RBC	Mitral	ebi-a-GCST	SQMtTf	453596	rs963837	0,081955	0,105294	0,43637
RBC	Mitral	ebi-a-GCST	SQMtTf	453596	rs9771385	0,082918	0,105317	0,431099
RBC	Mitral	ebi-a-GCST	SQMtTf	453596	rs9806453	0,09194	0,104942	0,380972
RBC	Mitral	ebi-a-GCST	SQMtTf	453596	rs9829214	0,084074	0,105228	0,424309
RBC	Mitral	ebi-a-GCST	SQMtTf	453596	rs9859077	0,08371	0,105237	0,426358
RBC	Mitral	ebi-a-GCST	SQMtTf	453596	rs9861443	0,085566	0,105316	0,416524
RBC	Mitral	ebi-a-GCST	SQMtTf	453596	rs9872347	0,07335	0,105281	0,485987
RBC	Mitral	ebi-a-GCST	SQMtTf	453596	rs988398	0,090439	0,105317	0,39049

RBC	Mitral	ebi-a-GCST	SQMtTf	453596	rs9892942	0,086325	0,105385	0,412708
RBC	Mitral	ebi-a-GCST	SQMtTf	453596	rs9895661	0,08664	0,105436	0,411227
RBC	Mitral	ebi-a-GCST	SQMtTf	453596	rs9959574	0,092023	0,104909	0,380391
RBC	Mitral	ebi-a-GCST	SQMtTf	453596	rs9984968	0,087233	0,105292	0,4074
RBC	Mitral	ebi-a-GCST	SQMtTf	453596	All	0,086676	0,105131	0,409676
WBC	Mitral	ebi-a-GCST	OO1PJt	454015	rs1004921(	0,19247	0,119188	0,106345
WBC	Mitral	ebi-a-GCST	OO1PJt	454015	rs1008656	0,189088	0,119177	0,112601
WBC	Mitral	ebi-a-GCST	OO1PJt	454015	rs1010499	0,189746	0,119272	0,111639
WBC	Mitral	ebi-a-GCST	OO1PJt	454015	rs1013875	0,190953	0,119316	0,109509
WBC	Mitral	ebi-a-GCST	OO1PJt	454015	rs1014696	0,194332	0,119115	0,102792
WBC	Mitral	ebi-a-GCST	OO1PJt	454015	rs1016476	0,185291	0,119236	0,120188
WBC	Mitral	ebi-a-GCST	OO1PJt	454015	rs1020175	0,189518	0,119196	0,111841
WBC	Mitral	ebi-a-GCST	OO1PJt	454015	rs1033568	0,197504	0,119302	0,097823
WBC	Mitral	ebi-a-GCST	OO1PJt	454015	rs1040132	0,188095	0,119133	0,114366
WBC	Mitral	ebi-a-GCST	OO1PJt	454015	rs1046690	0,199308	0,119086	0,094201
WBC	Mitral	ebi-a-GCST	OO1PJt	454015	rs1047891	0,19299	0,119242	0,105562
WBC	Mitral	ebi-a-GCST	OO1PJt	454015	rs1049478	0,185816	0,119192	0,119005
WBC	Mitral	ebi-a-GCST	OO1PJt	454015	rs1060286	0,198304	0,118522	0,094298
WBC	Mitral	ebi-a-GCST	OO1PJt	454015	rs1060402	0,198155	0,118876	0,095533
WBC	Mitral	ebi-a-GCST	OO1PJt	454015	rs1074809	0,190004	0,119226	0,111016
WBC	Mitral	ebi-a-GCST	OO1PJt	454015	rs1075728	0,192472	0,119369	0,106873
WBC	Mitral	ebi-a-GCST	OO1PJt	454015	rs1076599	0,187582	0,119083	0,115205
WBC	Mitral	ebi-a-GCST	OO1PJt	454015	rs1080853	0,191571	0,119206	0,108042
WBC	Mitral	ebi-a-GCST	OO1PJt	454015	rs1081482(	0,193576	0,119092	0,10407
WBC	Mitral	ebi-a-GCST	OO1PJt	454015	rs1082872	0,178391	0,119804	0,136483
WBC	Mitral	ebi-a-GCST	OO1PJt	454015	rs1084470(	0,204043	0,11885	0,086013
WBC	Mitral	ebi-a-GCST	OO1PJt	454015	rs1088957	0,192741	0,119747	0,107491
WBC	Mitral	ebi-a-GCST	OO1PJt	454015	rs1091661	0,200551	0,11886	0,091546
WBC	Mitral	ebi-a-GCST	OO1PJt	454015	rs1093501	0,18718	0,119107	0,11606
WBC	Mitral	ebi-a-GCST	OO1PJt	454015	rs1098633	0,194362	0,119167	0,102889
WBC	Mitral	ebi-a-GCST	OO1PJt	454015	rs1099243	0,192164	0,119201	0,106941
WBC	Mitral	ebi-a-GCST	OO1PJt	454015	rs1102217	0,196871	0,118914	0,097807
WBC	Mitral	ebi-a-GCST	OO1PJt	454015	rs1106488	0,193206	0,119218	0,105101
WBC	Mitral	ebi-a-GCST	OO1PJt	454015	rs1118770	0,19276	0,119189	0,105821
WBC	Mitral	ebi-a-GCST	OO1PJt	454015	rs1118783	0,195236	0,119169	0,101358
WBC	Mitral	ebi-a-GCST	OO1PJt	454015	rs1121273	0,186529	0,118982	0,116949
WBC	Mitral	ebi-a-GCST	OO1PJt	454015	rs1122370	0,195541	0,118876	0,099987
WBC	Mitral	ebi-a-GCST	OO1PJt	454015	rs1122820	0,189889	0,119257	0,111326
WBC	Mitral	ebi-a-GCST	OO1PJt	454015	rs1124411	0,187992	0,119112	0,114502
WBC	Mitral	ebi-a-GCST	OO1PJt	454015	rs1125643	0,187001	0,119026	0,116162
WBC	Mitral	ebi-a-GCST	OO1PJt	454015	rs1134736	0,191681	0,11922	0,107881
WBC	Mitral	ebi-a-GCST	OO1PJt	454015	rs1137023	0,188302	0,119172	0,114089
WBC	Mitral	ebi-a-GCST	OO1PJt	454015	rs1140506	0,188573	0,119356	0,114124
WBC	Mitral	ebi-a-GCST	OO1PJt	454015	rs1143537	0,192658	0,119153	0,1059
WBC	Mitral	ebi-a-GCST	OO1PJt	454015	rs1158721	0,193519	0,119159	0,104368
WBC	Mitral	ebi-a-GCST	OO1PJt	454015	rs1159352(	0,192006	0,119234	0,107326
WBC	Mitral	ebi-a-GCST	OO1PJt	454015	rs1160096	0,191386	0,119199	0,108361
WBC	Mitral	ebi-a-GCST	OO1PJt	454015	rs1161148(	0,191601	0,11919	0,107938
WBC	Mitral	ebi-a-GCST	OO1PJt	454015	rs1164265	0,194385	0,119113	0,102694
WBC	Mitral	ebi-a-GCST	OO1PJt	454015	rs1164412	0,194888	0,119146	0,101901

WBC	Mitral	ebi-a-GCST	OO1PJt	454015	rs1164769‹	0,188616	0,119151	0,113422
WBC	Mitral	ebi-a-GCST	OO1PJt	454015	rs1167309‹	0,190852	0,119892	0,111417
WBC	Mitral	ebi-a-GCST	OO1PJt	454015	rs1169159‹	0,190808	0,119206	0,109453
WBC	Mitral	ebi-a-GCST	OO1PJt	454015	rs1170685‹	0,188209	0,119308	0,114681
WBC	Mitral	ebi-a-GCST	OO1PJt	454015	rs1171255‹	0,191052	0,119289	0,109247
WBC	Mitral	ebi-a-GCST	OO1PJt	454015	rs1172362‹	0,183196	0,119278	0,124568
WBC	Mitral	ebi-a-GCST	OO1PJt	454015	rs1172570‹	0,152734	0,119777	0,202254
WBC	Mitral	ebi-a-GCST	OO1PJt	454015	rs1173566‹	0,188909	0,119273	0,11323
WBC	Mitral	ebi-a-GCST	OO1PJt	454015	rs1174177‹	0,184442	0,119158	0,121652
WBC	Mitral	ebi-a-GCST	OO1PJt	454015	rs1174466‹	0,191354	0,119241	0,108544
WBC	Mitral	ebi-a-GCST	OO1PJt	454015	rs1176874‹	0,190822	0,119211	0,10944
WBC	Mitral	ebi-a-GCST	OO1PJt	454015	rs1177848‹	0,184303	0,118721	0,120564
WBC	Mitral	ebi-a-GCST	OO1PJt	454015	rs1180016‹	0,18992	0,119227	0,111178
WBC	Mitral	ebi-a-GCST	OO1PJt	454015	rs1180838‹	0,187827	0,119099	0,114782
WBC	Mitral	ebi-a-GCST	OO1PJt	454015	rs1212123‹	0,188288	0,119182	0,114147
WBC	Mitral	ebi-a-GCST	OO1PJt	454015	rs1227996	0,199677	0,11911	0,093658
WBC	Mitral	ebi-a-GCST	OO1PJt	454015	rs1236325‹	0,200802	0,118492	0,090144
WBC	Mitral	ebi-a-GCST	OO1PJt	454015	rs1242301‹	0,202376	0,118247	0,086995
WBC	Mitral	ebi-a-GCST	OO1PJt	454015	rs1242477‹	0,186034	0,119007	0,118001
WBC	Mitral	ebi-a-GCST	OO1PJt	454015	rs1245035	0,190422	0,119234	0,110255
WBC	Mitral	ebi-a-GCST	OO1PJt	454015	rs1249472‹	0,18723	0,119113	0,11598
WBC	Mitral	ebi-a-GCST	OO1PJt	454015	rs1255061‹	0,20175	0,119004	0,090015
WBC	Mitral	ebi-a-GCST	OO1PJt	454015	rs1255524‹	0,192774	0,119416	0,106461
WBC	Mitral	ebi-a-GCST	OO1PJt	454015	rs1260326	0,196573	0,119446	0,099822
WBC	Mitral	ebi-a-GCST	OO1PJt	454015	rs1267610‹	0,179348	0,118441	0,129965
WBC	Mitral	ebi-a-GCST	OO1PJt	454015	rs1273784‹	0,192662	0,119142	0,105861
WBC	Mitral	ebi-a-GCST	OO1PJt	454015	rs1278407‹	0,185089	0,118958	0,119728
WBC	Mitral	ebi-a-GCST	OO1PJt	454015	rs1285886	0,181491	0,119027	0,12731
WBC	Mitral	ebi-a-GCST	OO1PJt	454015	rs1287440‹	0,188656	0,119231	0,113587
WBC	Mitral	ebi-a-GCST	OO1PJt	454015	rs1288649	0,203345	0,118453	0,086038
WBC	Mitral	ebi-a-GCST	OO1PJt	454015	rs1305502‹	0,186817	0,119153	0,116909
WBC	Mitral	ebi-a-GCST	OO1PJt	454015	rs1313285‹	0,198915	0,119071	0,094809
WBC	Mitral	ebi-a-GCST	OO1PJt	454015	rs1315565‹	0,190377	0,119218	0,110294
WBC	Mitral	ebi-a-GCST	OO1PJt	454015	rs1318072‹	0,191876	0,119237	0,107574
WBC	Mitral	ebi-a-GCST	OO1PJt	454015	rs1323126‹	0,178841	0,118061	0,129817
WBC	Mitral	ebi-a-GCST	OO1PJt	454015	rs1339297‹	0,188177	0,119186	0,114369
WBC	Mitral	ebi-a-GCST	OO1PJt	454015	rs1380281‹	0,184803	0,119081	0,120682
WBC	Mitral	ebi-a-GCST	OO1PJt	454015	rs1382846‹	0,193575	0,119335	0,10478
WBC	Mitral	ebi-a-GCST	OO1PJt	454015	rs139402	0,194123	0,119267	0,103602
WBC	Mitral	ebi-a-GCST	OO1PJt	454015	rs1411424	0,198527	0,119246	0,095943
WBC	Mitral	ebi-a-GCST	OO1PJt	454015	rs1412445	0,178824	0,118388	0,130917
WBC	Mitral	ebi-a-GCST	OO1PJt	454015	rs1415431‹	0,191101	0,119219	0,108948
WBC	Mitral	ebi-a-GCST	OO1PJt	454015	rs1417256	0,194021	0,119068	0,103209
WBC	Mitral	ebi-a-GCST	OO1PJt	454015	rs1430037‹	0,190929	0,119204	0,109221
WBC	Mitral	ebi-a-GCST	OO1PJt	454015	rs14408	0,173617	0,119426	0,146013
WBC	Mitral	ebi-a-GCST	OO1PJt	454015	rs1452099‹	0,183012	0,118713	0,123165
WBC	Mitral	ebi-a-GCST	OO1PJt	454015	rs1468102	0,181143	0,118527	0,126442
WBC	Mitral	ebi-a-GCST	OO1PJt	454015	rs1469217	0,198044	0,118976	0,095998
WBC	Mitral	ebi-a-GCST	OO1PJt	454015	rs1487131‹	0,191805	0,119278	0,107824
WBC	Mitral	ebi-a-GCST	OO1PJt	454015	rs1506494‹	0,192894	0,119183	0,105562

WBC	Mitral	ebi-a-GCST	OO1PJt	454015	rs1611236	0,201476	0,119383	0,09148
WBC	Mitral	ebi-a-GCST	OO1PJt	454015	rs1685007[illegible]	0,185376	0,119679	0,121395
WBC	Mitral	ebi-a-GCST	OO1PJt	454015	rs1693960[illegible]	0,196544	0,119278	0,099397
WBC	Mitral	ebi-a-GCST	OO1PJt	454015	rs1697807[illegible]	0,188187	0,119213	0,114433
WBC	Mitral	ebi-a-GCST	OO1PJt	454015	rs1704143[illegible]	0,190666	0,119277	0,109929
WBC	Mitral	ebi-a-GCST	OO1PJt	454015	rs1719900[illegible]	0,195557	0,118963	0,100207
WBC	Mitral	ebi-a-GCST	OO1PJt	454015	rs1727086[illegible]	0,185479	0,119359	0,120193
WBC	Mitral	ebi-a-GCST	OO1PJt	454015	rs1738074	0,193041	0,119265	0,105534
WBC	Mitral	ebi-a-GCST	OO1PJt	454015	rs1738668[illegible]	0,197051	0,119013	0,097781
WBC	Mitral	ebi-a-GCST	OO1PJt	454015	rs174549	0,18981	0,119364	0,111794
WBC	Mitral	ebi-a-GCST	OO1PJt	454015	rs175714	0,189457	0,119203	0,111978
WBC	Mitral	ebi-a-GCST	OO1PJt	454015	rs1773733[illegible]	0,193084	0,11916	0,10515
WBC	Mitral	ebi-a-GCST	OO1PJt	454015	rs1775869[illegible]	0,188125	0,119279	0,114753
WBC	Mitral	ebi-a-GCST	OO1PJt	454015	rs1778507[illegible]	0,190311	0,119225	0,110436
WBC	Mitral	ebi-a-GCST	OO1PJt	454015	rs177918	0,190341	0,119289	0,110571
WBC	Mitral	ebi-a-GCST	OO1PJt	454015	rs1779807	0,195286	0,118979	0,100725
WBC	Mitral	ebi-a-GCST	OO1PJt	454015	rs180943	0,195677	0,119121	0,100449
WBC	Mitral	ebi-a-GCST	OO1PJt	454015	rs1822534	0,187555	0,119396	0,116214
WBC	Mitral	ebi-a-GCST	OO1PJt	454015	rs1860545	0,1852	0,118914	0,119368
WBC	Mitral	ebi-a-GCST	OO1PJt	454015	rs1891033	0,192387	0,119171	0,106444
WBC	Mitral	ebi-a-GCST	OO1PJt	454015	rs1919316	0,192599	0,119146	0,105988
WBC	Mitral	ebi-a-GCST	OO1PJt	454015	rs1930249	0,192695	0,119222	0,106037
WBC	Mitral	ebi-a-GCST	OO1PJt	454015	rs1933295	0,187903	0,119103	0,114645
WBC	Mitral	ebi-a-GCST	OO1PJt	454015	rs1947897	0,186877	0,119129	0,116719
WBC	Mitral	ebi-a-GCST	OO1PJt	454015	rs1997577	0,194144	0,119082	0,103031
WBC	Mitral	ebi-a-GCST	OO1PJt	454015	rs2012623[illegible]	0,192412	0,119169	0,106396
WBC	Mitral	ebi-a-GCST	OO1PJt	454015	rs2024115	0,19675	0,118892	0,097952
WBC	Mitral	ebi-a-GCST	OO1PJt	454015	rs2038700	0,190467	0,119536	0,111075
WBC	Mitral	ebi-a-GCST	OO1PJt	454015	rs2049045	0,191337	0,119213	0,108494
WBC	Mitral	ebi-a-GCST	OO1PJt	454015	rs2068330	0,188186	0,119233	0,114496
WBC	Mitral	ebi-a-GCST	OO1PJt	454015	rs2080506	0,184956	0,118857	0,119679
WBC	Mitral	ebi-a-GCST	OO1PJt	454015	rs2082382	0,185539	0,119187	0,119541
WBC	Mitral	ebi-a-GCST	OO1PJt	454015	rs2084312	0,197485	0,119149	0,097424
WBC	Mitral	ebi-a-GCST	OO1PJt	454015	rs2089979	0,184777	0,119496	0,122032
WBC	Mitral	ebi-a-GCST	OO1PJt	454015	rs2091084	0,194925	0,119088	0,101669
WBC	Mitral	ebi-a-GCST	OO1PJt	454015	rs2107717	0,1872	0,119082	0,115943
WBC	Mitral	ebi-a-GCST	OO1PJt	454015	rs2158799	0,202271	0,119592	0,090771
WBC	Mitral	ebi-a-GCST	OO1PJt	454015	rs218264	0,187227	0,119327	0,116642
WBC	Mitral	ebi-a-GCST	OO1PJt	454015	rs2194067	0,189226	0,119205	0,112421
WBC	Mitral	ebi-a-GCST	OO1PJt	454015	rs2205190	0,188493	0,119186	0,113762
WBC	Mitral	ebi-a-GCST	OO1PJt	454015	rs2227322	0,173514	0,122922	0,158074
WBC	Mitral	ebi-a-GCST	OO1PJt	454015	rs2229075	0,19627	0,118909	0,098823
WBC	Mitral	ebi-a-GCST	OO1PJt	454015	rs2240717	0,192375	0,119287	0,106809
WBC	Mitral	ebi-a-GCST	OO1PJt	454015	rs2249742	0,205185	0,121403	0,091005
WBC	Mitral	ebi-a-GCST	OO1PJt	454015	rs2260302	0,193178	0,119266	0,105291
WBC	Mitral	ebi-a-GCST	OO1PJt	454015	rs228606	0,18019	0,118562	0,128562
WBC	Mitral	ebi-a-GCST	OO1PJt	454015	rs2290846	0,189088	0,119229	0,112755
WBC	Mitral	ebi-a-GCST	OO1PJt	454015	rs2322586	0,188294	0,119163	0,114076
WBC	Mitral	ebi-a-GCST	OO1PJt	454015	rs2358581	0,178277	0,118917	0,133829
WBC	Mitral	ebi-a-GCST	OO1PJt	454015	rs2371108	0,196447	0,119141	0,099176

WBC	Mitral	ebi-a-GCST	OO1PJt	454015	rs238914	0,187707	0,119409	0,115958
WBC	Mitral	ebi-a-GCST	OO1PJt	454015	rs2412771	0,187382	0,119228	0,116037
WBC	Mitral	ebi-a-GCST	OO1PJt	454015	rs2421200	0,205105	0,118501	0,083482
WBC	Mitral	ebi-a-GCST	OO1PJt	454015	rs2439963	0,192928	0,119138	0,10537
WBC	Mitral	ebi-a-GCST	OO1PJt	454015	rs2462661	0,190824	0,119256	0,109572
WBC	Mitral	ebi-a-GCST	OO1PJt	454015	rs247826	0,187206	0,119403	0,116917
WBC	Mitral	ebi-a-GCST	OO1PJt	454015	rs2519093	0,184417	0,119477	0,122703
WBC	Mitral	ebi-a-GCST	OO1PJt	454015	rs2562751	0,195392	0,118928	0,100394
WBC	Mitral	ebi-a-GCST	OO1PJt	454015	rs257063	0,186666	0,119042	0,116866
WBC	Mitral	ebi-a-GCST	OO1PJt	454015	rs2594836	0,212805	0,118489	0,072496
WBC	Mitral	ebi-a-GCST	OO1PJt	454015	rs2615061	0,196046	0,119092	0,099728
WBC	Mitral	ebi-a-GCST	OO1PJt	454015	rs2632372	0,193963	0,119262	0,103872
WBC	Mitral	ebi-a-GCST	OO1PJt	454015	rs2710804	0,186696	0,119268	0,117499
WBC	Mitral	ebi-a-GCST	OO1PJt	454015	rs2729707	0,194934	0,119284	0,102217
WBC	Mitral	ebi-a-GCST	OO1PJt	454015	rs2759321	0,193652	0,119084	0,103912
WBC	Mitral	ebi-a-GCST	OO1PJt	454015	rs2803606	0,188305	0,119162	0,11405
WBC	Mitral	ebi-a-GCST	OO1PJt	454015	rs2807742	0,186146	0,119383	0,11894
WBC	Mitral	ebi-a-GCST	OO1PJt	454015	rs284317	0,183609	0,118995	0,12283
WBC	Mitral	ebi-a-GCST	OO1PJt	454015	rs2850567[illegible]	0,18832	0,11927	0,11435
WBC	Mitral	ebi-a-GCST	OO1PJt	454015	rs2853075[illegible]	0,189759	0,119328	0,111783
WBC	Mitral	ebi-a-GCST	OO1PJt	454015	rs2857176[illegible]	0,192202	0,119251	0,107018
WBC	Mitral	ebi-a-GCST	OO1PJt	454015	rs2857622[illegible]	0,196876	0,119174	0,098535
WBC	Mitral	ebi-a-GCST	OO1PJt	454015	rs2867816[illegible]	0,191075	0,119259	0,109115
WBC	Mitral	ebi-a-GCST	OO1PJt	454015	rs2957419	0,197245	0,11874	0,096683
WBC	Mitral	ebi-a-GCST	OO1PJt	454015	rs2964199	0,197902	0,118771	0,095664
WBC	Mitral	ebi-a-GCST	OO1PJt	454015	rs2979489	0,18765	0,119184	0,115383
WBC	Mitral	ebi-a-GCST	OO1PJt	454015	rs2992836	0,184592	0,119074	0,121085
WBC	Mitral	ebi-a-GCST	OO1PJt	454015	rs303753	0,186396	0,119195	0,117865
WBC	Mitral	ebi-a-GCST	OO1PJt	454015	rs3128932	0,186	0,119094	0,118337
WBC	Mitral	ebi-a-GCST	OO1PJt	454015	rs3184504	0,196554	0,120823	0,10378
WBC	Mitral	ebi-a-GCST	OO1PJt	454015	rs3395198[illegible]	0,186195	0,119129	0,118059
WBC	Mitral	ebi-a-GCST	OO1PJt	454015	rs3419844[illegible]	0,198201	0,119199	0,096358
WBC	Mitral	ebi-a-GCST	OO1PJt	454015	rs3429835[illegible]	0,193245	0,119199	0,104975
WBC	Mitral	ebi-a-GCST	OO1PJt	454015	rs3459908[illegible]	0,19212	0,119639	0,108311
WBC	Mitral	ebi-a-GCST	OO1PJt	454015	rs3463454[illegible]	0,188148	0,119387	0,115037
WBC	Mitral	ebi-a-GCST	OO1PJt	454015	rs3499033[illegible]	0,18731	0,119043	0,11561
WBC	Mitral	ebi-a-GCST	OO1PJt	454015	rs3511294[illegible]	0,194732	0,119203	0,10234
WBC	Mitral	ebi-a-GCST	OO1PJt	454015	rs3589858[illegible]	0,185078	0,118883	0,119515
WBC	Mitral	ebi-a-GCST	OO1PJt	454015	rs3592965[illegible]	0,190403	0,119213	0,110229
WBC	Mitral	ebi-a-GCST	OO1PJt	454015	rs3605189[illegible]	0,195327	0,118901	0,100431
WBC	Mitral	ebi-a-GCST	OO1PJt	454015	rs3608452[illegible]	0,193589	0,119145	0,104202
WBC	Mitral	ebi-a-GCST	OO1PJt	454015	rs3732378	0,193415	0,119205	0,104686
WBC	Mitral	ebi-a-GCST	OO1PJt	454015	rs3735485	0,191369	0,11944	0,109107
WBC	Mitral	ebi-a-GCST	OO1PJt	454015	rs3740049	0,190368	0,119228	0,11034
WBC	Mitral	ebi-a-GCST	OO1PJt	454015	rs3747869	0,191142	0,119335	0,109219
WBC	Mitral	ebi-a-GCST	OO1PJt	454015	rs3754224	0,190285	0,119226	0,110488
WBC	Mitral	ebi-a-GCST	OO1PJt	454015	rs3762297	0,187498	0,119254	0,11589
WBC	Mitral	ebi-a-GCST	OO1PJt	454015	rs3771101[illegible]	0,19084	0,119346	0,109811
WBC	Mitral	ebi-a-GCST	OO1PJt	454015	rs3781454	0,184291	0,119202	0,122094
WBC	Mitral	ebi-a-GCST	OO1PJt	454015	rs3793537	0,193358	0,119233	0,104871

WBC	Mitral	ebi-a-GCST	OO1PJt	454015	rs3795503	0,192271	0,119209	0,106768
WBC	Mitral	ebi-a-GCST	OO1PJt	454015	rs3812849	0,187264	0,119206	0,1162
WBC	Mitral	ebi-a-GCST	OO1PJt	454015	rs3818717	0,187459	0,119244	0,115936
WBC	Mitral	ebi-a-GCST	OO1PJt	454015	rs3822733	0,199107	0,118913	0,094054
WBC	Mitral	ebi-a-GCST	OO1PJt	454015	rs3856364	0,191687	0,11921	0,10784
WBC	Mitral	ebi-a-GCST	OO1PJt	454015	rs385893	0,198596	0,118993	0,095123
WBC	Mitral	ebi-a-GCST	OO1PJt	454015	rs398474	0,19275	0,119183	0,105822
WBC	Mitral	ebi-a-GCST	OO1PJt	454015	rs4074672	0,188571	0,119254	0,113819
WBC	Mitral	ebi-a-GCST	OO1PJt	454015	rs4127253(	0,192426	0,119177	0,106393
WBC	Mitral	ebi-a-GCST	OO1PJt	454015	rs4131338:	0,174141	0,117475	0,138243
WBC	Mitral	ebi-a-GCST	OO1PJt	454015	rs4276561	0,184661	0,118958	0,120585
WBC	Mitral	ebi-a-GCST	OO1PJt	454015	rs430989	0,189707	0,119203	0,111505
WBC	Mitral	ebi-a-GCST	OO1PJt	454015	rs445	0,173602	0,120155	0,14851
WBC	Mitral	ebi-a-GCST	OO1PJt	454015	rs4468717	0,188819	0,11918	0,113122
WBC	Mitral	ebi-a-GCST	OO1PJt	454015	rs4490348	0,191358	0,119208	0,108439
WBC	Mitral	ebi-a-GCST	OO1PJt	454015	rs4535497	0,191107	0,119216	0,108928
WBC	Mitral	ebi-a-GCST	OO1PJt	454015	rs4570393	0,191624	0,119194	0,10791
WBC	Mitral	ebi-a-GCST	OO1PJt	454015	rs4599108	0,187429	0,119195	0,115844
WBC	Mitral	ebi-a-GCST	OO1PJt	454015	rs4623401	0,193774	0,119225	0,104103
WBC	Mitral	ebi-a-GCST	OO1PJt	454015	rs4632345	0,185383	0,118931	0,119058
WBC	Mitral	ebi-a-GCST	OO1PJt	454015	rs464609	0,188782	0,119189	0,113219
WBC	Mitral	ebi-a-GCST	OO1PJt	454015	rs4657690	0,195398	0,118947	0,10044
WBC	Mitral	ebi-a-GCST	OO1PJt	454015	rs4682844	0,184908	0,119314	0,121198
WBC	Mitral	ebi-a-GCST	OO1PJt	454015	rs4689	0,190699	0,119236	0,109746
WBC	Mitral	ebi-a-GCST	OO1PJt	454015	rs4703890	0,196297	0,119262	0,099777
WBC	Mitral	ebi-a-GCST	OO1PJt	454015	rs4704826	0,201256	0,118282	0,08885
WBC	Mitral	ebi-a-GCST	OO1PJt	454015	rs4707609	0,181493	0,118961	0,127097
WBC	Mitral	ebi-a-GCST	OO1PJt	454015	rs4721650	0,189198	0,119195	0,112446
WBC	Mitral	ebi-a-GCST	OO1PJt	454015	rs4733823	0,187542	0,119266	0,115843
WBC	Mitral	ebi-a-GCST	OO1PJt	454015	rs47340	0,189882	0,119233	0,111265
WBC	Mitral	ebi-a-GCST	OO1PJt	454015	rs4734879	0,18897	0,119274	0,113117
WBC	Mitral	ebi-a-GCST	OO1PJt	454015	rs4746522	0,192024	0,119201	0,107196
WBC	Mitral	ebi-a-GCST	OO1PJt	454015	rs4748429	0,193765	0,119059	0,103639
WBC	Mitral	ebi-a-GCST	OO1PJt	454015	rs4760	0,170411	0,119714	0,154597
WBC	Mitral	ebi-a-GCST	OO1PJt	454015	rs4761234	0,194522	0,119103	0,102422
WBC	Mitral	ebi-a-GCST	OO1PJt	454015	rs479404	0,189812	0,119213	0,111338
WBC	Mitral	ebi-a-GCST	OO1PJt	454015	rs4805881	0,198882	0,11866	0,093724
WBC	Mitral	ebi-a-GCST	OO1PJt	454015	rs4807440	0,191899	0,119344	0,107846
WBC	Mitral	ebi-a-GCST	OO1PJt	454015	rs4844390	0,185472	0,119196	0,119701
WBC	Mitral	ebi-a-GCST	OO1PJt	454015	rs4908508	0,191287	0,11933	0,108934
WBC	Mitral	ebi-a-GCST	OO1PJt	454015	rs4924450	0,190837	0,11923	0,109469
WBC	Mitral	ebi-a-GCST	OO1PJt	454015	rs4970996	0,201462	0,1177	0,08696
WBC	Mitral	ebi-a-GCST	OO1PJt	454015	rs4980661	0,191393	0,11925	0,1085
WBC	Mitral	ebi-a-GCST	OO1PJt	454015	rs527544	0,196861	0,1189	0,097786
WBC	Mitral	ebi-a-GCST	OO1PJt	454015	rs533483	0,194861	0,119032	0,101622
WBC	Mitral	ebi-a-GCST	OO1PJt	454015	rs5565917(	0,190181	0,119229	0,110694
WBC	Mitral	ebi-a-GCST	OO1PJt	454015	rs5570927:	0,195801	0,119517	0,101366
WBC	Mitral	ebi-a-GCST	OO1PJt	454015	rs5601126:	0,186449	0,119245	0,117918
WBC	Mitral	ebi-a-GCST	OO1PJt	454015	rs560194	0,192175	0,119189	0,106884
WBC	Mitral	ebi-a-GCST	OO1PJt	454015	rs5618886!	0,194944	0,11931	0,102274

WBC	Mitral	ebi-a-GCST	OO1PJt	454015	rs5622702	0,192023	0,119248	0,107338
WBC	Mitral	ebi-a-GCST	OO1PJt	454015	rs5629302	0,19257	0,11975	0,107812
WBC	Mitral	ebi-a-GCST	OO1PJt	454015	rs5638817	0,192353	0,120118	0,109297
WBC	Mitral	ebi-a-GCST	OO1PJt	454015	rs5667456	0,193716	0,119142	0,103967
WBC	Mitral	ebi-a-GCST	OO1PJt	454015	rs5707001	0,190712	0,119227	0,109695
WBC	Mitral	ebi-a-GCST	OO1PJt	454015	rs5746451	0,194842	0,119212	0,102173
WBC	Mitral	ebi-a-GCST	OO1PJt	454015	rs5883393	0,193545	0,11908	0,104091
WBC	Mitral	ebi-a-GCST	OO1PJt	454015	rs5940418	0,19318	0,119189	0,105062
WBC	Mitral	ebi-a-GCST	OO1PJt	454015	rs5969707	0,18497	0,119568	0,121866
WBC	Mitral	ebi-a-GCST	OO1PJt	454015	rs5997742	0,191332	0,119208	0,108488
WBC	Mitral	ebi-a-GCST	OO1PJt	454015	rs6029234	0,207577	0,118351	0,079446
WBC	Mitral	ebi-a-GCST	OO1PJt	454015	rs6045615	0,19133	0,11925	0,108614
WBC	Mitral	ebi-a-GCST	OO1PJt	454015	rs6046684	0,191084	0,119231	0,109014
WBC	Mitral	ebi-a-GCST	OO1PJt	454015	rs6069534	0,190529	0,119348	0,110397
WBC	Mitral	ebi-a-GCST	OO1PJt	454015	rs6091892	0,194011	0,119213	0,103646
WBC	Mitral	ebi-a-GCST	OO1PJt	454015	rs611461	0,189458	0,119234	0,112068
WBC	Mitral	ebi-a-GCST	OO1PJt	454015	rs614943	0,19862	0,118353	0,093306
WBC	Mitral	ebi-a-GCST	OO1PJt	454015	rs6159214	0,188341	0,119263	0,11429
WBC	Mitral	ebi-a-GCST	OO1PJt	454015	rs6173928	0,185269	0,11888	0,119124
WBC	Mitral	ebi-a-GCST	OO1PJt	454015	rs6186376	0,192355	0,119833	0,108452
WBC	Mitral	ebi-a-GCST	OO1PJt	454015	rs6191836	0,195607	0,118848	0,099792
WBC	Mitral	ebi-a-GCST	OO1PJt	454015	rs6207580	0,188804	0,119195	0,113196
WBC	Mitral	ebi-a-GCST	OO1PJt	454015	rs6217324	0,187974	0,119192	0,114777
WBC	Mitral	ebi-a-GCST	OO1PJt	454015	rs6222704	0,188293	0,119241	0,114314
WBC	Mitral	ebi-a-GCST	OO1PJt	454015	rs6232971	0,189709	0,119247	0,111635
WBC	Mitral	ebi-a-GCST	OO1PJt	454015	rs633323	0,19131	0,119203	0,108513
WBC	Mitral	ebi-a-GCST	OO1PJt	454015	rs6437277	0,187462	0,119067	0,115391
WBC	Mitral	ebi-a-GCST	OO1PJt	454015	rs6440732	0,189763	0,119259	0,111568
WBC	Mitral	ebi-a-GCST	OO1PJt	454015	rs6445424	0,190245	0,119221	0,110547
WBC	Mitral	ebi-a-GCST	OO1PJt	454015	rs6446561	0,187293	0,119224	0,116197
WBC	Mitral	ebi-a-GCST	OO1PJt	454015	rs6474403	0,19065	0,119211	0,109761
WBC	Mitral	ebi-a-GCST	OO1PJt	454015	rs6488901	0,18899	0,119172	0,112772
WBC	Mitral	ebi-a-GCST	OO1PJt	454015	rs6500550	0,190681	0,1193	0,10997
WBC	Mitral	ebi-a-GCST	OO1PJt	454015	rs6543094	0,18641	0,119125	0,117625
WBC	Mitral	ebi-a-GCST	OO1PJt	454015	rs6580229	0,194107	0,119319	0,103782
WBC	Mitral	ebi-a-GCST	OO1PJt	454015	rs6583435	0,195889	0,119082	0,09997
WBC	Mitral	ebi-a-GCST	OO1PJt	454015	rs6679677	0,209348	0,118772	0,077967
WBC	Mitral	ebi-a-GCST	OO1PJt	454015	rs6696259	0,195471	0,11917	0,10095
WBC	Mitral	ebi-a-GCST	OO1PJt	454015	rs6753819	0,183326	0,118725	0,122559
WBC	Mitral	ebi-a-GCST	OO1PJt	454015	rs6755895	0,191225	0,11929	0,10893
WBC	Mitral	ebi-a-GCST	OO1PJt	454015	rs6760993	0,195512	0,119068	0,100587
WBC	Mitral	ebi-a-GCST	OO1PJt	454015	rs6779340	0,182828	0,118937	0,124249
WBC	Mitral	ebi-a-GCST	OO1PJt	454015	rs6808307	0,192274	0,119175	0,106663
WBC	Mitral	ebi-a-GCST	OO1PJt	454015	rs6817881	0,186459	0,119041	0,117268
WBC	Mitral	ebi-a-GCST	OO1PJt	454015	rs6831368	0,187544	0,11924	0,115758
WBC	Mitral	ebi-a-GCST	OO1PJt	454015	rs6878780	0,197001	0,118824	0,097334
WBC	Mitral	ebi-a-GCST	OO1PJt	454015	rs6902672	0,190008	0,119236	0,111039
WBC	Mitral	ebi-a-GCST	OO1PJt	454015	rs6924387	0,183665	0,118893	0,122396
WBC	Mitral	ebi-a-GCST	OO1PJt	454015	rs6924861	0,193227	0,119323	0,105369
WBC	Mitral	ebi-a-GCST	OO1PJt	454015	rs6927569	0,190959	0,119343	0,109579

WBC	Mitral	ebi-a-GCST	OO1PJt	454015	rs696	0,184852	0,118996	0,12032
WBC	Mitral	ebi-a-GCST	OO1PJt	454015	rs696825	0,188236	0,1193	0,114602
WBC	Mitral	ebi-a-GCST	OO1PJt	454015	rs7005996	0,189585	0,119231	0,11182
WBC	Mitral	ebi-a-GCST	OO1PJt	454015	rs7018391	0,190267	0,11921	0,110472
WBC	Mitral	ebi-a-GCST	OO1PJt	454015	rs7036656	0,19779	0,11924	0,097166
WBC	Mitral	ebi-a-GCST	OO1PJt	454015	rs7082470	0,200511	0,11919	0,092516
WBC	Mitral	ebi-a-GCST	OO1PJt	454015	rs7098414	0,195163	0,118977	0,100934
WBC	Mitral	ebi-a-GCST	OO1PJt	454015	rs7134738	0,193053	0,11916	0,105206
WBC	Mitral	ebi-a-GCST	OO1PJt	454015	rs7146875(	0,186166	0,118998	0,117715
WBC	Mitral	ebi-a-GCST	OO1PJt	454015	rs7151032!	0,185005	0,118905	0,119731
WBC	Mitral	ebi-a-GCST	OO1PJt	454015	rs7154870{	0,178418	0,118338	0,131631
WBC	Mitral	ebi-a-GCST	OO1PJt	454015	rs7157692(	0,18732	0,119059	0,115641
WBC	Mitral	ebi-a-GCST	OO1PJt	454015	rs7159281	0,192265	0,119186	0,106713
WBC	Mitral	ebi-a-GCST	OO1PJt	454015	rs7163850	0,191472	0,119207	0,108226
WBC	Mitral	ebi-a-GCST	OO1PJt	454015	rs7177338	0,18722	0,119174	0,116188
WBC	Mitral	ebi-a-GCST	OO1PJt	454015	rs7180079	0,17982	0,118919	0,130501
WBC	Mitral	ebi-a-GCST	OO1PJt	454015	rs7214290	0,192973	0,119189	0,105438
WBC	Mitral	ebi-a-GCST	OO1PJt	454015	rs7225843	0,17993	0,11909	0,13082
WBC	Mitral	ebi-a-GCST	OO1PJt	454015	rs723585	0,198981	0,118911	0,094257
WBC	Mitral	ebi-a-GCST	OO1PJt	454015	rs7267112	0,196447	0,118704	0,097938
WBC	Mitral	ebi-a-GCST	OO1PJt	454015	rs7272602	0,197537	0,119314	0,097801
WBC	Mitral	ebi-a-GCST	OO1PJt	454015	rs7279086	0,184529	0,119216	0,121658
WBC	Mitral	ebi-a-GCST	OO1PJt	454015	rs7283930	0,187766	0,119106	0,11492
WBC	Mitral	ebi-a-GCST	OO1PJt	454015	rs7293364(	0,188569	0,119164	0,113552
WBC	Mitral	ebi-a-GCST	OO1PJt	454015	rs7295177	0,193989	0,119081	0,103302
WBC	Mitral	ebi-a-GCST	OO1PJt	454015	rs7297371	0,191303	0,119245	0,108651
WBC	Mitral	ebi-a-GCST	OO1PJt	454015	rs729761	0,186391	0,119076	0,11751
WBC	Mitral	ebi-a-GCST	OO1PJt	454015	rs7302887	0,187464	0,119071	0,115396
WBC	Mitral	ebi-a-GCST	OO1PJt	454015	rs7326825	0,183339	0,11903	0,123492
WBC	Mitral	ebi-a-GCST	OO1PJt	454015	rs7356226{	0,189225	0,119202	0,112414
WBC	Mitral	ebi-a-GCST	OO1PJt	454015	rs7399089	0,194555	0,119052	0,102218
WBC	Mitral	ebi-a-GCST	OO1PJt	454015	rs740422	0,189292	0,119198	0,112275
WBC	Mitral	ebi-a-GCST	OO1PJt	454015	rs743580	0,193661	0,119123	0,104009
WBC	Mitral	ebi-a-GCST	OO1PJt	454015	rs7469372	0,187311	0,119171	0,116003
WBC	Mitral	ebi-a-GCST	OO1PJt	454015	rs7487314	0,186525	0,119278	0,11787
WBC	Mitral	ebi-a-GCST	OO1PJt	454015	rs7547562	0,192007	0,119255	0,107385
WBC	Mitral	ebi-a-GCST	OO1PJt	454015	rs7552783	0,19499	0,119128	0,101671
WBC	Mitral	ebi-a-GCST	OO1PJt	454015	rs7569257	0,193831	0,119342	0,104341
WBC	Mitral	ebi-a-GCST	OO1PJt	454015	rs7589371!	0,186647	0,119121	0,117145
WBC	Mitral	ebi-a-GCST	OO1PJt	454015	rs763361	0,190768	0,119221	0,109573
WBC	Mitral	ebi-a-GCST	OO1PJt	454015	rs7639292	0,18822	0,119256	0,114501
WBC	Mitral	ebi-a-GCST	OO1PJt	454015	rs7641045	0,189759	0,119202	0,111403
WBC	Mitral	ebi-a-GCST	OO1PJt	454015	rs7642810(	0,172126	0,118968	0,147947
WBC	Mitral	ebi-a-GCST	OO1PJt	454015	rs764358	0,192564	0,119164	0,106104
WBC	Mitral	ebi-a-GCST	OO1PJt	454015	rs7705526	0,179895	0,119102	0,130934
WBC	Mitral	ebi-a-GCST	OO1PJt	454015	rs7776857	0,184907	0,119074	0,120453
WBC	Mitral	ebi-a-GCST	OO1PJt	454015	rs7803075	0,192772	0,119315	0,10617
WBC	Mitral	ebi-a-GCST	OO1PJt	454015	rs7822169	0,192011	0,119291	0,107483
WBC	Mitral	ebi-a-GCST	OO1PJt	454015	rs7846314	0,172904	0,119206	0,14693
WBC	Mitral	ebi-a-GCST	OO1PJt	454015	rs7890816	0,184573	0,118808	0,120293

WBC	Mitral	ebi-a-GCST	OO1PJt	454015	rs7923752(	0,192893	0,119151	0,105471
WBC	Mitral	ebi-a-GCST	OO1PJt	454015	rs7927292(	0,19731	0,119083	0,09754
WBC	Mitral	ebi-a-GCST	OO1PJt	454015	rs7949917	0,188135	0,11916	0,114372
WBC	Mitral	ebi-a-GCST	OO1PJt	454015	rs7955734	0,199967	0,119007	0,0929
WBC	Mitral	ebi-a-GCST	OO1PJt	454015	rs7971658	0,19314	0,119326	0,105536
WBC	Mitral	ebi-a-GCST	OO1PJt	454015	rs7986401(	0,188016	0,119143	0,114549
WBC	Mitral	ebi-a-GCST	OO1PJt	454015	rs8024737	0,188159	0,11914	0,114266
WBC	Mitral	ebi-a-GCST	OO1PJt	454015	rs806321	0,193572	0,119185	0,104348
WBC	Mitral	ebi-a-GCST	OO1PJt	454015	rs8071086	0,191398	0,119219	0,1084
WBC	Mitral	ebi-a-GCST	OO1PJt	454015	rs8079218	0,197577	0,118791	0,096267
WBC	Mitral	ebi-a-GCST	OO1PJt	454015	rs8081395	0,179217	0,11945	0,133522
WBC	Mitral	ebi-a-GCST	OO1PJt	454015	rs8176580	0,188401	0,119205	0,113997
WBC	Mitral	ebi-a-GCST	OO1PJt	454015	rs8179	0,189164	0,119472	0,113347
WBC	Mitral	ebi-a-GCST	OO1PJt	454015	rs865031	0,184042	0,118742	0,121159
WBC	Mitral	ebi-a-GCST	OO1PJt	454015	rs8705	0,184056	0,119257	0,122746
WBC	Mitral	ebi-a-GCST	OO1PJt	454015	rs880749	0,189361	0,119375	0,112678
WBC	Mitral	ebi-a-GCST	OO1PJt	454015	rs895819	0,199273	0,118813	0,093504
WBC	Mitral	ebi-a-GCST	OO1PJt	454015	rs9165	0,185892	0,119064	0,118458
WBC	Mitral	ebi-a-GCST	OO1PJt	454015	rs9264277	0,193891	0,119359	0,104283
WBC	Mitral	ebi-a-GCST	OO1PJt	454015	rs9313822	0,194515	0,118992	0,102114
WBC	Mitral	ebi-a-GCST	OO1PJt	454015	rs9329341	0,19454	0,119149	0,102522
WBC	Mitral	ebi-a-GCST	OO1PJt	454015	rs935655	0,187535	0,119122	0,115415
WBC	Mitral	ebi-a-GCST	OO1PJt	454015	rs9375447	0,191622	0,119218	0,107985
WBC	Mitral	ebi-a-GCST	OO1PJt	454015	rs9390461	0,188198	0,119201	0,114373
WBC	Mitral	ebi-a-GCST	OO1PJt	454015	rs9429767	0,195268	0,118989	0,100785
WBC	Mitral	ebi-a-GCST	OO1PJt	454015	rs9466071	0,190953	0,119229	0,109251
WBC	Mitral	ebi-a-GCST	OO1PJt	454015	rs9508005	0,191029	0,119207	0,109046
WBC	Mitral	ebi-a-GCST	OO1PJt	454015	rs9526795	0,198297	0,118431	0,094058
WBC	Mitral	ebi-a-GCST	OO1PJt	454015	rs9590390	0,188281	0,119183	0,114162
WBC	Mitral	ebi-a-GCST	OO1PJt	454015	rs9656395	0,191325	0,119259	0,108652
WBC	Mitral	ebi-a-GCST	OO1PJt	454015	rs9680589	0,186366	0,119001	0,117327
WBC	Mitral	ebi-a-GCST	OO1PJt	454015	rs9815043	0,185451	0,119291	0,120039
WBC	Mitral	ebi-a-GCST	OO1PJt	454015	rs9819371	0,188114	0,119327	0,11492
WBC	Mitral	ebi-a-GCST	OO1PJt	454015	rs9863	0,184342	0,11904	0,121485
WBC	Mitral	ebi-a-GCST	OO1PJt	454015	rs9867398	0,191826	0,119197	0,107546
WBC	Mitral	ebi-a-GCST	OO1PJt	454015	rs987121	0,193127	0,119165	0,10509
WBC	Mitral	ebi-a-GCST	OO1PJt	454015	rs9885207	0,191046	0,11926	0,109171
WBC	Mitral	ebi-a-GCST	OO1PJt	454015	rs9925985	0,186965	0,11902	0,116212
WBC	Mitral	ebi-a-GCST	OO1PJt	454015	rs9933582	0,19591	0,119099	0,099982
WBC	Mitral	ebi-a-GCST	OO1PJt	454015	rs9947760	0,187411	0,119247	0,116038
WBC	Mitral	ebi-a-GCST	OO1PJt	454015	rs9970896	0,198362	0,119538	0,097035
WBC	Mitral	ebi-a-GCST	OO1PJt	454015	rs9977672	0,184315	0,119047	0,121562
WBC	Mitral	ebi-a-GCST	OO1PJt	454015	All	0,190567	0,119008	0,109313
Ht	Mitral	ebi-a-GCST	CkbBol	444474	rs1004156!	-0,11883	0,128702	0,355846
Ht	Mitral	ebi-a-GCST	CkbBol	444474	rs1015330	-0,11314	0,12885	0,379905
Ht	Mitral	ebi-a-GCST	CkbBol	444474	rs1016144	-0,11441	0,128873	0,374674
Ht	Mitral	ebi-a-GCST	CkbBol	444474	rs1016834!	-0,08551	0,130762	0,51313
Ht	Mitral	ebi-a-GCST	CkbBol	444474	rs1026522	-0,10669	0,130543	0,413784
Ht	Mitral	ebi-a-GCST	CkbBol	444474	rs1045813(	-0,11275	0,128854	0,381551
Ht	Mitral	ebi-a-GCST	CkbBol	444474	rs1052571	-0,11165	0,128847	0,386198

Ht	Mitral	ebi-a-GCST CkbBol	444474	rs1075871	-0,12207	0,128683	0,342806
Ht	Mitral	ebi-a-GCST CkbBol	444474	rs1077835	-0,10808	0,128685	0,400982
Ht	Mitral	ebi-a-GCST CkbBol	444474	rs1081644[illegible]	-0,12147	0,128159	0,343241
Ht	Mitral	ebi-a-GCST CkbBol	444474	rs1084045[illegible]	-0,1173	0,128868	0,362712
Ht	Mitral	ebi-a-GCST CkbBol	444474	rs1085031[illegible]	-0,11744	0,128699	0,361486
Ht	Mitral	ebi-a-GCST CkbBol	444474	rs1085904[illegible]	-0,10646	0,128345	0,40684
Ht	Mitral	ebi-a-GCST CkbBol	444474	rs1089897[illegible]	-0,11568	0,128854	0,369318
Ht	Mitral	ebi-a-GCST CkbBol	444474	rs1090002[illegible]	-0,11993	0,128768	0,351662
Ht	Mitral	ebi-a-GCST CkbBol	444474	rs1090994[illegible]	-0,11945	0,128777	0,353617
Ht	Mitral	ebi-a-GCST CkbBol	444474	rs1095693[illegible]	-0,11814	0,129005	0,359778
Ht	Mitral	ebi-a-GCST CkbBol	444474	rs1095860[illegible]	-0,10639	0,128275	0,406871
Ht	Mitral	ebi-a-GCST CkbBol	444474	rs1097163[illegible]	-0,10971	0,128652	0,393786
Ht	Mitral	ebi-a-GCST CkbBol	444474	rs1105637[illegible]	-0,1138	0,128878	0,377221
Ht	Mitral	ebi-a-GCST CkbBol	444474	rs1109825[illegible]	-0,1235	0,128299	0,335744
Ht	Mitral	ebi-a-GCST CkbBol	444474	rs1110542	-0,10664	0,128463	0,406486
Ht	Mitral	ebi-a-GCST CkbBol	444474	rs1112492[illegible]	-0,1174	0,128913	0,362439
Ht	Mitral	ebi-a-GCST CkbBol	444474	rs1125386[illegible]	-0,11708	0,128808	0,363381
Ht	Mitral	ebi-a-GCST CkbBol	444474	rs1125853[illegible]	-0,10952	0,128749	0,394958
Ht	Mitral	ebi-a-GCST CkbBol	444474	rs1128756[illegible]	-0,11071	0,128828	0,390162
Ht	Mitral	ebi-a-GCST CkbBol	444474	rs1130520[illegible]	-0,11362	0,128868	0,377968
Ht	Mitral	ebi-a-GCST CkbBol	444474	rs1136701[illegible]	-0,11514	0,12893	0,371844
Ht	Mitral	ebi-a-GCST CkbBol	444474	rs1159862[illegible]	-0,10792	0,128999	0,402803
Ht	Mitral	ebi-a-GCST CkbBol	444474	rs1161548[illegible]	-0,10616	0,128746	0,409621
Ht	Mitral	ebi-a-GCST CkbBol	444474	rs1166758[illegible]	-0,10664	0,128607	0,406988
Ht	Mitral	ebi-a-GCST CkbBol	444474	rs1167749[illegible]	-0,11686	0,12877	0,364147
Ht	Mitral	ebi-a-GCST CkbBol	444474	rs1169057[illegible]	-0,11134	0,1288	0,387343
Ht	Mitral	ebi-a-GCST CkbBol	444474	rs1169248[illegible]	-0,10954	0,128638	0,394456
Ht	Mitral	ebi-a-GCST CkbBol	444474	rs1169490[illegible]	-0,11609	0,128978	0,368091
Ht	Mitral	ebi-a-GCST CkbBol	444474	rs1171079[illegible]	-0,11102	0,128734	0,388485
Ht	Mitral	ebi-a-GCST CkbBol	444474	rs1171119[illegible]	-0,11981	0,128431	0,350894
Ht	Mitral	ebi-a-GCST CkbBol	444474	rs1172001[illegible]	-0,10825	0,128662	0,400159
Ht	Mitral	ebi-a-GCST CkbBol	444474	rs1175870[illegible]	-0,10585	0,128427	0,40981
Ht	Mitral	ebi-a-GCST CkbBol	444474	rs1197893[illegible]	-0,11921	0,128503	0,353557
Ht	Mitral	ebi-a-GCST CkbBol	444474	rs1212520[illegible]	-0,10784	0,128459	0,401209
Ht	Mitral	ebi-a-GCST CkbBol	444474	rs1221420[illegible]	-0,11699	0,128746	0,363518
Ht	Mitral	ebi-a-GCST CkbBol	444474	rs1234735[illegible]	-0,1173	0,128722	0,362137
Ht	Mitral	ebi-a-GCST CkbBol	444474	rs123698	-0,10841	0,129096	0,401026
Ht	Mitral	ebi-a-GCST CkbBol	444474	rs1242198	-0,10712	0,128731	0,405354
Ht	Mitral	ebi-a-GCST CkbBol	444474	rs1250561[illegible]	-0,11188	0,128859	0,385253
Ht	Mitral	ebi-a-GCST CkbBol	444474	rs1252771[illegible]	-0,1126	0,128865	0,382243
Ht	Mitral	ebi-a-GCST CkbBol	444474	rs1256061	-0,13039	0,128215	0,30918
Ht	Mitral	ebi-a-GCST CkbBol	444474	rs1257415	-0,11276	0,128883	0,381621
Ht	Mitral	ebi-a-GCST CkbBol	444474	rs1258495[illegible]	-0,11562	0,128851	0,369571
Ht	Mitral	ebi-a-GCST CkbBol	444474	rs1258792[illegible]	-0,11409	0,12888	0,376029
Ht	Mitral	ebi-a-GCST CkbBol	444474	rs1259160[illegible]	-0,1201	0,128392	0,349571
Ht	Mitral	ebi-a-GCST CkbBol	444474	rs1277294[illegible]	-0,11041	0,12882	0,391389
Ht	Mitral	ebi-a-GCST CkbBol	444474	rs1280044[illegible]	-0,11366	0,12886	0,377747
Ht	Mitral	ebi-a-GCST CkbBol	444474	rs1281151[illegible]	-0,11494	0,129024	0,373005
Ht	Mitral	ebi-a-GCST CkbBol	444474	rs1281441[illegible]	-0,11712	0,128737	0,362935
Ht	Mitral	ebi-a-GCST CkbBol	444474	rs128494	-0,11506	0,129163	0,37301

Ht	Mitral	ebi-a-GCST	CkbBol	444474	rs1288186[illegible]	-0,11136	0,128764	0,387136
Ht	Mitral	ebi-a-GCST	CkbBol	444474	rs1288926[illegible]	-0,11974	0,128822	0,352634
Ht	Mitral	ebi-a-GCST	CkbBol	444474	rs1292071	-0,12497	0,128583	0,331094
Ht	Mitral	ebi-a-GCST	CkbBol	444474	rs1294587[illegible]	-0,12103	0,128812	0,347446
Ht	Mitral	ebi-a-GCST	CkbBol	444474	rs1296773[illegible]	-0,11081	0,128818	0,389693
Ht	Mitral	ebi-a-GCST	CkbBol	444474	rs12985	-0,11308	0,128956	0,380547
Ht	Mitral	ebi-a-GCST	CkbBol	444474	rs1298766[illegible]	-0,11887	0,128623	0,355407
Ht	Mitral	ebi-a-GCST	CkbBol	444474	rs1300528[illegible]	-0,11301	0,128844	0,380416
Ht	Mitral	ebi-a-GCST	CkbBol	444474	rs1306224[illegible]	-0,10573	0,128378	0,41017
Ht	Mitral	ebi-a-GCST	CkbBol	444474	rs1307639[illegible]	-0,11299	0,128912	0,380761
Ht	Mitral	ebi-a-GCST	CkbBol	444474	rs1310353[illegible]	-0,11027	0,128827	0,392015
Ht	Mitral	ebi-a-GCST	CkbBol	444474	rs1310732[illegible]	-0,12733	0,128638	0,32227
Ht	Mitral	ebi-a-GCST	CkbBol	444474	rs1314635[illegible]	-0,11613	0,129105	0,368381
Ht	Mitral	ebi-a-GCST	CkbBol	444474	rs1323682[illegible]	-0,11141	0,128812	0,387082
Ht	Mitral	ebi-a-GCST	CkbBol	444474	rs1338921[illegible]	-0,11203	0,128872	0,384673
Ht	Mitral	ebi-a-GCST	CkbBol	444474	rs1340817	-0,10769	0,128864	0,40333
Ht	Mitral	ebi-a-GCST	CkbBol	444474	rs1341555[illegible]	-0,11492	0,128857	0,372478
Ht	Mitral	ebi-a-GCST	CkbBol	444474	rs1354674	-0,11477	0,128889	0,373241
Ht	Mitral	ebi-a-GCST	CkbBol	444474	rs1383196[illegible]	-0,12013	0,12841	0,349529
Ht	Mitral	ebi-a-GCST	CkbBol	444474	rs1404256[illegible]	-0,10916	0,12851	0,395634
Ht	Mitral	ebi-a-GCST	CkbBol	444474	rs1420624[illegible]	-0,11984	0,128641	0,35154
Ht	Mitral	ebi-a-GCST	CkbBol	444474	rs1426374	-0,10743	0,128636	0,403615
Ht	Mitral	ebi-a-GCST	CkbBol	444474	rs1434282	-0,11708	0,128886	0,363678
Ht	Mitral	ebi-a-GCST	CkbBol	444474	rs1438577[illegible]	-0,11663	0,12879	0,365169
Ht	Mitral	ebi-a-GCST	CkbBol	444474	rs1438898	-0,10977	0,128985	0,394737
Ht	Mitral	ebi-a-GCST	CkbBol	444474	rs1445561	-0,11696	0,128747	0,363637
Ht	Mitral	ebi-a-GCST	CkbBol	444474	rs1472226	-0,10953	0,128591	0,394347
Ht	Mitral	ebi-a-GCST	CkbBol	444474	rs1478045[illegible]	-0,108	0,128541	0,400809
Ht	Mitral	ebi-a-GCST	CkbBol	444474	rs1479559	-0,11965	0,128779	0,352836
Ht	Mitral	ebi-a-GCST	CkbBol	444474	rs1484621[illegible]	-0,11984	0,128619	0,351465
Ht	Mitral	ebi-a-GCST	CkbBol	444474	rs1508443[illegible]	-0,10733	0,128978	0,405304
Ht	Mitral	ebi-a-GCST	CkbBol	444474	rs1508507[illegible]	-0,11577	0,129002	0,369503
Ht	Mitral	ebi-a-GCST	CkbBol	444474	rs1537520	-0,10821	0,128412	0,399419
Ht	Mitral	ebi-a-GCST	CkbBol	444474	rs154121	-0,11182	0,128865	0,385536
Ht	Mitral	ebi-a-GCST	CkbBol	444474	rs1611236	-0,10958	0,128719	0,394612
Ht	Mitral	ebi-a-GCST	CkbBol	444474	rs165944	-0,11163	0,128834	0,386248
Ht	Mitral	ebi-a-GCST	CkbBol	444474	rs168019	-0,11862	0,128579	0,356251
Ht	Mitral	ebi-a-GCST	CkbBol	444474	rs1687406[illegible]	-0,11261	0,12897	0,382578
Ht	Mitral	ebi-a-GCST	CkbBol	444474	rs1690997[illegible]	-0,11509	0,128856	0,37175
Ht	Mitral	ebi-a-GCST	CkbBol	444474	rs1691848[illegible]	-0,11494	0,128849	0,372358
Ht	Mitral	ebi-a-GCST	CkbBol	444474	rs1694275[illegible]	-0,10455	0,12766	0,412796
Ht	Mitral	ebi-a-GCST	CkbBol	444474	rs1700644[illegible]	-0,12268	0,128766	0,340727
Ht	Mitral	ebi-a-GCST	CkbBol	444474	rs1727840[illegible]	-0,11574	0,12924	0,370497
Ht	Mitral	ebi-a-GCST	CkbBol	444474	rs174574	-0,11427	0,129037	0,375865
Ht	Mitral	ebi-a-GCST	CkbBol	444474	rs1747636[illegible]	-0,08157	0,132316	0,537582
Ht	Mitral	ebi-a-GCST	CkbBol	444474	rs1764532[illegible]	-0,10929	0,128588	0,395386
Ht	Mitral	ebi-a-GCST	CkbBol	444474	rs1765474[illegible]	-0,10874	0,128882	0,398832
Ht	Mitral	ebi-a-GCST	CkbBol	444474	rs1781669[illegible]	-0,11772	0,128856	0,36095
Ht	Mitral	ebi-a-GCST	CkbBol	444474	rs1800562	-0,11345	0,130862	0,385963
Ht	Mitral	ebi-a-GCST	CkbBol	444474	rs1800961	-0,10029	0,128527	0,435202

Ht	Mitral	ebi-a-GCST CkbBol	444474	rs1831977	-0,11353	0,128888	0,378388
Ht	Mitral	ebi-a-GCST CkbBol	444474	rs1840885[illegible]	-0,11673	0,129205	0,366288
Ht	Mitral	ebi-a-GCST CkbBol	444474	rs1841677	-0,11304	0,128873	0,380404
Ht	Mitral	ebi-a-GCST CkbBol	444474	rs1855557	-0,10974	0,128703	0,393853
Ht	Mitral	ebi-a-GCST CkbBol	444474	rs1868274	-0,12747	0,128697	0,32196
Ht	Mitral	ebi-a-GCST CkbBol	444474	rs1874228	-0,11867	0,12882	0,356939
Ht	Mitral	ebi-a-GCST CkbBol	444474	rs1893989	-0,10997	0,128812	0,393253
Ht	Mitral	ebi-a-GCST CkbBol	444474	rs1924930	-0,11108	0,128804	0,388471
Ht	Mitral	ebi-a-GCST CkbBol	444474	rs1949481	-0,11054	0,128747	0,390585
Ht	Mitral	ebi-a-GCST CkbBol	444474	rs1987070	-0,11554	0,128899	0,370061
Ht	Mitral	ebi-a-GCST CkbBol	444474	rs2014780[illegible]	-0,1118	0,128776	0,385315
Ht	Mitral	ebi-a-GCST CkbBol	444474	rs2015803	-0,10583	0,128675	0,410835
Ht	Mitral	ebi-a-GCST CkbBol	444474	rs2029466	-0,12109	0,128997	0,347887
Ht	Mitral	ebi-a-GCST CkbBol	444474	rs2043082	-0,11843	0,128726	0,357562
Ht	Mitral	ebi-a-GCST CkbBol	444474	rs2047293	-0,11038	0,128754	0,391278
Ht	Mitral	ebi-a-GCST CkbBol	444474	rs2052284	-0,12869	0,127973	0,314602
Ht	Mitral	ebi-a-GCST CkbBol	444474	rs2059427	-0,11446	0,12894	0,374707
Ht	Mitral	ebi-a-GCST CkbBol	444474	rs2103901	-0,11823	0,128721	0,358337
Ht	Mitral	ebi-a-GCST CkbBol	444474	rs218264	-0,10758	0,12944	0,405922
Ht	Mitral	ebi-a-GCST CkbBol	444474	rs2184540	-0,11308	0,128873	0,380222
Ht	Mitral	ebi-a-GCST CkbBol	444474	rs2186037	-0,11286	0,129071	0,381914
Ht	Mitral	ebi-a-GCST CkbBol	444474	rs2209098	-0,11806	0,128887	0,359677
Ht	Mitral	ebi-a-GCST CkbBol	444474	rs2255293	-0,11592	0,128827	0,368225
Ht	Mitral	ebi-a-GCST CkbBol	444474	rs2281089	-0,11575	0,128859	0,369054
Ht	Mitral	ebi-a-GCST CkbBol	444474	rs2293088	-0,10718	0,128426	0,403949
Ht	Mitral	ebi-a-GCST CkbBol	444474	rs2294915	-0,11882	0,128918	0,356703
Ht	Mitral	ebi-a-GCST CkbBol	444474	rs2333024	-0,11902	0,128621	0,354773
Ht	Mitral	ebi-a-GCST CkbBol	444474	rs2343596	-0,11714	0,128743	0,362903
Ht	Mitral	ebi-a-GCST CkbBol	444474	rs2379120	-0,11542	0,128912	0,370617
Ht	Mitral	ebi-a-GCST CkbBol	444474	rs2396083	-0,13004	0,129038	0,313575
Ht	Mitral	ebi-a-GCST CkbBol	444474	rs2414053	-0,11473	0,128855	0,37326
Ht	Mitral	ebi-a-GCST CkbBol	444474	rs2538043	-0,11269	0,128844	0,381795
Ht	Mitral	ebi-a-GCST CkbBol	444474	rs2541119	-0,11555	0,128871	0,369911
Ht	Mitral	ebi-a-GCST CkbBol	444474	rs2561566	-0,1229	0,128609	0,339269
Ht	Mitral	ebi-a-GCST CkbBol	444474	rs2576161	-0,1139	0,128859	0,376729
Ht	Mitral	ebi-a-GCST CkbBol	444474	rs258753	-0,11463	0,128878	0,373783
Ht	Mitral	ebi-a-GCST CkbBol	444474	rs2628117	-0,10543	0,127873	0,409674
Ht	Mitral	ebi-a-GCST CkbBol	444474	rs2799185	-0,11193	0,128825	0,384915
Ht	Mitral	ebi-a-GCST CkbBol	444474	rs2823139	-0,12261	0,128976	0,34177
Ht	Mitral	ebi-a-GCST CkbBol	444474	rs2823270	-0,11329	0,129122	0,380257
Ht	Mitral	ebi-a-GCST CkbBol	444474	rs2834287	-0,11145	0,129012	0,387653
Ht	Mitral	ebi-a-GCST CkbBol	444474	rs2835435	-0,11038	0,129053	0,39239
Ht	Mitral	ebi-a-GCST CkbBol	444474	rs2841640[illegible]	-0,1137	0,128851	0,377542
Ht	Mitral	ebi-a-GCST CkbBol	444474	rs2843233[illegible]	-0,11171	0,12919	0,387188
Ht	Mitral	ebi-a-GCST CkbBol	444474	rs2845494[illegible]	-0,10845	0,128568	0,39895
Ht	Mitral	ebi-a-GCST CkbBol	444474	rs2860637[illegible]	-0,11672	0,128785	0,364771
Ht	Mitral	ebi-a-GCST CkbBol	444474	rs2871567[illegible]	-0,11298	0,128841	0,380542
Ht	Mitral	ebi-a-GCST CkbBol	444474	rs2917949	-0,11039	0,128855	0,391619
Ht	Mitral	ebi-a-GCST CkbBol	444474	rs2943557	-0,11234	0,128865	0,383329
Ht	Mitral	ebi-a-GCST CkbBol	444474	rs2943637	-0,11511	0,128859	0,371674

Ht	Mitral	ebi-a-GCST	CkbBol	444474	rs2970870	-0,10944	0,128611	0,394808
Ht	Mitral	ebi-a-GCST	CkbBol	444474	rs3112629	-0,11937	0,128621	0,353389
Ht	Mitral	ebi-a-GCST	CkbBol	444474	rs3184504	-0,11649	0,130519	0,372115
Ht	Mitral	ebi-a-GCST	CkbBol	444474	rs3213545	-0,11944	0,128811	0,353803
Ht	Mitral	ebi-a-GCST	CkbBol	444474	rs333947	-0,11191	0,128798	0,3849
Ht	Mitral	ebi-a-GCST	CkbBol	444474	rs34164109	-0,12082	0,13109	0,356703
Ht	Mitral	ebi-a-GCST	CkbBol	444474	rs3461812(	-0,10429	0,127892	0,414803
Ht	Mitral	ebi-a-GCST	CkbBol	444474	rs3462714[illegible]	-0,1226	0,127857	0,337636
Ht	Mitral	ebi-a-GCST	CkbBol	444474	rs3476152[illegible]	-0,10053	0,127598	0,430753
Ht	Mitral	ebi-a-GCST	CkbBol	444474	rs3493361[illegible]	-0,10973	0,128735	0,394026
Ht	Mitral	ebi-a-GCST	CkbBol	444474	rs3512440[illegible]	-0,1135	0,129157	0,379514
Ht	Mitral	ebi-a-GCST	CkbBol	444474	rs3521910[illegible]	-0,11055	0,128757	0,390583
Ht	Mitral	ebi-a-GCST	CkbBol	444474	rs3524099[illegible]	-0,12103	0,128802	0,347401
Ht	Mitral	ebi-a-GCST	CkbBol	444474	rs3573649[illegible]	-0,12612	0,127718	0,323398
Ht	Mitral	ebi-a-GCST	CkbBol	444474	rs3606573[illegible]	-0,12651	0,128948	0,326557
Ht	Mitral	ebi-a-GCST	CkbBol	444474	rs3684176[illegible]	-0,12441	0,129459	0,336567
Ht	Mitral	ebi-a-GCST	CkbBol	444474	rs3685014[illegible]	-0,11876	0,128511	0,355435
Ht	Mitral	ebi-a-GCST	CkbBol	444474	rs3745471	-0,11693	0,12876	0,363818
Ht	Mitral	ebi-a-GCST	CkbBol	444474	rs3754140	-0,12221	0,129303	0,344572
Ht	Mitral	ebi-a-GCST	CkbBol	444474	rs3764573[illegible]	-0,10352	0,127962	0,418516
Ht	Mitral	ebi-a-GCST	CkbBol	444474	rs3791020	-0,10909	0,12867	0,396529
Ht	Mitral	ebi-a-GCST	CkbBol	444474	rs3809627	-0,11829	0,12899	0,359124
Ht	Mitral	ebi-a-GCST	CkbBol	444474	rs3809770	-0,11194	0,128795	0,384788
Ht	Mitral	ebi-a-GCST	CkbBol	444474	rs3811444	-0,11414	0,128853	0,375723
Ht	Mitral	ebi-a-GCST	CkbBol	444474	rs3847057	-0,10607	0,128684	0,409775
Ht	Mitral	ebi-a-GCST	CkbBol	444474	rs3859158	-0,1146	0,128866	0,373829
Ht	Mitral	ebi-a-GCST	CkbBol	444474	rs3932815	-0,11238	0,128873	0,383218
Ht	Mitral	ebi-a-GCST	CkbBol	444474	rs395379	-0,1127	0,128843	0,381734
Ht	Mitral	ebi-a-GCST	CkbBol	444474	rs4075958	-0,11501	0,129066	0,372872
Ht	Mitral	ebi-a-GCST	CkbBol	444474	rs4082052	-0,11205	0,12886	0,38456
Ht	Mitral	ebi-a-GCST	CkbBol	444474	rs4103	-0,10937	0,128585	0,394992
Ht	Mitral	ebi-a-GCST	CkbBol	444474	rs4126804[illegible]	-0,11248	0,128816	0,38255
Ht	Mitral	ebi-a-GCST	CkbBol	444474	rs4127110[illegible]	-0,11444	0,128892	0,374625
Ht	Mitral	ebi-a-GCST	CkbBol	444474	rs4127817[illegible]	-0,11629	0,128871	0,366846
Ht	Mitral	ebi-a-GCST	CkbBol	444474	rs4136494[illegible]	-0,11279	0,128838	0,381357
Ht	Mitral	ebi-a-GCST	CkbBol	444474	rs4141740	-0,10687	0,12854	0,405732
Ht	Mitral	ebi-a-GCST	CkbBol	444474	rs4233199	-0,11475	0,128861	0,373192
Ht	Mitral	ebi-a-GCST	CkbBol	444474	rs4233937	-0,11446	0,128875	0,374471
Ht	Mitral	ebi-a-GCST	CkbBol	444474	rs4240624	-0,10663	0,128662	0,407251
Ht	Mitral	ebi-a-GCST	CkbBol	444474	rs4522556	-0,11664	0,12881	0,365204
Ht	Mitral	ebi-a-GCST	CkbBol	444474	rs460879	-0,11347	0,129096	0,379434
Ht	Mitral	ebi-a-GCST	CkbBol	444474	rs4655791	-0,11436	0,128859	0,374824
Ht	Mitral	ebi-a-GCST	CkbBol	444474	rs4677156	-0,12442	0,127506	0,329172
Ht	Mitral	ebi-a-GCST	CkbBol	444474	rs4681673	-0,11807	0,128886	0,359627
Ht	Mitral	ebi-a-GCST	CkbBol	444474	rs4715354	-0,11375	0,128983	0,377812
Ht	Mitral	ebi-a-GCST	CkbBol	444474	rs4745984	-0,11542	0,128846	0,370371
Ht	Mitral	ebi-a-GCST	CkbBol	444474	rs4760682	-0,09162	0,128968	0,477443
Ht	Mitral	ebi-a-GCST	CkbBol	444474	rs4765552	-0,12012	0,128413	0,349572
Ht	Mitral	ebi-a-GCST	CkbBol	444474	rs4783719	-0,11928	0,128591	0,353615
Ht	Mitral	ebi-a-GCST	CkbBol	444474	rs4804981	-0,11289	0,128838	0,380901

Ht	Mitral	ebi-a-GCST CkbBol	444474	rs4820091	-0,11261	0,128912	0,382363
Ht	Mitral	ebi-a-GCST CkbBol	444474	rs485073	-0,12053	0,128331	0,347634
Ht	Mitral	ebi-a-GCST CkbBol	444474	rs4886755	-0,12325	0,129238	0,34025
Ht	Mitral	ebi-a-GCST CkbBol	444474	rs4985717	-0,12647	0,127421	0,320936
Ht	Mitral	ebi-a-GCST CkbBol	444474	rs498936	-0,11646	0,128834	0,36603
Ht	Mitral	ebi-a-GCST CkbBol	444474	rs5369	-0,10481	0,12847	0,414589
Ht	Mitral	ebi-a-GCST CkbBol	444474	rs548793	-0,10843	0,128474	0,398698
Ht	Mitral	ebi-a-GCST CkbBol	444474	rs550057	-0,09653	0,130306	0,458839
Ht	Mitral	ebi-a-GCST CkbBol	444474	rs5573197:	-0,11733	0,128992	0,363047
Ht	Mitral	ebi-a-GCST CkbBol	444474	rs5573329(	-0,11437	0,129113	0,375735
Ht	Mitral	ebi-a-GCST CkbBol	444474	rs559406	-0,12522	0,12815	0,328485
Ht	Mitral	ebi-a-GCST CkbBol	444474	rs5597144	-0,10792	0,128828	0,4022
Ht	Mitral	ebi-a-GCST CkbBol	444474	rs5601104	-0,11335	0,128948	0,379395
Ht	Mitral	ebi-a-GCST CkbBol	444474	rs5626110	-0,11105	0,129574	0,391432
Ht	Mitral	ebi-a-GCST CkbBol	444474	rs5635360	-0,11567	0,128826	0,369247
Ht	Mitral	ebi-a-GCST CkbBol	444474	rs565728	-0,11553	0,128846	0,369916
Ht	Mitral	ebi-a-GCST CkbBol	444474	rs5751348	-0,11354	0,129064	0,379021
Ht	Mitral	ebi-a-GCST CkbBol	444474	rs5854292(	-0,10953	0,128834	0,395221
Ht	Mitral	ebi-a-GCST CkbBol	444474	rs5863888	-0,12135	0,128345	0,344388
Ht	Mitral	ebi-a-GCST CkbBol	444474	rs5887955	-0,11005	0,1294	0,395066
Ht	Mitral	ebi-a-GCST CkbBol	444474	rs6054535	-0,11062	0,128777	0,390334
Ht	Mitral	ebi-a-GCST CkbBol	444474	rs6070139	-0,12787	0,12813	0,318278
Ht	Mitral	ebi-a-GCST CkbBol	444474	rs6112066	-0,10632	0,128098	0,406543
Ht	Mitral	ebi-a-GCST CkbBol	444474	rs6126017	-0,11124	0,128976	0,388411
Ht	Mitral	ebi-a-GCST CkbBol	444474	rs6134000	-0,11233	0,128846	0,383303
Ht	Mitral	ebi-a-GCST CkbBol	444474	rs6159113	-0,1222	0,128307	0,340881
Ht	Mitral	ebi-a-GCST CkbBol	444474	rs616111	-0,1122	0,12888	0,383992
Ht	Mitral	ebi-a-GCST CkbBol	444474	rs6175095	-0,12028	0,128896	0,350721
Ht	Mitral	ebi-a-GCST CkbBol	444474	rs6188066	-0,10384	0,128019	0,417278
Ht	Mitral	ebi-a-GCST CkbBol	444474	rs6205112(	-0,10943	0,128852	0,395713
Ht	Mitral	ebi-a-GCST CkbBol	444474	rs6213978	-0,11358	0,12888	0,378169
Ht	Mitral	ebi-a-GCST CkbBol	444474	rs6245916	-0,1127	0,128836	0,381702
Ht	Mitral	ebi-a-GCST CkbBol	444474	rs6415788	-0,10744	0,128838	0,404313
Ht	Mitral	ebi-a-GCST CkbBol	444474	rs6462990	-0,10968	0,128639	0,393887
Ht	Mitral	ebi-a-GCST CkbBol	444474	rs6474401	-0,11581	0,128881	0,368883
Ht	Mitral	ebi-a-GCST CkbBol	444474	rs654698	-0,1176	0,128725	0,360922
Ht	Mitral	ebi-a-GCST CkbBol	444474	rs6574100	-0,11029	0,128809	0,391869
Ht	Mitral	ebi-a-GCST CkbBol	444474	rs659185	-0,10145	0,128649	0,430362
Ht	Mitral	ebi-a-GCST CkbBol	444474	rs6598541	-0,11543	0,128831	0,370254
Ht	Mitral	ebi-a-GCST CkbBol	444474	rs6602909	-0,123	0,128573	0,338724
Ht	Mitral	ebi-a-GCST CkbBol	444474	rs6650529	-0,11187	0,12881	0,385114
Ht	Mitral	ebi-a-GCST CkbBol	444474	rs6660288	-0,108	0,128619	0,401077
Ht	Mitral	ebi-a-GCST CkbBol	444474	rs6666223	-0,10637	0,128211	0,406719
Ht	Mitral	ebi-a-GCST CkbBol	444474	rs6733795	-0,11709	0,128745	0,363111
Ht	Mitral	ebi-a-GCST CkbBol	444474	rs6742339	-0,11673	0,12884	0,36492
Ht	Mitral	ebi-a-GCST CkbBol	444474	rs6744374	-0,11491	0,128844	0,372455
Ht	Mitral	ebi-a-GCST CkbBol	444474	rs6804756(	-0,11623	0,128844	0,366991
Ht	Mitral	ebi-a-GCST CkbBol	444474	rs6818353	-0,11945	0,128574	0,352852
Ht	Mitral	ebi-a-GCST CkbBol	444474	rs6972333	-0,11675	0,128782	0,364634
Ht	Mitral	ebi-a-GCST CkbBol	444474	rs7030248	-0,12575	0,128433	0,327525

Ht	Mitral	ebi-a-GCST	CkbBol	444474	rs7032549	-0,11777	0,128881	0,360848
Ht	Mitral	ebi-a-GCST	CkbBol	444474	rs7088969	-0,11553	0,128848	0,369931
Ht	Mitral	ebi-a-GCST	CkbBol	444474	rs7091301	-0,10802	0,128413	0,40023
Ht	Mitral	ebi-a-GCST	CkbBol	444474	rs7148623	-0,11846	0,128624	0,357059
Ht	Mitral	ebi-a-GCST	CkbBol	444474	rs7182622	-0,11718	0,128751	0,362738
Ht	Mitral	ebi-a-GCST	CkbBol	444474	rs7247407	-0,11469	0,128868	0,373471
Ht	Mitral	ebi-a-GCST	CkbBol	444474	rs7258534	-0,11516	0,128853	0,371475
Ht	Mitral	ebi-a-GCST	CkbBol	444474	rs7271743	-0,12108	0,128704	0,346827
Ht	Mitral	ebi-a-GCST	CkbBol	444474	rs7273059	-0,1198	0,128815	0,352381
Ht	Mitral	ebi-a-GCST	CkbBol	444474	rs7275288	-0,13697	0,127889	0,284162
Ht	Mitral	ebi-a-GCST	CkbBol	444474	rs7276009	-0,12559	0,128349	0,32783
Ht	Mitral	ebi-a-GCST	CkbBol	444474	rs7276206	-0,11307	0,128913	0,380446
Ht	Mitral	ebi-a-GCST	CkbBol	444474	rs7319798	-0,11242	0,128827	0,382864
Ht	Mitral	ebi-a-GCST	CkbBol	444474	rs7386656	-0,10826	0,128498	0,399518
Ht	Mitral	ebi-a-GCST	CkbBol	444474	rs7441457	-0,11552	0,128871	0,370028
Ht	Mitral	ebi-a-GCST	CkbBol	444474	rs7462614	-0,10689	0,128542	0,405651
Ht	Mitral	ebi-a-GCST	CkbBol	444474	rs7485603	-0,11116	0,128733	0,387871
Ht	Mitral	ebi-a-GCST	CkbBol	444474	rs7525164	-0,12257	0,128802	0,341286
Ht	Mitral	ebi-a-GCST	CkbBol	444474	rs752590	-0,12228	0,128901	0,342796
Ht	Mitral	ebi-a-GCST	CkbBol	444474	rs7550758	-0,12842	0,127365	0,313315
Ht	Mitral	ebi-a-GCST	CkbBol	444474	rs7554456	-0,11603	0,128878	0,367964
Ht	Mitral	ebi-a-GCST	CkbBol	444474	rs7556629	-0,10952	0,128704	0,39482
Ht	Mitral	ebi-a-GCST	CkbBol	444474	rs757869	-0,11444	0,128901	0,374619
Ht	Mitral	ebi-a-GCST	CkbBol	444474	rs7579264	-0,10133	0,128957	0,432025
Ht	Mitral	ebi-a-GCST	CkbBol	444474	rs7579941	-0,10949	0,128717	0,394975
Ht	Mitral	ebi-a-GCST	CkbBol	444474	rs7583880	-0,11962	0,12879	0,353009
Ht	Mitral	ebi-a-GCST	CkbBol	444474	rs7587636	-0,10985	0,128742	0,393522
Ht	Mitral	ebi-a-GCST	CkbBol	444474	rs7589807	-0,11195	0,128816	0,384802
Ht	Mitral	ebi-a-GCST	CkbBol	444474	rs760077	-0,11873	0,129317	0,35856
Ht	Mitral	ebi-a-GCST	CkbBol	444474	rs7625643	-0,12091	0,128543	0,346893
Ht	Mitral	ebi-a-GCST	CkbBol	444474	rs7629106	-0,11443	0,128855	0,374498
Ht	Mitral	ebi-a-GCST	CkbBol	444474	rs7676376	-0,11594	0,128819	0,368129
Ht	Mitral	ebi-a-GCST	CkbBol	444474	rs7697279	-0,11747	0,128709	0,361393
Ht	Mitral	ebi-a-GCST	CkbBol	444474	rs7737147	-0,11313	0,128849	0,379931
Ht	Mitral	ebi-a-GCST	CkbBol	444474	rs7754216	-0,1183	0,128992	0,359095
Ht	Mitral	ebi-a-GCST	CkbBol	444474	rs7768871	-0,10829	0,128773	0,400374
Ht	Mitral	ebi-a-GCST	CkbBol	444474	rs7782121	-0,11384	0,12889	0,377129
Ht	Mitral	ebi-a-GCST	CkbBol	444474	rs7801581	-0,11899	0,128515	0,354503
Ht	Mitral	ebi-a-GCST	CkbBol	444474	rs791270	-0,114	0,128884	0,376437
Ht	Mitral	ebi-a-GCST	CkbBol	444474	rs7918480	-0,11058	0,128769	0,390467
Ht	Mitral	ebi-a-GCST	CkbBol	444474	rs7928717	-0,11722	0,128797	0,362748
Ht	Mitral	ebi-a-GCST	CkbBol	444474	rs7943988	-0,1142	0,128879	0,375567
Ht	Mitral	ebi-a-GCST	CkbBol	444474	rs7971133	-0,10564	0,128467	0,410878
Ht	Mitral	ebi-a-GCST	CkbBol	444474	rs797192	-0,10942	0,128681	0,395163
Ht	Mitral	ebi-a-GCST	CkbBol	444474	rs797343	-0,10799	0,128805	0,40182
Ht	Mitral	ebi-a-GCST	CkbBol	444474	rs7975576	-0,11778	0,12882	0,360546
Ht	Mitral	ebi-a-GCST	CkbBol	444474	rs8000803	-0,11271	0,128867	0,381794
Ht	Mitral	ebi-a-GCST	CkbBol	444474	rs800881	-0,11818	0,128938	0,359379
Ht	Mitral	ebi-a-GCST	CkbBol	444474	rs8060857	-0,11285	0,128876	0,38121
Ht	Mitral	ebi-a-GCST	CkbBol	444474	rs8115555	-0,11509	0,12886	0,371794

Ht	Mitral	ebi-a-GCST CkbBol	444474	rs8116919	-0,11687	0,128777	0,364129
Ht	Mitral	ebi-a-GCST CkbBol	444474	rs833061	-0,11588	0,129253	0,369963
Ht	Mitral	ebi-a-GCST CkbBol	444474	rs833805	-0,10613	0,129433	0,412254
Ht	Mitral	ebi-a-GCST CkbBol	444474	rs837763	-0,10795	0,129127	0,403163
Ht	Mitral	ebi-a-GCST CkbBol	444474	rs847821	-0,1112	0,128778	0,387849
Ht	Mitral	ebi-a-GCST CkbBol	444474	rs853199	-0,11083	0,128809	0,389547
Ht	Mitral	ebi-a-GCST CkbBol	444474	rs855791	-0,09958	0,130846	0,446633
Ht	Mitral	ebi-a-GCST CkbBol	444474	rs863678	-0,11395	0,129058	0,377267
Ht	Mitral	ebi-a-GCST CkbBol	444474	rs866929	-0,10821	0,128682	0,400394
Ht	Mitral	ebi-a-GCST CkbBol	444474	rs871841	-0,10973	0,128991	0,394961
Ht	Mitral	ebi-a-GCST CkbBol	444474	rs909236	-0,11104	0,128754	0,388438
Ht	Mitral	ebi-a-GCST CkbBol	444474	rs9288762	-0,11666	0,128826	0,365184
Ht	Mitral	ebi-a-GCST CkbBol	444474	rs9302635	-0,11876	0,128737	0,356285
Ht	Mitral	ebi-a-GCST CkbBol	444474	rs9320278	-0,11231	0,12889	0,383546
Ht	Mitral	ebi-a-GCST CkbBol	444474	rs9327454	-0,11401	0,128923	0,376528
Ht	Mitral	ebi-a-GCST CkbBol	444474	rs9370102	-0,11267	0,128849	0,38188
Ht	Mitral	ebi-a-GCST CkbBol	444474	rs9400888	-0,12682	0,12736	0,319356
Ht	Mitral	ebi-a-GCST CkbBol	444474	rs9420446	-0,11442	0,128893	0,374684
Ht	Mitral	ebi-a-GCST CkbBol	444474	rs9505097	-0,11941	0,128996	0,354592
Ht	Mitral	ebi-a-GCST CkbBol	444474	rs9526465	-0,11746	0,128707	0,36146
Ht	Mitral	ebi-a-GCST CkbBol	444474	rs9633514	-0,13467	0,127581	0,291162
Ht	Mitral	ebi-a-GCST CkbBol	444474	rs9639026	-0,11516	0,12884	0,371405
Ht	Mitral	ebi-a-GCST CkbBol	444474	rs9645770	-0,10923	0,128656	0,395869
Ht	Mitral	ebi-a-GCST CkbBol	444474	rs972762	-0,11752	0,128862	0,36176
Ht	Mitral	ebi-a-GCST CkbBol	444474	rs9771385	-0,12	0,128898	0,35187
Ht	Mitral	ebi-a-GCST CkbBol	444474	rs9784210	-0,10759	0,128317	0,401782
Ht	Mitral	ebi-a-GCST CkbBol	444474	rs9806453	-0,10504	0,128468	0,413585
Ht	Mitral	ebi-a-GCST CkbBol	444474	rs9816588	-0,11405	0,129006	0,376654
Ht	Mitral	ebi-a-GCST CkbBol	444474	rs988397	-0,10967	0,128926	0,394976
Ht	Mitral	ebi-a-GCST CkbBol	444474	rs9895661	-0,11505	0,129106	0,372841
Ht	Mitral	ebi-a-GCST CkbBol	444474	rs999010	-0,11087	0,129139	0,390607
Ht	Mitral	ebi-a-GCST CkbBol	444474	All	-0,11404	0,128606	0,37521
Hb	Mitral	ebi-a-GCST fkLGOH	430885	rs1011173[illegible]	-0,08991	0,117763	0,4452
Hb	Mitral	ebi-a-GCST fkLGOH	430885	rs1016834[illegible]	-0,06079	0,119315	0,610417
Hb	Mitral	ebi-a-GCST fkLGOH	430885	rs1026522[illegible]	-0,08001	0,119156	0,501902
Hb	Mitral	ebi-a-GCST fkLGOH	430885	rs1042312[illegible]	-0,09031	0,117733	0,443015
Hb	Mitral	ebi-a-GCST fkLGOH	430885	rs1043893	-0,08808	0,117858	0,454862
Hb	Mitral	ebi-a-GCST fkLGOH	430885	rs1055253	-0,08765	0,117866	0,457078
Hb	Mitral	ebi-a-GCST fkLGOH	430885	rs1060257	-0,08202	0,117607	0,485538
Hb	Mitral	ebi-a-GCST fkLGOH	430885	rs1062601	-0,10847	0,116315	0,351043
Hb	Mitral	ebi-a-GCST fkLGOH	430885	rs1075871	-0,09453	0,117695	0,421892
Hb	Mitral	ebi-a-GCST fkLGOH	430885	rs1077954[illegible]	-0,078	0,116794	0,504257
Hb	Mitral	ebi-a-GCST fkLGOH	430885	rs1077959[illegible]	-0,08912	0,117873	0,449599
Hb	Mitral	ebi-a-GCST fkLGOH	430885	rs1080784[illegible]	-0,08665	0,117866	0,462263
Hb	Mitral	ebi-a-GCST fkLGOH	430885	rs1081221[illegible]	-0,08487	0,117784	0,471158
Hb	Mitral	ebi-a-GCST fkLGOH	430885	rs1081749[illegible]	-0,09681	0,11737	0,409456
Hb	Mitral	ebi-a-GCST fkLGOH	430885	rs1081946[illegible]	-0,08583	0,117862	0,466453
Hb	Mitral	ebi-a-GCST fkLGOH	430885	rs1084014[illegible]	-0,08501	0,118069	0,471532
Hb	Mitral	ebi-a-GCST fkLGOH	430885	rs1084045[illegible]	-0,09036	0,117884	0,443384
Hb	Mitral	ebi-a-GCST fkLGOH	430885	rs1085904[illegible]	-0,08002	0,117382	0,495422

Hb	Mitral	ebi-a-GCST	fkLGOH	430885	rs1089897	-0,08859	0,117853	0,452254
Hb	Mitral	ebi-a-GCST	fkLGOH	430885	rs1090002	-0,0926	0,117778	0,431749
Hb	Mitral	ebi-a-GCST	fkLGOH	430885	rs1090408	-0,09202	0,117723	0,434401
Hb	Mitral	ebi-a-GCST	fkLGOH	430885	rs1091467	-0,08574	0,11784	0,46686
Hb	Mitral	ebi-a-GCST	fkLGOH	430885	rs1095693	-0,09097	0,117993	0,440714
Hb	Mitral	ebi-a-GCST	fkLGOH	430885	rs1095860	-0,07958	0,117323	0,497586
Hb	Mitral	ebi-a-GCST	fkLGOH	430885	rs1105637	-0,08701	0,117889	0,460461
Hb	Mitral	ebi-a-GCST	fkLGOH	430885	rs1109825	-0,09627	0,117346	0,412014
Hb	Mitral	ebi-a-GCST	fkLGOH	430885	rs1110542	-0,08028	0,11749	0,494395
Hb	Mitral	ebi-a-GCST	fkLGOH	430885	rs1112492	-0,09023	0,117907	0,444104
Hb	Mitral	ebi-a-GCST	fkLGOH	430885	rs1112666	-0,0906	0,117726	0,441544
Hb	Mitral	ebi-a-GCST	fkLGOH	430885	rs1119794	-0,08432	0,117766	0,474003
Hb	Mitral	ebi-a-GCST	fkLGOH	430885	rs1121384	-0,08286	0,117792	0,4818
Hb	Mitral	ebi-a-GCST	fkLGOH	430885	rs1125242	-0,09124	0,117641	0,437976
Hb	Mitral	ebi-a-GCST	fkLGOH	430885	rs1125386	-0,09023	0,117832	0,443801
Hb	Mitral	ebi-a-GCST	fkLGOH	430885	rs1125853	-0,08298	0,117763	0,481014
Hb	Mitral	ebi-a-GCST	fkLGOH	430885	rs1125975	-0,08181	0,117714	0,487079
Hb	Mitral	ebi-a-GCST	fkLGOH	430885	rs1130511	-0,08338	0,117756	0,478903
Hb	Mitral	ebi-a-GCST	fkLGOH	430885	rs1136701	-0,08818	0,117923	0,454586
Hb	Mitral	ebi-a-GCST	fkLGOH	430885	rs1149225	-0,09162	0,11764	0,436099
Hb	Mitral	ebi-a-GCST	fkLGOH	430885	rs1155692	-0,08772	0,117916	0,456928
Hb	Mitral	ebi-a-GCST	fkLGOH	430885	rs1159862	-0,08169	0,117958	0,4886
Hb	Mitral	ebi-a-GCST	fkLGOH	430885	rs1161548	-0,08104	0,117696	0,491102
Hb	Mitral	ebi-a-GCST	fkLGOH	430885	rs1164111	-0,08686	0,117958	0,461501
Hb	Mitral	ebi-a-GCST	fkLGOH	430885	rs1166758	-0,07995	0,117636	0,496726
Hb	Mitral	ebi-a-GCST	fkLGOH	430885	rs1167124	-0,08482	0,117796	0,471465
Hb	Mitral	ebi-a-GCST	fkLGOH	430885	rs1168601	-0,08556	0,117838	0,467775
Hb	Mitral	ebi-a-GCST	fkLGOH	430885	rs1169057	-0,08474	0,117813	0,471987
Hb	Mitral	ebi-a-GCST	fkLGOH	430885	rs1169490	-0,08904	0,117965	0,450383
Hb	Mitral	ebi-a-GCST	fkLGOH	430885	rs1170472	-0,08624	0,117862	0,464363
Hb	Mitral	ebi-a-GCST	fkLGOH	430885	rs1172001	-0,08213	0,117667	0,485197
Hb	Mitral	ebi-a-GCST	fkLGOH	430885	rs1175550	-0,07858	0,117868	0,504974
Hb	Mitral	ebi-a-GCST	fkLGOH	430885	rs1175870	-0,07674	0,11754	0,51382
Hb	Mitral	ebi-a-GCST	fkLGOH	430885	rs1178409	-0,0856	0,117858	0,467633
Hb	Mitral	ebi-a-GCST	fkLGOH	430885	rs1179705	-0,08634	0,117861	0,463816
Hb	Mitral	ebi-a-GCST	fkLGOH	430885	rs1197088	-0,09226	0,117788	0,433492
Hb	Mitral	ebi-a-GCST	fkLGOH	430885	rs1197893	-0,09231	0,117542	0,432275
Hb	Mitral	ebi-a-GCST	fkLGOH	430885	rs1202534	-0,08393	0,117777	0,476101
Hb	Mitral	ebi-a-GCST	fkLGOH	430885	rs1212988	-0,0923	0,117543	0,4323
Hb	Mitral	ebi-a-GCST	fkLGOH	430885	rs1219267	-0,0865	0,118034	0,46366
Hb	Mitral	ebi-a-GCST	fkLGOH	430885	rs1226578	-0,08779	0,11787	0,456387
Hb	Mitral	ebi-a-GCST	fkLGOH	430885	rs1232597	-0,0812	0,117377	0,48905
Hb	Mitral	ebi-a-GCST	fkLGOH	430885	rs123698	-0,08207	0,118043	0,48692
Hb	Mitral	ebi-a-GCST	fkLGOH	430885	rs1239682	-0,09444	0,117523	0,42162
Hb	Mitral	ebi-a-GCST	fkLGOH	430885	rs1256061	-0,10284	0,117264	0,380511
Hb	Mitral	ebi-a-GCST	fkLGOH	430885	rs1257415	-0,08606	0,117888	0,465376
Hb	Mitral	ebi-a-GCST	fkLGOH	430885	rs1258495	-0,08869	0,117864	0,451759
Hb	Mitral	ebi-a-GCST	fkLGOH	430885	rs1271873	-0,08513	0,11782	0,46998
Hb	Mitral	ebi-a-GCST	fkLGOH	430885	rs1274278	-0,07391	0,116709	0,526549
Hb	Mitral	ebi-a-GCST	fkLGOH	430885	rs1280044	-0,08688	0,117877	0,461116

Hb	Mitral	ebi-a-GCST	fkLGOH	430885	rs1281151:	-0,08798	0,117999	0,45592
Hb	Mitral	ebi-a-GCST	fkLGOH	430885	rs128494	-0,08798	0,118087	0,456222
Hb	Mitral	ebi-a-GCST	fkLGOH	430885	rs1288186!	-0,08406	0,117815	0,475523
Hb	Mitral	ebi-a-GCST	fkLGOH	430885	rs1288926:	-0,09194	0,117804	0,435129
Hb	Mitral	ebi-a-GCST	fkLGOH	430885	rs1289435	-0,08781	0,117882	0,456337
Hb	Mitral	ebi-a-GCST	fkLGOH	430885	rs1290784	-0,08204	0,117805	0,486183
Hb	Mitral	ebi-a-GCST	fkLGOH	430885	rs1296773	-0,0843	0,117824	0,47434
Hb	Mitral	ebi-a-GCST	fkLGOH	430885	rs1298766:	-0,09198	0,117657	0,434331
Hb	Mitral	ebi-a-GCST	fkLGOH	430885	rs1300528:	-0,08616	0,117874	0,464835
Hb	Mitral	ebi-a-GCST	fkLGOH	430885	rs1300770!	-0,08419	0,117819	0,474858
Hb	Mitral	ebi-a-GCST	fkLGOH	430885	rs1308028:	-0,08501	0,117884	0,470853
Hb	Mitral	ebi-a-GCST	fkLGOH	430885	rs1310353	-0,08367	0,117837	0,477663
Hb	Mitral	ebi-a-GCST	fkLGOH	430885	rs1310732!	-0,09943	0,117645	0,397996
Hb	Mitral	ebi-a-GCST	fkLGOH	430885	rs1314582!	-0,09107	0,117719	0,439168
Hb	Mitral	ebi-a-GCST	fkLGOH	430885	rs1320735:	-0,08947	0,118201	0,449067
Hb	Mitral	ebi-a-GCST	fkLGOH	430885	rs1330678(	-0,09271	0,117912	0,431711
Hb	Mitral	ebi-a-GCST	fkLGOH	430885	rs1337628!	-0,09016	0,117833	0,444201
Hb	Mitral	ebi-a-GCST	fkLGOH	430885	rs1338921!	-0,08502	0,117917	0,470907
Hb	Mitral	ebi-a-GCST	fkLGOH	430885	rs1339653!	-0,08829	0,117858	0,453795
Hb	Mitral	ebi-a-GCST	fkLGOH	430885	rs1340124:	-0,08339	0,117783	0,478947
Hb	Mitral	ebi-a-GCST	fkLGOH	430885	rs1340817	-0,08134	0,117855	0,490101
Hb	Mitral	ebi-a-GCST	fkLGOH	430885	rs1341555(	-0,08802	0,117867	0,455177
Hb	Mitral	ebi-a-GCST	fkLGOH	430885	rs1354674	-0,08789	0,117897	0,455954
Hb	Mitral	ebi-a-GCST	fkLGOH	430885	rs13621	-0,08652	0,117921	0,463121
Hb	Mitral	ebi-a-GCST	fkLGOH	430885	rs1373866	-0,08062	0,117308	0,491908
Hb	Mitral	ebi-a-GCST	fkLGOH	430885	rs1380582!	-0,09706	0,116781	0,405905
Hb	Mitral	ebi-a-GCST	fkLGOH	430885	rs1390783:	-0,08717	0,117874	0,459568
Hb	Mitral	ebi-a-GCST	fkLGOH	430885	rs1399562	-0,08569	0,117871	0,467253
Hb	Mitral	ebi-a-GCST	fkLGOH	430885	rs1414946(	-0,08902	0,117826	0,449958
Hb	Mitral	ebi-a-GCST	fkLGOH	430885	rs1420624(	-0,09444	0,117725	0,422435
Hb	Mitral	ebi-a-GCST	fkLGOH	430885	rs1426374	-0,08178	0,117628	0,486879
Hb	Mitral	ebi-a-GCST	fkLGOH	430885	rs1427445	-0,0819	0,117835	0,487051
Hb	Mitral	ebi-a-GCST	fkLGOH	430885	rs1438577:	-0,09084	0,117873	0,440914
Hb	Mitral	ebi-a-GCST	fkLGOH	430885	rs1439916:	-0,09353	0,117718	0,426898
Hb	Mitral	ebi-a-GCST	fkLGOH	430885	rs1472329!	-0,09064	0,117668	0,441107
Hb	Mitral	ebi-a-GCST	fkLGOH	430885	rs1474138	-0,0916	0,117615	0,436106
Hb	Mitral	ebi-a-GCST	fkLGOH	430885	rs1479559	-0,09236	0,117789	0,43299
Hb	Mitral	ebi-a-GCST	fkLGOH	430885	rs1480839	-0,08348	0,117724	0,478242
Hb	Mitral	ebi-a-GCST	fkLGOH	430885	rs1484621(	-0,09242	0,11764	0,432078
Hb	Mitral	ebi-a-GCST	fkLGOH	430885	rs1489674(	-0,0838	0,117755	0,476707
Hb	Mitral	ebi-a-GCST	fkLGOH	430885	rs1508443(	-0,08016	0,118026	0,497011
Hb	Mitral	ebi-a-GCST	fkLGOH	430885	rs1611236	-0,08232	0,117767	0,484554
Hb	Mitral	ebi-a-GCST	fkLGOH	430885	rs165944	-0,08504	0,117841	0,470497
Hb	Mitral	ebi-a-GCST	fkLGOH	430885	rs1678960	-0,09002	0,117764	0,444627
Hb	Mitral	ebi-a-GCST	fkLGOH	430885	rs1687406(	-0,0859	0,117957	0,466494
Hb	Mitral	ebi-a-GCST	fkLGOH	430885	rs1690233!	-0,08459	0,117772	0,472584
Hb	Mitral	ebi-a-GCST	fkLGOH	430885	rs1690997(	-0,08817	0,117864	0,45444
Hb	Mitral	ebi-a-GCST	fkLGOH	430885	rs1694275:	-0,07828	0,11673	0,502477
Hb	Mitral	ebi-a-GCST	fkLGOH	430885	rs1700342:	-0,08393	0,117774	0,476053
Hb	Mitral	ebi-a-GCST	fkLGOH	430885	rs1700644:	-0,09525	0,117775	0,418667

Hb	Mitral	ebi-a-GCST fkLGOH	430885	rs1718513!	-0,08532	0,117836	0,469038
Hb	Mitral	ebi-a-GCST fkLGOH	430885	rs1727840(	-0,08861	0,118173	0,453358
Hb	Mitral	ebi-a-GCST fkLGOH	430885	rs174574	-0,08748	0,118117	0,458907
Hb	Mitral	ebi-a-GCST fkLGOH	430885	rs1747636‹	-0,0558	0,120742	0,643952
Hb	Mitral	ebi-a-GCST fkLGOH	430885	rs1765474:	-0,08241	0,117868	0,484457
Hb	Mitral	ebi-a-GCST fkLGOH	430885	rs1768022!	-0,08854	0,117919	0,452718
Hb	Mitral	ebi-a-GCST fkLGOH	430885	rs1781669:	-0,09028	0,117844	0,443629
Hb	Mitral	ebi-a-GCST fkLGOH	430885	rs1800562	-0,087	0,121599	0,474308
Hb	Mitral	ebi-a-GCST fkLGOH	430885	rs1800961	-0,07437	0,117529	0,526858
Hb	Mitral	ebi-a-GCST fkLGOH	430885	rs181669	-0,09027	0,117765	0,443368
Hb	Mitral	ebi-a-GCST fkLGOH	430885	rs1840885:	-0,08959	0,118164	0,448347
Hb	Mitral	ebi-a-GCST fkLGOH	430885	rs1855557	-0,08308	0,117726	0,480388
Hb	Mitral	ebi-a-GCST fkLGOH	430885	rs1863127	-0,08491	0,117851	0,471245
Hb	Mitral	ebi-a-GCST fkLGOH	430885	rs1868274	-0,09881	0,11767	0,401045
Hb	Mitral	ebi-a-GCST fkLGOH	430885	rs1874228	-0,09142	0,117826	0,437803
Hb	Mitral	ebi-a-GCST fkLGOH	430885	rs1923032	-0,08792	0,117875	0,455754
Hb	Mitral	ebi-a-GCST fkLGOH	430885	rs1924930	-0,08466	0,117807	0,472379
Hb	Mitral	ebi-a-GCST fkLGOH	430885	rs1926704!	-0,08968	0,11785	0,446691
Hb	Mitral	ebi-a-GCST fkLGOH	430885	rs1949481	-0,08418	0,117755	0,474707
Hb	Mitral	ebi-a-GCST fkLGOH	430885	rs1962310	-0,08733	0,117882	0,45878
Hb	Mitral	ebi-a-GCST fkLGOH	430885	rs1987070	-0,0887	0,117918	0,451907
Hb	Mitral	ebi-a-GCST fkLGOH	430885	rs1996802	-0,08901	0,117825	0,449971
Hb	Mitral	ebi-a-GCST fkLGOH	430885	rs2015803	-0,07998	0,117665	0,496675
Hb	Mitral	ebi-a-GCST fkLGOH	430885	rs2020971	-0,0865	0,117861	0,462988
Hb	Mitral	ebi-a-GCST fkLGOH	430885	rs2043082	-0,09203	0,117773	0,434538
Hb	Mitral	ebi-a-GCST fkLGOH	430885	rs2052284	-0,09905	0,117	0,397242
Hb	Mitral	ebi-a-GCST fkLGOH	430885	rs2060631	-0,09073	0,117704	0,440797
Hb	Mitral	ebi-a-GCST fkLGOH	430885	rs2070895	-0,08136	0,11776	0,489641
Hb	Mitral	ebi-a-GCST fkLGOH	430885	rs2093380	-0,08813	0,117892	0,454729
Hb	Mitral	ebi-a-GCST fkLGOH	430885	rs2117795	-0,09062	0,117761	0,441569
Hb	Mitral	ebi-a-GCST fkLGOH	430885	rs218264	-0,08139	0,118276	0,491371
Hb	Mitral	ebi-a-GCST fkLGOH	430885	rs2184540	-0,08631	0,11789	0,464089
Hb	Mitral	ebi-a-GCST fkLGOH	430885	rs2209098	-0,09078	0,117881	0,441234
Hb	Mitral	ebi-a-GCST fkLGOH	430885	rs2228445	-0,09026	0,118004	0,444341
Hb	Mitral	ebi-a-GCST fkLGOH	430885	rs2243314	-0,0852	0,118039	0,470416
Hb	Mitral	ebi-a-GCST fkLGOH	430885	rs2255293	-0,08906	0,117847	0,449832
Hb	Mitral	ebi-a-GCST fkLGOH	430885	rs2294915	-0,09228	0,117967	0,434085
Hb	Mitral	ebi-a-GCST fkLGOH	430885	rs2298428	-0,087	0,117885	0,460486
Hb	Mitral	ebi-a-GCST fkLGOH	430885	rs2303937	-0,08171	0,117559	0,487027
Hb	Mitral	ebi-a-GCST fkLGOH	430885	rs2339851	-0,08895	0,117916	0,450623
Hb	Mitral	ebi-a-GCST fkLGOH	430885	rs2354314	-0,08603	0,117875	0,4655
Hb	Mitral	ebi-a-GCST fkLGOH	430885	rs2399972	-0,0893	0,117864	0,448653
Hb	Mitral	ebi-a-GCST fkLGOH	430885	rs241812	-0,08472	0,117775	0,471948
Hb	Mitral	ebi-a-GCST fkLGOH	430885	rs2426160	-0,07823	0,117493	0,505503
Hb	Mitral	ebi-a-GCST fkLGOH	430885	rs2439422	-0,08855	0,117843	0,452381
Hb	Mitral	ebi-a-GCST fkLGOH	430885	rs2519093	-0,10542	0,119234	0,376614
Hb	Mitral	ebi-a-GCST fkLGOH	430885	rs2541119	-0,08853	0,117871	0,452627
Hb	Mitral	ebi-a-GCST fkLGOH	430885	rs2561566	-0,095	0,117617	0,419244
Hb	Mitral	ebi-a-GCST fkLGOH	430885	rs2576161	-0,08711	0,117875	0,459889
Hb	Mitral	ebi-a-GCST fkLGOH	430885	rs258753	-0,08779	0,117893	0,456456

Hb	Mitral	ebi-a-GCST	fkLGOH	430885	rs2645490	-0,09065	0,117974	0,442275
Hb	Mitral	ebi-a-GCST	fkLGOH	430885	rs2823139	-0,09519	0,117966	0,419727
Hb	Mitral	ebi-a-GCST	fkLGOH	430885	rs2834287	-0,08496	0,117965	0,471389
Hb	Mitral	ebi-a-GCST	fkLGOH	430885	rs2835435	-0,08387	0,118013	0,477287
Hb	Mitral	ebi-a-GCST	fkLGOH	430885	rs2843233(	-0,08494	0,118154	0,472206
Hb	Mitral	ebi-a-GCST	fkLGOH	430885	rs2860637(	-0,09011	0,117821	0,444362
Hb	Mitral	ebi-a-GCST	fkLGOH	430885	rs2861249,	-0,08225	0,117582	0,484225
Hb	Mitral	ebi-a-GCST	fkLGOH	430885	rs2864153:	-0,08059	0,117641	0,49331
Hb	Mitral	ebi-a-GCST	fkLGOH	430885	rs2870238	-0,08659	0,118071	0,463351
Hb	Mitral	ebi-a-GCST	fkLGOH	430885	rs2871974	-0,08868	0,117854	0,451772
Hb	Mitral	ebi-a-GCST	fkLGOH	430885	rs290671	-0,09125	0,117686	0,43812
Hb	Mitral	ebi-a-GCST	fkLGOH	430885	rs2934849	-0,08915	0,117855	0,44938
Hb	Mitral	ebi-a-GCST	fkLGOH	430885	rs2943637	-0,08852	0,117908	0,452795
Hb	Mitral	ebi-a-GCST	fkLGOH	430885	rs2950015	-0,08377	0,118384	0,479188
Hb	Mitral	ebi-a-GCST	fkLGOH	430885	rs2970870	-0,08298	0,117634	0,48054
Hb	Mitral	ebi-a-GCST	fkLGOH	430885	rs3112629	-0,09162	0,117634	0,436042
Hb	Mitral	ebi-a-GCST	fkLGOH	430885	rs3129430	-0,08753	0,117903	0,457864
Hb	Mitral	ebi-a-GCST	fkLGOH	430885	rs3184504	-0,08913	0,119481	0,455696
Hb	Mitral	ebi-a-GCST	fkLGOH	430885	rs3213545	-0,09301	0,117861	0,430035
Hb	Mitral	ebi-a-GCST	fkLGOH	430885	rs333947	-0,08525	0,117815	0,469308
Hb	Mitral	ebi-a-GCST	fkLGOH	430885	rs339995	-0,07931	0,117465	0,499547
Hb	Mitral	ebi-a-GCST	fkLGOH	430885	rs3423572(	-0,09165	0,117609	0,435806
Hb	Mitral	ebi-a-GCST	fkLGOH	430885	rs3441683,	-0,08342	0,117662	0,478363
Hb	Mitral	ebi-a-GCST	fkLGOH	430885	rs3462714{	-0,0954	0,116928	0,414554
Hb	Mitral	ebi-a-GCST	fkLGOH	430885	rs3493361:	-0,08353	0,117737	0,478041
Hb	Mitral	ebi-a-GCST	fkLGOH	430885	rs3510624,	-0,09188	0,117567	0,434481
Hb	Mitral	ebi-a-GCST	fkLGOH	430885	rs3512440(	-0,08664	0,118084	0,463122
Hb	Mitral	ebi-a-GCST	fkLGOH	430885	rs3521910	-0,08422	0,117763	0,474511
Hb	Mitral	ebi-a-GCST	fkLGOH	430885	rs3524099	-0,09494	0,117874	0,420551
Hb	Mitral	ebi-a-GCST	fkLGOH	430885	rs357282	-0,09221	0,117751	0,433556
Hb	Mitral	ebi-a-GCST	fkLGOH	430885	rs3606573:	-0,09839	0,117911	0,40402
Hb	Mitral	ebi-a-GCST	fkLGOH	430885	rs3684176:	-0,0956	0,11826	0,418873
Hb	Mitral	ebi-a-GCST	fkLGOH	430885	rs3740689	-0,08313	0,117787	0,480339
Hb	Mitral	ebi-a-GCST	fkLGOH	430885	rs3741414	-0,07777	0,11748	0,507962
Hb	Mitral	ebi-a-GCST	fkLGOH	430885	rs3745471	-0,09029	0,117794	0,443372
Hb	Mitral	ebi-a-GCST	fkLGOH	430885	rs3754140	-0,09408	0,118181	0,426
Hb	Mitral	ebi-a-GCST	fkLGOH	430885	rs3755397	-0,09037	0,117738	0,442741
Hb	Mitral	ebi-a-GCST	fkLGOH	430885	rs3772219	-0,09017	0,118035	0,44493
Hb	Mitral	ebi-a-GCST	fkLGOH	430885	rs3777891	-0,0814	0,11748	0,488373
Hb	Mitral	ebi-a-GCST	fkLGOH	430885	rs3780474	-0,09044	0,117907	0,443061
Hb	Mitral	ebi-a-GCST	fkLGOH	430885	rs3791020	-0,08274	0,117684	0,482002
Hb	Mitral	ebi-a-GCST	fkLGOH	430885	rs3791995	-0,09427	0,117619	0,42283
Hb	Mitral	ebi-a-GCST	fkLGOH	430885	rs3807997	-0,09005	0,11776	0,444439
Hb	Mitral	ebi-a-GCST	fkLGOH	430885	rs3809627	-0,09061	0,117933	0,442314
Hb	Mitral	ebi-a-GCST	fkLGOH	430885	rs3811444	-0,08753	0,118003	0,458216
Hb	Mitral	ebi-a-GCST	fkLGOH	430885	rs3814570	-0,09043	0,117756	0,442506
Hb	Mitral	ebi-a-GCST	fkLGOH	430885	rs382429	-0,08935	0,118025	0,449024
Hb	Mitral	ebi-a-GCST	fkLGOH	430885	rs3859158	-0,08781	0,117889	0,456362
Hb	Mitral	ebi-a-GCST	fkLGOH	430885	rs3895874	-0,08584	0,117841	0,46634
Hb	Mitral	ebi-a-GCST	fkLGOH	430885	rs3925798	-0,08724	0,117873	0,459207

Hb	Mitral	ebi-a-GCST fkLGOH	430885	rs4074995	-0,08893	0,11803	0,451192
Hb	Mitral	ebi-a-GCST fkLGOH	430885	rs4127817	-0,08943	0,11789	0,448105
Hb	Mitral	ebi-a-GCST fkLGOH	430885	rs4130742	-0,0881	0,118005	0,455299
Hb	Mitral	ebi-a-GCST fkLGOH	430885	rs4233937	-0,08765	0,117895	0,457203
Hb	Mitral	ebi-a-GCST fkLGOH	430885	rs4240624	-0,08056	0,117663	0,493582
Hb	Mitral	ebi-a-GCST fkLGOH	430885	rs4322206	-0,08739	0,117873	0,458434
Hb	Mitral	ebi-a-GCST fkLGOH	430885	rs4456287	-0,08314	0,117732	0,480087
Hb	Mitral	ebi-a-GCST fkLGOH	430885	rs4522556	-0,0897	0,117828	0,446495
Hb	Mitral	ebi-a-GCST fkLGOH	430885	rs464605	-0,08956	0,117903	0,447468
Hb	Mitral	ebi-a-GCST fkLGOH	430885	rs4660253	-0,0759	0,117234	0,517369
Hb	Mitral	ebi-a-GCST fkLGOH	430885	rs4677156	-0,09687	0,116593	0,406079
Hb	Mitral	ebi-a-GCST fkLGOH	430885	rs4683603	-0,09575	0,117267	0,414195
Hb	Mitral	ebi-a-GCST fkLGOH	430885	rs4715354	-0,08692	0,118007	0,461387
Hb	Mitral	ebi-a-GCST fkLGOH	430885	rs4745984	-0,08855	0,117864	0,452456
Hb	Mitral	ebi-a-GCST fkLGOH	430885	rs4760682	-0,06813	0,117786	0,562978
Hb	Mitral	ebi-a-GCST fkLGOH	430885	rs483180	-0,08971	0,11785	0,446525
Hb	Mitral	ebi-a-GCST fkLGOH	430885	rs4833291	-0,08217	0,117533	0,484448
Hb	Mitral	ebi-a-GCST fkLGOH	430885	rs4836845	-0,08183	0,117491	0,486112
Hb	Mitral	ebi-a-GCST fkLGOH	430885	rs4846923	-0,08376	0,117738	0,476844
Hb	Mitral	ebi-a-GCST fkLGOH	430885	rs4886755	-0,09539	0,118167	0,419501
Hb	Mitral	ebi-a-GCST fkLGOH	430885	rs4895441	-0,09138	0,118562	0,44088
Hb	Mitral	ebi-a-GCST fkLGOH	430885	rs4899258	-0,08627	0,117882	0,464247
Hb	Mitral	ebi-a-GCST fkLGOH	430885	rs4925184	-0,08792	0,11792	0,455904
Hb	Mitral	ebi-a-GCST fkLGOH	430885	rs4979174	-0,09296	0,117464	0,428741
Hb	Mitral	ebi-a-GCST fkLGOH	430885	rs5369	-0,07924	0,117473	0,499987
Hb	Mitral	ebi-a-GCST fkLGOH	430885	rs5573197	-0,09009	0,117967	0,445054
Hb	Mitral	ebi-a-GCST fkLGOH	430885	rs5573329	-0,08743	0,118075	0,459016
Hb	Mitral	ebi-a-GCST fkLGOH	430885	rs5578119	-0,08298	0,117945	0,481712
Hb	Mitral	ebi-a-GCST fkLGOH	430885	rs5591495	-0,08665	0,117889	0,462313
Hb	Mitral	ebi-a-GCST fkLGOH	430885	rs559406	-0,09728	0,117194	0,406514
Hb	Mitral	ebi-a-GCST fkLGOH	430885	rs5597144	-0,0813	0,11784	0,490264
Hb	Mitral	ebi-a-GCST fkLGOH	430885	rs5601104	-0,08657	0,117943	0,462948
Hb	Mitral	ebi-a-GCST fkLGOH	430885	rs5608561	-0,08449	0,117895	0,473599
Hb	Mitral	ebi-a-GCST fkLGOH	430885	rs564640	-0,08275	0,117695	0,481986
Hb	Mitral	ebi-a-GCST fkLGOH	430885	rs565728	-0,08866	0,117864	0,451914
Hb	Mitral	ebi-a-GCST fkLGOH	430885	rs5751348	-0,08668	0,118091	0,462966
Hb	Mitral	ebi-a-GCST fkLGOH	430885	rs5752720	-0,08386	0,117796	0,476512
Hb	Mitral	ebi-a-GCST fkLGOH	430885	rs5782885	-0,09166	0,117922	0,436975
Hb	Mitral	ebi-a-GCST fkLGOH	430885	rs5832428	-0,08614	0,117975	0,465291
Hb	Mitral	ebi-a-GCST fkLGOH	430885	rs5836526	-0,0845	0,117819	0,473229
Hb	Mitral	ebi-a-GCST fkLGOH	430885	rs5854292	-0,08342	0,117815	0,478926
Hb	Mitral	ebi-a-GCST fkLGOH	430885	rs5927198	-0,08697	0,117875	0,460647
Hb	Mitral	ebi-a-GCST fkLGOH	430885	rs5995385	-0,09406	0,117833	0,424716
Hb	Mitral	ebi-a-GCST fkLGOH	430885	rs599908	-0,08756	0,11787	0,457586
Hb	Mitral	ebi-a-GCST fkLGOH	430885	rs6016505	-0,08805	0,117888	0,455126
Hb	Mitral	ebi-a-GCST fkLGOH	430885	rs6173955	-0,08609	0,118181	0,466321
Hb	Mitral	ebi-a-GCST fkLGOH	430885	rs6175095	-0,09299	0,117895	0,430267
Hb	Mitral	ebi-a-GCST fkLGOH	430885	rs6188066	-0,07784	0,117064	0,506099
Hb	Mitral	ebi-a-GCST fkLGOH	430885	rs6213978	-0,08682	0,117885	0,461457
Hb	Mitral	ebi-a-GCST fkLGOH	430885	rs636202	-0,0854	0,117893	0,468848

Hb	Mitral	ebi-a-GCST fkLGOH	430885	rs6415788	-0,08082	0,117849	0,492853
Hb	Mitral	ebi-a-GCST fkLGOH	430885	rs6428637	-0,0894	0,117837	0,448062
Hb	Mitral	ebi-a-GCST fkLGOH	430885	rs643884	-0,08968	0,117863	0,446713
Hb	Mitral	ebi-a-GCST fkLGOH	430885	rs6451719	-0,09221	0,117557	0,432816
Hb	Mitral	ebi-a-GCST fkLGOH	430885	rs6465351	-0,09471	0,117632	0,420723
Hb	Mitral	ebi-a-GCST fkLGOH	430885	rs6474401	-0,09005	0,118042	0,445543
Hb	Mitral	ebi-a-GCST fkLGOH	430885	rs6547599	-0,08795	0,117861	0,455546
Hb	Mitral	ebi-a-GCST fkLGOH	430885	rs6602909	-0,0961	0,117614	0,413883
Hb	Mitral	ebi-a-GCST fkLGOH	430885	rs6651436	-0,08544	0,117826	0,468384
Hb	Mitral	ebi-a-GCST fkLGOH	430885	rs6653062	-0,075	0,116778	0,520715
Hb	Mitral	ebi-a-GCST fkLGOH	430885	rs6678517	-0,08599	0,117857	0,465628
Hb	Mitral	ebi-a-GCST fkLGOH	430885	rs6679233	-0,08272	0,117631	0,481913
Hb	Mitral	ebi-a-GCST fkLGOH	430885	rs6690625	-0,08388	0,117801	0,476413
Hb	Mitral	ebi-a-GCST fkLGOH	430885	rs6702992	-0,09349	0,117412	0,425886
Hb	Mitral	ebi-a-GCST fkLGOH	430885	rs6733795	-0,08998	0,117763	0,444823
Hb	Mitral	ebi-a-GCST fkLGOH	430885	rs6775517	-0,09323	0,11781	0,428718
Hb	Mitral	ebi-a-GCST fkLGOH	430885	rs6864465	-0,0876	0,117886	0,457449
Hb	Mitral	ebi-a-GCST fkLGOH	430885	rs6904682	-0,08297	0,117839	0,481383
Hb	Mitral	ebi-a-GCST fkLGOH	430885	rs6926552	-0,08737	0,117884	0,458586
Hb	Mitral	ebi-a-GCST fkLGOH	430885	rs6972333	-0,08972	0,117799	0,446296
Hb	Mitral	ebi-a-GCST fkLGOH	430885	rs7004509	-0,0891	0,117936	0,449937
Hb	Mitral	ebi-a-GCST fkLGOH	430885	rs7032549	-0,09048	0,117873	0,442748
Hb	Mitral	ebi-a-GCST fkLGOH	430885	rs7145036	-0,08583	0,11791	0,466647
Hb	Mitral	ebi-a-GCST fkLGOH	430885	rs715880	-0,08237	0,11764	0,483826
Hb	Mitral	ebi-a-GCST fkLGOH	430885	rs7182622	-0,09022	0,117773	0,44365
Hb	Mitral	ebi-a-GCST fkLGOH	430885	rs7211013	-0,08677	0,11787	0,46163
Hb	Mitral	ebi-a-GCST fkLGOH	430885	rs7244849	-0,09135	0,117658	0,437507
Hb	Mitral	ebi-a-GCST fkLGOH	430885	rs7247407	-0,08788	0,117887	0,456015
Hb	Mitral	ebi-a-GCST fkLGOH	430885	rs7263897	-0,08042	0,117533	0,493815
Hb	Mitral	ebi-a-GCST fkLGOH	430885	rs7271743	-0,0933	0,117705	0,427969
Hb	Mitral	ebi-a-GCST fkLGOH	430885	rs7273059	-0,09234	0,117814	0,433173
Hb	Mitral	ebi-a-GCST fkLGOH	430885	rs7275288	-0,10883	0,116947	0,352056
Hb	Mitral	ebi-a-GCST fkLGOH	430885	rs7276367	-0,09106	0,117718	0,439195
Hb	Mitral	ebi-a-GCST fkLGOH	430885	rs7282824	-0,08517	0,11784	0,469812
Hb	Mitral	ebi-a-GCST fkLGOH	430885	rs7283527	-0,0879	0,117956	0,456148
Hb	Mitral	ebi-a-GCST fkLGOH	430885	rs7291756	-0,0861	0,117916	0,465295
Hb	Mitral	ebi-a-GCST fkLGOH	430885	rs7319798	-0,08583	0,117835	0,466387
Hb	Mitral	ebi-a-GCST fkLGOH	430885	rs7324534	-0,08691	0,117865	0,46092
Hb	Mitral	ebi-a-GCST fkLGOH	430885	rs742536	-0,08274	0,117701	0,482075
Hb	Mitral	ebi-a-GCST fkLGOH	430885	rs743590	-0,08732	0,117875	0,458845
Hb	Mitral	ebi-a-GCST fkLGOH	430885	rs744103	-0,10354	0,117822	0,379522
Hb	Mitral	ebi-a-GCST fkLGOH	430885	rs7441457	-0,08865	0,117886	0,452058
Hb	Mitral	ebi-a-GCST fkLGOH	430885	rs7525164	-0,09501	0,117802	0,419922
Hb	Mitral	ebi-a-GCST fkLGOH	430885	rs752590	-0,09468	0,117887	0,421916
Hb	Mitral	ebi-a-GCST fkLGOH	430885	rs7550758	-0,1003	0,116455	0,389084
Hb	Mitral	ebi-a-GCST fkLGOH	430885	rs7579264	-0,07606	0,117871	0,518757
Hb	Mitral	ebi-a-GCST fkLGOH	430885	rs7589807	-0,08533	0,117827	0,468929
Hb	Mitral	ebi-a-GCST fkLGOH	430885	rs760077	-0,09088	0,118165	0,441831
Hb	Mitral	ebi-a-GCST fkLGOH	430885	rs7601531	-0,08773	0,117889	0,456765
Hb	Mitral	ebi-a-GCST fkLGOH	430885	rs7611163	-0,08377	0,117719	0,476701

Hb	Mitral	ebi-a-GCST	fkLGOH	430885	rs7676376	-0,08903	0,117837	0,449949
Hb	Mitral	ebi-a-GCST	fkLGOH	430885	rs7693188	-0,07425	0,117753	0,528308
Hb	Mitral	ebi-a-GCST	fkLGOH	430885	rs7697279	-0,09058	0,117737	0,441679
Hb	Mitral	ebi-a-GCST	fkLGOH	430885	rs77389	-0,08453	0,117787	0,472991
Hb	Mitral	ebi-a-GCST	fkLGOH	430885	rs7748640	-0,09278	0,117643	0,430313
Hb	Mitral	ebi-a-GCST	fkLGOH	430885	rs7754216	-0,09157	0,118029	0,437835
Hb	Mitral	ebi-a-GCST	fkLGOH	430885	rs7766720	-0,08871	0,117863	0,451636
Hb	Mitral	ebi-a-GCST	fkLGOH	430885	rs7862262	-0,08637	0,117858	0,463651
Hb	Mitral	ebi-a-GCST	fkLGOH	430885	rs7883956	-0,08437	0,117779	0,473763
Hb	Mitral	ebi-a-GCST	fkLGOH	430885	rs7885958	-0,08829	0,117854	0,453753
Hb	Mitral	ebi-a-GCST	fkLGOH	430885	rs791270	-0,08719	0,117887	0,459547
Hb	Mitral	ebi-a-GCST	fkLGOH	430885	rs7916396	-0,0827	0,117645	0,482079
Hb	Mitral	ebi-a-GCST	fkLGOH	430885	rs7960795	-0,08007	0,117228	0,494613
Hb	Mitral	ebi-a-GCST	fkLGOH	430885	rs797192	-0,0828	0,117703	0,481759
Hb	Mitral	ebi-a-GCST	fkLGOH	430885	rs797343	-0,08189	0,11779	0,486934
Hb	Mitral	ebi-a-GCST	fkLGOH	430885	rs8000803	-0,08607	0,117867	0,465245
Hb	Mitral	ebi-a-GCST	fkLGOH	430885	rs800881	-0,09057	0,117899	0,442357
Hb	Mitral	ebi-a-GCST	fkLGOH	430885	rs8020977	-0,08399	0,117827	0,475965
Hb	Mitral	ebi-a-GCST	fkLGOH	430885	rs8026198	-0,09037	0,117765	0,442861
Hb	Mitral	ebi-a-GCST	fkLGOH	430885	rs8032019	-0,09284	0,117584	0,4298
Hb	Mitral	ebi-a-GCST	fkLGOH	430885	rs833805	-0,07991	0,118302	0,499364
Hb	Mitral	ebi-a-GCST	fkLGOH	430885	rs847821	-0,08441	0,117803	0,473672
Hb	Mitral	ebi-a-GCST	fkLGOH	430885	rs855791	-0,06819	0,121393	0,574305
Hb	Mitral	ebi-a-GCST	fkLGOH	430885	rs863678	-0,08707	0,118002	0,460591
Hb	Mitral	ebi-a-GCST	fkLGOH	430885	rs866929	-0,08171	0,117699	0,487526
Hb	Mitral	ebi-a-GCST	fkLGOH	430885	rs885506	-0,0898	0,117785	0,445828
Hb	Mitral	ebi-a-GCST	fkLGOH	430885	rs8887	-0,08134	0,118016	0,490706
Hb	Mitral	ebi-a-GCST	fkLGOH	430885	rs909236	-0,08435	0,117775	0,473863
Hb	Mitral	ebi-a-GCST	fkLGOH	430885	rs9258357	-0,08632	0,118073	0,464734
Hb	Mitral	ebi-a-GCST	fkLGOH	430885	rs9302635	-0,09238	0,117786	0,432871
Hb	Mitral	ebi-a-GCST	fkLGOH	430885	rs9303620	-0,09016	0,117887	0,444385
Hb	Mitral	ebi-a-GCST	fkLGOH	430885	rs9327454	-0,08718	0,117902	0,45965
Hb	Mitral	ebi-a-GCST	fkLGOH	430885	rs9370102	-0,08603	0,117854	0,465386
Hb	Mitral	ebi-a-GCST	fkLGOH	430885	rs9400888	-0,09821	0,116446	0,399007
Hb	Mitral	ebi-a-GCST	fkLGOH	430885	rs9403391	-0,08729	0,117881	0,459002
Hb	Mitral	ebi-a-GCST	fkLGOH	430885	rs9456859	-0,08164	0,11754	0,487315
Hb	Mitral	ebi-a-GCST	fkLGOH	430885	rs9645770	-0,08287	0,117672	0,481291
Hb	Mitral	ebi-a-GCST	fkLGOH	430885	rs9690544	-0,08994	0,11786	0,445389
Hb	Mitral	ebi-a-GCST	fkLGOH	430885	rs9771385	-0,09251	0,117884	0,432616
Hb	Mitral	ebi-a-GCST	fkLGOH	430885	rs9816588	-0,08719	0,11798	0,459915
Hb	Mitral	ebi-a-GCST	fkLGOH	430885	rs9861140	-0,08324	0,117638	0,479171
Hb	Mitral	ebi-a-GCST	fkLGOH	430885	rs9895661	-0,08811	0,118095	0,455584
Hb	Mitral	ebi-a-GCST	fkLGOH	430885	rs9953010	-0,08603	0,117851	0,465419
Hb	Mitral	ebi-a-GCST	fkLGOH	430885	rs999010	-0,08443	0,118052	0,474507
Hb	Mitral	ebi-a-GCST	fkLGOH	430885	All	-0,08727	0,117657	0,458263
MCV	Mitral	ebi-a-GCST	bFhbvJ	436723	rs1004867	-0,05595	0,079106	0,479383
MCV	Mitral	ebi-a-GCST	bFhbvJ	436723	rs1007893	-0,06446	0,079401	0,416892
MCV	Mitral	ebi-a-GCST	bFhbvJ	436723	rs1008313	-0,06233	0,079482	0,432894
MCV	Mitral	ebi-a-GCST	bFhbvJ	436723	rs1008383	-0,06572	0,079207	0,406713
MCV	Mitral	ebi-a-GCST	bFhbvJ	436723	rs1009372	-0,06288	0,079485	0,428875

MCV	Mitral	ebi-a-GCST	bFhbvJ	436723	rs1009791	-0,06564	0,079331	0,40799
MCV	Mitral	ebi-a-GCST	bFhbvJ	436723	rs1015242	-0,06671	0,079028	0,398567
MCV	Mitral	ebi-a-GCST	bFhbvJ	436723	rs1015647	-0,06373	0,079477	0,42263
MCV	Mitral	ebi-a-GCST	bFhbvJ	436723	rs1017213	-0,06323	0,079476	0,426285
MCV	Mitral	ebi-a-GCST	bFhbvJ	436723	rs1021181	-0,06612	0,079342	0,404663
MCV	Mitral	ebi-a-GCST	bFhbvJ	436723	rs1022688	-0,0623	0,079461	0,433042
MCV	Mitral	ebi-a-GCST	bFhbvJ	436723	rs1024901	-0,06225	0,079472	0,433423
MCV	Mitral	ebi-a-GCST	bFhbvJ	436723	rs1027010	-0,06612	0,079215	0,403879
MCV	Mitral	ebi-a-GCST	bFhbvJ	436723	rs10274	-0,05817	0,079473	0,464209
MCV	Mitral	ebi-a-GCST	bFhbvJ	436723	rs1047891	-0,06437	0,079509	0,418174
MCV	Mitral	ebi-a-GCST	bFhbvJ	436723	rs1049457	-0,05989	0,079409	0,450696
MCV	Mitral	ebi-a-GCST	bFhbvJ	436723	rs1075865	-0,06068	0,080125	0,448864
MCV	Mitral	ebi-a-GCST	bFhbvJ	436723	rs1079552	-0,06615	0,07913	0,403187
MCV	Mitral	ebi-a-GCST	bFhbvJ	436723	rs1080499	-0,0633	0,079475	0,425758
MCV	Mitral	ebi-a-GCST	bFhbvJ	436723	rs1081140	-0,065	0,079412	0,413063
MCV	Mitral	ebi-a-GCST	bFhbvJ	436723	rs1084674	-0,06253	0,079494	0,431553
MCV	Mitral	ebi-a-GCST	bFhbvJ	436723	rs1089381	-0,06212	0,079503	0,434614
MCV	Mitral	ebi-a-GCST	bFhbvJ	436723	rs1090015	-0,06466	0,079373	0,415315
MCV	Mitral	ebi-a-GCST	bFhbvJ	436723	rs1090125	-0,05807	0,079585	0,46562
MCV	Mitral	ebi-a-GCST	bFhbvJ	436723	rs1090284	-0,06263	0,079478	0,430697
MCV	Mitral	ebi-a-GCST	bFhbvJ	436723	rs1092339	-0,06155	0,079547	0,439075
MCV	Mitral	ebi-a-GCST	bFhbvJ	436723	rs1105517	-0,06651	0,079154	0,400762
MCV	Mitral	ebi-a-GCST	bFhbvJ	436723	rs1106238	-0,06331	0,079494	0,425783
MCV	Mitral	ebi-a-GCST	bFhbvJ	436723	rs1108822	-0,05956	0,079024	0,45106
MCV	Mitral	ebi-a-GCST	bFhbvJ	436723	rs1109729	-0,06011	0,079147	0,447577
MCV	Mitral	ebi-a-GCST	bFhbvJ	436723	rs1110488	-0,06082	0,079523	0,444411
MCV	Mitral	ebi-a-GCST	bFhbvJ	436723	rs1113150	-0,06319	0,079483	0,426613
MCV	Mitral	ebi-a-GCST	bFhbvJ	436723	rs1113959	-0,06232	0,079501	0,433136
MCV	Mitral	ebi-a-GCST	bFhbvJ	436723	rs1117035	-0,06279	0,079477	0,429488
MCV	Mitral	ebi-a-GCST	bFhbvJ	436723	rs1117737	-0,0598	0,079192	0,450175
MCV	Mitral	ebi-a-GCST	bFhbvJ	436723	rs1121148	-0,06557	0,079599	0,410072
MCV	Mitral	ebi-a-GCST	bFhbvJ	436723	rs1121805	-0,06364	0,079525	0,423549
MCV	Mitral	ebi-a-GCST	bFhbvJ	436723	rs1121896	-0,06228	0,079466	0,433212
MCV	Mitral	ebi-a-GCST	bFhbvJ	436723	rs1123470	-0,06275	0,079481	0,429788
MCV	Mitral	ebi-a-GCST	bFhbvJ	436723	rs1124466	-0,06059	0,079362	0,44518
MCV	Mitral	ebi-a-GCST	bFhbvJ	436723	rs1125771	-0,06461	0,079414	0,415856
MCV	Mitral	ebi-a-GCST	bFhbvJ	436723	rs1129019	-0,06098	0,079306	0,441913
MCV	Mitral	ebi-a-GCST	bFhbvJ	436723	rs1130460	-0,06422	0,079466	0,419015
MCV	Mitral	ebi-a-GCST	bFhbvJ	436723	rs1130975	-0,06293	0,079477	0,428462
MCV	Mitral	ebi-a-GCST	bFhbvJ	436723	rs1134043	-0,06393	0,079457	0,421027
MCV	Mitral	ebi-a-GCST	bFhbvJ	436723	rs1134634	-0,05889	0,07947	0,45865
MCV	Mitral	ebi-a-GCST	bFhbvJ	436723	rs1139311	-0,05949	0,07922	0,452675
MCV	Mitral	ebi-a-GCST	bFhbvJ	436723	rs1141658	-0,06625	0,079498	0,404633
MCV	Mitral	ebi-a-GCST	bFhbvJ	436723	rs1150569	-0,06406	0,079492	0,420309
MCV	Mitral	ebi-a-GCST	bFhbvJ	436723	rs1155715	-0,06536	0,079411	0,410494
MCV	Mitral	ebi-a-GCST	bFhbvJ	436723	rs1157812	-0,06281	0,079483	0,429373
MCV	Mitral	ebi-a-GCST	bFhbvJ	436723	rs1160013	-0,06268	0,079509	0,430487
MCV	Mitral	ebi-a-GCST	bFhbvJ	436723	rs1162713	-0,06681	0,079224	0,399074
MCV	Mitral	ebi-a-GCST	bFhbvJ	436723	rs1162748	-0,06668	0,079751	0,403066
MCV	Mitral	ebi-a-GCST	bFhbvJ	436723	rs1162830	-0,06516	0,079428	0,411984

MCV	Mitral	ebi-a-GCST	bFhbvJ	436723	rs1163820	-0,0642	0,079456	0,419078
MCV	Mitral	ebi-a-GCST	bFhbvJ	436723	rs1164302	-0,06307	0,07949	0,42751
MCV	Mitral	ebi-a-GCST	bFhbvJ	436723	rs1169087	-0,06469	0,079426	0,415382
MCV	Mitral	ebi-a-GCST	bFhbvJ	436723	rs1169581	-0,06325	0,079506	0,426283
MCV	Mitral	ebi-a-GCST	bFhbvJ	436723	rs1171008	-0,06504	0,079343	0,412375
MCV	Mitral	ebi-a-GCST	bFhbvJ	436723	rs1171076	-0,06622	0,079312	0,403788
MCV	Mitral	ebi-a-GCST	bFhbvJ	436723	rs1171119	-0,06727	0,079288	0,396169
MCV	Mitral	ebi-a-GCST	bFhbvJ	436723	rs1171420	-0,05801	0,079475	0,465417
MCV	Mitral	ebi-a-GCST	bFhbvJ	436723	rs1172337	-0,06195	0,079524	0,435985
MCV	Mitral	ebi-a-GCST	bFhbvJ	436723	rs1177380	-0,05864	0,079073	0,458337
MCV	Mitral	ebi-a-GCST	bFhbvJ	436723	rs1178731	-0,06125	0,079427	0,440647
MCV	Mitral	ebi-a-GCST	bFhbvJ	436723	rs1183613	-0,0598	0,079068	0,449457
MCV	Mitral	ebi-a-GCST	bFhbvJ	436723	rs1184962	-0,06009	0,079251	0,448353
MCV	Mitral	ebi-a-GCST	bFhbvJ	436723	rs1190541	-0,06217	0,079463	0,434003
MCV	Mitral	ebi-a-GCST	bFhbvJ	436723	rs1203331	-0,06314	0,079522	0,427201
MCV	Mitral	ebi-a-GCST	bFhbvJ	436723	rs1214664	-0,06447	0,079438	0,417062
MCV	Mitral	ebi-a-GCST	bFhbvJ	436723	rs1219394	-0,06462	0,079557	0,416681
MCV	Mitral	ebi-a-GCST	bFhbvJ	436723	rs1224712	-0,06338	0,079474	0,425157
MCV	Mitral	ebi-a-GCST	bFhbvJ	436723	rs1235615	-0,06263	0,079475	0,430632
MCV	Mitral	ebi-a-GCST	bFhbvJ	436723	rs1250561	-0,06514	0,079544	0,412865
MCV	Mitral	ebi-a-GCST	bFhbvJ	436723	rs1252847	-0,06252	0,079468	0,431422
MCV	Mitral	ebi-a-GCST	bFhbvJ	436723	rs1258217	-0,0653	0,079526	0,411615
MCV	Mitral	ebi-a-GCST	bFhbvJ	436723	rs1259697	-0,06251	0,079474	0,431568
MCV	Mitral	ebi-a-GCST	bFhbvJ	436723	rs1260518	-0,06337	0,079474	0,425206
MCV	Mitral	ebi-a-GCST	bFhbvJ	436723	rs1261764	-0,06445	0,079397	0,416916
MCV	Mitral	ebi-a-GCST	bFhbvJ	436723	rs1270183	-0,06456	0,079413	0,416216
MCV	Mitral	ebi-a-GCST	bFhbvJ	436723	rs1288186	-0,06409	0,079424	0,419691
MCV	Mitral	ebi-a-GCST	bFhbvJ	436723	rs1291434	-0,06339	0,079473	0,425102
MCV	Mitral	ebi-a-GCST	bFhbvJ	436723	rs1294106	-0,06498	0,079376	0,413009
MCV	Mitral	ebi-a-GCST	bFhbvJ	436723	rs1302950	-0,06215	0,079463	0,434146
MCV	Mitral	ebi-a-GCST	bFhbvJ	436723	rs1318907	-0,0633	0,079487	0,425807
MCV	Mitral	ebi-a-GCST	bFhbvJ	436723	rs1321336	-0,05382	0,079018	0,495808
MCV	Mitral	ebi-a-GCST	bFhbvJ	436723	rs1327723	-0,0662	0,079496	0,40499
MCV	Mitral	ebi-a-GCST	bFhbvJ	436723	rs1330144	-0,06292	0,079481	0,428542
MCV	Mitral	ebi-a-GCST	bFhbvJ	436723	rs1332101	-0,06518	0,07952	0,412405
MCV	Mitral	ebi-a-GCST	bFhbvJ	436723	rs136211	-0,06207	0,079523	0,435062
MCV	Mitral	ebi-a-GCST	bFhbvJ	436723	rs137921	-0,06154	0,079421	0,438438
MCV	Mitral	ebi-a-GCST	bFhbvJ	436723	rs1384539	-0,06306	0,079484	0,427535
MCV	Mitral	ebi-a-GCST	bFhbvJ	436723	rs1399746	-0,05992	0,079353	0,450174
MCV	Mitral	ebi-a-GCST	bFhbvJ	436723	rs1401130	-0,0655	0,079257	0,408595
MCV	Mitral	ebi-a-GCST	bFhbvJ	436723	rs1404256	-0,0656	0,079295	0,408037
MCV	Mitral	ebi-a-GCST	bFhbvJ	436723	rs140522	-0,06145	0,080276	0,444002
MCV	Mitral	ebi-a-GCST	bFhbvJ	436723	rs1405540	-0,06327	0,079533	0,42628
MCV	Mitral	ebi-a-GCST	bFhbvJ	436723	rs1406696	-0,06592	0,07928	0,405693
MCV	Mitral	ebi-a-GCST	bFhbvJ	436723	rs1408272	-0,06793	0,08154	0,404785
MCV	Mitral	ebi-a-GCST	bFhbvJ	436723	rs1414946	-0,0658	0,07961	0,408479
MCV	Mitral	ebi-a-GCST	bFhbvJ	436723	rs1415192	-0,06273	0,079475	0,429947
MCV	Mitral	ebi-a-GCST	bFhbvJ	436723	rs1437475	-0,06333	0,079484	0,425616
MCV	Mitral	ebi-a-GCST	bFhbvJ	436723	rs1442045	-0,06284	0,079548	0,429552
MCV	Mitral	ebi-a-GCST	bFhbvJ	436723	rs1444541	-0,06434	0,079401	0,417744

MCV	Mitral	ebi-a-GCST	bFhbvJ	436723	rs1449427:	-0,06266	0,07948	0,430467
MCV	Mitral	ebi-a-GCST	bFhbvJ	436723	rs1452945:	-0,06416	0,079473	0,419457
MCV	Mitral	ebi-a-GCST	bFhbvJ	436723	rs1457552(	-0,06196	0,07943	0,435372
MCV	Mitral	ebi-a-GCST	bFhbvJ	436723	rs1460089:	-0,06229	0,079486	0,433212
MCV	Mitral	ebi-a-GCST	bFhbvJ	436723	rs1465424:	-0,06043	0,079379	0,446492
MCV	Mitral	ebi-a-GCST	bFhbvJ	436723	rs1487358!	-0,06526	0,079371	0,410991
MCV	Mitral	ebi-a-GCST	bFhbvJ	436723	rs1492903-	-0,06302	0,079534	0,428146
MCV	Mitral	ebi-a-GCST	bFhbvJ	436723	rs1504253!	-0,06177	0,079467	0,437017
MCV	Mitral	ebi-a-GCST	bFhbvJ	436723	rs1520195	-0,06409	0,079449	0,419834
MCV	Mitral	ebi-a-GCST	bFhbvJ	436723	rs1569419	-0,0644	0,079507	0,417938
MCV	Mitral	ebi-a-GCST	bFhbvJ	436723	rs1584063	-0,06243	0,079486	0,432208
MCV	Mitral	ebi-a-GCST	bFhbvJ	436723	rs159058	-0,06354	0,079542	0,424416
MCV	Mitral	ebi-a-GCST	bFhbvJ	436723	rs166864	-0,06553	0,079404	0,409208
MCV	Mitral	ebi-a-GCST	bFhbvJ	436723	rs1678527	-0,06291	0,079479	0,428603
MCV	Mitral	ebi-a-GCST	bFhbvJ	436723	rs1684334(	-0,05872	0,079649	0,460968
MCV	Mitral	ebi-a-GCST	bFhbvJ	436723	rs1694830!	-0,0618	0,079457	0,436687
MCV	Mitral	ebi-a-GCST	bFhbvJ	436723	rs1701516!	-0,06079	0,07945	0,44422
MCV	Mitral	ebi-a-GCST	bFhbvJ	436723	rs1706865!	-0,06	0,07945	0,450169
MCV	Mitral	ebi-a-GCST	bFhbvJ	436723	rs1711638-	-0,06074	0,079457	0,444586
MCV	Mitral	ebi-a-GCST	bFhbvJ	436723	rs1711671(	-0,06625	0,079292	0,403424
MCV	Mitral	ebi-a-GCST	bFhbvJ	436723	rs1744231:	-0,06429	0,07943	0,418254
MCV	Mitral	ebi-a-GCST	bFhbvJ	436723	rs174548	-0,0634	0,079503	0,425174
MCV	Mitral	ebi-a-GCST	bFhbvJ	436723	rs1746264(	-0,06633	0,07925	0,402637
MCV	Mitral	ebi-a-GCST	bFhbvJ	436723	rs1746452!	-0,06257	0,079477	0,431086
MCV	Mitral	ebi-a-GCST	bFhbvJ	436723	rs1747636-	-0,0538	0,079694	0,499639
MCV	Mitral	ebi-a-GCST	bFhbvJ	436723	rs1769965!	-0,06429	0,079501	0,418682
MCV	Mitral	ebi-a-GCST	bFhbvJ	436723	rs1775869!	-0,06085	0,079571	0,444423
MCV	Mitral	ebi-a-GCST	bFhbvJ	436723	rs1780237!	-0,06493	0,079323	0,413033
MCV	Mitral	ebi-a-GCST	bFhbvJ	436723	rs1782763	-0,05862	0,079293	0,459735
MCV	Mitral	ebi-a-GCST	bFhbvJ	436723	rs1814313:	-0,06318	0,079475	0,426664
MCV	Mitral	ebi-a-GCST	bFhbvJ	436723	rs1854039	-0,06433	0,079409	0,417904
MCV	Mitral	ebi-a-GCST	bFhbvJ	436723	rs1866051	-0,06303	0,079504	0,427898
MCV	Mitral	ebi-a-GCST	bFhbvJ	436723	rs1880791:	-0,06246	0,079481	0,431921
MCV	Mitral	ebi-a-GCST	bFhbvJ	436723	rs1891621:	-0,06544	0,079326	0,409392
MCV	Mitral	ebi-a-GCST	bFhbvJ	436723	rs1913466	-0,0632	0,079477	0,426524
MCV	Mitral	ebi-a-GCST	bFhbvJ	436723	rs1958078	-0,0623	0,079501	0,433239
MCV	Mitral	ebi-a-GCST	bFhbvJ	436723	rs198358	-0,06458	0,079467	0,41638
MCV	Mitral	ebi-a-GCST	bFhbvJ	436723	rs1992383	-0,06124	0,079541	0,44135
MCV	Mitral	ebi-a-GCST	bFhbvJ	436723	rs2005277	-0,06202	0,079468	0,435127
MCV	Mitral	ebi-a-GCST	bFhbvJ	436723	rs2015803	-0,06095	0,079317	0,44222
MCV	Mitral	ebi-a-GCST	bFhbvJ	436723	rs2020204!	-0,0642	0,07946	0,419088
MCV	Mitral	ebi-a-GCST	bFhbvJ	436723	rs2026428	-0,0627	0,0797	0,431453
MCV	Mitral	ebi-a-GCST	bFhbvJ	436723	rs2067663	-0,06248	0,079531	0,432096
MCV	Mitral	ebi-a-GCST	bFhbvJ	436723	rs2074534	-0,06159	0,079431	0,438095
MCV	Mitral	ebi-a-GCST	bFhbvJ	436723	rs2075672	-0,07484	0,079972	0,349379
MCV	Mitral	ebi-a-GCST	bFhbvJ	436723	rs2125060	-0,06284	0,079479	0,429179
MCV	Mitral	ebi-a-GCST	bFhbvJ	436723	rs2140875	-0,064	0,079476	0,420665
MCV	Mitral	ebi-a-GCST	bFhbvJ	436723	rs218264	-0,07229	0,080423	0,368699
MCV	Mitral	ebi-a-GCST	bFhbvJ	436723	rs2236044	-0,06599	0,079079	0,404032
MCV	Mitral	ebi-a-GCST	bFhbvJ	436723	rs2236314	-0,06169	0,079496	0,437774

MCV	Mitral	ebi-a-GCST	bFhbvJ	436723	rs2238368	-0,04297	0,079789	0,590222
MCV	Mitral	ebi-a-GCST	bFhbvJ	436723	rs2240460	-0,06713	0,079047	0,395763
MCV	Mitral	ebi-a-GCST	bFhbvJ	436723	rs2243834	-0,06474	0,079481	0,415325
MCV	Mitral	ebi-a-GCST	bFhbvJ	436723	rs2273799	-0,05968	0,079497	0,452852
MCV	Mitral	ebi-a-GCST	bFhbvJ	436723	rs2277339	-0,06609	0,079443	0,405444
MCV	Mitral	ebi-a-GCST	bFhbvJ	436723	rs2299902	-0,06419	0,079427	0,419007
MCV	Mitral	ebi-a-GCST	bFhbvJ	436723	rs230542	-0,06342	0,079495	0,424994
MCV	Mitral	ebi-a-GCST	bFhbvJ	436723	rs2351959	-0,06406	0,07949	0,420329
MCV	Mitral	ebi-a-GCST	bFhbvJ	436723	rs2364368	-0,06625	0,079145	0,40252
MCV	Mitral	ebi-a-GCST	bFhbvJ	436723	rs2366541	-0,0652	0,079363	0,411322
MCV	Mitral	ebi-a-GCST	bFhbvJ	436723	rs2386886	-0,06112	0,07947	0,441834
MCV	Mitral	ebi-a-GCST	bFhbvJ	436723	rs2393750	-0,06429	0,079449	0,418406
MCV	Mitral	ebi-a-GCST	bFhbvJ	436723	rs2395043	-0,06281	0,079488	0,429453
MCV	Mitral	ebi-a-GCST	bFhbvJ	436723	rs243067	-0,0557	0,079715	0,484682
MCV	Mitral	ebi-a-GCST	bFhbvJ	436723	rs2438206	-0,06272	0,079493	0,43012
MCV	Mitral	ebi-a-GCST	bFhbvJ	436723	rs2439406	-0,06636	0,079119	0,401609
MCV	Mitral	ebi-a-GCST	bFhbvJ	436723	rs2461848	-0,0619	0,07944	0,43583
MCV	Mitral	ebi-a-GCST	bFhbvJ	436723	rs2492301	-0,06686	0,079372	0,399556
MCV	Mitral	ebi-a-GCST	bFhbvJ	436723	rs2518142	-0,0636	0,079473	0,423522
MCV	Mitral	ebi-a-GCST	bFhbvJ	436723	rs2537592	-0,06355	0,079496	0,424061
MCV	Mitral	ebi-a-GCST	bFhbvJ	436723	rs2542573	-0,06319	0,079489	0,426655
MCV	Mitral	ebi-a-GCST	bFhbvJ	436723	rs2553791	-0,06429	0,079402	0,418108
MCV	Mitral	ebi-a-GCST	bFhbvJ	436723	rs2556096	-0,06419	0,079474	0,419242
MCV	Mitral	ebi-a-GCST	bFhbvJ	436723	rs26233	-0,0646	0,079479	0,416367
MCV	Mitral	ebi-a-GCST	bFhbvJ	436723	rs2701128	-0,0634	0,079488	0,42507
MCV	Mitral	ebi-a-GCST	bFhbvJ	436723	rs27128	-0,0618	0,079458	0,436736
MCV	Mitral	ebi-a-GCST	bFhbvJ	436723	rs2713936	-0,06091	0,079505	0,443624
MCV	Mitral	ebi-a-GCST	bFhbvJ	436723	rs271620	-0,061	0,079496	0,442853
MCV	Mitral	ebi-a-GCST	bFhbvJ	436723	rs2738786	-0,06794	0,078638	0,387604
MCV	Mitral	ebi-a-GCST	bFhbvJ	436723	rs2796457	-0,0599	0,079285	0,449938
MCV	Mitral	ebi-a-GCST	bFhbvJ	436723	rs2834256	-0,05725	0,07952	0,47158
MCV	Mitral	ebi-a-GCST	bFhbvJ	436723	rs2834712	-0,06664	0,079309	0,400759
MCV	Mitral	ebi-a-GCST	bFhbvJ	436723	rs2836422	-0,06153	0,079479	0,438839
MCV	Mitral	ebi-a-GCST	bFhbvJ	436723	rs2849885!	-0,06722	0,079829	0,399725
MCV	Mitral	ebi-a-GCST	bFhbvJ	436723	rs2862103!	-0,06404	0,079446	0,420209
MCV	Mitral	ebi-a-GCST	bFhbvJ	436723	rs2875621!	-0,06568	0,079191	0,406918
MCV	Mitral	ebi-a-GCST	bFhbvJ	436723	rs2906773	-0,0623	0,079463	0,43303
MCV	Mitral	ebi-a-GCST	bFhbvJ	436723	rs2979469	-0,0605	0,079519	0,446741
MCV	Mitral	ebi-a-GCST	bFhbvJ	436723	rs3009576	-0,06162	0,079421	0,437828
MCV	Mitral	ebi-a-GCST	bFhbvJ	436723	rs3025651	-0,06166	0,079599	0,438562
MCV	Mitral	ebi-a-GCST	bFhbvJ	436723	rs3109917	-0,06391	0,079453	0,421161
MCV	Mitral	ebi-a-GCST	bFhbvJ	436723	rs3211960	-0,06448	0,079471	0,417141
MCV	Mitral	ebi-a-GCST	bFhbvJ	436723	rs322873	-0,05819	0,079398	0,463605
MCV	Mitral	ebi-a-GCST	bFhbvJ	436723	rs338533	-0,06167	0,079445	0,437605
MCV	Mitral	ebi-a-GCST	bFhbvJ	436723	rs3421223!	-0,06323	0,079478	0,426286
MCV	Mitral	ebi-a-GCST	bFhbvJ	436723	rs3451743!	-0,06225	0,079481	0,433522
MCV	Mitral	ebi-a-GCST	bFhbvJ	436723	rs3452308!	-0,06524	0,079406	0,411271
MCV	Mitral	ebi-a-GCST	bFhbvJ	436723	rs347519	-0,06337	0,079484	0,425287
MCV	Mitral	ebi-a-GCST	bFhbvJ	436723	rs3493119!	-0,05924	0,079484	0,456072
MCV	Mitral	ebi-a-GCST	bFhbvJ	436723	rs3515020!	-0,06031	0,079337	0,44714

MCV	Mitral	ebi-a-GCST	bFhbvJ	436723	rs3533684(	-0,06249	0,079476	0,431677
MCV	Mitral	ebi-a-GCST	bFhbvJ	436723	rs3535198!	-0,06409	0,079428	0,419696
MCV	Mitral	ebi-a-GCST	bFhbvJ	436723	rs3536096‹	-0,06604	0,079747	0,407581
MCV	Mitral	ebi-a-GCST	bFhbvJ	436723	rs3538800:	-0,06436	0,079493	0,418121
MCV	Mitral	ebi-a-GCST	bFhbvJ	436723	rs35892197	-0,0651	0,079338	0,411897
MCV	Mitral	ebi-a-GCST	bFhbvJ	436723	rs3597982ł	-0,05836	0,079447	0,462571
MCV	Mitral	ebi-a-GCST	bFhbvJ	436723	rs3599852‹	-0,05928	0,079587	0,456333
MCV	Mitral	ebi-a-GCST	bFhbvJ	436723	rs3622515:	-0,06479	0,079475	0,414929
MCV	Mitral	ebi-a-GCST	bFhbvJ	436723	rs3740685	-0,06025	0,079445	0,448245
MCV	Mitral	ebi-a-GCST	bFhbvJ	436723	rs3752574	-0,0623	0,079478	0,433149
MCV	Mitral	ebi-a-GCST	bFhbvJ	436723	rs3768321	-0,05858	0,079271	0,459931
MCV	Mitral	ebi-a-GCST	bFhbvJ	436723	rs3796948	-0,06171	0,079427	0,437182
MCV	Mitral	ebi-a-GCST	bFhbvJ	436723	rs3809627	-0,06065	0,079617	0,446227
MCV	Mitral	ebi-a-GCST	bFhbvJ	436723	rs3811444	-0,06349	0,079787	0,426167
MCV	Mitral	ebi-a-GCST	bFhbvJ	436723	rs3811473	-0,06054	0,07943	0,445923
MCV	Mitral	ebi-a-GCST	bFhbvJ	436723	rs381500	-0,06923	0,079462	0,383609
MCV	Mitral	ebi-a-GCST	bFhbvJ	436723	rs3823735	-0,06545	0,079387	0,409684
MCV	Mitral	ebi-a-GCST	bFhbvJ	436723	rs3847694	-0,06299	0,079498	0,42818
MCV	Mitral	ebi-a-GCST	bFhbvJ	436723	rs3869550	-0,06321	0,07951	0,426613
MCV	Mitral	ebi-a-GCST	bFhbvJ	436723	rs395564	-0,06791	0,079374	0,392263
MCV	Mitral	ebi-a-GCST	bFhbvJ	436723	rs41298087	-0,05046	0,079706	0,526642
MCV	Mitral	ebi-a-GCST	bFhbvJ	436723	rs4149056	-0,06892	0,079047	0,3833
MCV	Mitral	ebi-a-GCST	bFhbvJ	436723	rs4302194	-0,06556	0,079228	0,40799
MCV	Mitral	ebi-a-GCST	bFhbvJ	436723	rs4332427	-0,06331	0,07951	0,425892
MCV	Mitral	ebi-a-GCST	bFhbvJ	436723	rs4378991	-0,06381	0,079495	0,422163
MCV	Mitral	ebi-a-GCST	bFhbvJ	436723	rs440837	-0,05993	0,079076	0,448552
MCV	Mitral	ebi-a-GCST	bFhbvJ	436723	rs4434423	-0,0605	0,079256	0,445268
MCV	Mitral	ebi-a-GCST	bFhbvJ	436723	rs4472338	-0,06855	0,079442	0,388211
MCV	Mitral	ebi-a-GCST	bFhbvJ	436723	rs4474982	-0,06487	0,07934	0,413566
MCV	Mitral	ebi-a-GCST	bFhbvJ	436723	rs4535497	-0,0636	0,079581	0,424178
MCV	Mitral	ebi-a-GCST	bFhbvJ	436723	rs4541821	-0,06282	0,079497	0,429428
MCV	Mitral	ebi-a-GCST	bFhbvJ	436723	rs4585697	-0,06466	0,079431	0,415597
MCV	Mitral	ebi-a-GCST	bFhbvJ	436723	rs4622761	-0,06306	0,079484	0,427548
MCV	Mitral	ebi-a-GCST	bFhbvJ	436723	rs4663199	-0,0626	0,079517	0,431173
MCV	Mitral	ebi-a-GCST	bFhbvJ	436723	rs4679875	-0,06498	0,079311	0,412641
MCV	Mitral	ebi-a-GCST	bFhbvJ	436723	rs4716059	-0,06827	0,079494	0,390459
MCV	Mitral	ebi-a-GCST	bFhbvJ	436723	rs4737010	-0,06354	0,079616	0,424797
MCV	Mitral	ebi-a-GCST	bFhbvJ	436723	rs474384	-0,06139	0,079505	0,440011
MCV	Mitral	ebi-a-GCST	bFhbvJ	436723	rs4783994	-0,06348	0,079472	0,424451
MCV	Mitral	ebi-a-GCST	bFhbvJ	436723	rs4801530	-0,06297	0,079479	0,428189
MCV	Mitral	ebi-a-GCST	bFhbvJ	436723	rs4832016	-0,06341	0,079479	0,424942
MCV	Mitral	ebi-a-GCST	bFhbvJ	436723	rs4890628	-0,05502	0,079817	0,490607
MCV	Mitral	ebi-a-GCST	bFhbvJ	436723	rs495124	-0,06622	0,079182	0,402987
MCV	Mitral	ebi-a-GCST	bFhbvJ	436723	rs496321	-0,06288	0,079542	0,429248
MCV	Mitral	ebi-a-GCST	bFhbvJ	436723	rs4982211	-0,05912	0,079307	0,45597
MCV	Mitral	ebi-a-GCST	bFhbvJ	436723	rs507964	-0,05955	0,079358	0,453037
MCV	Mitral	ebi-a-GCST	bFhbvJ	436723	rs5564155:	-0,06117	0,079443	0,441345
MCV	Mitral	ebi-a-GCST	bFhbvJ	436723	rs5564881(	-0,06554	0,079441	0,409361
MCV	Mitral	ebi-a-GCST	bFhbvJ	436723	rs5566490(	-0,06587	0,079191	0,405496
MCV	Mitral	ebi-a-GCST	bFhbvJ	436723	rs5582515:	-0,06375	0,079462	0,42236

MCV	Mitral	ebi-a-GCST	bFhbvJ	436723	rs5585367[illegible]	-0,06022	0,079294	0,447617
MCV	Mitral	ebi-a-GCST	bFhbvJ	436723	rs5608492[illegible]	-0,06616	0,079322	0,404274
MCV	Mitral	ebi-a-GCST	bFhbvJ	436723	rs5618923[illegible]	-0,06024	0,079489	0,448518
MCV	Mitral	ebi-a-GCST	bFhbvJ	436723	rs5622352[illegible]	-0,06411	0,079571	0,420399
MCV	Mitral	ebi-a-GCST	bFhbvJ	436723	rs5629571[illegible]	-0,06088	0,079396	0,443211
MCV	Mitral	ebi-a-GCST	bFhbvJ	436723	rs5635638[illegible]	-0,06322	0,079697	0,427632
MCV	Mitral	ebi-a-GCST	bFhbvJ	436723	rs5719970[illegible]	-0,06484	0,079411	0,414236
MCV	Mitral	ebi-a-GCST	bFhbvJ	436723	rs5743204[illegible]	-0,05709	0,079283	0,471457
MCV	Mitral	ebi-a-GCST	bFhbvJ	436723	rs5749445	-0,05906	0,079733	0,458885
MCV	Mitral	ebi-a-GCST	bFhbvJ	436723	rs5756503[illegible]	-0,06415	0,079471	0,41952
MCV	Mitral	ebi-a-GCST	bFhbvJ	436723	rs5811125[illegible]	-0,06664	0,079309	0,400783
MCV	Mitral	ebi-a-GCST	bFhbvJ	436723	rs5814140[illegible]	-0,04859	0,079067	0,538815
MCV	Mitral	ebi-a-GCST	bFhbvJ	436723	rs5865877[illegible]	-0,06476	0,079399	0,414709
MCV	Mitral	ebi-a-GCST	bFhbvJ	436723	rs5998599	-0,06228	0,079633	0,434165
MCV	Mitral	ebi-a-GCST	bFhbvJ	436723	rs6004041	-0,06461	0,079368	0,415631
MCV	Mitral	ebi-a-GCST	bFhbvJ	436723	rs600619	-0,06125	0,07938	0,440312
MCV	Mitral	ebi-a-GCST	bFhbvJ	436723	rs6006393	-0,06632	0,079446	0,403825
MCV	Mitral	ebi-a-GCST	bFhbvJ	436723	rs6014993	-0,07203	0,080095	0,368473
MCV	Mitral	ebi-a-GCST	bFhbvJ	436723	rs602261	-0,06197	0,079564	0,436074
MCV	Mitral	ebi-a-GCST	bFhbvJ	436723	rs6053030	-0,06228	0,079496	0,43339
MCV	Mitral	ebi-a-GCST	bFhbvJ	436723	rs6060987	-0,06542	0,079535	0,410784
MCV	Mitral	ebi-a-GCST	bFhbvJ	436723	rs6066313[illegible]	-0,06336	0,079483	0,425388
MCV	Mitral	ebi-a-GCST	bFhbvJ	436723	rs6069712[illegible]	-0,0674	0,079167	0,394582
MCV	Mitral	ebi-a-GCST	bFhbvJ	436723	rs6073289	-0,06282	0,07951	0,429465
MCV	Mitral	ebi-a-GCST	bFhbvJ	436723	rs6075741[illegible]	-0,06349	0,079523	0,42465
MCV	Mitral	ebi-a-GCST	bFhbvJ	436723	rs611010	-0,0624	0,079478	0,432352
MCV	Mitral	ebi-a-GCST	bFhbvJ	436723	rs6131371[illegible]	-0,06589	0,079442	0,40685
MCV	Mitral	ebi-a-GCST	bFhbvJ	436723	rs6165800[illegible]	-0,06386	0,079443	0,421493
MCV	Mitral	ebi-a-GCST	bFhbvJ	436723	rs6182397[illegible]	-0,06552	0,079589	0,410377
MCV	Mitral	ebi-a-GCST	bFhbvJ	436723	rs6182872[illegible]	-0,06262	0,079505	0,43093
MCV	Mitral	ebi-a-GCST	bFhbvJ	436723	rs6188087[illegible]	-0,06487	0,07935	0,41361
MCV	Mitral	ebi-a-GCST	bFhbvJ	436723	rs619247	-0,06516	0,079375	0,411729
MCV	Mitral	ebi-a-GCST	bFhbvJ	436723	rs6196645[illegible]	-0,06606	0,079298	0,404788
MCV	Mitral	ebi-a-GCST	bFhbvJ	436723	rs6203665[illegible]	-0,06452	0,079525	0,4172
MCV	Mitral	ebi-a-GCST	bFhbvJ	436723	rs6219891[illegible]	-0,06483	0,079391	0,41416
MCV	Mitral	ebi-a-GCST	bFhbvJ	436723	rs6226075[illegible]	-0,06503	0,079459	0,413146
MCV	Mitral	ebi-a-GCST	bFhbvJ	436723	rs6229295[illegible]	-0,0616	0,079478	0,438296
MCV	Mitral	ebi-a-GCST	bFhbvJ	436723	rs6232971[illegible]	-0,06248	0,079486	0,431809
MCV	Mitral	ebi-a-GCST	bFhbvJ	436723	rs6241053[illegible]	-0,06586	0,079641	0,408273
MCV	Mitral	ebi-a-GCST	bFhbvJ	436723	rs634216	-0,06321	0,079476	0,426404
MCV	Mitral	ebi-a-GCST	bFhbvJ	436723	rs6449591	-0,06462	0,079386	0,415677
MCV	Mitral	ebi-a-GCST	bFhbvJ	436723	rs6449960	-0,06332	0,079521	0,425907
MCV	Mitral	ebi-a-GCST	bFhbvJ	436723	rs6458869	-0,06436	0,079465	0,418016
MCV	Mitral	ebi-a-GCST	bFhbvJ	436723	rs6460528	-0,0603	0,079195	0,44644
MCV	Mitral	ebi-a-GCST	bFhbvJ	436723	rs646179	-0,06564	0,079415	0,408469
MCV	Mitral	ebi-a-GCST	bFhbvJ	436723	rs6472232	-0,06363	0,079475	0,423378
MCV	Mitral	ebi-a-GCST	bFhbvJ	436723	rs6474359	-0,06819	0,079277	0,389734
MCV	Mitral	ebi-a-GCST	bFhbvJ	436723	rs653690	-0,04953	0,079688	0,534246
MCV	Mitral	ebi-a-GCST	bFhbvJ	436723	rs6578125	-0,06027	0,07941	0,447894
MCV	Mitral	ebi-a-GCST	bFhbvJ	436723	rs6592965	-0,07913	0,079704	0,320827

MCV	Mitral	ebi-a-GCST	bFhbvJ	436723	rs661566	-0,06077	0,079313	0,443584
MCV	Mitral	ebi-a-GCST	bFhbvJ	436723	rs6661909	-0,06604	0,079419	0,405662
MCV	Mitral	ebi-a-GCST	bFhbvJ	436723	rs6678627	-0,06885	0,078811	0,382324
MCV	Mitral	ebi-a-GCST	bFhbvJ	436723	rs6697193	-0,05982	0,079062	0,44925
MCV	Mitral	ebi-a-GCST	bFhbvJ	436723	rs6698055	-0,0604	0,079417	0,446966
MCV	Mitral	ebi-a-GCST	bFhbvJ	436723	rs6715064	-0,06585	0,079219	0,405871
MCV	Mitral	ebi-a-GCST	bFhbvJ	436723	rs6730342	-0,06301	0,07948	0,427907
MCV	Mitral	ebi-a-GCST	bFhbvJ	436723	rs6730558	-0,05789	0,079512	0,46655
MCV	Mitral	ebi-a-GCST	bFhbvJ	436723	rs6734238	-0,06225	0,079486	0,433566
MCV	Mitral	ebi-a-GCST	bFhbvJ	436723	rs6746242(	-0,06439	0,079415	0,417469
MCV	Mitral	ebi-a-GCST	bFhbvJ	436723	rs6750645(	-0,06321	0,079477	0,426416
MCV	Mitral	ebi-a-GCST	bFhbvJ	436723	rs6771737	-0,062	0,079498	0,435443
MCV	Mitral	ebi-a-GCST	bFhbvJ	436723	rs6777554	-0,05883	0,079523	0,459441
MCV	Mitral	ebi-a-GCST	bFhbvJ	436723	rs6810965	-0,06129	0,079452	0,440468
MCV	Mitral	ebi-a-GCST	bFhbvJ	436723	rs6814917(	-0,0584	0,079477	0,462443
MCV	Mitral	ebi-a-GCST	bFhbvJ	436723	rs6844176	-0,06569	0,079476	0,408464
MCV	Mitral	ebi-a-GCST	bFhbvJ	436723	rs684562	-0,06408	0,079443	0,419854
MCV	Mitral	ebi-a-GCST	bFhbvJ	436723	rs6854749	-0,05504	0,079272	0,487479
MCV	Mitral	ebi-a-GCST	bFhbvJ	436723	rs6874435	-0,06162	0,079413	0,43778
MCV	Mitral	ebi-a-GCST	bFhbvJ	436723	rs6925032	-0,05813	0,079318	0,463597
MCV	Mitral	ebi-a-GCST	bFhbvJ	436723	rs6934903	-0,0589	0,08115	0,467913
MCV	Mitral	ebi-a-GCST	bFhbvJ	436723	rs6984764	-0,07098	0,078994	0,368902
MCV	Mitral	ebi-a-GCST	bFhbvJ	436723	rs699579	-0,06774	0,078976	0,391075
MCV	Mitral	ebi-a-GCST	bFhbvJ	436723	rs7012705	-0,06286	0,079494	0,429067
MCV	Mitral	ebi-a-GCST	bFhbvJ	436723	rs7041878	-0,06269	0,079524	0,430544
MCV	Mitral	ebi-a-GCST	bFhbvJ	436723	rs707901	-0,05921	0,079877	0,458514
MCV	Mitral	ebi-a-GCST	bFhbvJ	436723	rs7088425	-0,06199	0,079545	0,435806
MCV	Mitral	ebi-a-GCST	bFhbvJ	436723	rs7110899	-0,06199	0,079443	0,435216
MCV	Mitral	ebi-a-GCST	bFhbvJ	436723	rs7117878	-0,05711	0,078965	0,46955
MCV	Mitral	ebi-a-GCST	bFhbvJ	436723	rs7121112	-0,05399	0,079043	0,494572
MCV	Mitral	ebi-a-GCST	bFhbvJ	436723	rs7132545!	-0,06094	0,079417	0,442844
MCV	Mitral	ebi-a-GCST	bFhbvJ	436723	rs7137095	-0,06248	0,079494	0,43189
MCV	Mitral	ebi-a-GCST	bFhbvJ	436723	rs7149479:	-0,05706	0,079727	0,474147
MCV	Mitral	ebi-a-GCST	bFhbvJ	436723	rs7177197	-0,06503	0,079285	0,412076
MCV	Mitral	ebi-a-GCST	bFhbvJ	436723	rs7194355	-0,05904	0,079304	0,456553
MCV	Mitral	ebi-a-GCST	bFhbvJ	436723	rs7204459	-0,06438	0,079529	0,418245
MCV	Mitral	ebi-a-GCST	bFhbvJ	436723	rs7212408	-0,06497	0,079418	0,413293
MCV	Mitral	ebi-a-GCST	bFhbvJ	436723	rs7218708	-0,04836	0,079228	0,541616
MCV	Mitral	ebi-a-GCST	bFhbvJ	436723	rs7251797	-0,06427	0,079406	0,418298
MCV	Mitral	ebi-a-GCST	bFhbvJ	436723	rs725518	-0,06552	0,079514	0,409953
MCV	Mitral	ebi-a-GCST	bFhbvJ	436723	rs7263028	-0,06371	0,079457	0,422688
MCV	Mitral	ebi-a-GCST	bFhbvJ	436723	rs7265567	-0,06449	0,079462	0,417018
MCV	Mitral	ebi-a-GCST	bFhbvJ	436723	rs7266090{	-0,06576	0,079578	0,40863
MCV	Mitral	ebi-a-GCST	bFhbvJ	436723	rs7269879{	-0,06225	0,079655	0,434477
MCV	Mitral	ebi-a-GCST	bFhbvJ	436723	rs7279610(	-0,06068	0,0795	0,445267
MCV	Mitral	ebi-a-GCST	bFhbvJ	436723	rs7287773	-0,06333	0,079485	0,425566
MCV	Mitral	ebi-a-GCST	bFhbvJ	436723	rs7298217!	-0,06573	0,079454	0,408084
MCV	Mitral	ebi-a-GCST	bFhbvJ	436723	rs7299062(	-0,06627	0,079503	0,404542
MCV	Mitral	ebi-a-GCST	bFhbvJ	436723	rs7325021	-0,06343	0,079513	0,425044
MCV	Mitral	ebi-a-GCST	bFhbvJ	436723	rs7328815(	-0,06442	0,079469	0,417576

MCV	Mitral	ebi-a-GCST	bFhbvJ	436723	rs7336989(	-0,06242	0,079495	0,432306
MCV	Mitral	ebi-a-GCST	bFhbvJ	436723	rs741951	-0,06618	0,079501	0,405167
MCV	Mitral	ebi-a-GCST	bFhbvJ	436723	rs7482541	-0,05861	0,07944	0,46066
MCV	Mitral	ebi-a-GCST	bFhbvJ	436723	rs7524028:	-0,06445	0,079597	0,418117
MCV	Mitral	ebi-a-GCST	bFhbvJ	436723	rs754205	-0,06108	0,079502	0,442339
MCV	Mitral	ebi-a-GCST	bFhbvJ	436723	rs7547301(	-0,05945	0,079069	0,452093
MCV	Mitral	ebi-a-GCST	bFhbvJ	436723	rs7560864	-0,06005	0,079341	0,449143
MCV	Mitral	ebi-a-GCST	bFhbvJ	436723	rs7566716:	-0,0594	0,079306	0,453874
MCV	Mitral	ebi-a-GCST	bFhbvJ	436723	rs760719	-0,06466	0,081428	0,427184
MCV	Mitral	ebi-a-GCST	bFhbvJ	436723	rs761590	-0,06434	0,079422	0,417908
MCV	Mitral	ebi-a-GCST	bFhbvJ	436723	rs7630482:	-0,06364	0,079479	0,423308
MCV	Mitral	ebi-a-GCST	bFhbvJ	436723	rs7642571!	-0,06244	0,079474	0,432042
MCV	Mitral	ebi-a-GCST	bFhbvJ	436723	rs7656203{	-0,06232	0,079478	0,432987
MCV	Mitral	ebi-a-GCST	bFhbvJ	436723	rs7665417	-0,06658	0,079298	0,401105
MCV	Mitral	ebi-a-GCST	bFhbvJ	436723	rs7691825	-0,06135	0,079414	0,439806
MCV	Mitral	ebi-a-GCST	bFhbvJ	436723	rs7705509!	-0,05793	0,079486	0,466088
MCV	Mitral	ebi-a-GCST	bFhbvJ	436723	rs7705526	-0,05415	0,079468	0,495639
MCV	Mitral	ebi-a-GCST	bFhbvJ	436723	rs7720871{	-0,06111	0,079338	0,441167
MCV	Mitral	ebi-a-GCST	bFhbvJ	436723	rs7753053:	-0,05867	0,079093	0,458188
MCV	Mitral	ebi-a-GCST	bFhbvJ	436723	rs7754216:	-0,06474	0,079508	0,415518
MCV	Mitral	ebi-a-GCST	bFhbvJ	436723	rs7777063	-0,06526	0,079523	0,411852
MCV	Mitral	ebi-a-GCST	bFhbvJ	436723	rs7805890	-0,06813	0,079392	0,390781
MCV	Mitral	ebi-a-GCST	bFhbvJ	436723	rs7812293	-0,06408	0,079447	0,419909
MCV	Mitral	ebi-a-GCST	bFhbvJ	436723	rs782725	-0,06193	0,079487	0,435879
MCV	Mitral	ebi-a-GCST	bFhbvJ	436723	rs7830832	-0,06146	0,079426	0,439053
MCV	Mitral	ebi-a-GCST	bFhbvJ	436723	rs7837822:	-0,05918	0,079321	0,455599
MCV	Mitral	ebi-a-GCST	bFhbvJ	436723	rs7874418:	-0,06428	0,079838	0,420754
MCV	Mitral	ebi-a-GCST	bFhbvJ	436723	rs7899945(	-0,06473	0,079412	0,415012
MCV	Mitral	ebi-a-GCST	bFhbvJ	436723	rs7906958	-0,06471	0,079384	0,41501
MCV	Mitral	ebi-a-GCST	bFhbvJ	436723	rs7953440	-0,05913	0,07928	0,455757
MCV	Mitral	ebi-a-GCST	bFhbvJ	436723	rs7978096:	-0,0623	0,079464	0,433004
MCV	Mitral	ebi-a-GCST	bFhbvJ	436723	rs798750	-0,06165	0,079407	0,437517
MCV	Mitral	ebi-a-GCST	bFhbvJ	436723	rs7997295	-0,06276	0,079481	0,429737
MCV	Mitral	ebi-a-GCST	bFhbvJ	436723	rs8013143	-0,06624	0,079495	0,404706
MCV	Mitral	ebi-a-GCST	bFhbvJ	436723	rs8015022	-0,06299	0,07955	0,428483
MCV	Mitral	ebi-a-GCST	bFhbvJ	436723	rs806704	-0,06083	0,079413	0,443697
MCV	Mitral	ebi-a-GCST	bFhbvJ	436723	rs8087619	-0,0651	0,07934	0,411907
MCV	Mitral	ebi-a-GCST	bFhbvJ	436723	rs8109008	-0,065	0,079383	0,412913
MCV	Mitral	ebi-a-GCST	bFhbvJ	436723	rs8110787	-0,0667	0,080018	0,404491
MCV	Mitral	ebi-a-GCST	bFhbvJ	436723	rs8126001	-0,06381	0,079491	0,42216
MCV	Mitral	ebi-a-GCST	bFhbvJ	436723	rs8137853	-0,06197	0,079491	0,435609
MCV	Mitral	ebi-a-GCST	bFhbvJ	436723	rs866429	-0,06416	0,079465	0,419461
MCV	Mitral	ebi-a-GCST	bFhbvJ	436723	rs869785	-0,05419	0,079756	0,496836
MCV	Mitral	ebi-a-GCST	bFhbvJ	436723	rs875741	-0,06277	0,079546	0,430015
MCV	Mitral	ebi-a-GCST	bFhbvJ	436723	rs8887	-0,05704	0,079889	0,475243
MCV	Mitral	ebi-a-GCST	bFhbvJ	436723	rs920112	-0,0601	0,079431	0,449303
MCV	Mitral	ebi-a-GCST	bFhbvJ	436723	rs9258357	-0,06237	0,079624	0,433425
MCV	Mitral	ebi-a-GCST	bFhbvJ	436723	rs9264277	-0,06195	0,079513	0,435948
MCV	Mitral	ebi-a-GCST	bFhbvJ	436723	rs9287979	-0,06498	0,07942	0,413253
MCV	Mitral	ebi-a-GCST	bFhbvJ	436723	rs9372860	-0,06245	0,079476	0,432028

MCV	Mitral	ebi-a-GCST	bFhbvJ	436723	rs9381093	-0,06865	0,080736	0,395147
MCV	Mitral	ebi-a-GCST	bFhbvJ	436723	rs939249	-0,06168	0,079408	0,437342
MCV	Mitral	ebi-a-GCST	bFhbvJ	436723	rs9419958	-0,05955	0,0794	0,45323
MCV	Mitral	ebi-a-GCST	bFhbvJ	436723	rs9429742	-0,06036	0,079447	0,447373
MCV	Mitral	ebi-a-GCST	bFhbvJ	436723	rs9438547	-0,06359	0,07952	0,42388
MCV	Mitral	ebi-a-GCST	bFhbvJ	436723	rs9457799	-0,06125	0,079397	0,440472
MCV	Mitral	ebi-a-GCST	bFhbvJ	436723	rs9468618	-0,06434	0,079437	0,417945
MCV	Mitral	ebi-a-GCST	bFhbvJ	436723	rs9471708	-0,10032	0,080832	0,214592
MCV	Mitral	ebi-a-GCST	bFhbvJ	436723	rs9487023	-0,05971	0,080179	0,456465
MCV	Mitral	ebi-a-GCST	bFhbvJ	436723	rs9521026	-0,06409	0,079493	0,42012
MCV	Mitral	ebi-a-GCST	bFhbvJ	436723	rs9532562	-0,06242	0,079532	0,432516
MCV	Mitral	ebi-a-GCST	bFhbvJ	436723	rs9549317	-0,06434	0,079399	0,417715
MCV	Mitral	ebi-a-GCST	bFhbvJ	436723	rs9551453	-0,06159	0,079426	0,438104
MCV	Mitral	ebi-a-GCST	bFhbvJ	436723	rs9579583	-0,06283	0,079501	0,429342
MCV	Mitral	ebi-a-GCST	bFhbvJ	436723	rs9579588	-0,06261	0,079486	0,43085
MCV	Mitral	ebi-a-GCST	bFhbvJ	436723	rs9595815	-0,06266	0,079474	0,430417
MCV	Mitral	ebi-a-GCST	bFhbvJ	436723	rs9672995	-0,06875	0,079175	0,385206
MCV	Mitral	ebi-a-GCST	bFhbvJ	436723	rs9676912	-0,0628	0,079499	0,429567
MCV	Mitral	ebi-a-GCST	bFhbvJ	436723	rs9802945	-0,06197	0,079446	0,435377
MCV	Mitral	ebi-a-GCST	bFhbvJ	436723	rs9817946	-0,06207	0,079446	0,434669
MCV	Mitral	ebi-a-GCST	bFhbvJ	436723	rs9831543	-0,06799	0,079285	0,391145
MCV	Mitral	ebi-a-GCST	bFhbvJ	436723	rs9837654	-0,06357	0,079481	0,423849
MCV	Mitral	ebi-a-GCST	bFhbvJ	436723	rs9882812	-0,05758	0,079088	0,466577
MCV	Mitral	ebi-a-GCST	bFhbvJ	436723	rs9892942	-0,06268	0,079695	0,431586
MCV	Mitral	ebi-a-GCST	bFhbvJ	436723	rs9931440	-0,0635	0,079534	0,424623
MCV	Mitral	ebi-a-GCST	bFhbvJ	436723	All	-0,06297	0,079372	0,427551
MCH	Mitral	ebi-a-GCST	nlYUdJ	424022	rs1002331(	-0,07323	0,081899	0,371241
MCH	Mitral	ebi-a-GCST	nlYUdJ	424022	rs1004867(	-0,06364	0,081541	0,435097
MCH	Mitral	ebi-a-GCST	nlYUdJ	424022	rs1006476	-0,0716	0,081976	0,382457
MCH	Mitral	ebi-a-GCST	nlYUdJ	424022	rs1008313	-0,07058	0,081968	0,389194
MCH	Mitral	ebi-a-GCST	nlYUdJ	424022	rs1018669	-0,07107	0,081961	0,385866
MCH	Mitral	ebi-a-GCST	nlYUdJ	424022	rs1022688	-0,07054	0,081944	0,389342
MCH	Mitral	ebi-a-GCST	nlYUdJ	424022	rs1024901	-0,07061	0,081949	0,388886
MCH	Mitral	ebi-a-GCST	nlYUdJ	424022	rs1027663(	-0,07203	0,081955	0,379435
MCH	Mitral	ebi-a-GCST	nlYUdJ	424022	rs1047891	-0,07276	0,081992	0,374865
MCH	Mitral	ebi-a-GCST	nlYUdJ	424022	rs1075865(	-0,06902	0,082581	0,403264
MCH	Mitral	ebi-a-GCST	nlYUdJ	424022	rs1079356!	-0,07115	0,082189	0,386641
MCH	Mitral	ebi-a-GCST	nlYUdJ	424022	rs1079552	-0,07489	0,081568	0,358539
MCH	Mitral	ebi-a-GCST	nlYUdJ	424022	rs1079954	-0,06902	0,081896	0,399384
MCH	Mitral	ebi-a-GCST	nlYUdJ	424022	rs1084674	-0,0709	0,081966	0,387072
MCH	Mitral	ebi-a-GCST	nlYUdJ	424022	rs1089381	-0,07043	0,081983	0,390329
MCH	Mitral	ebi-a-GCST	nlYUdJ	424022	rs1090015	-0,07308	0,081843	0,371862
MCH	Mitral	ebi-a-GCST	nlYUdJ	424022	rs1090284	-0,0709	0,081964	0,387053
MCH	Mitral	ebi-a-GCST	nlYUdJ	424022	rs1092339	-0,06952	0,082082	0,397032
MCH	Mitral	ebi-a-GCST	nlYUdJ	424022	rs1105517	-0,07466	0,081589	0,360124
MCH	Mitral	ebi-a-GCST	nlYUdJ	424022	rs1106238(	-0,07161	0,081973	0,382354
MCH	Mitral	ebi-a-GCST	nlYUdJ	424022	rs1107276	-0,07064	0,08197	0,388813
MCH	Mitral	ebi-a-GCST	nlYUdJ	424022	rs1109832	-0,07293	0,081936	0,373413
MCH	Mitral	ebi-a-GCST	nlYUdJ	424022	rs1110488	-0,06916	0,081995	0,398976
MCH	Mitral	ebi-a-GCST	nlYUdJ	424022	rs1110699!	-0,06754	0,08184	0,409253

MCH	Mitral	ebi-a-GCST nlYUdJ	424022	rs1113959(	-0,07057	0,08199	0,389421
MCH	Mitral	ebi-a-GCST nlYUdJ	424022	rs1115271(	-0,07431	0,081896	0,364184
MCH	Mitral	ebi-a-GCST nlYUdJ	424022	rs1117737{	-0,06809	0,081634	0,404201
MCH	Mitral	ebi-a-GCST nlYUdJ	424022	rs1119386:	-0,06981	0,08193	0,394146
MCH	Mitral	ebi-a-GCST nlYUdJ	424022	rs1121148(	-0,07359	0,082041	0,369731
MCH	Mitral	ebi-a-GCST nlYUdJ	424022	rs1121264	-0,07269	0,081975	0,375205
MCH	Mitral	ebi-a-GCST nlYUdJ	424022	rs1121896{	-0,0702	0,081972	0,391796
MCH	Mitral	ebi-a-GCST nlYUdJ	424022	rs1122779:	-0,06575	0,08191	0,422135
MCH	Mitral	ebi-a-GCST nlYUdJ	424022	rs1123470:	-0,07103	0,081969	0,386169
MCH	Mitral	ebi-a-GCST nlYUdJ	424022	rs1124466!	-0,06839	0,081839	0,403339
MCH	Mitral	ebi-a-GCST nlYUdJ	424022	rs1125771(	-0,07281	0,081884	0,373927
MCH	Mitral	ebi-a-GCST nlYUdJ	424022	rs1128756!	-0,07403	0,081973	0,3665
MCH	Mitral	ebi-a-GCST nlYUdJ	424022	rs1134634	-0,06691	0,081951	0,414268
MCH	Mitral	ebi-a-GCST nlYUdJ	424022	rs1137082	-0,07214	0,081974	0,378832
MCH	Mitral	ebi-a-GCST nlYUdJ	424022	rs1137193(	-0,07121	0,081962	0,384945
MCH	Mitral	ebi-a-GCST nlYUdJ	424022	rs1139311:	-0,06728	0,081675	0,410106
MCH	Mitral	ebi-a-GCST nlYUdJ	424022	rs1141658!	-0,07533	0,08201	0,358338
MCH	Mitral	ebi-a-GCST nlYUdJ	424022	rs1142316:	-0,06992	0,081969	0,393688
MCH	Mitral	ebi-a-GCST nlYUdJ	424022	rs1150856	-0,07002	0,081898	0,392561
MCH	Mitral	ebi-a-GCST nlYUdJ	424022	rs1152536!	-0,07176	0,082007	0,381546
MCH	Mitral	ebi-a-GCST nlYUdJ	424022	rs1155715	-0,07404	0,081891	0,365912
MCH	Mitral	ebi-a-GCST nlYUdJ	424022	rs1162713{	-0,0761	0,081683	0,351516
MCH	Mitral	ebi-a-GCST nlYUdJ	424022	rs1162748!	-0,07631	0,082409	0,354473
MCH	Mitral	ebi-a-GCST nlYUdJ	424022	rs1162830	-0,07345	0,081899	0,369782
MCH	Mitral	ebi-a-GCST nlYUdJ	424022	rs1163820	-0,07258	0,081935	0,375734
MCH	Mitral	ebi-a-GCST nlYUdJ	424022	rs1168864:	-0,07358	0,081812	0,368461
MCH	Mitral	ebi-a-GCST nlYUdJ	424022	rs1171076(	-0,07492	0,081774	0,359584
MCH	Mitral	ebi-a-GCST nlYUdJ	424022	rs1171119:	-0,07473	0,081724	0,360504
MCH	Mitral	ebi-a-GCST nlYUdJ	424022	rs1172337:	-0,07017	0,082016	0,392242
MCH	Mitral	ebi-a-GCST nlYUdJ	424022	rs1172472!	-0,06879	0,0818	0,400404
MCH	Mitral	ebi-a-GCST nlYUdJ	424022	rs1178731:	-0,06948	0,081903	0,396231
MCH	Mitral	ebi-a-GCST nlYUdJ	424022	rs1180760:	-0,06915	0,081863	0,398285
MCH	Mitral	ebi-a-GCST nlYUdJ	424022	rs1181396{	-0,07288	0,081878	0,373399
MCH	Mitral	ebi-a-GCST nlYUdJ	424022	rs1188351:	-0,0701	0,081915	0,392149
MCH	Mitral	ebi-a-GCST nlYUdJ	424022	rs118839	-0,06899	0,081785	0,398899
MCH	Mitral	ebi-a-GCST nlYUdJ	424022	rs1190541	-0,07046	0,081943	0,389881
MCH	Mitral	ebi-a-GCST nlYUdJ	424022	rs1199558	-0,07164	0,081982	0,382173
MCH	Mitral	ebi-a-GCST nlYUdJ	424022	rs1219394{	-0,07289	0,082029	0,374195
MCH	Mitral	ebi-a-GCST nlYUdJ	424022	rs1219522	-0,07467	0,081783	0,361249
MCH	Mitral	ebi-a-GCST nlYUdJ	424022	rs1223237!	-0,07115	0,082007	0,385579
MCH	Mitral	ebi-a-GCST nlYUdJ	424022	rs1224712	-0,07172	0,081958	0,381511
MCH	Mitral	ebi-a-GCST nlYUdJ	424022	rs1234415!	-0,07199	0,081946	0,379657
MCH	Mitral	ebi-a-GCST nlYUdJ	424022	rs1243243	-0,06801	0,081842	0,405981
MCH	Mitral	ebi-a-GCST nlYUdJ	424022	rs1245002{	-0,06741	0,081556	0,408479
MCH	Mitral	ebi-a-GCST nlYUdJ	424022	rs1247422:	-0,07032	0,081948	0,390855
MCH	Mitral	ebi-a-GCST nlYUdJ	424022	rs1249058:	-0,07017	0,081941	0,39179
MCH	Mitral	ebi-a-GCST nlYUdJ	424022	rs1250561(	-0,07347	0,08202	0,370364
MCH	Mitral	ebi-a-GCST nlYUdJ	424022	rs1258217(	-0,07344	0,081988	0,37041
MCH	Mitral	ebi-a-GCST nlYUdJ	424022	rs1258246:	-0,06648	0,081841	0,416615
MCH	Mitral	ebi-a-GCST nlYUdJ	424022	rs1259697:	-0,07082	0,081955	0,387517

MCH	Mitral	ebi-a-GCST	nlYUdJ	424022	rs1275189	-0,07102	0,081972	0,386264
MCH	Mitral	ebi-a-GCST	nlYUdJ	424022	rs1280346[illegible]	-0,0714	0,081968	0,383701
MCH	Mitral	ebi-a-GCST	nlYUdJ	424022	rs1300770[illegible]	-0,06899	0,081963	0,399943
MCH	Mitral	ebi-a-GCST	nlYUdJ	424022	rs130436	-0,06872	0,082099	0,40254
MCH	Mitral	ebi-a-GCST	nlYUdJ	424022	rs1318907[illegible]	-0,07163	0,081971	0,382204
MCH	Mitral	ebi-a-GCST	nlYUdJ	424022	rs1325027[illegible]	-0,06811	0,081943	0,405845
MCH	Mitral	ebi-a-GCST	nlYUdJ	424022	rs1330678[illegible]	-0,07468	0,081991	0,362386
MCH	Mitral	ebi-a-GCST	nlYUdJ	424022	rs1332101	-0,07359	0,082001	0,369492
MCH	Mitral	ebi-a-GCST	nlYUdJ	424022	rs1335536[illegible]	-0,07281	0,081881	0,373912
MCH	Mitral	ebi-a-GCST	nlYUdJ	424022	rs1393627[illegible]	-0,07267	0,081941	0,375141
MCH	Mitral	ebi-a-GCST	nlYUdJ	424022	rs1404256[illegible]	-0,07418	0,081754	0,364229
MCH	Mitral	ebi-a-GCST	nlYUdJ	424022	rs1404467[illegible]	-0,07005	0,081986	0,39288
MCH	Mitral	ebi-a-GCST	nlYUdJ	424022	rs140522	-0,06984	0,082554	0,397541
MCH	Mitral	ebi-a-GCST	nlYUdJ	424022	rs1406696	-0,07448	0,081737	0,362161
MCH	Mitral	ebi-a-GCST	nlYUdJ	424022	rs1414946[illegible]	-0,07483	0,082158	0,362418
MCH	Mitral	ebi-a-GCST	nlYUdJ	424022	rs1417922	-0,07193	0,081946	0,380055
MCH	Mitral	ebi-a-GCST	nlYUdJ	424022	rs1429804[illegible]	-0,07098	0,081975	0,386579
MCH	Mitral	ebi-a-GCST	nlYUdJ	424022	rs1437475[illegible]	-0,07162	0,081964	0,382254
MCH	Mitral	ebi-a-GCST	nlYUdJ	424022	rs1442045[illegible]	-0,07115	0,082015	0,385648
MCH	Mitral	ebi-a-GCST	nlYUdJ	424022	rs1449427[illegible]	-0,07097	0,081961	0,386514
MCH	Mitral	ebi-a-GCST	nlYUdJ	424022	rs1460089[illegible]	-0,07054	0,081972	0,389477
MCH	Mitral	ebi-a-GCST	nlYUdJ	424022	rs1475518[illegible]	-0,0738	0,082033	0,368294
MCH	Mitral	ebi-a-GCST	nlYUdJ	424022	rs1482572[illegible]	-0,0706	0,081973	0,389097
MCH	Mitral	ebi-a-GCST	nlYUdJ	424022	rs1492903[illegible]	-0,07137	0,082041	0,384358
MCH	Mitral	ebi-a-GCST	nlYUdJ	424022	rs1504253[illegible]	-0,06988	0,081956	0,393826
MCH	Mitral	ebi-a-GCST	nlYUdJ	424022	rs1508443[illegible]	-0,06884	0,081925	0,400778
MCH	Mitral	ebi-a-GCST	nlYUdJ	424022	rs1550861	-0,07215	0,081944	0,378578
MCH	Mitral	ebi-a-GCST	nlYUdJ	424022	rs1556069	-0,06962	0,081876	0,395159
MCH	Mitral	ebi-a-GCST	nlYUdJ	424022	rs1569419	-0,07289	0,081998	0,374055
MCH	Mitral	ebi-a-GCST	nlYUdJ	424022	rs159058	-0,07184	0,082019	0,381087
MCH	Mitral	ebi-a-GCST	nlYUdJ	424022	rs1684334[illegible]	-0,06685	0,082137	0,415698
MCH	Mitral	ebi-a-GCST	nlYUdJ	424022	rs1694830[illegible]	-0,07014	0,081933	0,391986
MCH	Mitral	ebi-a-GCST	nlYUdJ	424022	rs1696775[illegible]	-0,07036	0,081942	0,390534
MCH	Mitral	ebi-a-GCST	nlYUdJ	424022	rs1701516[illegible]	-0,06915	0,08192	0,398581
MCH	Mitral	ebi-a-GCST	nlYUdJ	424022	rs1706865[illegible]	-0,06849	0,081911	0,403103
MCH	Mitral	ebi-a-GCST	nlYUdJ	424022	rs1711638[illegible]	-0,0688	0,081942	0,401112
MCH	Mitral	ebi-a-GCST	nlYUdJ	424022	rs1711671[illegible]	-0,07586	0,08177	0,353536
MCH	Mitral	ebi-a-GCST	nlYUdJ	424022	rs1730215[illegible]	-0,07243	0,081942	0,376747
MCH	Mitral	ebi-a-GCST	nlYUdJ	424022	rs1746264[illegible]	-0,07498	0,081703	0,35878
MCH	Mitral	ebi-a-GCST	nlYUdJ	424022	rs1747636[illegible]	-0,06252	0,082109	0,4464
MCH	Mitral	ebi-a-GCST	nlYUdJ	424022	rs1760801	-0,07424	0,081813	0,364173
MCH	Mitral	ebi-a-GCST	nlYUdJ	424022	rs1769965[illegible]	-0,07266	0,081982	0,375461
MCH	Mitral	ebi-a-GCST	nlYUdJ	424022	rs1772858[illegible]	-0,0697	0,081935	0,394944
MCH	Mitral	ebi-a-GCST	nlYUdJ	424022	rs1775869[illegible]	-0,06894	0,082075	0,400914
MCH	Mitral	ebi-a-GCST	nlYUdJ	424022	rs1811069	-0,07059	0,082054	0,389658
MCH	Mitral	ebi-a-GCST	nlYUdJ	424022	rs1871930[illegible]	-0,06885	0,081983	0,401027
MCH	Mitral	ebi-a-GCST	nlYUdJ	424022	rs1880791[illegible]	-0,07075	0,081964	0,388024
MCH	Mitral	ebi-a-GCST	nlYUdJ	424022	rs1913467	-0,0716	0,081965	0,382389
MCH	Mitral	ebi-a-GCST	nlYUdJ	424022	rs1935958	-0,06954	0,081898	0,395855
MCH	Mitral	ebi-a-GCST	nlYUdJ	424022	rs1957391	-0,07517	0,081809	0,358169

MCH	Mitral	ebi-a-GCST nlYUdJ	424022	rs1958078	-0,07051	0,081998	0,389864
MCH	Mitral	ebi-a-GCST nlYUdJ	424022	rs198358	-0,07316	0,081955	0,372059
MCH	Mitral	ebi-a-GCST nlYUdJ	424022	rs1992383	-0,06957	0,082014	0,396268
MCH	Mitral	ebi-a-GCST nlYUdJ	424022	rs2005277	-0,07023	0,081952	0,391443
MCH	Mitral	ebi-a-GCST nlYUdJ	424022	rs2020204!	-0,07247	0,081936	0,37643
MCH	Mitral	ebi-a-GCST nlYUdJ	424022	rs2067663	-0,07077	0,082017	0,388195
MCH	Mitral	ebi-a-GCST nlYUdJ	424022	rs2071243	-0,06873	0,081846	0,401065
MCH	Mitral	ebi-a-GCST nlYUdJ	424022	rs2075672	-0,08647	0,082725	0,295898
MCH	Mitral	ebi-a-GCST nlYUdJ	424022	rs2137283	-0,06361	0,082173	0,438905
MCH	Mitral	ebi-a-GCST nlYUdJ	424022	rs2140875	-0,07228	0,081953	0,377783
MCH	Mitral	ebi-a-GCST nlYUdJ	424022	rs2150165	-0,07098	0,081969	0,38653
MCH	Mitral	ebi-a-GCST nlYUdJ	424022	rs218264	-0,08074	0,08286	0,329849
MCH	Mitral	ebi-a-GCST nlYUdJ	424022	rs2208649	-0,07548	0,081578	0,354832
MCH	Mitral	ebi-a-GCST nlYUdJ	424022	rs2237648	-0,07165	0,081963	0,381991
MCH	Mitral	ebi-a-GCST nlYUdJ	424022	rs2238368	-0,04733	0,082491	0,566105
MCH	Mitral	ebi-a-GCST nlYUdJ	424022	rs2240816	-0,07433	0,081757	0,363235
MCH	Mitral	ebi-a-GCST nlYUdJ	424022	rs2243834	-0,0736	0,081988	0,369337
MCH	Mitral	ebi-a-GCST nlYUdJ	424022	rs2251551	-0,07005	0,081907	0,392409
MCH	Mitral	ebi-a-GCST nlYUdJ	424022	rs2273799	-0,06763	0,08199	0,409442
MCH	Mitral	ebi-a-GCST nlYUdJ	424022	rs2298715	-0,0737	0,08182	0,367703
MCH	Mitral	ebi-a-GCST nlYUdJ	424022	rs2300150	-0,07198	0,081967	0,379877
MCH	Mitral	ebi-a-GCST nlYUdJ	424022	rs2323983	-0,07115	0,081966	0,385338
MCH	Mitral	ebi-a-GCST nlYUdJ	424022	rs2351959	-0,07204	0,08195	0,379378
MCH	Mitral	ebi-a-GCST nlYUdJ	424022	rs2361710	-0,07211	0,081956	0,37894
MCH	Mitral	ebi-a-GCST nlYUdJ	424022	rs238540	-0,07409	0,081741	0,364707
MCH	Mitral	ebi-a-GCST nlYUdJ	424022	rs2393750	-0,07263	0,081927	0,375337
MCH	Mitral	ebi-a-GCST nlYUdJ	424022	rs2422277	-0,06752	0,081867	0,409518
MCH	Mitral	ebi-a-GCST nlYUdJ	424022	rs2439423	-0,07199	0,081968	0,379782
MCH	Mitral	ebi-a-GCST nlYUdJ	424022	rs2468024	-0,07268	0,081997	0,375441
MCH	Mitral	ebi-a-GCST nlYUdJ	424022	rs2492301	-0,07578	0,081848	0,354518
MCH	Mitral	ebi-a-GCST nlYUdJ	424022	rs2501369	-0,0714	0,081972	0,38371
MCH	Mitral	ebi-a-GCST nlYUdJ	424022	rs2503772	-0,06849	0,081663	0,401665
MCH	Mitral	ebi-a-GCST nlYUdJ	424022	rs250737	-0,0687	0,081737	0,400656
MCH	Mitral	ebi-a-GCST nlYUdJ	424022	rs2537594	-0,07286	0,08199	0,374166
MCH	Mitral	ebi-a-GCST nlYUdJ	424022	rs2556096	-0,07257	0,081956	0,37589
MCH	Mitral	ebi-a-GCST nlYUdJ	424022	rs2577775	-0,07073	0,081954	0,388142
MCH	Mitral	ebi-a-GCST nlYUdJ	424022	rs26234	-0,07291	0,081963	0,373725
MCH	Mitral	ebi-a-GCST nlYUdJ	424022	rs2657907	-0,07142	0,081968	0,383552
MCH	Mitral	ebi-a-GCST nlYUdJ	424022	rs2661794	-0,07202	0,081994	0,379764
MCH	Mitral	ebi-a-GCST nlYUdJ	424022	rs2672092	-0,0761	0,081447	0,350128
MCH	Mitral	ebi-a-GCST nlYUdJ	424022	rs2692685	-0,07015	0,081938	0,391947
MCH	Mitral	ebi-a-GCST nlYUdJ	424022	rs2713936	-0,06924	0,081978	0,398327
MCH	Mitral	ebi-a-GCST nlYUdJ	424022	rs271620	-0,06862	0,082029	0,402831
MCH	Mitral	ebi-a-GCST nlYUdJ	424022	rs2767482	-0,06556	0,08171	0,422365
MCH	Mitral	ebi-a-GCST nlYUdJ	424022	rs2796459	-0,06811	0,081753	0,404746
MCH	Mitral	ebi-a-GCST nlYUdJ	424022	rs2811972	-0,06653	0,081937	0,416814
MCH	Mitral	ebi-a-GCST nlYUdJ	424022	rs2834257	-0,06467	0,082011	0,430343
MCH	Mitral	ebi-a-GCST nlYUdJ	424022	rs2834712	-0,07502	0,081765	0,358853
MCH	Mitral	ebi-a-GCST nlYUdJ	424022	rs2836422	-0,06986	0,081956	0,394004
MCH	Mitral	ebi-a-GCST nlYUdJ	424022	rs2845847	-0,06996	0,081908	0,393009

MCH	Mitral	ebi-a-GCST	nlYUdJ	424022	rs2966450	-0,07455	0,081803	0,362134
MCH	Mitral	ebi-a-GCST	nlYUdJ	424022	rs29743	-0,06986	0,081904	0,393667
MCH	Mitral	ebi-a-GCST	nlYUdJ	424022	rs3109917	-0,07222	0,081931	0,37805
MCH	Mitral	ebi-a-GCST	nlYUdJ	424022	rs314751	-0,06852	0,081812	0,402308
MCH	Mitral	ebi-a-GCST	nlYUdJ	424022	rs3173805	-0,07235	0,081922	0,377146
MCH	Mitral	ebi-a-GCST	nlYUdJ	424022	rs320370	-0,07666	0,081796	0,348634
MCH	Mitral	ebi-a-GCST	nlYUdJ	424022	rs322694	-0,07191	0,081946	0,380225
MCH	Mitral	ebi-a-GCST	nlYUdJ	424022	rs322873	-0,0673	0,081828	0,410818
MCH	Mitral	ebi-a-GCST	nlYUdJ	424022	rs33674	-0,07193	0,081963	0,380148
MCH	Mitral	ebi-a-GCST	nlYUdJ	424022	rs3451483(	-0,07193	0,081969	0,380171
MCH	Mitral	ebi-a-GCST	nlYUdJ	424022	rs3452308!	-0,07377	0,081881	0,367615
MCH	Mitral	ebi-a-GCST	nlYUdJ	424022	rs3463927.	-0,07276	0,081938	0,374539
MCH	Mitral	ebi-a-GCST	nlYUdJ	424022	rs34651	-0,07249	0,081949	0,376384
MCH	Mitral	ebi-a-GCST	nlYUdJ	424022	rs3465854:	-0,07195	0,081967	0,380064
MCH	Mitral	ebi-a-GCST	nlYUdJ	424022	rs3489148!	-0,07173	0,081958	0,381458
MCH	Mitral	ebi-a-GCST	nlYUdJ	424022	rs3490468	-0,0724	0,081931	0,376885
MCH	Mitral	ebi-a-GCST	nlYUdJ	424022	rs3493119!	-0,06696	0,081986	0,414075
MCH	Mitral	ebi-a-GCST	nlYUdJ	424022	rs3515020:	-0,06805	0,081811	0,405503
MCH	Mitral	ebi-a-GCST	nlYUdJ	424022	rs3515898!	-0,06833	0,081949	0,404407
MCH	Mitral	ebi-a-GCST	nlYUdJ	424022	rs3536096	-0,07426	0,082195	0,366291
MCH	Mitral	ebi-a-GCST	nlYUdJ	424022	rs3560260!	-0,06909	0,081979	0,399354
MCH	Mitral	ebi-a-GCST	nlYUdJ	424022	rs3599852	-0,06629	0,08221	0,420066
MCH	Mitral	ebi-a-GCST	nlYUdJ	424022	rs3719189:	-0,07357	0,081932	0,369201
MCH	Mitral	ebi-a-GCST	nlYUdJ	424022	rs3740685	-0,06825	0,081929	0,404841
MCH	Mitral	ebi-a-GCST	nlYUdJ	424022	rs3756668	-0,07003	0,081997	0,393041
MCH	Mitral	ebi-a-GCST	nlYUdJ	424022	rs3760137	-0,07101	0,081978	0,386351
MCH	Mitral	ebi-a-GCST	nlYUdJ	424022	rs3768321	-0,06504	0,081769	0,426382
MCH	Mitral	ebi-a-GCST	nlYUdJ	424022	rs3809627	-0,06884	0,08211	0,401846
MCH	Mitral	ebi-a-GCST	nlYUdJ	424022	rs3811444	-0,07151	0,082077	0,383623
MCH	Mitral	ebi-a-GCST	nlYUdJ	424022	rs381500	-0,07798	0,081934	0,341229
MCH	Mitral	ebi-a-GCST	nlYUdJ	424022	rs3822750	-0,07545	0,081336	0,353588
MCH	Mitral	ebi-a-GCST	nlYUdJ	424022	rs3847694	-0,07129	0,081972	0,384489
MCH	Mitral	ebi-a-GCST	nlYUdJ	424022	rs395564	-0,07644	0,081835	0,350266
MCH	Mitral	ebi-a-GCST	nlYUdJ	424022	rs4129808	-0,05652	0,082322	0,49235
MCH	Mitral	ebi-a-GCST	nlYUdJ	424022	rs4144580	-0,07335	0,081809	0,369911
MCH	Mitral	ebi-a-GCST	nlYUdJ	424022	rs4149056	-0,07758	0,081469	0,340977
MCH	Mitral	ebi-a-GCST	nlYUdJ	424022	rs42042	-0,07047	0,081969	0,389942
MCH	Mitral	ebi-a-GCST	nlYUdJ	424022	rs429961	-0,08882	0,082342	0,280719
MCH	Mitral	ebi-a-GCST	nlYUdJ	424022	rs4332427	-0,07163	0,081994	0,38231
MCH	Mitral	ebi-a-GCST	nlYUdJ	424022	rs4378991	-0,07226	0,081988	0,3781
MCH	Mitral	ebi-a-GCST	nlYUdJ	424022	rs440837	-0,0679	0,081508	0,404833
MCH	Mitral	ebi-a-GCST	nlYUdJ	424022	rs4472338	-0,07658	0,081888	0,3497
MCH	Mitral	ebi-a-GCST	nlYUdJ	424022	rs4496693	-0,07088	0,081957	0,387118
MCH	Mitral	ebi-a-GCST	nlYUdJ	424022	rs4585697	-0,07275	0,081899	0,37441
MCH	Mitral	ebi-a-GCST	nlYUdJ	424022	rs4672884	-0,06895	0,081781	0,399173
MCH	Mitral	ebi-a-GCST	nlYUdJ	424022	rs4680338	-0,0722	0,081933	0,378198
MCH	Mitral	ebi-a-GCST	nlYUdJ	424022	rs4777602	-0,06869	0,081857	0,401374
MCH	Mitral	ebi-a-GCST	nlYUdJ	424022	rs4778058	-0,07492	0,08153	0,358123
MCH	Mitral	ebi-a-GCST	nlYUdJ	424022	rs4779585	-0,07227	0,081946	0,377839
MCH	Mitral	ebi-a-GCST	nlYUdJ	424022	rs4878598	-0,07261	0,08191	0,375374

MCH	Mitral	ebi-a-GCST	nlYUdJ	424022	rs4890628	-0,0626	0,082359	0,447171
MCH	Mitral	ebi-a-GCST	nlYUdJ	424022	rs496321	-0,07119	0,082009	0,385385
MCH	Mitral	ebi-a-GCST	nlYUdJ	424022	rs4970485	-0,07203	0,081974	0,37956
MCH	Mitral	ebi-a-GCST	nlYUdJ	424022	rs500422	-0,07507	0,081707	0,358219
MCH	Mitral	ebi-a-GCST	nlYUdJ	424022	rs507964	-0,06824	0,081809	0,404229
MCH	Mitral	ebi-a-GCST	nlYUdJ	424022	rs522749	-0,0711	0,081983	0,385838
MCH	Mitral	ebi-a-GCST	nlYUdJ	424022	rs5564155	-0,06894	0,081941	0,40013
MCH	Mitral	ebi-a-GCST	nlYUdJ	424022	rs5564881	-0,0743	0,081928	0,364434
MCH	Mitral	ebi-a-GCST	nlYUdJ	424022	rs5571612	-0,06751	0,081466	0,407297
MCH	Mitral	ebi-a-GCST	nlYUdJ	424022	rs5576021	-0,07314	0,081916	0,371954
MCH	Mitral	ebi-a-GCST	nlYUdJ	424022	rs5593813	-0,07241	0,081952	0,376902
MCH	Mitral	ebi-a-GCST	nlYUdJ	424022	rs5608492	-0,07457	0,081781	0,361845
MCH	Mitral	ebi-a-GCST	nlYUdJ	424022	rs5612910	-0,07381	0,081903	0,367465
MCH	Mitral	ebi-a-GCST	nlYUdJ	424022	rs5614270	-0,0567	0,081691	0,487596
MCH	Mitral	ebi-a-GCST	nlYUdJ	424022	rs5622352	-0,07268	0,082091	0,375942
MCH	Mitral	ebi-a-GCST	nlYUdJ	424022	rs5635638	-0,07154	0,082169	0,383932
MCH	Mitral	ebi-a-GCST	nlYUdJ	424022	rs5719970	-0,07305	0,08188	0,372279
MCH	Mitral	ebi-a-GCST	nlYUdJ	424022	rs5743204	-0,06512	0,081735	0,425611
MCH	Mitral	ebi-a-GCST	nlYUdJ	424022	rs5750254	-0,0713	0,082024	0,384739
MCH	Mitral	ebi-a-GCST	nlYUdJ	424022	rs5756503	-0,07231	0,081942	0,377532
MCH	Mitral	ebi-a-GCST	nlYUdJ	424022	rs5811125	-0,07455	0,081756	0,361862
MCH	Mitral	ebi-a-GCST	nlYUdJ	424022	rs5814140	-0,05666	0,081466	0,486763
MCH	Mitral	ebi-a-GCST	nlYUdJ	424022	rs5912218	-0,07125	0,081968	0,38471
MCH	Mitral	ebi-a-GCST	nlYUdJ	424022	rs595982	-0,0718	0,081971	0,381085
MCH	Mitral	ebi-a-GCST	nlYUdJ	424022	rs5994579	-0,06469	0,082199	0,431311
MCH	Mitral	ebi-a-GCST	nlYUdJ	424022	rs6014993	-0,07943	0,082417	0,335189
MCH	Mitral	ebi-a-GCST	nlYUdJ	424022	rs604256	-0,06868	0,081981	0,402163
MCH	Mitral	ebi-a-GCST	nlYUdJ	424022	rs6060987	-0,07384	0,082016	0,367928
MCH	Mitral	ebi-a-GCST	nlYUdJ	424022	rs6069712	-0,076	0,081606	0,351722
MCH	Mitral	ebi-a-GCST	nlYUdJ	424022	rs6073289	-0,07112	0,082002	0,385758
MCH	Mitral	ebi-a-GCST	nlYUdJ	424022	rs6073958	-0,07178	0,081999	0,381384
MCH	Mitral	ebi-a-GCST	nlYUdJ	424022	rs6075741	-0,072	0,082043	0,380158
MCH	Mitral	ebi-a-GCST	nlYUdJ	424022	rs6083326	-0,07086	0,081984	0,387423
MCH	Mitral	ebi-a-GCST	nlYUdJ	424022	rs6084653	-0,06479	0,08158	0,427057
MCH	Mitral	ebi-a-GCST	nlYUdJ	424022	rs6090040	-0,07359	0,081853	0,368633
MCH	Mitral	ebi-a-GCST	nlYUdJ	424022	rs6131276	-0,07377	0,081827	0,367276
MCH	Mitral	ebi-a-GCST	nlYUdJ	424022	rs6182397	-0,07481	0,08217	0,362612
MCH	Mitral	ebi-a-GCST	nlYUdJ	424022	rs6182872	-0,07097	0,081975	0,386624
MCH	Mitral	ebi-a-GCST	nlYUdJ	424022	rs6204584	-0,07067	0,082014	0,388887
MCH	Mitral	ebi-a-GCST	nlYUdJ	424022	rs6220812	-0,07321	0,081859	0,371137
MCH	Mitral	ebi-a-GCST	nlYUdJ	424022	rs6227077	-0,06923	0,08182	0,39747
MCH	Mitral	ebi-a-GCST	nlYUdJ	424022	rs6241053	-0,07489	0,082199	0,36223
MCH	Mitral	ebi-a-GCST	nlYUdJ	424022	rs6458869	-0,07288	0,081951	0,373816
MCH	Mitral	ebi-a-GCST	nlYUdJ	424022	rs6460528	-0,06831	0,081643	0,402776
MCH	Mitral	ebi-a-GCST	nlYUdJ	424022	rs646179	-0,07427	0,081892	0,364462
MCH	Mitral	ebi-a-GCST	nlYUdJ	424022	rs6512645	-0,07414	0,081926	0,365477
MCH	Mitral	ebi-a-GCST	nlYUdJ	424022	rs653690	-0,0592	0,08203	0,470482
MCH	Mitral	ebi-a-GCST	nlYUdJ	424022	rs6578125	-0,06825	0,08189	0,404564
MCH	Mitral	ebi-a-GCST	nlYUdJ	424022	rs6592965	-0,08538	0,08201	0,297827
MCH	Mitral	ebi-a-GCST	nlYUdJ	424022	rs6673121	-0,07152	0,081964	0,382895

MCH	Mitral	ebi-a-GCST	nlYUdJ	424022	rs6711700	-0,06864	0,081913	0,40203
MCH	Mitral	ebi-a-GCST	nlYUdJ	424022	rs6717013	-0,06933	0,081842	0,396948
MCH	Mitral	ebi-a-GCST	nlYUdJ	424022	rs6730558	-0,06762	0,081902	0,409029
MCH	Mitral	ebi-a-GCST	nlYUdJ	424022	rs6734238	-0,07033	0,08199	0,391032
MCH	Mitral	ebi-a-GCST	nlYUdJ	424022	rs6772028	-0,07395	0,081964	0,366923
MCH	Mitral	ebi-a-GCST	nlYUdJ	424022	rs6773362	-0,06873	0,081782	0,400701
MCH	Mitral	ebi-a-GCST	nlYUdJ	424022	rs6777554	-0,06704	0,081996	0,413584
MCH	Mitral	ebi-a-GCST	nlYUdJ	424022	rs6802828	-0,06985	0,081879	0,393633
MCH	Mitral	ebi-a-GCST	nlYUdJ	424022	rs6803535	-0,07516	0,081934	0,358986
MCH	Mitral	ebi-a-GCST	nlYUdJ	424022	rs6814917	-0,06667	0,081943	0,415899
MCH	Mitral	ebi-a-GCST	nlYUdJ	424022	rs6818511	-0,07408	0,081791	0,365076
MCH	Mitral	ebi-a-GCST	nlYUdJ	424022	rs6844176	-0,07392	0,081943	0,366983
MCH	Mitral	ebi-a-GCST	nlYUdJ	424022	rs6854749	-0,06325	0,081711	0,438856
MCH	Mitral	ebi-a-GCST	nlYUdJ	424022	rs6925032	-0,06629	0,081773	0,417556
MCH	Mitral	ebi-a-GCST	nlYUdJ	424022	rs6934711	-0,07023	0,082031	0,391909
MCH	Mitral	ebi-a-GCST	nlYUdJ	424022	rs6934903	-0,06714	0,084283	0,425669
MCH	Mitral	ebi-a-GCST	nlYUdJ	424022	rs6984764	-0,07958	0,081403	0,328292
MCH	Mitral	ebi-a-GCST	nlYUdJ	424022	rs699579	-0,07564	0,081385	0,352695
MCH	Mitral	ebi-a-GCST	nlYUdJ	424022	rs7041878	-0,07102	0,081992	0,386404
MCH	Mitral	ebi-a-GCST	nlYUdJ	424022	rs7072014	-0,0698	0,081908	0,394104
MCH	Mitral	ebi-a-GCST	nlYUdJ	424022	rs7088425	-0,07021	0,082042	0,392096
MCH	Mitral	ebi-a-GCST	nlYUdJ	424022	rs7117878	-0,06434	0,08139	0,429235
MCH	Mitral	ebi-a-GCST	nlYUdJ	424022	rs7121112	-0,06159	0,081468	0,449655
MCH	Mitral	ebi-a-GCST	nlYUdJ	424022	rs7132546	-0,07589	0,081568	0,352194
MCH	Mitral	ebi-a-GCST	nlYUdJ	424022	rs7137095	-0,07084	0,081968	0,387436
MCH	Mitral	ebi-a-GCST	nlYUdJ	424022	rs7146002	-0,07131	0,081963	0,384321
MCH	Mitral	ebi-a-GCST	nlYUdJ	424022	rs7153233	-0,07117	0,081964	0,38525
MCH	Mitral	ebi-a-GCST	nlYUdJ	424022	rs7155375	-0,07414	0,081792	0,364692
MCH	Mitral	ebi-a-GCST	nlYUdJ	424022	rs7250362	-0,06935	0,081809	0,396583
MCH	Mitral	ebi-a-GCST	nlYUdJ	424022	rs725518	-0,07383	0,081987	0,367863
MCH	Mitral	ebi-a-GCST	nlYUdJ	424022	rs7265551	-0,06985	0,081884	0,393625
MCH	Mitral	ebi-a-GCST	nlYUdJ	424022	rs7279176	-0,07134	0,081964	0,384096
MCH	Mitral	ebi-a-GCST	nlYUdJ	424022	rs7279610	-0,06925	0,081958	0,398136
MCH	Mitral	ebi-a-GCST	nlYUdJ	424022	rs7283484	-0,07107	0,081973	0,385931
MCH	Mitral	ebi-a-GCST	nlYUdJ	424022	rs7287773	-0,07166	0,081969	0,381991
MCH	Mitral	ebi-a-GCST	nlYUdJ	424022	rs7298217	-0,07457	0,081946	0,362826
MCH	Mitral	ebi-a-GCST	nlYUdJ	424022	rs7298874	-0,07416	0,081952	0,365483
MCH	Mitral	ebi-a-GCST	nlYUdJ	424022	rs7304121	-0,07246	0,081921	0,376414
MCH	Mitral	ebi-a-GCST	nlYUdJ	424022	rs732520	-0,06325	0,081638	0,438495
MCH	Mitral	ebi-a-GCST	nlYUdJ	424022	rs7328815	-0,07242	0,081934	0,376771
MCH	Mitral	ebi-a-GCST	nlYUdJ	424022	rs7336989	-0,07068	0,081982	0,3886
MCH	Mitral	ebi-a-GCST	nlYUdJ	424022	rs7365151	-0,07505	0,081536	0,357318
MCH	Mitral	ebi-a-GCST	nlYUdJ	424022	rs7383931	-0,06999	0,08191	0,392851
MCH	Mitral	ebi-a-GCST	nlYUdJ	424022	rs7399292	-0,07126	0,081964	0,384607
MCH	Mitral	ebi-a-GCST	nlYUdJ	424022	rs741951	-0,07476	0,081986	0,361817
MCH	Mitral	ebi-a-GCST	nlYUdJ	424022	rs7439258	-0,07666	0,081714	0,348165
MCH	Mitral	ebi-a-GCST	nlYUdJ	424022	rs7474773	-0,07062	0,081983	0,388992
MCH	Mitral	ebi-a-GCST	nlYUdJ	424022	rs7482541	-0,06672	0,081912	0,415343
MCH	Mitral	ebi-a-GCST	nlYUdJ	424022	rs748968	-0,07364	0,081978	0,369
MCH	Mitral	ebi-a-GCST	nlYUdJ	424022	rs7502345	-0,067	0,081536	0,41127

MCH	Mitral	ebi-a-GCST	nlYUdJ	424022	rs7511310(	-0,0728	0,081857	0,373836
MCH	Mitral	ebi-a-GCST	nlYUdJ	424022	rs7524028:	-0,07277	0,082073	0,37524
MCH	Mitral	ebi-a-GCST	nlYUdJ	424022	rs754205	-0,06909	0,082002	0,399471
MCH	Mitral	ebi-a-GCST	nlYUdJ	424022	rs7545753:	-0,07127	0,082003	0,384802
MCH	Mitral	ebi-a-GCST	nlYUdJ	424022	rs7566716:	-0,06737	0,081769	0,410012
MCH	Mitral	ebi-a-GCST	nlYUdJ	424022	rs7573697(	-0,07006	0,081908	0,392376
MCH	Mitral	ebi-a-GCST	nlYUdJ	424022	rs7607996	-0,06933	0,08184	0,396886
MCH	Mitral	ebi-a-GCST	nlYUdJ	424022	rs7617840	-0,07053	0,081973	0,389592
MCH	Mitral	ebi-a-GCST	nlYUdJ	424022	rs762679	-0,07116	0,081972	0,385334
MCH	Mitral	ebi-a-GCST	nlYUdJ	424022	rs7642571!	-0,07076	0,081954	0,387895
MCH	Mitral	ebi-a-GCST	nlYUdJ	424022	rs7643833:	-0,07083	0,081962	0,387469
MCH	Mitral	ebi-a-GCST	nlYUdJ	424022	rs7666468(	-0,07214	0,081934	0,378582
MCH	Mitral	ebi-a-GCST	nlYUdJ	424022	rs7696175	-0,07106	0,081989	0,386137
MCH	Mitral	ebi-a-GCST	nlYUdJ	424022	rs7700687	-0,07215	0,081953	0,378683
MCH	Mitral	ebi-a-GCST	nlYUdJ	424022	rs7705509!	-0,06653	0,081933	0,416769
MCH	Mitral	ebi-a-GCST	nlYUdJ	424022	rs7705526	-0,06223	0,081924	0,447478
MCH	Mitral	ebi-a-GCST	nlYUdJ	424022	rs7711581(	-0,0715	0,081968	0,383065
MCH	Mitral	ebi-a-GCST	nlYUdJ	424022	rs7754216:	-0,0733	0,082003	0,371367
MCH	Mitral	ebi-a-GCST	nlYUdJ	424022	rs7777063	-0,07428	0,082051	0,365313
MCH	Mitral	ebi-a-GCST	nlYUdJ	424022	rs7805890	-0,07681	0,081859	0,348091
MCH	Mitral	ebi-a-GCST	nlYUdJ	424022	rs7808168!	-0,07557	0,08158	0,3543
MCH	Mitral	ebi-a-GCST	nlYUdJ	424022	rs782725	-0,07	0,081988	0,393219
MCH	Mitral	ebi-a-GCST	nlYUdJ	424022	rs7837822:	-0,067	0,08179	0,412722
MCH	Mitral	ebi-a-GCST	nlYUdJ	424022	rs7840612!	-0,07163	0,081961	0,38213
MCH	Mitral	ebi-a-GCST	nlYUdJ	424022	rs7865022!	-0,07211	0,081957	0,378971
MCH	Mitral	ebi-a-GCST	nlYUdJ	424022	rs7867137	-0,072	0,081967	0,379747
MCH	Mitral	ebi-a-GCST	nlYUdJ	424022	rs7869455:	-0,07125	0,081976	0,384784
MCH	Mitral	ebi-a-GCST	nlYUdJ	424022	rs7874418	-0,0727	0,082334	0,377254
MCH	Mitral	ebi-a-GCST	nlYUdJ	424022	rs7888826:	-0,07069	0,081973	0,388497
MCH	Mitral	ebi-a-GCST	nlYUdJ	424022	rs7891208(	-0,11655	0,083745	0,164019
MCH	Mitral	ebi-a-GCST	nlYUdJ	424022	rs7906958	-0,07301	0,081853	0,372381
MCH	Mitral	ebi-a-GCST	nlYUdJ	424022	rs7937415	-0,07094	0,082	0,386985
MCH	Mitral	ebi-a-GCST	nlYUdJ	424022	rs7945994	-0,06763	0,081554	0,406943
MCH	Mitral	ebi-a-GCST	nlYUdJ	424022	rs7978096:	-0,07045	0,081953	0,389958
MCH	Mitral	ebi-a-GCST	nlYUdJ	424022	rs8012643	-0,07122	0,082034	0,385265
MCH	Mitral	ebi-a-GCST	nlYUdJ	424022	rs8013143	-0,07515	0,081997	0,359385
MCH	Mitral	ebi-a-GCST	nlYUdJ	424022	rs8020091	-0,07243	0,081918	0,376584
MCH	Mitral	ebi-a-GCST	nlYUdJ	424022	rs8062719	-0,07206	0,082042	0,379762
MCH	Mitral	ebi-a-GCST	nlYUdJ	424022	rs806704	-0,06898	0,081888	0,399551
MCH	Mitral	ebi-a-GCST	nlYUdJ	424022	rs8074949	-0,07456	0,081678	0,361291
MCH	Mitral	ebi-a-GCST	nlYUdJ	424022	rs8110787	-0,07591	0,082671	0,358517
MCH	Mitral	ebi-a-GCST	nlYUdJ	424022	rs8176751	-0,0693	0,08197	0,397867
MCH	Mitral	ebi-a-GCST	nlYUdJ	424022	rs854775	-0,07228	0,081952	0,377801
MCH	Mitral	ebi-a-GCST	nlYUdJ	424022	rs869785	-0,06188	0,082267	0,451926
MCH	Mitral	ebi-a-GCST	nlYUdJ	424022	rs875742	-0,07108	0,082043	0,386276
MCH	Mitral	ebi-a-GCST	nlYUdJ	424022	rs877908	-0,07663	0,083861	0,360858
MCH	Mitral	ebi-a-GCST	nlYUdJ	424022	rs8887	-0,06401	0,08262	0,438513
MCH	Mitral	ebi-a-GCST	nlYUdJ	424022	rs911000	-0,07372	0,081852	0,367744
MCH	Mitral	ebi-a-GCST	nlYUdJ	424022	rs920112	-0,06868	0,081889	0,401654
MCH	Mitral	ebi-a-GCST	nlYUdJ	424022	rs9258357	-0,07061	0,082224	0,390505

MCH	Mitral	ebi-a-GCST	nlYUdJ	424022	rs9260419	-0,07185	0,082029	0,381103
MCH	Mitral	ebi-a-GCST	nlYUdJ	424022	rs9264277	-0,07013	0,082008	0,392453
MCH	Mitral	ebi-a-GCST	nlYUdJ	424022	rs9287979	-0,07327	0,081891	0,370933
MCH	Mitral	ebi-a-GCST	nlYUdJ	424022	rs9289629	-0,0636	0,081773	0,436714
MCH	Mitral	ebi-a-GCST	nlYUdJ	424022	rs9375124	-0,07013	0,081915	0,391946
MCH	Mitral	ebi-a-GCST	nlYUdJ	424022	rs9381093	-0,07624	0,082915	0,357846
MCH	Mitral	ebi-a-GCST	nlYUdJ	424022	rs9420907	-0,06752	0,081841	0,409385
MCH	Mitral	ebi-a-GCST	nlYUdJ	424022	rs9457800	-0,06978	0,081908	0,394249
MCH	Mitral	ebi-a-GCST	nlYUdJ	424022	rs9464759	-0,06077	0,081469	0,45572
MCH	Mitral	ebi-a-GCST	nlYUdJ	424022	rs9468618	-0,07313	0,081928	0,372069
MCH	Mitral	ebi-a-GCST	nlYUdJ	424022	rs9471708	-0,10481	0,082866	0,205953
MCH	Mitral	ebi-a-GCST	nlYUdJ	424022	rs9487023	-0,06774	0,082873	0,413697
MCH	Mitral	ebi-a-GCST	nlYUdJ	424022	rs9521026	-0,07234	0,081968	0,377512
MCH	Mitral	ebi-a-GCST	nlYUdJ	424022	rs9532563	-0,07078	0,082006	0,388076
MCH	Mitral	ebi-a-GCST	nlYUdJ	424022	rs9577577	-0,07354	0,08184	0,368879
MCH	Mitral	ebi-a-GCST	nlYUdJ	424022	rs9579583	-0,07114	0,081985	0,385563
MCH	Mitral	ebi-a-GCST	nlYUdJ	424022	rs9595815	-0,07094	0,081959	0,386718
MCH	Mitral	ebi-a-GCST	nlYUdJ	424022	rs9676912	-0,07113	0,081969	0,385517
MCH	Mitral	ebi-a-GCST	nlYUdJ	424022	rs9714342	-0,07415	0,081662	0,363873
MCH	Mitral	ebi-a-GCST	nlYUdJ	424022	rs9811850	-0,07462	0,081694	0,360998
MCH	Mitral	ebi-a-GCST	nlYUdJ	424022	rs9833415	-0,06878	0,081778	0,40032
MCH	Mitral	ebi-a-GCST	nlYUdJ	424022	rs9862438	-0,07474	0,081548	0,359382
MCH	Mitral	ebi-a-GCST	nlYUdJ	424022	rs9892942	-0,07109	0,082361	0,388062
MCH	Mitral	ebi-a-GCST	nlYUdJ	424022	rs9921972	-0,07104	0,081962	0,38611
MCH	Mitral	ebi-a-GCST	nlYUdJ	424022	rs9941367	-0,07114	0,081964	0,385427
MCH	Mitral	ebi-a-GCST	nlYUdJ	424022	rs994729	-0,06959	0,081905	0,395493
MCH	Mitral	ebi-a-GCST	nlYUdJ	424022	All	-0,07128	0,081845	0,383806
MCHC	Mitral	ebi-a-GCST	X3XZqd	424022	rs1002331	-0,14715	0,138186	0,286947
MCHC	Mitral	ebi-a-GCST	X3XZqd	424022	rs1009910	-0,14956	0,138247	0,279328
MCHC	Mitral	ebi-a-GCST	X3XZqd	424022	rs1012777	-0,135	0,13825	0,328828
MCHC	Mitral	ebi-a-GCST	X3XZqd	424022	rs1016059	-0,14443	0,138288	0,296294
MCHC	Mitral	ebi-a-GCST	X3XZqd	424022	rs1040553	-0,13971	0,138229	0,31217
MCHC	Mitral	ebi-a-GCST	X3XZqd	424022	rs1042391	-0,12564	0,138814	0,365416
MCHC	Mitral	ebi-a-GCST	X3XZqd	424022	rs1075728	-0,14397	0,138201	0,297542
MCHC	Mitral	ebi-a-GCST	X3XZqd	424022	rs1087623	-0,13521	0,138217	0,327968
MCHC	Mitral	ebi-a-GCST	X3XZqd	424022	rs1092339	-0,14007	0,138315	0,311214
MCHC	Mitral	ebi-a-GCST	X3XZqd	424022	rs1108582	-0,14952	0,138782	0,281317
MCHC	Mitral	ebi-a-GCST	X3XZqd	424022	rs1114532	-0,14419	0,138247	0,29697
MCHC	Mitral	ebi-a-GCST	X3XZqd	424022	rs1121896	-0,14007	0,138279	0,31109
MCHC	Mitral	ebi-a-GCST	X3XZqd	424022	rs1126431	-0,1414	0,138185	0,306187
MCHC	Mitral	ebi-a-GCST	X3XZqd	424022	rs1128756	-0,15345	0,138715	0,268629
MCHC	Mitral	ebi-a-GCST	X3XZqd	424022	rs1138818	-0,14242	0,138209	0,302788
MCHC	Mitral	ebi-a-GCST	X3XZqd	424022	rs1142476	-0,1551	0,138583	0,263049
MCHC	Mitral	ebi-a-GCST	X3XZqd	424022	rs1153566	-0,13424	0,138207	0,331413
MCHC	Mitral	ebi-a-GCST	X3XZqd	424022	rs1156897	-0,14613	0,138288	0,290644
MCHC	Mitral	ebi-a-GCST	X3XZqd	424022	rs1158490	-0,14783	0,138397	0,285456
MCHC	Mitral	ebi-a-GCST	X3XZqd	424022	rs1164302	-0,14289	0,138215	0,301218
MCHC	Mitral	ebi-a-GCST	X3XZqd	424022	rs1166819	-0,14099	0,138716	0,309427
MCHC	Mitral	ebi-a-GCST	X3XZqd	424022	rs1167629	-0,13701	0,138282	0,321792
MCHC	Mitral	ebi-a-GCST	X3XZqd	424022	rs1173566	-0,14734	0,138429	0,287161

MCHC	Mitral	ebi-a-GCST	X3XZqd	424022	rs1175550	-0,12353	0,139299	0,375182
MCHC	Mitral	ebi-a-GCST	X3XZqd	424022	rs1215035[illegible]	-0,14471	0,138191	0,29502
MCHC	Mitral	ebi-a-GCST	X3XZqd	424022	rs1215102[illegible]	-0,14432	0,138308	0,29674
MCHC	Mitral	ebi-a-GCST	X3XZqd	424022	rs1225576[illegible]	-0,13779	0,138207	0,318774
MCHC	Mitral	ebi-a-GCST	X3XZqd	424022	rs1227707[illegible]	-0,1442	0,138195	0,296747
MCHC	Mitral	ebi-a-GCST	X3XZqd	424022	rs1232867[illegible]	-0,13869	0,138186	0,315546
MCHC	Mitral	ebi-a-GCST	X3XZqd	424022	rs1245147[illegible]	-0,15145	0,138445	0,273971
MCHC	Mitral	ebi-a-GCST	X3XZqd	424022	rs1247422[illegible]	-0,14042	0,138194	0,309564
MCHC	Mitral	ebi-a-GCST	X3XZqd	424022	rs1260606	-0,14642	0,13824	0,289534
MCHC	Mitral	ebi-a-GCST	X3XZqd	424022	rs1271859[illegible]	-0,13833	0,138195	0,31684
MCHC	Mitral	ebi-a-GCST	X3XZqd	424022	rs1272105[illegible]	-0,14483	0,138395	0,295324
MCHC	Mitral	ebi-a-GCST	X3XZqd	424022	rs1276296	-0,13405	0,138197	0,332041
MCHC	Mitral	ebi-a-GCST	X3XZqd	424022	rs1290466[illegible]	-0,13013	0,13824	0,346527
MCHC	Mitral	ebi-a-GCST	X3XZqd	424022	rs1306632[illegible]	-0,13768	0,138416	0,3199
MCHC	Mitral	ebi-a-GCST	X3XZqd	424022	rs1309237[illegible]	-0,13874	0,138314	0,315809
MCHC	Mitral	ebi-a-GCST	X3XZqd	424022	rs1316500[illegible]	-0,14135	0,138194	0,306396
MCHC	Mitral	ebi-a-GCST	X3XZqd	424022	rs131785	-0,14491	0,13819	0,294347
MCHC	Mitral	ebi-a-GCST	X3XZqd	424022	rs1330678[illegible]	-0,17178	0,141103	0,223454
MCHC	Mitral	ebi-a-GCST	X3XZqd	424022	rs1340849[illegible]	-0,14613	0,138187	0,290284
MCHC	Mitral	ebi-a-GCST	X3XZqd	424022	rs1398883	-0,14114	0,138257	0,307314
MCHC	Mitral	ebi-a-GCST	X3XZqd	424022	rs1403449[illegible]	-0,14173	0,138191	0,305078
MCHC	Mitral	ebi-a-GCST	X3XZqd	424022	rs1414946[illegible]	-0,15066	0,138719	0,277438
MCHC	Mitral	ebi-a-GCST	X3XZqd	424022	rs1419114	-0,15103	0,138987	0,277198
MCHC	Mitral	ebi-a-GCST	X3XZqd	424022	rs1441963[illegible]	-0,14138	0,138193	0,30628
MCHC	Mitral	ebi-a-GCST	X3XZqd	424022	rs1461102	-0,14826	0,138205	0,283375
MCHC	Mitral	ebi-a-GCST	X3XZqd	424022	rs1477302[illegible]	-0,14455	0,138232	0,295699
MCHC	Mitral	ebi-a-GCST	X3XZqd	424022	rs1500865[illegible]	-0,13567	0,138175	0,326168
MCHC	Mitral	ebi-a-GCST	X3XZqd	424022	rs1655300	-0,14685	0,13821	0,288006
MCHC	Mitral	ebi-a-GCST	X3XZqd	424022	rs1668871	-0,14702	0,138554	0,288653
MCHC	Mitral	ebi-a-GCST	X3XZqd	424022	rs1701172[illegible]	-0,13899	0,138346	0,315063
MCHC	Mitral	ebi-a-GCST	X3XZqd	424022	rs1711671[illegible]	-0,15213	0,138234	0,271099
MCHC	Mitral	ebi-a-GCST	X3XZqd	424022	rs174559	-0,13929	0,138351	0,314038
MCHC	Mitral	ebi-a-GCST	X3XZqd	424022	rs1751482	-0,13442	0,138187	0,330672
MCHC	Mitral	ebi-a-GCST	X3XZqd	424022	rs1775869[illegible]	-0,14031	0,138196	0,309956
MCHC	Mitral	ebi-a-GCST	X3XZqd	424022	rs1786782[illegible]	-0,138	0,138198	0,317997
MCHC	Mitral	ebi-a-GCST	X3XZqd	424022	rs1800562	-0,1475	0,144353	0,306883
MCHC	Mitral	ebi-a-GCST	X3XZqd	424022	rs1810193	-0,11685	0,138454	0,39869
MCHC	Mitral	ebi-a-GCST	X3XZqd	424022	rs1830348[illegible]	-0,13456	0,138179	0,330162
MCHC	Mitral	ebi-a-GCST	X3XZqd	424022	rs1986656	-0,14026	0,138211	0,310204
MCHC	Mitral	ebi-a-GCST	X3XZqd	424022	rs2022003	-0,17738	0,141075	0,208638
MCHC	Mitral	ebi-a-GCST	X3XZqd	424022	rs2060779	-0,12985	0,138291	0,347762
MCHC	Mitral	ebi-a-GCST	X3XZqd	424022	rs2119151	-0,14267	0,138189	0,301882
MCHC	Mitral	ebi-a-GCST	X3XZqd	424022	rs2139559	-0,14181	0,138214	0,304874
MCHC	Mitral	ebi-a-GCST	X3XZqd	424022	rs2285005	-0,13782	0,13846	0,31956
MCHC	Mitral	ebi-a-GCST	X3XZqd	424022	rs228909	-0,1422	0,138295	0,303827
MCHC	Mitral	ebi-a-GCST	X3XZqd	424022	rs2291079	-0,15132	0,138211	0,273587
MCHC	Mitral	ebi-a-GCST	X3XZqd	424022	rs2468024	-0,14693	0,138394	0,288382
MCHC	Mitral	ebi-a-GCST	X3XZqd	424022	rs247826	-0,13969	0,138234	0,312245
MCHC	Mitral	ebi-a-GCST	X3XZqd	424022	rs2562163	-0,13475	0,138594	0,33092
MCHC	Mitral	ebi-a-GCST	X3XZqd	424022	rs2723514	-0,14602	0,138246	0,290861

MCHC	Mitral	ebi-a-GCST	X3XZqd	424022	rs2746050	-0,13803	0,138261	0,318105
MCHC	Mitral	ebi-a-GCST	X3XZqd	424022	rs2811708	-0,14711	0,138275	0,287389
MCHC	Mitral	ebi-a-GCST	X3XZqd	424022	rs2836092!	-0,13851	0,138187	0,316165
MCHC	Mitral	ebi-a-GCST	X3XZqd	424022	rs2842375	-0,13913	0,138193	0,314053
MCHC	Mitral	ebi-a-GCST	X3XZqd	424022	rs2884789!	-0,13854	0,139136	0,319394
MCHC	Mitral	ebi-a-GCST	X3XZqd	424022	rs320370	-0,15109	0,138209	0,274302
MCHC	Mitral	ebi-a-GCST	X3XZqd	424022	rs3416410!	-0,14903	0,143169	0,297901
MCHC	Mitral	ebi-a-GCST	X3XZqd	424022	rs3443094!	-0,14741	0,138302	0,286478
MCHC	Mitral	ebi-a-GCST	X3XZqd	424022	rs3451194:	-0,15268	0,138985	0,271972
MCHC	Mitral	ebi-a-GCST	X3XZqd	424022	rs3460727!	-0,13902	0,138223	0,314537
MCHC	Mitral	ebi-a-GCST	X3XZqd	424022	rs34651	-0,14804	0,138388	0,284742
MCHC	Mitral	ebi-a-GCST	X3XZqd	424022	rs3485686!	-0,14225	0,13822	0,303415
MCHC	Mitral	ebi-a-GCST	X3XZqd	424022	rs3491446:	-0,1382	0,138219	0,31739
MCHC	Mitral	ebi-a-GCST	X3XZqd	424022	rs3557796:	-0,12781	0,138271	0,355296
MCHC	Mitral	ebi-a-GCST	X3XZqd	424022	rs35592	-0,15009	0,138199	0,277462
MCHC	Mitral	ebi-a-GCST	X3XZqd	424022	rs3560260!	-0,13852	0,138271	0,316445
MCHC	Mitral	ebi-a-GCST	X3XZqd	424022	rs3746444:	-0,13358	0,138223	0,333831
MCHC	Mitral	ebi-a-GCST	X3XZqd	424022	rs3760047	-0,13744	0,139706	0,325234
MCHC	Mitral	ebi-a-GCST	X3XZqd	424022	rs3772570(	-0,13105	0,138233	0,343124
MCHC	Mitral	ebi-a-GCST	X3XZqd	424022	rs3811444	-0,14455	0,138943	0,298163
MCHC	Mitral	ebi-a-GCST	X3XZqd	424022	rs3812442	-0,14254	0,138196	0,302339
MCHC	Mitral	ebi-a-GCST	X3XZqd	424022	rs3827181	-0,13735	0,138204	0,320316
MCHC	Mitral	ebi-a-GCST	X3XZqd	424022	rs3856396	-0,14892	0,138263	0,281441
MCHC	Mitral	ebi-a-GCST	X3XZqd	424022	rs415895	-0,12908	0,138619	0,351747
MCHC	Mitral	ebi-a-GCST	X3XZqd	424022	rs4304949	-0,14911	0,138202	0,280631
MCHC	Mitral	ebi-a-GCST	X3XZqd	424022	rs444329	-0,14126	0,138189	0,306665
MCHC	Mitral	ebi-a-GCST	X3XZqd	424022	rs458741	-0,14571	0,138228	0,29183
MCHC	Mitral	ebi-a-GCST	X3XZqd	424022	rs4594828	-0,13073	0,138195	0,34416
MCHC	Mitral	ebi-a-GCST	X3XZqd	424022	rs4652305	-0,14086	0,138185	0,308044
MCHC	Mitral	ebi-a-GCST	X3XZqd	424022	rs4704959	-0,14593	0,138262	0,291223
MCHC	Mitral	ebi-a-GCST	X3XZqd	424022	rs4727976	-0,14513	0,138256	0,293829
MCHC	Mitral	ebi-a-GCST	X3XZqd	424022	rs4737010	-0,14755	0,14137	0,296623
MCHC	Mitral	ebi-a-GCST	X3XZqd	424022	rs4760682	-0,15303	0,138205	0,268171
MCHC	Mitral	ebi-a-GCST	X3XZqd	424022	rs4814776	-0,14208	0,13823	0,304033
MCHC	Mitral	ebi-a-GCST	X3XZqd	424022	rs4894807	-0,13571	0,138193	0,326074
MCHC	Mitral	ebi-a-GCST	X3XZqd	424022	rs4944832	-0,14846	0,138191	0,282681
MCHC	Mitral	ebi-a-GCST	X3XZqd	424022	rs4947490	-0,14895	0,138201	0,281144
MCHC	Mitral	ebi-a-GCST	X3XZqd	424022	rs4984899	-0,14135	0,138417	0,307179
MCHC	Mitral	ebi-a-GCST	X3XZqd	424022	rs553963	-0,14548	0,138214	0,292545
MCHC	Mitral	ebi-a-GCST	X3XZqd	424022	rs5570927:	-0,14559	0,138224	0,292213
MCHC	Mitral	ebi-a-GCST	X3XZqd	424022	rs5585247(	-0,14787	0,138188	0,28458
MCHC	Mitral	ebi-a-GCST	X3XZqd	424022	rs5586471!	-0,14358	0,138195	0,298818
MCHC	Mitral	ebi-a-GCST	X3XZqd	424022	rs563995	-0,15807	0,138633	0,2542
MCHC	Mitral	ebi-a-GCST	X3XZqd	424022	rs5754100	-0,14329	0,138235	0,299934
MCHC	Mitral	ebi-a-GCST	X3XZqd	424022	rs6005859	-0,14086	0,138202	0,308089
MCHC	Mitral	ebi-a-GCST	X3XZqd	424022	rs6029516	-0,14115	0,138187	0,307049
MCHC	Mitral	ebi-a-GCST	X3XZqd	424022	rs6075741:	-0,14425	0,138299	0,296922
MCHC	Mitral	ebi-a-GCST	X3XZqd	424022	rs6084653	-0,12982	0,138247	0,347724
MCHC	Mitral	ebi-a-GCST	X3XZqd	424022	rs6161199:	-0,1341	0,138228	0,33196
MCHC	Mitral	ebi-a-GCST	X3XZqd	424022	rs6202348!	-0,14047	0,138187	0,309398

MCHC	Mitral	ebi-a-GCST	X3XZqd	424022	rs6255097!	-0,14917	0,138744	0,282322
MCHC	Mitral	ebi-a-GCST	X3XZqd	424022	rs6439359	-0,15378	0,138251	0,266013
MCHC	Mitral	ebi-a-GCST	X3XZqd	424022	rs6494533	-0,14834	0,138322	0,283514
MCHC	Mitral	ebi-a-GCST	X3XZqd	424022	rs6507690	-0,13467	0,138331	0,330287
MCHC	Mitral	ebi-a-GCST	X3XZqd	424022	rs6557914	-0,13974	0,138243	0,312101
MCHC	Mitral	ebi-a-GCST	X3XZqd	424022	rs6659327.	-0,10024	0,14009	0,47426
MCHC	Mitral	ebi-a-GCST	X3XZqd	424022	rs6682461.	-0,14517	0,13822	0,293577
MCHC	Mitral	ebi-a-GCST	X3XZqd	424022	rs6683362.	-0,14336	0,138236	0,299688
MCHC	Mitral	ebi-a-GCST	X3XZqd	424022	rs6727692!	-0,14301	0,138217	0,300818
MCHC	Mitral	ebi-a-GCST	X3XZqd	424022	rs6730558	-0,15277	0,138357	0,269528
MCHC	Mitral	ebi-a-GCST	X3XZqd	424022	rs6795085	-0,14744	0,138191	0,286001
MCHC	Mitral	ebi-a-GCST	X3XZqd	424022	rs710218	-0,14623	0,138436	0,290845
MCHC	Mitral	ebi-a-GCST	X3XZqd	424022	rs7247659	-0,13763	0,138412	0,320041
MCHC	Mitral	ebi-a-GCST	X3XZqd	424022	rs7264328(	-0,1445	0,138188	0,295717
MCHC	Mitral	ebi-a-GCST	X3XZqd	424022	rs7267950-	-0,13873	0,138193	0,315433
MCHC	Mitral	ebi-a-GCST	X3XZqd	424022	rs7283906(	-0,1504	0,138448	0,277339
MCHC	Mitral	ebi-a-GCST	X3XZqd	424022	rs7291756	-0,14163	0,138197	0,305421
MCHC	Mitral	ebi-a-GCST	X3XZqd	424022	rs7298027!	-0,14396	0,138236	0,29768
MCHC	Mitral	ebi-a-GCST	X3XZqd	424022	rs7305893(	-0,14141	0,138182	0,306143
MCHC	Mitral	ebi-a-GCST	X3XZqd	424022	rs7321000!	-0,13699	0,138774	0,323577
MCHC	Mitral	ebi-a-GCST	X3XZqd	424022	rs7342323!	-0,14903	0,138367	0,281443
MCHC	Mitral	ebi-a-GCST	X3XZqd	424022	rs7385804	-0,16329	0,139476	0,241689
MCHC	Mitral	ebi-a-GCST	X3XZqd	424022	rs7437563.	-0,14223	0,138236	0,303515
MCHC	Mitral	ebi-a-GCST	X3XZqd	424022	rs7441723!	-0,132	0,138891	0,341919
MCHC	Mitral	ebi-a-GCST	X3XZqd	424022	rs7578982	-0,13718	0,13821	0,320932
MCHC	Mitral	ebi-a-GCST	X3XZqd	424022	rs7630745	-0,1488	0,138206	0,281643
MCHC	Mitral	ebi-a-GCST	X3XZqd	424022	rs7672657(	-0,14251	0,138199	0,302459
MCHC	Mitral	ebi-a-GCST	X3XZqd	424022	rs7672851!	-0,1561	0,138274	0,258944
MCHC	Mitral	ebi-a-GCST	X3XZqd	424022	rs7702189	-0,14431	0,138593	0,297765
MCHC	Mitral	ebi-a-GCST	X3XZqd	424022	rs7737584(	-0,15341	0,13825	0,267143
MCHC	Mitral	ebi-a-GCST	X3XZqd	424022	rs7777063	-0,14791	0,138334	0,284956
MCHC	Mitral	ebi-a-GCST	X3XZqd	424022	rs7801451(	-0,14538	0,13821	0,292845
MCHC	Mitral	ebi-a-GCST	X3XZqd	424022	rs7826556!	-0,14956	0,1382	0,279153
MCHC	Mitral	ebi-a-GCST	X3XZqd	424022	rs7857288.	-0,14307	0,138185	0,300509
MCHC	Mitral	ebi-a-GCST	X3XZqd	424022	rs7857294(	-0,14238	0,138247	0,303053
MCHC	Mitral	ebi-a-GCST	X3XZqd	424022	rs7890903.	-0,13106	0,138318	0,343363
MCHC	Mitral	ebi-a-GCST	X3XZqd	424022	rs7897215.	-0,14713	0,138228	0,287132
MCHC	Mitral	ebi-a-GCST	X3XZqd	424022	rs7923316!	-0,14596	0,138185	0,290855
MCHC	Mitral	ebi-a-GCST	X3XZqd	424022	rs7997757!	-0,13877	0,138265	0,315561
MCHC	Mitral	ebi-a-GCST	X3XZqd	424022	rs8047112	-0,14363	0,138224	0,298768
MCHC	Mitral	ebi-a-GCST	X3XZqd	424022	rs806970	-0,13298	0,138346	0,336444
MCHC	Mitral	ebi-a-GCST	X3XZqd	424022	rs8073068	-0,15611	0,138226	0,258727
MCHC	Mitral	ebi-a-GCST	X3XZqd	424022	rs812097	-0,1646	0,138294	0,233973
MCHC	Mitral	ebi-a-GCST	X3XZqd	424022	rs8176693	-0,15262	0,138501	0,270482
MCHC	Mitral	ebi-a-GCST	X3XZqd	424022	rs818508	-0,1419	0,138187	0,304489
MCHC	Mitral	ebi-a-GCST	X3XZqd	424022	rs852424	-0,15183	0,138262	0,272132
MCHC	Mitral	ebi-a-GCST	X3XZqd	424022	rs855791	-0,12108	0,14346	0,398653
MCHC	Mitral	ebi-a-GCST	X3XZqd	424022	rs8887	-0,13145	0,139157	0,344865
MCHC	Mitral	ebi-a-GCST	X3XZqd	424022	rs921968	-0,15591	0,138448	0,260127
MCHC	Mitral	ebi-a-GCST	X3XZqd	424022	rs9258357	-0,14182	0,138696	0,306516

MCHC	Mitral	ebi-a-GCST	X3XZqd	424022	rs9268137	-0,14216	0,138225	0,303722
MCHC	Mitral	ebi-a-GCST	X3XZqd	424022	rs9381093	-0,1416	0,138223	0,305648
MCHC	Mitral	ebi-a-GCST	X3XZqd	424022	rs941718	-0,14826	0,138243	0,283505
MCHC	Mitral	ebi-a-GCST	X3XZqd	424022	rs941864	-0,14382	0,138191	0,297985
MCHC	Mitral	ebi-a-GCST	X3XZqd	424022	rs9487023	-0,13814	0,138952	0,320151
MCHC	Mitral	ebi-a-GCST	X3XZqd	424022	rs9505274	-0,14162	0,138185	0,305434
MCHC	Mitral	ebi-a-GCST	X3XZqd	424022	rs964184	-0,15718	0,138663	0,257001
MCHC	Mitral	ebi-a-GCST	X3XZqd	424022	rs9646283	-0,13659	0,138229	0,323098
MCHC	Mitral	ebi-a-GCST	X3XZqd	424022	rs9850274	-0,14367	0,138468	0,299474
MCHC	Mitral	ebi-a-GCST	X3XZqd	424022	rs9870317	-0,13648	0,138268	0,32362
MCHC	Mitral	ebi-a-GCST	X3XZqd	424022	rs9891699	-0,14507	0,138319	0,294258
MCHC	Mitral	ebi-a-GCST	X3XZqd	424022	rs9892222	-0,14448	0,138213	0,295854
MCHC	Mitral	ebi-a-GCST	X3XZqd	424022	rs9923936	-0,14133	0,138196	0,306461
MCHC	Mitral	ebi-a-GCST	X3XZqd	424022	rs9924236	-0,13081	0,138377	0,344486
MCHC	Mitral	ebi-a-GCST	X3XZqd	424022	All	-0,14282	0,138101	0,301053
