# Supplementary material for: Relação Causal entre Características das Células Sanguíneas e Doença Cardíaca Valvar: Um Estudo de Randomização Mendeliana com Duas Amostras
Source: Arq Bras Cardiol. 2026 Apr 14;123(3):e20250063. [Article in Portuguese] doi: 10.36660/abc.20250063 (PMC13128221; doi:10.36660/abc.20250063)
Supplement: Translation

# Translation: The Causal Relationship between Blood Cell Traits and Valvular Heart Disease: A Two-Sample Mendelian Randomization Study

**DOI:** 10.36660/abc.20250063i

**Authors:** Xiaoli Wu, Yufeng Li, Jinyu Tian

**Affiliations:** 1 China Department of Ultrasound - Panzhihua Central Hospital, Panzhihua – China

**Keywords:** Blood Cells, Heart Valve Diseases, Mitral Valve Insufficiency, Mendelian Randomization Analysis

## Abstract

**Background:**

The pathogenesis of valvular heart disease (VHD) remains unclear, highlighting the need for further research on risk factors.

**Objectives:**

To explore the potential causal links between blood cell traits and four types of VHD: rheumatic heart disease, mitral valve disease, aortic valve disease, and tricuspid valve disease.

**Methods:**

This study utilized summary statistics from large-scale GWAS and a two-sample Mendelian randomization approach, analyzing thirteen blood cell traits as exposures and four VHD phenotypes as outcomes. The primary MR method was inverse-variance weighted (IVW), supplemented by MR-Egger, weighted median, and weighted mode methods, with extensive sensitivity analyses to verify result reliability, where a p value < 0.05 was considered statistically significant.

**Results:**

Among the thirteen blood cell traits examined, neutrophil count displayed a positive causal association with mitral valve disease (OR = 1.46, 95% CI: 1.16-1.85, p < 0.001) using the IVW method. This finding was further corroborated by the MR-Egger method (OR = 1.73, 95% CI: 1.09-2.75, p = 0.02), strengthening the evidence for this causal relationship. For this analysis, 253 robust genetic variants were used as instrumental variables. Additionally, mean corpuscular volume (MCV) showed a negative causal association with tricuspid valve disease (OR = 0.65, 95% CI: 0.44-0.96, p = 0.03) using the IVW method, based on 268 instrumental variables. Other blood cell traits did not show significant causal relationships with VHD. A comprehensive sensitivity analysis supported the robustness of our results.

**Conclusions:**

The findings of this study suggest potential causal involvement of neutrophil count and MCV in the development of mitral valve disease and tricuspid valve disease, respectively.

## Introduction

Valvular heart disease (VHD) is a category of cardiac disorders arising from the impaired structure and function of the heart valves, comprising aortic valve disease, mitral valve disease, tricuspid valve disease, and rheumatic heart disease.^1^Like coronary artery disease, valve disorders share many risk factors and pathobiological mechanisms related to inflammation, endothelial dysfunction, and calcification.^2^Due to the growing prevalence of other cardiovascular diseases and the aging population worldwide, VHD has become a notable cause of adverse cardiovascular events, contributing to the global economic burden.^3^Even with advancements in the diagnosis and treatment of VHD, its underlying pathogenesis remains not fully understood, underscoring the need for additional research into potential risk factors and causal mechanisms.

Human blood cells are vital for oxygen transport, hemostasis, and immune responses.^4^Cardiovascular disorders are influenced by inflammation and oxidative stress, which may be reflected in human blood cells traits.^5^Studies have explored the relationship between blood cell traits and cardiovascular outcomes.^6^Elevated white blood cell counts, particularly neutrophils, have been linked to increased cardiovascular risk.^7^Neutrophils contribute to atherosclerosis and plaque destabilization.^8^Monocytes are involved in vascular inflammation and remodeling, affecting cardiovascular disease risk.^9^Red blood cell (RBC) traits such as mean corpuscular volume (MCV), hemoglobin concentration, and red cell distribution width (RDW) have been investigated in cardiovascular diseases.^10^Lower MCV and hemoglobin levels may increase cardiovascular risk due to impaired oxygen delivery.^11^Higher RDW has been associated with adverse cardiovascular outcomes.^12^Biomarkers, including hs-CRP, RDW, and RBC parameters, have shown potential as predictors of complications in valve disease patients.^13^

Despite the exploration of associations between blood cell traits and various cardiovascular diseases in several observational studies, the specific causal relationships between these traits and subtypes of VHD remain poorly understood. A powerful analytical approach, Mendelian randomization (MR), utilizes genetic variants as instrumental variables (IVs) to evaluate potential causal associations between exposures and outcomes.^14^MR analyses leverage the random allocation of genetic variants during gamete formation to address issues of reverse causation and minimize confounding, providing more robust evidence for causality compared to traditional observational studies.^15^Using a two-sample MR design, we investigate the causal associations between 13 blood cell traits and 4 major types of VHD in this study.

## Methods

### Study design

This study, a two-sample MR study, relies on three instrumental variable assumptions^16^( Central Illustration ). First, at least one of the exposures is associated with the genetic variant. Second, none of the confounders of each exposure-outcome association is associated with the variant. Third, given the exposures and confounders, the variant is unrelated to the outcome. The reporting of this study strictly adhered to the Reporting of Observational Studies in Epidemiology using Mendelian Randomization (STROBE-MR).^17 , 18^

### Data sources

From the Blood Cell Consortium (BCX), we obtained summary statistics for the exposure instrument, drawn from a recent large-scale GWAS on blood cell traits, which involved 563,085 individuals of European ancestry.^19^From this GWAS, we acquired genetic variants associated with circulating levels of white blood cell, lymphocyte, monocyte, neutrophil, eosinophil, basophil, platelet, RBC count, hematocrit, hemoglobin, MCV, mean corpuscular hemoglobin, and mean corpuscular hemoglobin concentration. The application of fast GWA or fast GWA GLMM to 3000 traits in 456,422 array-genotyped and 49,960 whole-exome-sequenced individuals of European ancestry from the UK Biobank was conducted by Jiang LD et al.^20^Available for download from the fast GWA data portal (https://yanglab.westlake.edu.cn/data/ukb_fastgwa/imp_binary/), the GWAS summary data associated with VHD cover rheumatic disease of the heart valves (275 cases and 456,073 controls), mitral valve disease (889 cases and 455,459 controls), aortic valve disease (557 cases and 455,791 controls), and tricuspid valve disease (135 cases and 456,213 controls). The disease data are derived from the UK Biobank and are defined using PheCode. The detailed information for all GWAS data could be found in Supplementary Supplementary Table 1 .

**Table 1:** – Association between blood cell traits and VHD

No	Exposure	Outcome	N.SNPs	Methods	OR (95% CI)	or_lci95	or_uci95	p value
1	Baso	Mitral	152	MR-Egger	1.69	0.82	3.48	0.16
			152	Weighted Median	1.55	0.87	2.75	0.13
			152	IVW	1.27	0.89	1.81	0.19
			152	Weighted Mode	1.53	0.74	3.17	0.25
2	Eosino		383	MR-Egger	0.99	0.67	1.45	0.94
			383	Weighted Median	0.99	0.69	1.42	0.97
			383	IVW	0.97	0.79	1.19	0.77
			383	Weighted Mode	1.17	0.73	1.89	0.52
3	Lym		398	MR-Egger	0.79	0.50	1.25	0.32
			398	Weighted Median	0.96	0.66	1.40	0.83
			398	IVW	1.01	0.81	1.25	0.92
			398	Weighted Mode	0.89	0.54	1.48	0.66
4	Mono		479	MR-Egger	1.06	0.80	1.40	0.69
			479	Weighted Median	0.84	0.61	1.15	0.27
			479	IVW	1.00	0.84	1.19	0.99
			479	Weighted Mode	1.02	0.74	1.41	0.91
5	Neutro		347	MR-Egger	1.73	1.09	2.75	0.02
			347	Weighted Median	1.49	0.94	2.34	0.09
			347	IVW	1.46	1.16	1.85	0.00
			347	Weighted Mode	1.50	0.90	2.48	0.12
6	Plt		478	MR-Egger	0.91	0.67	1.21	0.51
			478	Weighted Median	0.94	0.70	1.27	0.71
			478	IVW	0.96	0.81	1.14	0.65
			478	Weighted Mode	0.96	0.70	1.32	0.82
7	RBC		410	MR-Egger	1.03	0.70	1.53	0.87
			410	Weighted Median	1.04	0.71	1.51	0.86
			410	IVW	1.09	0.89	1.34	0.41
			410	Weighted Mode	0.90	0.62	1.30	0.57
8	WBC		387	MR-Egger	1.93	1.19	3.13	0.01
			387	Weighted Median	1.18	0.79	1.77	0.42
			387	IVW	1.21	0.96	1.53	0.11
			387	Weighted Mode	1.30	0.79	2.16	0.30
9	Ht		338	MR-Egger	0.79	0.49	1.28	0.34
			338	Weighted Median	0.77	0.52	1.15	0.20
			338	IVW	0.89	0.69	1.15	0.38
			338	Weighted Mode	0.69	0.46	1.04	0.07
10	Hb		362	MR-Egger	0.88	0.58	1.35	0.56
			362	Weighted Median	0.90	0.60	1.34	0.59
			362	IVW	0.92	0.73	1.15	0.46
			362	Weighted Mode	0.85	0.56	1.29	0.44
11	MCV		430	MR-Egger	0.82	0.64	1.05	0.11
			430	Weighted Median	0.97	0.76	1.25	0.83
			430	IVW	0.94	0.80	1.10	0.43
			430	Weighted Mode	1.01	0.78	1.30	0.96
12	MCH		400	MR-Egger	0.95	0.73	1.24	0.72
			400	Weighted Median	0.99	0.74	1.32	0.93
			400	IVW	0.93	0.79	1.09	0.38
			400	Weighted Mode	1.12	0.83	1.51	0.45
13	MCHC		187	MR-Egger	1.01	0.64	1.58	0.97
			187	Weighted Median	0.93	0.61	1.43	0.74
			187	IVW	0.87	0.66	1.14	0.30
			187	Weighted Mode	0.97	0.64	1.48	0.90
1	Baso	Tricuspid	152	MR-Egger	1.02	0.16	6.48	0.99
			152	Weighted Median	1.06	0.26	4.33	0.93
			152	IVW	0.76	0.30	1.88	0.55
			152	Weighted Mode	1.51	0.23	9.70	0.67
2	Eosino		383	MR-Egger	0.93	0.34	2.52	0.89
			383	Weighted Median	0.43	0.19	1.00	0.05
			383	IVW	0.78	0.46	1.30	0.34
			383	Weighted Mode	0.44	0.14	1.32	0.14
3	Lym		398	MR-Egger	0.76	0.24	2.42	0.64
			398	Weighted Median	0.79	0.30	2.09	0.64
			398	IVW	1.09	0.63	1.90	0.75
			398	Weighted Mode	0.80	0.21	3.02	0.74
4	Mono		479	MR-Egger	1.38	0.68	2.81	0.38
			479	Weighted Median	1.33	0.61	2.92	0.48
			479	IVW	1.25	0.80	1.94	0.32
			479	Weighted Mode	1.36	0.64	2.89	0.43
5	Neutro		347	MR-Egger	0.85	0.27	2.70	0.79
			347	Weighted Median	0.60	0.20	1.78	0.36
			347	IVW	1.19	0.66	2.12	0.57
			347	Weighted Mode	0.79	0.22	2.77	0.71
6	Plt		478	MR-Egger	0.67	0.32	1.40	0.29
			478	Weighted Median	1.09	0.54	2.20	0.80
			478	IVW	0.86	0.57	1.31	0.49
			478	Weighted Mode	1.24	0.51	2.98	0.63
7	RBC		410	MR-Egger	3.27	1.22	8.74	0.02
			410	Weighted Median	2.44	0.95	6.26	0.06
			410	IVW	1.45	0.87	2.43	0.16
			410	Weighted Mode	3.11	1.11	8.68	0.03
8	WBC		387	MR-Egger	0.91	0.28	2.99	0.87
			387	Weighted Median	0.74	0.28	1.94	0.54
			387	IVW	1.29	0.73	2.29	0.38
			387	Weighted Mode	0.74	0.23	2.37	0.61
9	Ht		338	MR-Egger	1.24	0.39	3.95	0.72
			338	Weighted Median	1.21	0.42	3.52	0.72
			338	IVW	0.76	0.41	1.39	0.37
			338	Weighted Mode	1.58	0.45	5.52	0.47
10	Hb		362	MR-Egger	1.38	0.47	4.02	0.56
			362	Weighted Median	1.13	0.40	3.14	0.82
			362	IVW	0.76	0.42	1.36	0.36
			362	Weighted Mode	1.08	0.34	3.45	0.90
11	MCV		430	MR-Egger	0.48	0.26	0.90	0.02
			430	Weighted Median	0.61	0.31	1.20	0.16
			430	IVW	0.65	0.44	0.96	0.03
			430	Weighted Mode	0.54	0.27	1.08	0.08
12	MCH		400	MR-Egger	0.61	0.31	1.18	0.14
			400	Weighted Median	0.52	0.24	1.10	0.09
			400	IVW	0.75	0.50	1.12	0.16
			400	Weighted Mode	0.54	0.23	1.25	0.15
13	MCHC		187	MR-Egger	1.27	0.40	4.08	0.69
			187	Weighted Median	1.26	0.39	4.05	0.70
			187	IVW	1.17	0.58	2.35	0.67
			187	Weighted Mode	1.34	0.44	4.07	0.61

IVW: inverse-variance weighted; RBC: Red blood cell; MCV: mean corpuscular volume; MCH: mean corpuscular hemoglobin; MCHC: mean corpuscular hemoglobin concentration; WBC: white blood cell; Ht: hematocrit; Hb: hemoglobin; Plt: platelet.

Since the data were obtained from publicly available or ethically approved GWAS that comply with the Helsinki Declaration, no further ethical approval was necessary.

### Selection of IVs

Implementing a uniform set of criteria, our study meticulously selected SNPs as IVs. Encompassing several pivotal phases, the selection paradigm was implemented. During the initiation phase, potential IVs were identified as SNPs demonstrating genome-wide significance (p < 5 × 10^-8^, R^2^< 0.001, kb = 10,000).^21^Following this, a clumping procedure (R^2^< 0.001, window size = 10,000 kb) was conducted using the clump data function, designed to ensure that the selected SNPs were not in linkage disequilibrium (LD) with each other.^22^The F-statistic value was computed for each IV that correlated with exposure. SNPs with an F-value less than or equal to 10 were excluded from the analysis to ensure that only IVs with strong associations were selected.^23^

Before the MR analysis, we performed a harmonization procedure to ensure that the effect of each SNP on the exposure and the outcome corresponded to the same effect allele. We removed palindromic SNPs with intermediate allele frequencies to prevent ambiguity. The effect estimates for all blood cell traits were scaled to represent a per standard deviation (SD) increase, ensuring that the odds ratios for VHD are interpreted consistently across different exposures.

### Statistical analysis

Multiple complementary methods were employed in this study to explore the causal relationship between blood cell traits and VHD, including the inverse variance weighted (IVW) method, the weighted median approach, MR-Egger regression, and weighted mode. Employed as the primary analysis for causal estimates, the IVW method exhibited the highest precision when all IVs were valid.^24^When at least half of the SNPs served as effective IVs, the weighted median approach consistently estimated the causal effect.^23^Employed to confirm the presence of horizontal pleiotropy among IVs, the MR-Egger regression yields an effect estimate of horizontal pleiotropy via its intercept.^25^As complementary analyses, the weighted mode was performed.

Sensitivity analyses were conducted additionally to evaluate the reliability of our findings, wherein the MR-Egger intercept was employed to identify directional horizontal pleiotropy.^26^Following this, the MR pleiotropy residual sum and outlier (MR-PRESSO) test were utilized to identify potential horizontal pleiotropy and subsequently correct it by eliminating outliers.^27^The evaluation of heterogeneity between SNPs was conducted using the Cochran’s Q test.^28^The investigation into whether a single SNP influenced the genetic causal relationship between exposures and outcomes was conducted using the leave-one-out analysis.^29^Using the R software (version 4.3.1), all statistical analyses were carried out with the “TwoSampleMR” and “MR-PRESSO” packages. A two-sided p-value < 0.05 was considered statistically significant for the primary causal estimates.

## Results

### IVs selection

As exposures, a total of 13 blood cell traits were evaluated, while 4 VHD phenotypes were considered as outcomes. We identified a set of robust genetic variants to serve as IVs for each exposure-outcome pair after applying the stringent IV selection criteria (Supplementary Table 2 ). All IVs have an F value of more than 10, indicating they were strong enough to avoid weak instrument bias. All SNPs were found in the outcomes; no proxy SNPs were used. Details regarding the number of SNPs are provided in Supplementary Table 3.

**Table 2:** – Results of the heterogeneity test and the pleiotropy test of instrumental variables

Exposure	Outcome	Heterogeneity		Pleiotropy	
		Q statistics (IVW)	p value	MR-Egger Intercept	p value
Baso	Rheumatic	150.13	0.50	0.028	0.101
Eosino		399.23	0.26	-0.003	0.810
Lym		389.36	0.60	-0.004	0.679
Mono		514.38	0.12	0.006	0.447
Neutro		319.04	0.85	0.018	0.105
Plt		480.83	0.44	0.001	0.930
RBC		440.09	0.14	-0.008	0.449
WBC		440.60	0.03	-0.017	0.133
Ht		337.87	0.48	0.020	0.063
Hb		343.67	0.74	-0.001	0.937
MCV		425.15	0.54	0.007	0.387
MCH		446.84	0.05	0.020	0.033
MCHC		184.43	0.52	0.005	0.698
Baso	Mitral	143.74	0.65	-0.008	0.378
Eosino		331.83	0.97	-0.001	0.929
Lym		373.00	0.80	0.007	0.234
Mono		481.11	0.45	-0.002	0.623
Neutro		367.19	0.21	-0.005	0.407
Plt		507.78	0.16	0.002	0.623
RBC		429.80	0.23	0.002	0.750
WBC		438.76	0.03	-0.014	0.033
Ht		385.16	0.04	0.004	0.567
Hb		370.77	0.35	0.001	0.838
MCV		449.36	0.24	0.007	0.166
MCH		407.96	0.37	-0.001	0.823
MCHC		165.32	0.86	-0.006	0.409
Baso	Aortic	163.77	0.23	-0.006	0.619
Eosino		400.81	0.24	-0.004	0.547
Lym		384.74	0.66	-0.004	0.603
Mono		498.01	0.25	-0.005	0.323
Neutro		393.65	0.04	0.000	0.954
Plt		469.41	0.59	-0.009	0.134
RBC		448.82	0.08	-0.012	0.102
WBC		425.29	0.08	0.000	0.982
Ht		324.57	0.68	-0.007	0.380
Hb		382.41	0.21	-0.009	0.214
MCV		441.29	0.33	-0.008	0.197
MCH		391.36	0.60	0.006	0.339
MCHC		165.56	0.86	0.009	0.319
Baso	Tricuspid	139.49	0.74	-0.009	0.721
Eosino		358.88	0.80	-0.006	0.672
Lym		366.22	0.86	0.011	0.479
Mono		453.54	0.78	-0.004	0.725
Neutro		321.54	0.82	0.010	0.517
Plt		452.79	0.78	0.010	0.418
RBC		389.81	0.74	-0.026	0.058
WBC		398.53	0.32	0.010	0.507
Ht		331.10	0.58	-0.015	0.337
Hb		350.72	0.64	-0.018	0.194
MCV		416.95	0.65	0.014	0.221
MCH		395.75	0.54	0.010	0.438
MCHC		189.32	0.42	-0.003	0.859

IVW: inverse-variance weighted; RBC: Red blood cell; MCV: mean corpuscular volume; MCH: mean corpuscular hemoglobin; MCHC: mean corpuscular hemoglobin concentration; WBC: white blood cell; Ht: hematocrit; Hb: hemoglobin; Plt: platelet.

### MR analyses

The primary IVW analysis revealed a genetically predicted positive causal association between a higher neutrophil count and an increased risk of mitral valve disease ( Table 1 , Figure 1A ). This finding was consistent with the results from the MR-Egger method, strengthening the evidence for this causal relationship ( Table 1 ).

In addition, our analysis identified a genetically predicted negative causal relationship between MCV and the risk of tricuspid valve disease using the IVW method ( Table 1 ). The forest plot ( Figure 1B ) visually represented the causal estimates obtained from individual IVs. Although the weighted median did not reach statistical significance (OR = 0.61, 95% CI: 0.31-1.20, p = 0.16) ( Table 1 ), the MR-Egger estimate supported this association (OR = 0.48, 95% CI: 0.26-0.90, p = 0.02) ( Table 1 ). There was no robust evidence of causal associations for the remaining blood cell traits and VHD ( Table 1 ). The scatter plot ( Figure 1C -D) visualized the estimated effect sizes for SNPs linked to blood cell traits affecting VHD.

**Figure 1:** – Causal associations between blood cell traits and VHD. Forest plots of A) neutrophil count on mitral valve disease; B) MCV on tricuspid valve disease. Scatter plots of C) neutrophil count on mitral valve disease; D) MCV on tricuspid valve disease.

### Sensitivity analyses

Several sensitivity analyses were performed to assess the robustness of our findings and the validity of the MR assumptions. For the associations of neutrophil count with mitral valve disease and MCV with tricuspid valve disease, the Cochran’s Q test did not indicate significant heterogeneity ( Table 2 ). The MR-Egger intercept test showed no evidence of directional horizontal pleiotropy, suggesting that our results are unlikely to be biased by pleiotropic effects ( Table 2 ). Furthermore, no outliers were identified for the neutrophil count-mitral valve disease and MCV-tricuspid valve disease associations by the MR-PRESSO global test, except for the neutrophil count-aortic valve disease pair, where one outlier was detected (Supplementary Table 4). Removing this outlier did not affect the consistency of the causal estimate (OR = 1.13, 95% CI: 0.83-1.52, p = 0.44) (SupplementaryTable 4). Funnel plots excluded the publication biases (Supplementary Figure 1A -B), while the leave-one-out analyses confirmed that the causal estimates were not driven by any single influential SNP, further strengthening the robustness of our findings ( Supplementary Figure 1C-D ).

## Discussion

This comprehensive two-sample MR study investigated the potential causal relationships between 13 blood cell traits and 4 major types of VHD: rheumatic heart disease, mitral valve disease, aortic valve disease, and tricuspid valve disease. A positive causal association between neutrophil count and mitral valve disease risk, along with a negative causal association between MCV and tricuspid valve disease risk, is strongly supported by our findings. Our results, demonstrating consistency across multiple MR methods and robustness in rigorous sensitivity analyses, highlight the strength and reliability of these associations.

Our study revealed a positive causal link between neutrophil count and mitral valve disease risk, consistent with the established role of inflammation in VHD pathogenesis.^30^Neutrophils, as key mediators of inflammation, likely contribute to valvular lesion development through various mechanisms, including endothelial dysfunction, oxidative stress, and extracellular matrix remodeling.^31 , 32^These findings suggest that neutrophil-mediated inflammation may play a crucial role in the initiation and progression of mitral valve disease, highlighting potential therapeutic targets in neutrophil-driven inflammatory pathways.

Interestingly, we did not find evidence for a causal association between neutrophil count and rheumatic heart disease or aortic valve disease risk. This differential effect on VHD subtypes may be explained by distinct pathogenic mechanisms. Rheumatic heart disease is primarily driven by lymphocytes and antibody-mediated mechanisms,^33^M. W.</author></authors><secondary-authors><author>Ferretti, J. J.</author><author>Stevens, D. L.</author><author>Fischetti, V. A.</author></secondary-authors></contributors><auth-address>University of Oklahoma Health Sciences Center, Oklahoma City, OKVA Medical Center, Boise, IDThe Rockefeller University, New York, NYDepartment of Microbiology and Immunology, University of Oklahoma Health Sciences Center, Biomedical Research Center, Oklahoma City, OK</auth-address><titles><title>Post-Streptococcal Autoimmune Sequelae: Rheumatic Fever and Beyond</title><secondary-title>Streptococcus pyogenes: Basic Biology to Clinical Manifestations</secondary-title></titles><dates><year>2016</year></dates><pub-location>Oklahoma City (OK while calcific aortic valve disease is more closely linked to lipid deposition and osteogenic signaling pathways.^34 , 35^

Another intriguing finding is the negative causal association between MCV and tricuspid valve disease risk. Lower MCV, indicative of microcytic anemia, has been associated with various cardiovascular disorders due to increased oxidative stress and impaired oxygen delivery.^10 , 11^The mechanisms underlying this causal link may involve endothelial dysfunction, valvular damage, and altered immune responses.^36 - 38^

Our study did not find robust evidence for causal associations between other blood cell traits and VHD subtypes. This suggests that the causal roles of blood cell traits in VHD pathogenesis may be specific to certain cell types or disease subtypes. Alternatively, other unmeasured factors may play more dominant roles in these VHD conditions.

The potential therapeutic implications of these findings warrant careful consideration. While directly targeting neutrophil levels or MCV may not be feasible in clinical practice, exploring approaches to modulate the underlying pathways could lead to novel therapeutic strategies for VHD management.

Crucial to note are the limitations of our study and the need to interpret the findings with appropriate caution. First, like all MR studies, our results could potentially be influenced by unaccounted biological pleiotropy, though we aimed to rigorously evaluate and mitigate this possibility through complementary MR methods and sensitivity analyses. Second, a key limitation of our study is that the GWAS summary statistics were derived exclusively from individuals of European ancestry. Consequently, our findings may not be generalizable to populations of other ancestries, where genetic architectures and environmental factors may differ. Future research in diverse ancestral groups is essential to validate these causal relationships globally. Third, our findings offer valuable insights into the potential causal roles of neutrophil count and MCV in VHD pathogenesis, yet the specific cellular and molecular pathways involved remain speculative and require further investigation. Fourth, while some pathological features (such as stenosis and regurgitation) are of significant interest and are critical to understanding VHD, we were unable to find sufficient data for these subtypes in the available GWAS resources. As a result, we were unable to analyze these important pathological phenotypes, limiting our ability to fully explore their causal associations. Future research should focus on collecting more granular GWAS data on VHD subtypes and enhancing the detailed recording of different pathological features. Fifth, there exists a potential for sample overlap between the exposure and outcome GWAS datasets, which may introduce bias into two-sample MR analyses, particularly when IVs are weak. However, the IVs selected for our primary analyses exhibited F-statistics significantly exceeding 10, thereby qualifying as strong instruments. Consequently, the likelihood of such overlap substantially influencing our results is minimal. Nonetheless, future investigations utilizing non-overlapping subsets of data are warranted to further mitigate potential biases. Additionally, the VHD phenotypes in this study were defined using PheCodes, which may include clinically heterogeneous conditions. For example, mitral valve disease may encompass both stenosis and regurgitation, conditions with distinct pathologies. Due to the absence of publicly available GWAS data for these specific subtypes, we could not conduct sensitivity analyses on more granular phenotypes. This phenotypic heterogeneity could obscure or dilute subtype-specific causal effects, highlighting the need for future studies with more precise outcome definitions. Despite these limitations, our findings contribute to the growing body of evidence linking blood cell traits to the pathogenesis of cardiovascular disease. The identification of potential causal roles for neutrophil count and MCV in mitral valve disease and tricuspid valve disease, respectively, provides novel insights and encourages further exploration of the underlying biological mechanisms.

## Conclusion

This comprehensive two-sample MR study sheds light on the causal associations between specific blood cell traits and certain VHD subtypes. The positive causal link between neutrophil count and mitral valve disease risk, as well as the negative causal association between MCV and tricuspid valve disease risk, underscores the importance of considering these blood cell traits as potential risk factors and therapeutic targets in the context of VHD. These findings pave the way for future research aimed at translating these insights into improved prevention, early detection, and management strategies for VHD, while also opening new avenues for exploring the underlying biological mechanisms.

## *Supplemental Materials

Supplementary material For Supplementary Figure, please click here. https://abccardiol.org/supplementary-material/2026/12303/2025-0063_supplementary_figure_01.pdf

Supplementary material For Supplementary Tables, please click here. https://abccardiol.org/supplementary-material/2026/12303/2025-0063_supplementary_tables_02.pdf

Supplementary material For Supplementary Table 3, please click here. https://abccardiol.org/supplementary-material/2026/12303/2025-0063_supplementary_Table_03.pdf
